# Atmospheric conditions and composition that influence PM_2.5_ oxidative potential in Beijing, China

**DOI:** 10.5194/acp-21-5549-2021

**Published:** 2021-04-12

**Authors:** Steven J. Campbell, Kate Wolfer, Battist Utinger, Joe Westwood, Zhi-Hui Zhang, Nicolas Bukowiecki, Sarah S. Steimer, Tuan V. Vu, Jingsha Xu, Nicholas Straw, Steven Thomson, Atallah Elzein, Yele Sun, Di Liu, Linjie Li, Pingqing Fu, Alastair C. Lewis, Roy M. Harrison, William J. Bloss, Miranda Loh, Mark R. Miller, Zongbo Shi, Markus Kalberer

**Affiliations:** 1 Department of Environmental Sciences, University of Basel, Basel, Switzerland; 2 Department of Chemistry, University of Cambridge, Cambridge, UK; 3 School of Geography, Earth and Environmental Sciences, University of Birmingham, Birmingham, UK; 4 Centre for Cardiovascular Science, Queen’s Medical Research Institute, University of Edinburgh, Edinburgh, UK; 5 Wolfson Atmospheric Chemistry Laboratories, Department of Chemistry, University of York, York, UK; 6 State Key Laboratory of Atmospheric Boundary Layer Physics and Atmospheric Chemistry, Institute of Atmospheric Physics, Chinese Academy of Sciences, Beijing, China; 7 National Centre for Atmospheric Science, University of York, York, UK; 8 Institute of Surface Earth System Science, Tianjin University, Tianjin, China; 9 Institute of Occupational Medicine, Edinburgh, UK

## Abstract

Epidemiological studies have consistently linked exposure to PM_2.5_ with adverse health effects. The oxidative potential (OP) of aerosol particles has been widely suggested as a measure of their potential toxicity. Several acellular chemical assays are now readily employed to measure OP; however, uncertainty remains regarding the atmospheric conditions and specific chemical components of PM_2.5_ that drive OP. A limited number of studies have simultaneously utilised multiple OP assays with a wide range of concurrent measurements and investigated the seasonality of PM_2.5_ OP. In this work, filter samples were collected in winter 2016 and summer 2017 during the atmospheric pollution and human health in a Chinese megacity campaign (APHH-Beijing), and PM_2.5_ OP was analysed using four acellular methods: ascorbic acid (AA), dithiothreitol (DTT), 2,7-dichlorofluorescin/hydrogen peroxidase (DCFH) and electron paramagnetic resonance spectroscopy (EPR). Each assay reflects different oxidising properties of PM_2.5_, including particle-bound reactive oxygen species (DCFH), superoxide radical production (EPR) and catalytic redox chemistry (DTT/AA), and a combination of these four assays provided a detailed overall picture of the oxidising properties of PM_2.5_ at a central site in Beijing. Positive correlations of OP (normalised per volume of air) of all four assays with overall PM_2.5_ mass were observed, with stronger correlations in winter compared to summer. In contrast, when OP assay values were normalised for particle mass, days with higher PM_2.5_ mass concentrations (μgm^−3^) were found to have lower mass-normalised OP values as measured by AA and DTT. This finding supports that total PM_2.5_ mass concentrations alone may not always be the best indicator for particle toxicity. Univariate analysis of OP values and an extensive range of additional measurements, 107 in total, including PM_2.5_ composition, gas-phase composition and meteorological data, provided detailed insight into the chemical components and atmospheric processes that determine PM_2.5_ OP variability. Multivariate statistical analyses highlighted associations of OP assay responses with varying chemical components in PM_2.5_ for both mass- and volume-normalised data. AA and DTT assays were well predicted by a small set of measurements in multiple linear regression (MLR) models and indicated fossil fuel combustion, vehicle emissions and biogenic secondary organic aerosol (SOA) as influential particle sources in the assay response. Mass MLR models of OP associated with compositional source profiles predicted OP almost as well as volume MLR models, illustrating the influence of mass composition on both particle-level OP and total volume OP. Univariate and multivariate analysis showed that different assays cover different chemical spaces, and through comparison of mass- and volume-normalised data we demonstrate that mass-normalised OP provides a more nuanced picture of compositional drivers and sources of OP compared to volume-normalised analysis. This study constitutes one of the most extensive and comprehensive composition datasets currently available and provides a unique opportunity to explore chemical variations in PM_2.5_ and how they affect both PM_2.5_ OP and the concentrations of particle-bound reactive oxygen species.

## 1 Introduction

Large-scale epidemiological studies have consistently linked the exposure of airborne particulate matter (PM) with a range of adverse human health effects (Hart et al., 2015; Laden et al., 2006; Lepeule et al., 2012). A recent study by the World Health Organisation estimated that 1 in 8 deaths globally in 2014 were linked to air pollution exposure (World Health Organisation, 2016), with urban areas in India and China particularly affected (Lelieveld et al., 2020). However, large uncertainty remains regarding the physical and chemical characteristics of PM that result in adverse health outcomes upon exposure (Bates et al., 2019).

Studies have suggested that oxidative stress promoted by PM components in vivo could be a key mechanism that results in adverse health outcomes (Donaldson and Tran, 2002; Knaapen et al., 2004; Øvrevik et al., 2015). Oxidative stress occurs when excess concentrations of reactive oxygen species (ROS) overwhelm cellular anti-oxidant defences, resulting in an imbalance of the oxidant–antioxidant ratio in favour of the former, which can subsequently lead to inflammation and disease (Knaapen et al., 2004; Li et al., 2003, 2008). The term ROS typically refers to H_2_O_2_, in some cases including organic peroxides, the hydroxyl radical (•OH), superoxide (${\text{O}}_{2}^{•-}$) and organic oxygen-centred radicals. Particle-bound ROS is exogenously delivered into the lung through PM inhalation or can be produced in vivo via redox chemistry initiated by certain particle components, in addition to baseline tissue ROS produced by metabolic processes (Dellinger et al., 2001). The capability of PM to produce ROS with subsequent depletion of anti-oxidants upon inhalation is defined as oxidative potential (OP) (Bates et al., 2019).

OP is a fairly simple measure of PM redox activity but reflects a complex interplay of particle size, composition and chemistries which induce oxidative stress by free-radical generation, which triggers cellular signal transduction and damage. These effects can be both localised (to lung epithelial surfaces and alveoli, reviewed by Tao et al., 2003) and systemic, through immune system activation and cytokine release (Miyata and van Eeden, 2011), translocation of ultrafine particles into the circulatory system (Oberdorster et al., 1992), increased circulating monocytes (Tan et al., 2000), and propagation to other cells and organs (Laing et al., 2010; Meng and Zhang, 2006). Oxidative stress is implicated in the majority of toxicological effects related to air pollution (Ghio et al., 2012; Kelly, 2003; Pope and Dockery, 2006; Risom et al., 2005). A rapid and simple metric to capture the oxidative exposure burden which can be easily implemented for epidemiological studies will enable greater insight into the mechanisms of PM toxicity beyond total PM mass concentrations alone.

There are now a wide range of acellular chemical methods that attempt to quantify particle-bound ROS and the entire OP of PM, as typically acellular assays allow faster measurement and are less labour intensive compared to cell cultures or in vivo methods (Bates et al., 2019). These include, but are not limited to, the dithiothreitol assay (DTT), ascorbic acid assay (AA), 2,7-dichlorofluorescin/hydrogen peroxidase assay (DCFH), electron paramagnetic resonance (EPR) spectroscopy, glutathione assay (GSH) and 9-(1,1,3,3,tetramethylisoindolin-2-yloxyl-5-ethynyl)-10-(phenylethynyl)anthracene (BPEAnit). These acellular assays all have differing sensitivities to specific particle components that may contribute to increased particle-bound ROS concentrations and aerosol OP. For instance, DTT has been shown to be sensitive to soluble metals (Shinyashiki et al., 2009), including copper and manganese (Charrier et al., 2015; Charrier and Anastasio, 2012), as well as a range of organic particle components including water-soluble organic carbon (WSOC, a mixture of hundreds to thousands of compounds), oxidised polycyclic aromatic hydrocarbons (PAHs), e.g. quinones (Chung et al., 2006; McWhinney et al., 2013a), and humic-like substances (HULIS) (Dou et al., 2015; Verma et al., 2015a). AA is particularly sensitive to redox-active transition metals, most notably Fe (Godri et al., 2011) and Cu (Janssen et al., 2014; Pant et al., 2015), and has demonstrated sensitivity to organic carbon (Calas et al., 2018) including secondary organic aerosol (Campbell et al., 2019b). EPR is applied to speciate and quantify radical species bound to aerosol particles (Arangio et al., 2016; Campbell et al., 2019a; Chen et al., 2019; Gehling and Dellinger, 2013), so-called environmentally persistent free radicals (EPFR), or radicals formed upon suspension of particles into aqueous solution (Gehling et al., 2014; Tong et al., 2016, 2017) or in some cases into synthetic lung lining fluid (Tong et al., 2018) consisting of a mixture of AA, glutathione and uric acid. EPR has the advantage of not being influenced by the dark colour of particulate suspensions (detection is via magnetic excitation rather than magnetic absorbance), that it does not require extraction of the PM from the filter and that speciation of the free radical generated can be explored using spin-trap reagents that are selective for specific radicals (Miller et al., 2009). The DCFH assay has been shown to be particularly sensitive to hydrogen peroxide (H_2_O_2_) and organic peroxides (Venkatachari and Hopke, 2008; Wragg et al., 2016), also present in secondary organic aerosol (SOA) particles (Gallimore et al., 2017), and is a particularly useful assay for measuring particle-bound ROS (Wragg et al., 2016). The application of these four commonly used assays simultaneously allows different mechanisms of ROS generation to be assessed: the variability of particle-bound ROS (DCFH), the production of superoxide upon aqueous particle suspension (EPR) and the catalytic generation ofROS via redox-active components (DTT/AA). Therefore, these data provide a broad picture of the variability of both particle-bound ROS and OP, and comparison to a comprehensive compositional dataset provides a unique opportunity to probe the chemical changes in PM that affect the burden of particle-bound ROS and OP.

Despite several studies utilising the aforementioned assays, further exploratory work is required to determine specifically which sources, physical properties and chemical components influence aerosol OP variability. A limited number of investigations have explored the role of chemical composition on aerosol OP, and it is often unclear which specific chemical components are responsible for driving aerosol OP; for example, studies show transition metals such as Cu and Mn dominate DTT activity (Charrier et al., 2015; Charrier and Anastasio, 2012), whereas others highlight the enhanced role of organics, in particular water-soluble organic carbon (WSOC) such as HULIS, and quinones (Cho et al., 2005; Fang et al., 2016). Furthermore, several studies correlate volume-normalised OP measurements with compositional variability, but given the potential co-linearity of many aerosol components with overall mass, mass-normalised intrinsic OP values may provide additional insight into the effect of chemical composition on aerosol OP (Bates et al., 2019; Puthussery et al., 2020). Thus, a comprehensive characterisation of gas- and particle-phase pollution conditions combined with measurements utilising multiple OP assays simultaneously provides a wide range of information on particle-bound ROS and aerosol OP, allowing the identification of the most important components that drive aerosol OP. Ultimately, a greater understanding of the specific aerosol characteristics that influence OP, as well as specific sources that contribute more to aerosol OP, could allow the development of more targeted and efficient air pollution mitigation strategies. Further details of the selection of OP assays, their analytical scope, and biological and epidemiological applicability are described in Sect. S2 of the Supplement.

In this work, PM_2.5_ filter samples collected in winter 2016 and summer 2017 during the APHH campaign (Shi et al., 2019) were analysed using four acellular methods – AA, DCFH, DTT and EPR – providing a broad panel of data on the health-relevant properties of PM_2.5_, including particlebound ROS, redox-active components contributing to aerosol OP and the formation of superoxide radicals upon sample extraction. As the APHH campaign simultaneously captured one of the most extensive and comprehensive atmospheric composition datasets, including a range of PM compositional data, we aimed to establish which individual PM components and meteorological and atmospheric conditions contributed to the increased OP assay response, whether these influences and compositions differed substantially between assays, and if the compositions confirmed previous observations and reflected particular PM sources. We included 107 different measurements, comprising transition metals, AMS measurements, total elemental and organic carbon, and a broad panel of organic species relating to biomass and fossil fuel burning, cooking emissions, vehicular markers, secondary organic aerosol compounds, and gaseous species and general atmospheric conditions. We also sought to investigate the differences between volume-based and mass-based responses, as mass-based analysis facilitates site and temporal comparisons more readily than volume measurements and provides details on intrinsic particle properties that influence OP. In order to highlight underlying trends in such a broad and complex dataset, we also applied multivariate statistical analysis and developed multiple linear regression models to fully characterise the compositional factors driving each assay response.

## 2 Materials and methods

### 2.1 Air Pollution and Human Health in a Chinese Megacity (APHH) campaign

#### 2.1.1 Site description

High-volume 24 h aerosol filter samples were collected at the Institute of Atmospheric Physics (IAP) in Beijing, China (39°58′28″ N, 116°22′15″ E) (Fig. S1). Winter PM was collected during the months of November–December 2016, and summer PM was collected during the months of May – June 2017. *n* = 31 filters for winter 2016 and *n* = 34 filters for summer 2017 were collected. A PM_2.5_ high-volume sampler (RE-6070VFC, TICSH, USA) was used at a flow rate of ~ 1.06 m^3^ min^−1^. PM_2.5_ for subsequent OP analysis was collected onto quartz microfiber filters (Whatman, 20.3 × 25.4 cm) with a collection area of 405 cm^2^.

#### 2.1.2 PM_2.5_ composition, gas-phase composition and meteorological data

Oxidative potential measurements were correlated with a range of additional particle-phase composition, gas-phase composition and meteorological measurements conducted concurrently during the APHH-Beijing campaign (Shi et al., 2019). Briefly, the following composition data were collated: total organic and elemental carbon (OC, EC), soluble inorganic ions (K^+^, Na^+^, Ca^2+^, $\text{N}{\text{H}}_{4}^{+}$, $\text{N}{\text{O}}_{3}^{-}$, $\text{S}{\text{O}}_{4}^{2-}$ and Cl^−^) measured using ion chromatography (IC), low-oxidised organic aerosol and more-oxidised organic aerosol (LOOOA/MOOOA) as well as total organic (ORG) fractions using aerosol mass spectrometry (AMS), biomass burning markers (galactosan, mannosan and levoglucosan), 16 polycyclic aromatic hydrocarbons (PAHs) (see Elzein et al., 2019, 2020), C_24_-C_34_ *n*-alkanes, aerosol cooking markers (palmitic acid, stearic acid, cholesterol), vehicle exhaust markers (17a(H)-22, 29,30-trisnorhopane (C27a) and 17b(H)-21a-norhopane (C30ba)), isoprene SOA markers (2-methylglyceric acid, 2-methylerythritol, 2-methylthreitol, 3-hydroxyglutaric acid), C_5_-alkene triols (*cis*-2-methyl-1,3,4-trihydroxy-1-butene, 3-methyl-2,3,4-trihydroxy-1-butene, *trans*-2-methyl-1,3,4-trihydroxy-1-butene), *α*-pinene SOA tracers (*cis*-pinonic acid, pinic acid, 3-methyl-1,2,3-butanetricarboxylic acid (MBTCA), 2,3-dihydroxy-4-oxopentanoic acid, aged *α*-pinene SOA marker), *β*-caryophyllene SOA tracer (*β*-caryophyllinic acid), and an aromatic volatile organic compound (VOC) SOA tracer (3-isopropylpentanedioic acid) (Liu et al., 2021). The following additional data were obtained from the Centre for Environmental Data Analysis (CEDA) archive: concentrations of inorganic elements Al, Ti, V, Cr, Mn, Fe, Co, Ni, Cu, Zn, Cd, Sb, Ba and Pb in PM_2.5_ using X-ray fluorescence (XRF) (Xu et al., 2020a); gas-phase concentrations of methanol, acetonitrile, acetaldehyde, acrolein, acetone, isoprene, methacrolein, methyl ethyl ketone, benzene, toluene, C_2_-benzenes and C_3_-benzenes measured using proton-transfer-reaction time-of-flight mass spectrometry (PTR-ToF-MS) (Acton et al., 2018); gas-phase concentrations of O_3_, CO, NO, NO_2_, NO_y_ and SO_2_ as well as relative humidity (RH) and air temperature measurements (Shi et al., 2019); photolysis rates for *J*O^1^D and *J*NO_2_ (Whalley et al., 2021); and gas-phase concentrations of hydroxyl radicals (OH), peroxy radicals (HO_2_) and organic peroxy radicals (RO_2_) measured using fluorescence assay gas expansion (FAGE) (Whalley et al., 2021).

### 2.2 Oxidative potential measurements

#### 2.2.1 Reagents

Chemicals and gases were obtained from Sigma-Aldrich unless otherwise indicated and were used without further purification: ascorbic acid (≥99.0 %), Chelex™ 100 sodium form, 0.1 M HCl solution, 0.1 M NaOH solution, dichlorofluorescin-diacetate (DCFH-DA), 1 M potassium phosphate buffer solution, horseradish peroxidase (HRP), methanol (HPLC grade), and *o*-phenylenediamine (≥99.5 %). H_2_O used for the DCFH, HRP and AA solution was obtained from a Milli-Q high-purity water unit (resistivity ≥18.2MΩcm^−1^, Merck Millipore, USA). For DTT analysis, 9,10-phenanthrenequinone (PQN) (≥99 %), 5,5’-dithiobis(2-nitrobenzoic acid) (DTNB) (99 %), DL-dithiothreitol (DTT) (≥98 %), potassium phosphate dibasic (≥98 %, Krebs buffer), potassium phosphate monobasic (≥98 %, Krebs buffer) and methanol (≥99.9 %) were all obtained from Fisher Chemical. Nitrogen (oxygen free) was obtained from BOC (Cambridge, UK).

#### 2.2.2 Acellular oxidative potential assays

Four offline acellular methods for measuring PM_2.5_ oxidative potential and particle-bound ROS were utilised in this work. The DCFH/HRP assay (Fuller et al., 2014) quantifies the fluorescent product 2,7-dichlorofluorescein, an assay that is particularly sensitive to species which are likely particle-bound ROS. The ascorbic acid (AA) assay (Campbell et al., 2019b) quantifies the dominant product of AA oxidation, dehydroascorbic acid (DHA) via condensation with a dye and fluorescence spectroscopy. This is an AA-only assay and does not contain other components normally present in synthetic lung fluid (SLF); filter extracts are performed at pH 7, whereas the AA reaction with the filter extract is performed at pH 2 to improve assay stability and sensitivity (Campbell et al., 2019b). Electron paramagnetic resonance spectroscopy (EPR) (Miller et al., 2009) specifically targets the measurement of superoxide (${\text{O}}_{2}^{•-}$), and the dithiothreitol (DTT) assay (e.g. Cho et al., 2005) quantifies the rate of loss of DTT via absorbance measurements. These acellular methods have been widely applied in the literature to study particle OP and particle-bound ROS (Bates et al., 2019). For detailed descriptions of the assay protocols, see Sect. S3 in the Supplement. Assessing OP and particle-bound ROS in filters with the aforementioned assays is done offline. There is potential to underestimate PM OP and particle-bound ROS using offline filter-based analysis, as short-lived components which contribute to particle-bound ROS and OP may undergo degradation prior to analysis. However, using an offline method allows the opportunity to correlate with a wide range of additional composition measurements, allowing a more explicit characterisation of the chemical components of PM that contribute to observed acellular assay responses.

### 2.3 Statistical analysis

We aimed to analyse the data as thoroughly as possible with respect to characterising the OP measured by each assay and to robustly connect assays to both individual measurements and potential PM sources. As data were collated from several different experimental projects, and as analytical uncertainty values were not available for the majority of the data, the use of positive matrix factorisation (PMF) was not undertaken for source apportionment in this study and will be published subsequently for selected analyses (Xu et al., 2020a). Multiple analytical platforms were used for the acquisition of compositional data; uncertainty estimates for each measurement were not easily estimable; a factor-based chemical mass balance approach was not required specifically; and temperature, relative humidity, actinic flux and other non-mass measurements could also be influential on the OP response and are factors mainly independent of PM sources. On this basis we considered that PMF would not ultimately give useful models in the specific context of OP. However, these issues are managed adequately by principal component analysis (PCA), which is a useful general unsupervised method for examining underlying variance and latent effects in data and handles multicollinearity well, although it is not optimal for chemical mass balance source apportionment (Paatero and Tapper, 1994).

PCA and partial least squares regression (PLSR) models were produced in SIMCA+ 16.0 (Umetrics, Umeå, Sweden). Missing values were not altered prior to model construction, although measurements with more than 56% missing values per season were discarded from models. *R* ^2^ and *Q* ^2^ values were used to assess the goodness of fit of the model and the goodness of prediction of the data through 7-fold cross-validation respectively. Data were unit-variance-scaled and mean-centred to remove effects related to absolute data magnitude. Models were allowed to optimise to the maximum number of latent variables (LVs) at which the cumulative *Q* ^2^ value stabilised, which for most PLSR models was a single LV. PLSR model robustness was assessed through permutation testing, where the classifier (i.e. OP assay response) for all samples was randomly permuted 999 times and the PLSR model constructed for each permutation; the model was considered robust if the real model *R* ^2^ and *Q* ^2^ values outperformed those from all random permutation models. Negative *Q* ^2^ values indicate no predictive power of the data in the model, and LVs with *Q* ^2^ significantly lower than the *R* ^2^ value (arbitrarily defined for this study as *Q* ^2^ at more than 10% below the *R* ^2^) can be considered at least partially overfitted.

Spearman rank correlations (*R*_s_) between OP measurements and PM_2.5_ were calculated using Origin 2020 (Originlab Corporation, USA) and R and were used to assess the relationships between assay responses and individual measurements, with Mann–Whitney U tests used for pairwise testing of the differences in seasonal response for both assays and individual measurements. All other multivariate analyses, multiple linear regression models and selected univariate analyses were produced in R 4.0.2 (R Core Team, Vienna, Austria), implemented in RStudio 1.3.959 (Boston, Massachusetts, USA).

For multiple linear regression models, outlier values were arbitrarily deemed to be those greater than 5 times the standard deviation and replaced with the season median where appropriate for analysis. Measurement subsets manually selected as relevant to source composition were then subjected to a variable selection process, whereby pairwise Spearman correlations for all measurements were calculated, and measurements removed from subsets if they were highly correlated with other measurements but predicted OP more poorly than the other co-correlated measurements to reduce the number of variables contributing identical information in the final models. Multiple linear regression models were then further optimised from this initial subset using the *regsubsets* function in the *leaps* R package to allow for between 4–8 variables, which best predicted the OP response (models could be constructed with fewer or even more measurements, but the aim was to examine a small panel of contributors to potential source compositions). The variable selection process precludes the use of linear regression mode performance indicators such as the Akaike or Bayesian information criteria, as the optimised model basis sets are not identical. The stability of model predictions and features were assessed using bootstrap resampling of data, by randomly splitting one-fifth of the data as a test set and using the remaining samples to construct the model and predict the left-out samples, for 500 random iterations. Stability was also assessed though overall variance in OP predictions, measurement feature coefficients and model residuals plots, and run order/date bias (not differentiable as samples were analysed in date order) was assessed in residuals plots. Although not all data distributions were strictly normal when examined in the univariate kernel density plots, data were not log-transformed for multiple linear regression models, as this creates non-linearity in the model component response, which can complicate interpretation. Model residuals were plotted for manual examination and were all generally normally distributed despite the relatively small number of samples, and biases were related to periods of missing measurements or samples with values below the limit of quantification. Code developed for analysis is publicly available at https://github.com/katewolfer/Beijing (last access: 6 April 2021).

## 3 Results and discussion

Both volume-normalised (OP_v_, per m^3^ air) and particle-mass-normalised (OP_m_, per μg PM_2.5_) values are considered in this work, where the OP value of the specific assay and sample is normalised by the volume of air collected or by the total PM_2.5_ mass on the filter, respectively. OP_v_ is useful when considering exposure or epidemiological outcomes, but OP_m_ is likely a more informative metric when exploring how chemical composition influences PM_2.5_ OP, potentially enabling better OP response, site and composition inter-comparisons (Bates et al., 2019). Henceforth, OP_v_ and OP_m_ will be used when discussing the overall response of all four methods; specific discussion of the acellular methods will be referred to as AA_v_, DTT_v_, DCFH_v_, and EPR_v_ for volume-normalised values and AA_m_, DTT_m_, DCFH_m_, and EPR_m_ for mass-normalised values. For comparison of mass-normalised OP_m_ values, PM_2.5_ composition measurements were also normalised for total PM mass (e.g. ng/μg per μg PM_2.5_)

### 3.1 Seasonal variation of OP_m_ and OP_v_

**Figure 1:**
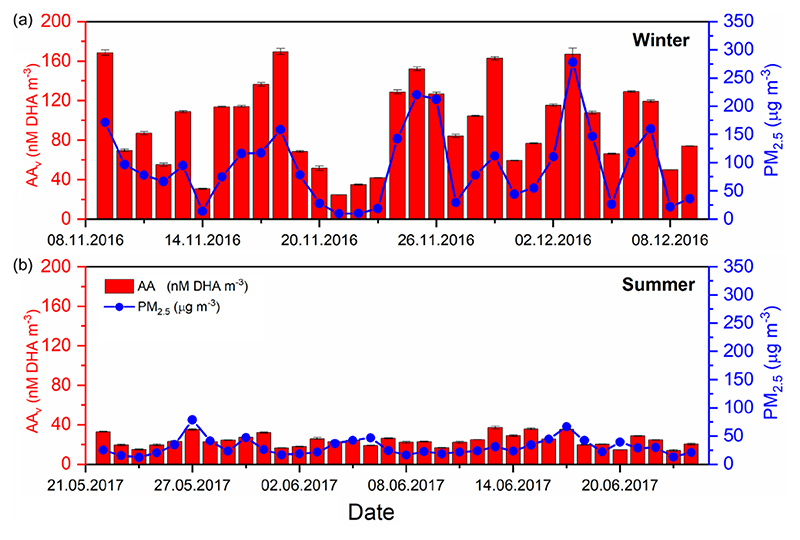
Time-averaged (24 h) volume-normalised AA_v_ (red bars) and PM_2.5_ mass (blue dots), analysed from 24 h high-volume filters, for both winter 2016 (8 November–8 December 2016) and summer 2017 (21 May–24 June 2017) (Shi et al., 2019; Xu et al., 2020a). Substantially higher average PM_2.5_ mass concentrations (μgm^−3^) and AA_v_ were observed in the winter season compared to the summer (see Table S1 for summary). DCFH_v_, DTT_v_ and EPR_v_ 24 h averaged datasets can be found in Figs. S8–S10 respectively.

The 24 h PM_2.5_ mass concentrations in winter 2016 (8 November–9 December 2016) ranged from 8.1–328.7 μgm^−3^, with an average PM_2.5_ mass of 98.7±75 μgm^−3^, whereas in summer 2017 (21 May–24 June 2017) PM_2.5_ concentrations ranged between 13.6–85μgm^−3^ with an average of 36.7±16 μgm^−3^ (Fig. S7) (Shi et al., 2019; Xu et al., 2020a). Average seasonal values for each assay are summarised in Table S1 in the Supplement. An example data set showing 24 h average data, for AA_v_ and PM_2.5_ mass in both the winter and summer campaign, is shown in Fig. 1 (for DCFH_v_, DTT_v_ and EPR_v_; see Sect. S6 “Summary statistics for all measurements”).

For all assays, a higher average was observed in the winter compared to the summer in Beijing (Table S1). The average AA_v_ was 96.7±42.7 nM DHA m^−3^ in the winter, whereas a mean value of 24.1±6.1 nMDHA m^−3^ was observed in the summer. Given the recent introduction of this AA-based assay, which measures the formation of the AA oxidation product DHA rather than measuring the decay of AA via UV absorbance, limited literature values are available for direct comparison (Campbell et al., 2019b). Average DCFH_v_ in the winter was 0.71±0.52 nmol H_2_O_2_ m^−3^ compared to 0.17±0.11 nmol H_2_O_2_m^−3^ in the summer, which is within the range of DCFH_v_ values observed in previous studies in Taiwan, the United States and Singapore (OP_DCFH_ 0.02–5.7 nmol H_2_O_2_m^−3^) (Hasson and Paulson, 2003; Hewitt and Kok, 1991; Hung and Wang, 2001; See et al., 2007; Venkatachari et al., 2005). Mean observed values for DTT_v_ in the winter and summer were 2.9±0.11 nmol min^−1^ m^−3^ and 0.9±0.40 nmol min^−1^ m^−3^, respectively. The mean values of DTT_v_ observed in this study are greater than those measured in similar studies in Beijing (Liu et al., 2014) (0.11–0.49, mean = 0.19 nmol min^−1^ m^−3^) with similar mass concentrations of PM_2.5_ (mean = 140 μgm^−3^), although they are within the range of DTT_v_ values observed in a number of previous studies in several locations, including Europe (Jedynska et al., 2017; Yang et al., 2015), the United States (Fang et al., 2015; Verma et al., 2014) and northern China (Liu et al., 2018) (0.1–14.7 nmol min^−1^ m^−3^). The mean EPR_v_ values, relating to the specific detection of ${\text{O}}_{2}^{•-}$, were 2.4 × 10^6^±1.6×10^6^ and 5.8×10^5^±4.1×10^6^ counts m^−3^ in the winter and summer campaign, respectively.

**Figure 2:**
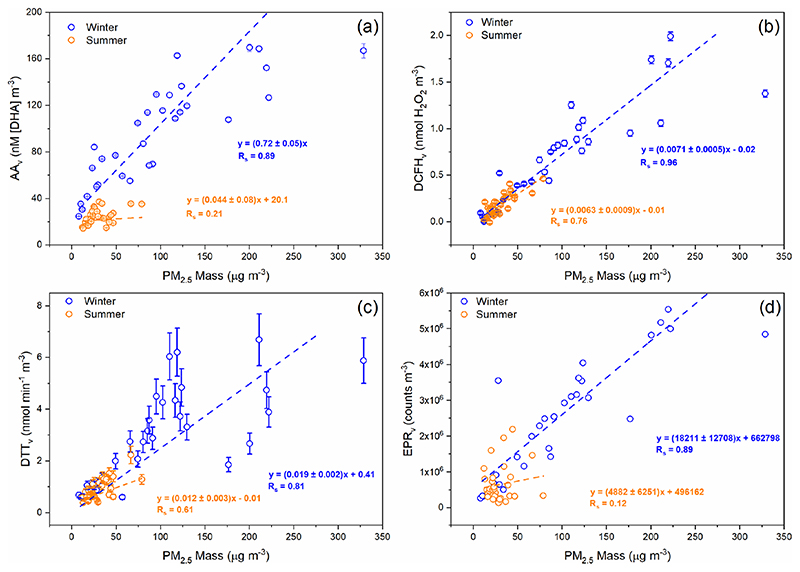
Comparison of PM_2.5_ OP_v_ during winter 2016 (blue) and summer 2017 (orange) vs. PM_2.5_ mass (μgm^−3^). **(a)** AA_v_, **(b)** DCFH_v_, **(c)** DTT_v_ and **(d)** EPR_v_. Each data point represents a 24 h average for OP measurements and PM_2.5_ mass. Corresponding *R*_s_ and linear fit equations are included. For AA_v_, DCFH_v_ and DTT_v_, error bars represent the standard deviation observed over three repeat measurements for each filter sample, and in some cases the error is smaller than the data point. Uncertainty values are unavailable for EPR_v_ measurements.

Spearman rank correlation coefficients (*R*_s_) of aerosol OP_v_ with PM_2.5_ vary between the winter and summer season, and also between OP assays, as illustrated in Fig. 2. All four assays, when normalised per volume (OP_v_), show a stronger correlation with PM_2.5_ mass concentration in the winter compared to the summer, consistent with results observed in Chamonix, France, by Calas et al. (2018). For example, DCFH_v_ correlates well with 24 h average total PM_2.5_ mass concentration (μgm^−3^) in both winter (*R*_s_ = 0.96) and summer (*R*_s_ = 0.76) (Fig. 2b), whereas AA_v_ correlates well in the winter (*R*_s_ = 0.89) and poorly in summer (*R*_s_ = 0.21). Similar correlations of DCFH_v_ with PM_2.5_ mass concentrations in both winter and summer suggest that species influencing DCFH_v_ variability (e.g. H_2_O_2_ and organic peroxides, likely particle-bound ROS) present in the particles are relatively consistent between both seasons. Similar to AA_v_, differences between the seasons are also observed for DTT_v_ and EPR_v_, where correlations of aerosol OP_v_ vs. PM_2.5_ are stronger in winter compared to summer (Fig. 2c and d), also generally consistent with previous studies, although in contrast to Calas et al. (2018), who observed no difference in EPR_v_ between seasons in Chamonix, in that study the spin-trap 5,5-dimethyl-1-pyrroline-N-oxide (DMPO) was used to study hydroxyl radicals, whereas in this study we focus on the formation of superoxide upon particle suspension in aqueous solution. The differences in the correlation shown in Fig. 2 suggests that the four assays are sensitive to different PM components and that in winter and summer different PM sources or components are important for the assay’s responses (Calas et al., 2018; Saffari et al., 2013; Verma et al., 2014). Figure 2 demonstrates that PM_2.5_ mass could be a reasonable predictor of total OP_v_ in winter but the poorer correlations between all OP_v_ assays and PM_2.5_ in the summer indicate that a more detailed understanding is necessary to elucidate and ultimately predict aerosol OP. However, the variability in the strength of correlation between OP_v_ and PM_2.5_ mass as well as the seasonal difference indicates that compositional differences in PM_2.5_ or additional atmospheric processes influence PM_2.5_ OP.

**Figure 3:**
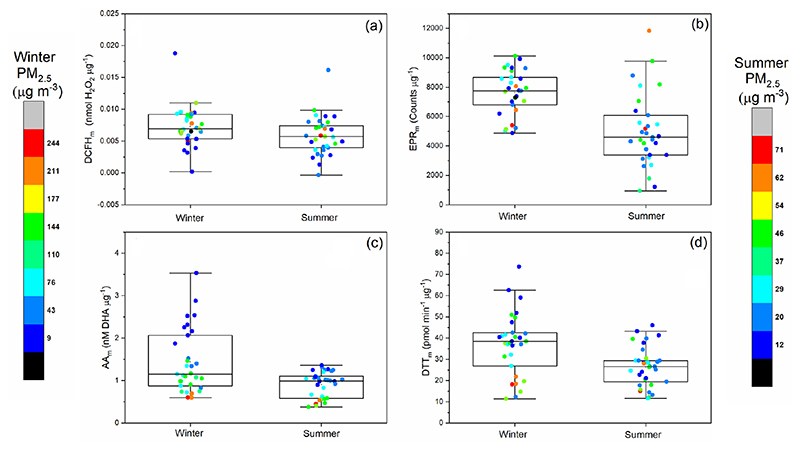
Summer and winter 24 h averaged mass-normalised OP_m_ **(a)** DCFH_m_ (nmol H_2_O_2_ μg^−1^), **(b)** EPR_m_ (counts μg^−1^), **(c)** AA_m_ (μMDHAμg^−1^) and **(d)** DTT_m_ (pmol min^−1^ μg^−1^). Box plots indicate the median, 25% and 75% percentiles, and the data range. Data points are colour coded with respect to the 24 h average PM_2.5_ mass (μgm^−3^), with a separate colour scale for winter and summer PM_2.5_ masses given the difference in total PM_2.5_ masses observed between the seasons.

To gain further insights into the potential particle-level compositional differences underlying assay OP response, the OP data for the four assays was normalised to the PM_2.5_ mass in each sample. As shown in Fig. 3, mass-normalised OP_m_ values vary up to a factor of 10 within a single season. AA_m_, DCFH_m_, DTT_m_ and EPR_m_ for both winter and summer are also displayed in Fig. 3, with colour bars indicating the 24 h average total PM_2.5_ mass (μgm^−3^) for the corresponding OP_m_ measurement. The average OP_m_ response observed in this study shows a similar trend to OP_v_ (Table S2), where higher OP_m_ values are observed for winter compared to summer (Fig. 3), as observed previously (Liu et al., 2018; Saffari et al., 2014). This demonstrates that there are specific properties of PM_2.5_ in the winter that result in overall higher intrinsic OP_m_ compared to the summer.

For AA_m_, an inverse relationship between total PM_2.5_ mass concentration and AA_m_ is observed in both seasons, where days with high PM_2.5_ mass loadings have correspondingly low AA_m_ values in both the winter and summer, with almost a factor of 6 difference between the AA_m_ on the highest PM_2.5_ mass day (PM_2.5_ = 328 μgm^−3^, AA_m_ = 0.6nM [DHA] μg^−1^) and lowest PM_2.5_ mass day observed during the winter campaign (PM_2.5_ = 8 μgm^−3^, AA_m_ = 3.53 nM [DHA] μg^−1^). A similar trend is observed for DTT_m_, where in general days with higher overall PM_2.5_ mass concentrations have correspondingly low DTT_m_ values, which has also been observed previously (J. Wang et al., 2020). The DTT_m_ response is also not correlated with Cu and Mn concentrations, despite the non-linear but monotonic relationship between these components being demonstrated in other studies (Charrier et al., 2016). These results indicate that on high-pollution days a large fraction of the PM mass might be OP-inactive, resulting in low intrinsic OP_m_ values. In general, smaller particles have been observed to have higher DTT_m_ values compared to larger particles (Bates et al., 2019; Janssen et al., 2014), an effect which may also play a role here. Another possibility is that on higher PM_2.5_ mass days, selected chemical species interact with or deactivate redox-active components present in PM_2.5_ (e.g. interaction of organics with metals (Tapparo et al., 2020), therefore reducing the observed OP_m_ signal. It is also possible that components present in PM_2.5_ on higher PM_2.5_ mass concentration days interfere with the assay response. It is currently unclear which chemical components are responsible for the observed inverse relationship between PM_2.5_ mass with AA_m_ and DTT_m_. However, statistically significant inverse correlations are observed between AA_m_ and DTT_m_ in both the winter and summer with the chemically undetermined “unknown” fraction of PM_2.5_ for DTT_m_ (*R*_s_ = −0.81) and AA_m_ (*R*_s_ = −0.75), implying that PM_2.5_ chemical components unaccounted for in this study are likely responsible for the lower intrinsic AA_m_ and DTT_m_ values on high PM_2.5_ mass days (see Sect. 3.2 “Univariate analysis of PM OP and additional measurements”, Figs. S11 and S12).

In contrast, higher DCFH_m_ responses are observed on days with greater PM_2.5_ mass concentrations in both winter and summer. Increased DCFH_m_ responses on more polluted days could indicate that the mass fraction of particle-bound ROS (e.g. organic peroxides from SOA) increases with increasing PM_2.5_ mass concentration or that the capacity of PM components to produce H_2_O_2_ upon extraction, as measured by DCFH, is enhanced. Despite the significant seasonal difference in EPR_m_, no obvious relationship between EPR_m_ and PM_2.5_ mass was observed in our study. There is potential to underestimate PM OP and particle-bound ROS using offline filter-based analysis, as short-lived components which contribute to particle-bound ROS and OP may undergo degradation prior to analysis. However, using an offline-based method allows the opportunity to correlate with a wide range of additional composition measurements, allowing a more explicit characterisation of the chemical components of PM that contribute to observed acellular assay responses.

Spearman rank correlations (*R*_s_) between the four assays, for mass-normalised OP_m_ and volume-normalised OP_v_, are presented in Table 1. In terms of OP_v_, all four assays show significantly strong correlations with each other in the winter season (*R*_s_ 0.72-0.89), but weaker correlations are observed between assays in the summer (*R*_s_ 0.01-0.58), a seasonal difference observed previously by Calas et al. (2018). In contrast, the only statistically significant correlation observed for OP_m_ is between AA_m_ and DTT_m_ in the winter season only (*R*_s_ = 0.58).

Seasonality of both OP_v_ and OP_m_ observed in the assays could be driven by changes in PM sources influencing overall OP, or a number of physical and chemical factors directly affecting particle composition. For instance, lower ambient temperatures in the winter may increase the partitioning of semi-volatile organic compounds, such as small quinones (e.g. anthracenequinone and 2,3-dimethylanthraquionone, Delgado-Saborit et al., 2013) and nitro-PAHs, which have been shown to influence DTT activity (Ntziachristos et al., 2007; Verma et al., 2011), observations which are supported by lab-based studies showing decreasing aerosol OP at higher temperatures (Biswas et al., 2009; Verma et al., 2011). Changing boundary layer height between the seasons may also contribute to higher concentrations of species which correlate with PM_2.5_ mass responsible for increasing aerosol OP during the winter, compared to summer, especially affecting OP_v_ seasonality (H. Wang et al., 2020). Furthermore, air mass history may be an important contributor to the observed seasonality of OP. For instance, it was observed that winter days with high PM_2.5_ mass concentrations typically originate from regional sources south of Beijing, which is widely industrialised, whereas high mass days in the summer typically have more varied air mass histories (Panagi et al., 2020; Steimer et al., 2020). There are likely varying contributions between different sources in different seasons, e.g. more photochemistry in the summer driving oxidation and biogenic sources, and more contributions from residential heating combustion in the winter (Xu et al., 2020a). In order to gain further insight into what causes the observed variability of OP, relationships between particle chemical composition and aerosol OP will be explored in detail below.

### 3.2 Univariate analysis of PM OP_m_ and additional measurements

Spearman rank correlations between OP_m_ of the four assays and 107 additional measurements conducted during the APHH campaign (see Sect. 2.1.2 “PM_2.5_ composition, gasphase composition and meteorological data”) were calculated for both the winter (*n* = 31) and summer (*n* = 34). We focus on OP_m_ in the forthcoming discussion; as mentioned previously, we consider it a particularly informative metric when determining the role of chemical composition on OP (Bates et al., 2019; Puthussery et al., 2020). All univariate statistical summaries are presented in Sect. S8.

**Figure 4:**
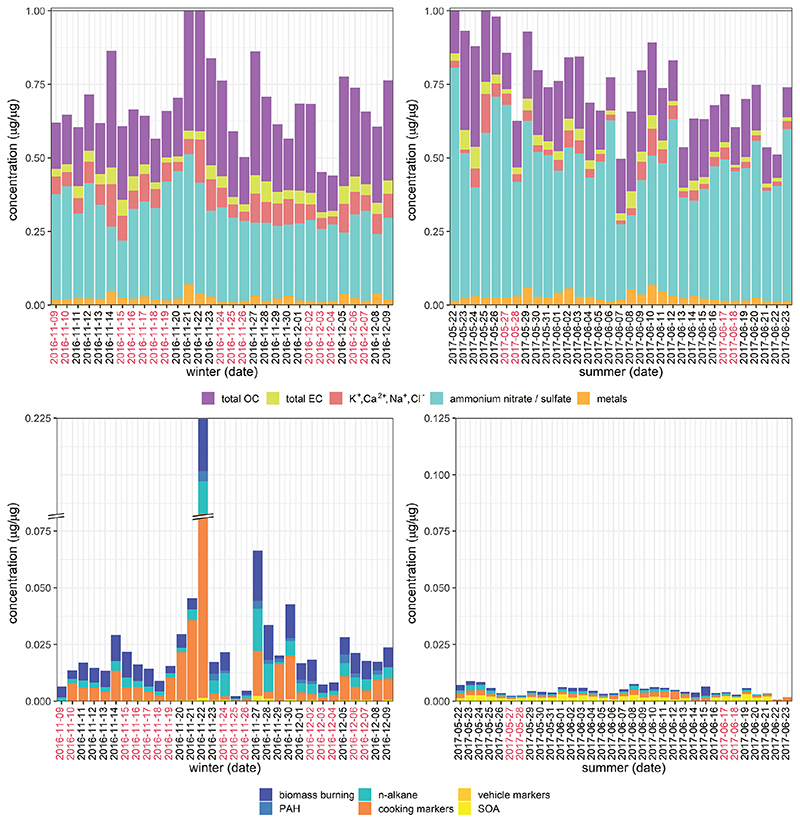
Stacked bar plots of total concentrations for mass-normalised data. OC: organic carbon; EC: elemental carbon; PAH: polycyclic aromatic hydrocarbon; SOA: secondary organic aerosol. “Metals” is the summed concentrations of Al, Ti, V, Cr, Mn, Fe, Co, Ni, Cu, Zn, Cd, Sb, Ba, Pb; “biomass burning” is the summed concentrations of palmitic acid, stearic acid and cholesterol; “PAH” is the summed concentrations of naphthalene, acenaphthylene, acenaphthene, fluorene, phenanthrene, fluoranthene, pyrene, benzo(a)anthracene, chrysene, benzo(b)fluoranthene, benzo(k)fluoranthene, benzo(a)pyrene, indeno(1,2,3-cd)pyrene, dibenzo(a,h)anthracene and benzo(ghi)perylene; “*n*-alkane” is the summed concentrations of C24, C25, C26, C27, C28, C29, C30, C31, C32, C33 and C34; “cooking markers” is the summed concentrations of palmitic acid, stearic acid and cholesterol; “vehicle markers” is the summed concentrations of 17a(H)-22,29,30-trisnorhopane (C27a) and 17b(H),21a(H)-norhopane (C30ba); “SOA” is the summed concentrations of 2-methylthreitol, 2-methylerythritol, 2-methylglyceric acid, *cis*-2-methyl-1,3,4-trihydroxy-1-butene, 3-methyl-2,3,4-trihydroxy-1-butene, trans-2-methyl-1,3,4-trihydroxy-1-butene, C5-alkene triols, 2-methyltetrols, 3-hydroxyglutaric acid, *cis*-pinonic acid, acid, MBTCA, *β*-caryophyllinic acid, glutaric acid derivative, 3-acetylpentanedioic acid, 3-acetylhexanedioic acid, 3-isopropylpentanedioic acid and 2,3-dihydroxy-4-oxopentanoic acid. Dates marked in red indicate partial or total day haze events as described in Shi et al. (2019). Measurement uncertainty values were unavailable for most data types, and for selected dates in the upper plots, the sum of the total mass measurements is slightly more than 1 (i.e. more than 1 μg per μg); for these dates, the data have been proportionately scaled. It should be noted that the OC measurement in the upper plots incorporates the variety of organic carbon species represented in the lower plots.

The majority of additional particle-phase composition, gas-phase composition and meteorological measurements differed significantly by season. Exceptions included Al, V, Zn, Pb, Ca^2+^, Na^+^, $\text{N}{\text{H}}_{4}^{+}$, acetaldehyde, acetonitrile, methanol, methyl ethyl ketone, methyl vinyl ketone/methacrolein, trans-2-methyl-1,3,4-trihydroxy-1-butene, *β*-caryophyllinic acid, 3-hydroxyglutaric acid, C5-alkene triols, cholesterol, LOOOA and MOOOA. Stacked bar plots illustrating the total daily concentrations for both mass-normalised and volume-normalised data are shown in Figs. 4 and S13. Total concentrations of individual PM components (excluding all composite measures) account for approximately 0.3–0.8 μg μg^−1^, i.e. 30%–80% of the total PM mass (data not shown). Interestingly there were no marked or characteristic changes in mass composition associated with haze days; however, haze events were generally correlated with increased biomass burning marker concentration and total organic carbon in winter for the mass-normalised data (also observed during recent later winter haze events in Beijing (Li et al., 2019), as well as small inorganic ion concentrations in both seasons in the volume-normalised data (Fig.S13).

IC measurements (K^+^, Na^+^, Ca^2+^, $\text{N}{\text{H}}_{4}^{+}$, $\text{N}{\text{O}}_{3}^{-}$ and $\text{S}{\text{O}}_{4}^{2-}$) account for the greatest proportion of total particle mass in both seasons, all of which are major components of secondary inorganic PM mass ($\text{N}{\text{H}}_{4}^{+}$, $\text{N}{\text{O}}_{3}^{-}$, $\text{S}{\text{O}}_{4}^{2-}$), mineral dust (Ca^2+^, K^+^) and marine aerosols (Na^+^, Cl^−^). These species were present at higher daily concentrations in summer than in winter. Summer compositions for each category were generally consistent for the whole sampling period, with a larger total proportion of SOA markers, whereas winter compositions were more variable, with greater contributions from elemental carbon, PAHs, *n*-alkanes and cooking-related compounds than for summer samples. Although PAHs are not redox-active (Charrier and Anastasio, 2012), they are precursors to redox-active oxy-PAHs (quinones) and nitro-PAHs (Atkinson and Arey, 2007) and have well-established intrinsic cellular toxicity (reviewed in Moorthy et al., 2015), mediated by their conversion to hydroxy-PAHs, which exert mutagenic and teratogenic effects and also induce transcriptional modifications and oxidative stress. EC and *n*-alkanes are also non-redox-active, and the exact mechanisms of their toxicities are unclear (Levy et al., 2012); however, SOA derived from the interaction of *n*-alkanes with NO_*x*_ with photooxidation (Lim and Ziemann, 2005; Presto et al., 2010) is likely both to contribute to the redox activity of samples (Tuet et al., 2017) and to have more toxic properties than its precursors (Xu et al., 2020b). The sample from 22 November 2016 has a particularly high concentration of cooking markers (palmitic acid, stearic acid and cholesterol). This could reflect the fact that the traditional Chinese winter solar term Xiao Xue (小雪, “light snow”) begins on this date (Li, 2006), a period associated with the preparation of warm foods as the ambient temperatures in northern China drop; a similar elevation of palmitic acid and stearic acid has been observed around the same week in a more recent study in Shanghai (Q. Wang et al., 2020).

**Figure 5:**
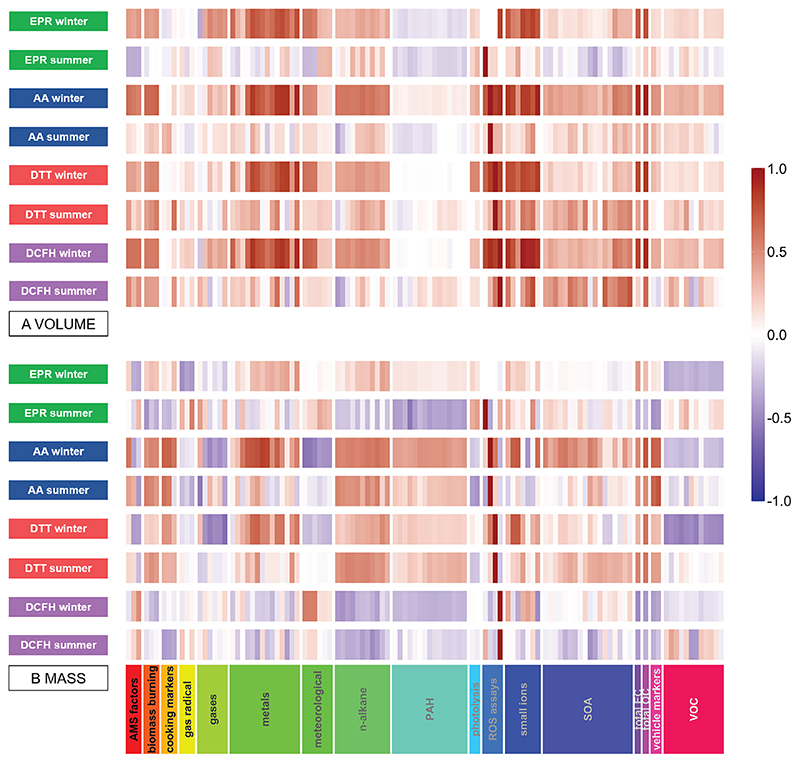
Heat maps demonstrating the correlation of OP, expressed as volume-normalised OP_v_ **(a)** and mass-normalised OP_m_ **(b)** vs. a range of additional measurements conducted during the APHH campaign. Red indicates positive correlation; blue indicates inverse correlation. For OP_m_, particle-phase components are also mass-normalised (μg per μg PM_2.5_), and for OP_v_ the components are volume-normalised (μg or ng per m^3^).

*R*_s_ values calculated for OP_v_ and OP_m_ with individual compositional measurements have strikingly different univariate correlations, as illustrated in correlation heat maps (Fig. 5). Cumulative scores, referring to the number of *R*_s_ correlations ≥ 0.5 for OP_m_ and OP_v_ (Table S3), demonstrate that for all assays, considerably more significant correlations are observed for OP_v_ in the winter compared to OP_m_. For both OP_v_ and OP_m_, all assays show more statistically significant correlations in winter compared to summer, particularly for the AA response (AA_m_, *n* = 54 winter, *n* = 15 summer, AA_v_, *n* = 67 winter, *n* = 4 summer).

Volume-based correlation analysis (Fig. 5a) indicates that a very large number of the 107 atmospheric components measured in this study correlate statistically significantly with all four assays. The large number of correlations in the volume-normalised data indicate strong collinearity between concentrations of chemical components in PM_2.5_ and overall PM_2.5_ mass concentrations likely due to meteorological processes, complicating analysis of the sources and processes contributing to OP variability in particles. However, the mass-based analysis (Fig. 5b) reveals that the mass fractions of chemical components and sources to which the four assays are sensitive to differ significantly (further illustrated by the weaker inter-assay correlations shown in Table 1), which demonstrates that mass-based analysis of OP data is also important to elucidate atmospheric processes and particle sources responsible for the different OP metrics.

A range of transition metals were all positively correlated with AA_m_ and DTT_m_, including V, Cr, Mn, Fe, Co, Ni, Zn, Cd and Pb (all *R*_s_ ≥ 0.5, *p* < 0.05). This reinforces the importance of their contribution to urban PM_2.5_ and potential to substantially influence PM_2.5_ OP, particularly Fe, Cr, V and Co, which are commonly major components of vehicle emissions and which can undergo redox-cycling reactions producing ROS (Charrier et al., 2014; Shen and Anastasio, 2012; Valko et al., 2005) contributing to higher AA_m_ and DTT_m_ in the winter compared to the summer. Stronger correlations between Fe and AA_m_ are observed in the winter (*R*_s_ 0.73) compared to summer (*R*_s_ 0.48) despite Fe concentrations (μg μg^−1^) being lower in winter samples than summer samples, again highlighting the enhanced role of redox-active transition metals in winter. It is not established whether this seasonal difference is related to the chemical availability (i.e. redox state, solubility, speciation) of Fe, to the variability of emission sources of Fe between the seasons or to some other important unknown additional contribution of Fe to AA_m_ in the summer; complexation of Fe in PM may differ between seasons, and the ligands directly influence the redox state and thus the bioavailability of the metal (Ghio et al., 1999). Ultimately, the direct correlation of transition metals measured only by inductively coupled plasma mass spectrometry (ICP-MS) with OP does not adequately reflect the nuances in redox behaviour of these species when they are complexed with organic ligands (Calas et al., 2017), as well as their range of oxidation states; this represents further gaps in the standard chemical (and particularly the transition metal and TM complex) characterisation of PM. The epidemiological effects related to bioavailability of the metal when complexed (Costa and Dreher, 1997) in humans are also still not fully explored, although it is clear from multiple atmospheric and clinical studies that complexation affects transition metal uptake both in the atmosphere and in the body. Interestingly, a mild inverse correlation of Fe with DCFH_m_ is observed (Table S8), which may be linked to the destruction of particlebound organic peroxides by Fe via Fenton-type chemistry (Charrier et al., 2014), a process which the DCFH assay is specifically sensitive to (Gallimore et al., 2017; Wragg et al., 2016) and which has been observed in other recent studies (Paulson et al., 2019). No significant positive correlation between any metals measured in this study and DCFH_m_ and EPR_m_ was observed. Few EPR studies have looked specifically at superoxide formation, as is the case here, but those conducted so far show that EPR specifically detecting ${\text{O}}_{2}^{•-}$ is less sensitive to transition metal chemistry compared to traditional EPR methods focussing on OH formation.

In the summer, from the measured transition metals, only Fe correlated significantly positively (Spearman p value < 0.05) with DTT_m_ and AA_m_ response (*R*_s_ = 0.48, 0.51 respectively), whereas in the winter, DTT_m_ and AA_m_ correlated with a number of transition metals, including V, Cr, Mn, Fe, Co, Ni, Zn and Cd. Of particular note, AA_m_ is mildly correlated with Cu in winter samples (*R*_s_ 0.48), whereas no correlation is observed between DTT_m_ and Cu in either winter or summer, in agreement with a recent online DTT study also (Puthussery et al., 2020). In contrast, previous reports from other locations have implicated Cu as a dominant contributor to DTT oxidation, considering volume-normalised and mass-normalised data (Calas et al., 2018; Charrier et al., 2015). Interestingly, in contrast with OP_m_, strong correlations (*R*_s_ > 0.6) are observed in this study between AA_v_, EPR_v_, DCFH_v_, and DTT_v_ and Cu in the winter, but poorer correlations are observed in the summer for all assays (*R*_s_ < 0.39). Higher average Cu concentrations in winter compared to summer (winter = 17.7 ng m^−3^, summer = 4.9 ng m^−3^) may explain the higher *R*_s_ observed for Cu vs. OP_v_ in winter compared to summer, whereas mass-normalised concentrations of Cu are more similar between the seasons. Poor correlation of Cu concentrations with AA_m_ and DTT_m_ response in winter may hint at more insoluble Cu complex formation observed at this site in Beijing, as predominantly water-soluble Cu participates in redox reactions; therefore the sensitivity of AA and DTT towards Cu probably depends on the soluble fraction of Cu (Bates et al., 2019; Charrier and Anastasio, 2012; Fang et al., 2016). Furthermore, the presence of organic chelating ligands in PM may reduce the redox activity of Cu and Fe (Charrier et al., 2014; Charrier and Anastasio, 2011; Shen and Anastasio, 2012).

Correlations between AA_m_ and DTT_m_ with total OC are observed in both summer and winter (Tables S6 and S7), and with total EC in the winter season, whereas DCFH_m_ is negatively correlated with total OC (Table S8). In contrast, DCFH_m_ is positively correlated with MOOOA and LOOOA, whereas DTT_m_ and AA_m_ show no correlation and even exhibit slight negative correlations with MOOOA and LOOOA in both summer and winter. This potentially indicates that the MOOOA and LOOOA AMS fractions, typically associated with water-soluble organic carbon content (Verma et al., 2015b), may contain higher concentrations of particle-bound ROS (i.e. organic peroxides) as measured by DCFH_m_, but on a per-mass basis these species may contribute less significantly to AA_m_ and DTT_m_ compared to redox-active transition metals and other organic components. Total OC and EC correlations with AA_m_ and DTT_m_ may relate to concentrations of redox-active organic components such as oxidised PAHs and quinones, which may not be represented by MOOOA and LOOOA factors and which have been shown to significantly contribute to DTT_m_ (Chung et al., 2006; McWhinney et al., 2013b).

Significant correlations are also observed between AA_m_ and a range of *n*-alkanes and hopanes (17a(H)-22, 29, 30-trisnorhopane (C27a) and 17b(H)-21a-norhopane (C30ba), Table S6), markers of primary organic aerosol emitted from vehicles (Schauer et al., 1999; Subramanian et al., 2006). Although these species are not redox-active, they are co-emitted with redox-active transition metals such as Fe, V and Cu from vehicle activity, either directly (Bates et al., 2019) or via dust resuspension, and other organics contributing to SOA (Platt et al., 2014) and highlight the potential importance of vehicular emissions on AA_m_. Vehicular emissions and dust resuspension have been previously shown to be the dominant sources of Cu and Fe in Beijing (Gao et al., 2014). EPR_m_, DTT_m_ and DCFH_m_ responses do not show any significant correlations with these organic traffic markers.

Notably, AA_m_ correlates well with *cis*-pinonic acid, pinic acid and 3-methyl-2,3,4-butanetricarboxylic acid (MBTCA) in both seasons, all of which are biogenic SOA markers and products of *α*-pinene oxidation, with MBTCA a marker for OH-initiated ageing of first-generation *α*-pinene oxidation products (Müller et al., 2012). AA sensitivity towards *α*-pinene SOA has been demonstrated previously (Campbell et al., 2019b; Tong et al., 2016). Although these three carboxylic acids are also not redox-active, they may correlate with the formation of particle-bound ROS such as peroxides or peroxy acids in SOA (Steimer et al., 2018), or with species that decompose and liberate ROS upon extraction (e.g. Tong et al., 2017); these processes are highly likely to contribute to AA_m_, highlighting the assay’s potential sensitivity to redox-active particle-phase components and particle-bound ROS. Generally, DTT_m_ has been previously shown to be relatively insensitive to SOA as observed here (Bates et al., 2015; Verma et al., 2015b), and both DTT_m_ and DCFH_m_ correlate poorly with the SOA markers analysed in the present study (Tables S7 and S8).

Compared to the three other assays, few significant correlations are observed between EPR_m_ and additional measurements, despite the much better correlations with the EPR_v_ data, particularly for the summer samples. However, seasonality in the EPR_m_ response is still observed, with substantial variability in the mass-normalised EPR_m_ response (≈ factor of 10 in the summer, factor of 2 in the winter, Fig. 3). Therefore, we observe differences in aerosol composition influencing EPR_m_, but with the current comprehensive measurements we are unable to determine the specific PM_2.5_ components responsible for the observed EPR_m_. As an example, recent studies have found associations between peroxide-containing highly oxygenated molecules (HOMs) in PM_2.5_ and superoxide formation in water (Chowdhury et al., 2019; Tong et al., 2019; Wei et al., 2021); thus HOMs, which were not measured in this study, could have contributed towards the observed EPR_m_ variability.

The univariate analysis presented here clearly shows that OP_m_ enables a more nuanced identification of aerosol components influencing the oxidising properties of PM_2.5_ as compared to OP_v_. Many more correlations are observed when considering volume-normalised OP_v_, likely related to collinearity of species with overall PM_2.5_ mass concentration due to meteorological effects. Metal and organic tracers of traffic emissions (exhaust and non-exhaust) such as Fe, Cu, and hopanes and SOA markers show especially strong correlations with AA_m_, whereas the other three OP_m_ metrics (DTT_m_, DCFH_m_ and EPR_m_) provide a less clear picture.

### 3.3 Multivariate modelling of OP from measured components

To assess potential latent influences from the individual components on assay response and hence on OP, a systematic multivariate analysis was undertaken. Initially, principal component analysis was applied to the whole set of independent measurements excluding the OP assay responses (i.e. the values to be predicted by the models) to investigate which contributed most to the variation in the data, whether there were relationships between measurements which characterised OP and if the OP_m_ response could be predicted from the individual component measurements.

**Figure 6:**
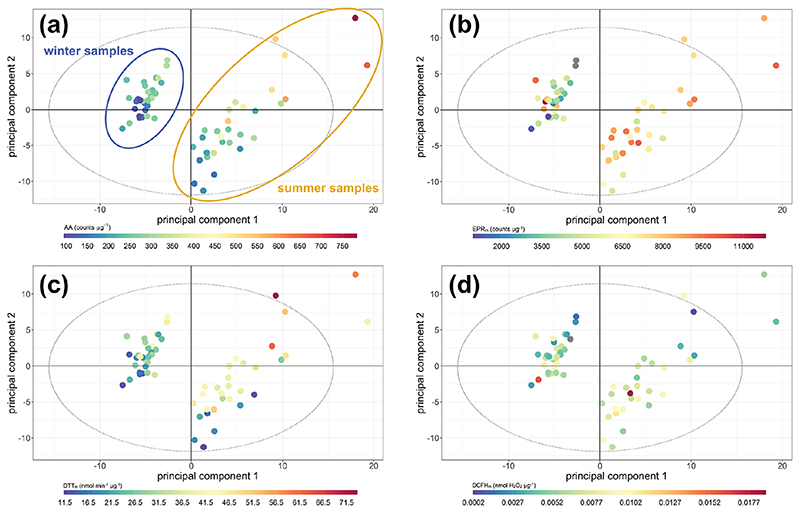
Principal component analysis score plot of all data. **(a)** Coloured by AA_m_ response; **(b)** coloured by EPR_m_ response; **(c)** coloured by DTT_m_ response; **(d)** coloured by DCFH_m_ response. Both principal component 1 and principal component 2 demonstrate variance associated with AA and DTT response, and there is greater variation associated with the winter response than the summer response (highlighted in panel **a**). PC 1 *R* ^2^ *X* 35.90 %, *Q* ^2^ 29.28 %; PC 2 *R* ^2^ *X* 19.34 %, *Q* ^2^ 23.73 %; the model included six principal components, with a cumulative *R* ^2^ *X* of 68.2 % and *Q* ^2^ of 50.5 %.

**Figure 7:**
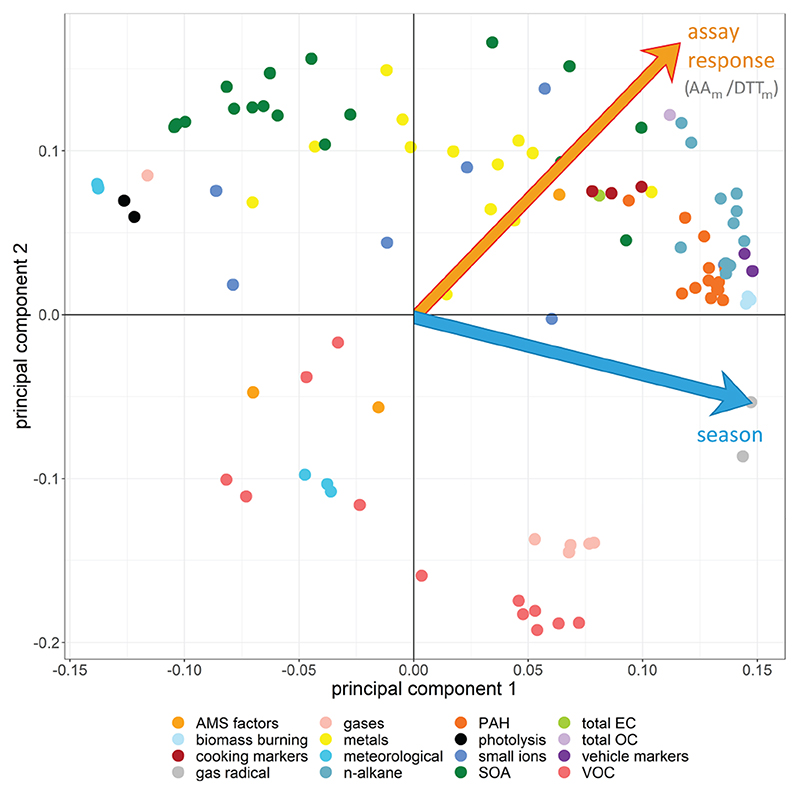
Principal component analysis loadings plot for all data points. Points are coloured by measurement category; fully labelled loadings are provided in Fig. S14. The plot is annotated with the same orientation as the score plot to indicate the direction of visualised trends for selected assays and for season from the latent variable origin as shown in Fig. 6. In PC 1, the winter classification is driven by increased gas radicals, n-alkanes, PAH, vehicle markers, biomass burning markers, total OC and selected metals and SOA markers; the summer classification is driven by increased temperature and photolysis, ozone (the single gas species in this section of the plot), selected SOA markers and metals, and selected VOCs. In PC 2, high AA_m_ and DTT_m_ response is associated with increased SOA, transition metals, cooking markers, n-alkanes and PAH concentrations in samples; low AA_m_ and DTT_m_ response associated with low VOCs, gases and selected meteorological parameters (relative humidity).

In the PCA model, the seasonal variation within the samples was clearly apparent (Fig. 6). The first four principal components (PCs) accounted for 68.2% of the observed variation in the dataset (*R* ^2^ or goodness of fit), of which 50.5% was stable through 7-fold cross-validation (*Q* ^2^, or model variation accounted for through cross-validation), indicating about half of the variation in the model was robust with respect to sample score prediction. The loadings plot (Fig. 7) indicated that seasonality in the first principal component was related to increased PAHs (Feng et al., 2019), *n*-alkanes (He et al., 2006) and biomass burning markers (He et al., 2006) in winter, as well as increased ozone (Zhao et al., 2018), ambient temperature and selected SOA markers (including 2-methylerythritol (Liang et al., 2012), and 2-methylglyceric acid (Ding et al., 2016; Shen et al., 2018)) in summer, findings which are consistent with existing volume-based studies. When scores were coloured by OP, the AA_m_ (Fig. 6a), DTT_m_ (Fig. 6c) and DCFH_m_ (Fig. 6d) assay responses could be observed in the second and sometimes also the first principal components (although the EPR_m_ response demonstrated no specific trend, Fig. 6b). When loadings plots were examined by general measurement category (Fig. 7), it was observed that some categories of measurements cluster together (e.g. PAH, *n*-alkanes, NO_*x*_, temperature, relative humidity), but this appeared to be related to strong correlation of these species with the OP_m_ measurement and known compound behaviour rather than to measurement bias, as other categories showed broader variation (e.g. inorganic and small organic ions, gases, metals and SOA markers).

**Figure 8:**
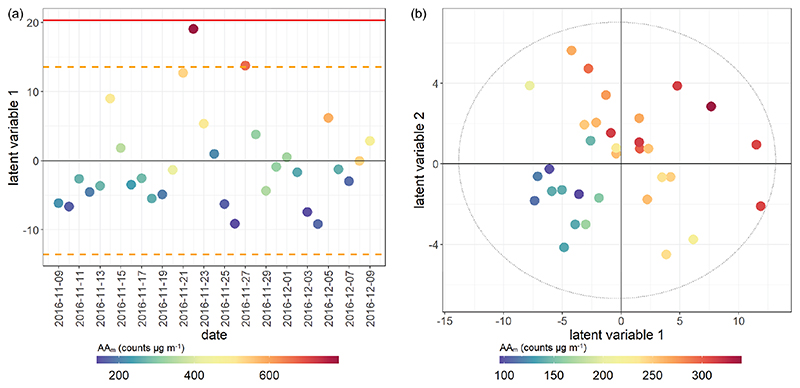
PLSR score plot for AA_m_ assay. Model performance parameters given in Table 2. **(a)** Winter samples; **(b)** summer samples. Points coloured by overall AA assay response for both seasons. Red bar indicates 2× SD for all scores; orange dotted line indicates 1× SD for all scores. Models which have only one latent variable have the x axis replaced by date for easier visualisation.

**Figure 9:**
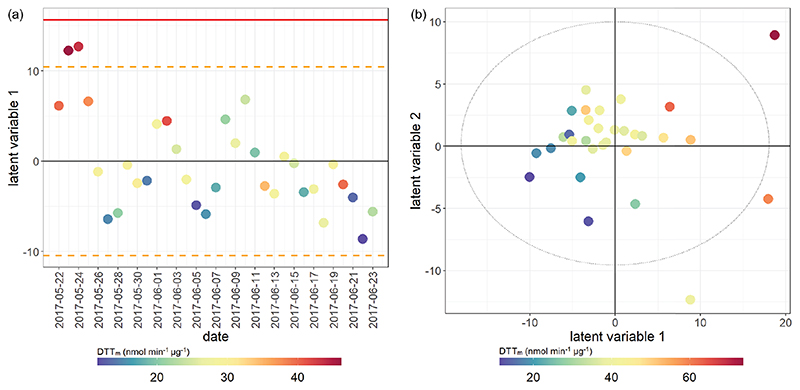
PLSR score plot for DTT_m_ assay. Model performance parameters given in Table 2. **(a)** Winter samples; **(b)** summer samples. Points coloured by overall DTT assay response for both seasons.

Partial least squares regression (PLSR) is a supervised regression extension of PCA, which models the variation in the data which is associated with a predefined sample classification (Eriksson et al., 2013). PLSR models were constructed for each individual OP assay and season to examine the most specific markers associated with seasonal assay response. Table 2 provides the performances for all PLSR models of OP assay response, and example PLSR score plots for all AA_m_ and DTT_m_ models are illustrated in Figs. 8 and 9 (analogous plots for other assays provided in Figs. S18 and S19). The performance indicators show that while the mass-normalised measurement data can be used to explain (*R* ^2^) and predict (*Q* ^2^) a large majority of the variation associated with AA_m_ summer/winter and DTT_m_ winter assay response, the other assay responses were less consistent; *R* ^2^ and *Q* ^2^ values for these models indicated that less than 70% of the variance in response can be predicted from the individual component measurements, and the predictions were much less stable through cross-validation. These results could suggest either that assay responses are not as adequately sensitive at μgμg^−1^ concentrations as for the total PM per sample, or that a proportion of the OP_m_ response is contributed to by species not measured directly in this campaign and which cannot also be inferred from total organic carbon measurements. As total OC is estimated from combustion properties of the sample rather than from a sum of individually validated component measurements, and as multiple organic and transition metal–organic complexed species contribute to the total OC measurements with unknown redox properties, these observations reiterate the need for more comprehensive chemical characterisation of PM. Similar to the univariate correlations, the summer samples were less well modelled in both mass-normalised and volume-normalised data, indicating either inadequate assay sensitivity (which may be compounded by the reduced collected filter PM mass in summer) or the influence of unmeasured components.

Table 3 shows the top 10 features in the variable importance in projection (VIP) for the PLSR loadings, which enable a ranking of the features which contribute most to the model (Naes and Martens, 1988). It is evident from these data that the features which best model the OP_m_ seasonal response are derived from multiple particle sources and atmospheric ageing processes. For example, the AA_m_ and DTT_m_ responses show similar trends in the multivariate models, but the main contributors to their responses have little overlap, with AA_m_ responses being more strongly associated with SOA tracers, PAHs, and general measures of organic carbon and the DTT_m_ responses more characterised by combustion and vehicle emissions markers (Figs. S19–S22; Figs. S17–S24 list the top 50 contributors to each assay model response). Notably, compounds which are not generally recognised as being redox-active were frequently observed to be important in PLSR classification, and though they do not directly contribute to the OP_m_ response, they are likely to be co-emitted with or be secondary products of redox-active particle components.

### 3.4 Multiple linear regression (MLR) modelling to predict OP_m_ associated with specific sources

While multivariate model loadings highlighted the measurements most associated with assay response, multivariate models are not always amenable to variable selection, which is important to characterise the chemical profiles contributing to each assay OP response. Multiple linear regression modelling has been used in previous studies (Calas et al., 2018) to establish contributors to total OP response, rather than looking at source apportionment from PMF models in relation to OP, and only simple forward variable selection was used for model refinement. In the present study, relevant measurements were grouped into six categories of known contributors to Beijing PM (biogenic SOA, biomass burning, coal and fossil power generation, cooking, dust, and vehicle emissions). The full method description, references, model formulae and performance parameters for the mass-normalised data models are presented in the methods (Sect. 2.3 “Statistical analysis”) and in Sect. S10. Briefly, literature sources (Table S13, Sect. S10) and the SPECIEU-ROPE database (Pernigotti et al., 2016) were used to establish which individual chemical measurements were likely to be characteristic of each source, with several measurements appearing in multiple categories (e.g. total EC). All proxy and composite measurements (except total EC, as multiple organic carbon species are represented in the dataset, but elemental carbon should be independent of the majority of these), AMS measurements, and general atmospheric measurements including temperature, relative humidity and actinic flux measurements were excluded from models entirely. Composite measures duplicate selected individual measurements; atmospheric measurements complicate model interpretation and are more likely to be useful as random effects terms in a mixed effects model approach (not pursued in the present study due to the complexity of model parameterisation and measurement uncertainties). Multiple linear regression models were then constructed for each assay and season for each category, using both mass-normalised and volume-normalised data.

MLR models further reinforced that not all putative sources and components of PM_2.5_ contribute equally to OP_m_ response (Table 4). OP_m_ assay response models based on measurements characteristic of vehicle emissions, coal/fossil fuel combustion and biomass burning gave accurate and robust predictions of OP_m_, and these are important contributors to PM (reported as mass per volume) in Beijing urban background sites (Yu et al., 2013; Zheng et al., 2005). As expected, OP_v_ models also gave very good predictions for these source profiles but additionally gave improved models of OP_v_ for biogenic SOA and dust compared with the OP_m_ data. Although the same base sets of predictors for each source were used for each model (season, OP assay and PM normalisation), there was only partial overlap of the final selected predictors between models from the same source and season, again illustrating the complex dynamic between OP and overall mass/volume composition. As with the PLSR models, the most important contributors to regression models were often not redox-active species, indicating that they are probably influencing or contributing to the oxidation state of the redox-active PM components. As observed in the univariate and multivariate analyses, the summer samples gave less robust linear regression models (and thus OP predictions) from both mass- and volume-normalised data. However, AA and DTT measurements produced the best models for all source contributions, indicating that these assays might be most optimal for measuring OP in an urban environment, as they appear to reflect the variety and composition of PM sources well.

Vehicle emissions, biogenic SOA and winter biomass burning contributions to AA and DTT response (as measured by the model *R* ^2^ value) were generally comparable across both assays, contrasting with the findings of Fang et al. (2016), who observed greater OP response in positive matrix factorisation–chemical mass balance (PMF-CMB) models associated with traffic emissions for AA_v_ over DTT_v_, as well as biomass burning for DTT_v_ over AA_v_ in multiple locations in the southeastern United States. However, a more recent study conducted in the coastal areas adjacent to Beijing (Liu et al., 2018) observed similar seasonality to the present study in the DTT OP_m_ response. Vehicle emissions (Wang et al., 2016; Yu et al., 2019), coal combustion (Ma et al., 2018; Yu et al., 2019), biomass burning (Ma et al., 2018) and dust (Yu et al., 2019) sources have been shown in other studies using PMF models to contribute to OP_v_ in Beijing, all using the DTT assay. Cooking markers (palmitic acid, stearic acid and cholesterol) contributed a substantial proportion of the known organic fraction of the PM mass and volume concentrations (see Fig. 4) but did not contribute robustly to the modelled OP response for either normalisation type, suggesting that either (i) they are not strongly contributing to or affected by oxidative conditions in PM or (ii) their variation over the sampling period cannot be linearly modelled. Similarly, biomass burning markers contribute a comparable number of variables in the model base sets but appear to contribute much more significantly to the OP_v_ than to the OP_m_ response. Biogenic SOA and dust models (which incorporate K^+^, Na^+^, Ca^2+^, Cl^−^, Al, Ti, Mn, Fe and Zn) explained a significant proportion of winter OP_v_ responses but were only strongly correlated with winter AA and DTT for mass-normalised models. These observations suggest these sources contribute to PM OP_v_ by total quantity rather than through their particularly high intrinsic OP - i.e. their mass as a proportion of the PM mass is smaller, but their concentration per volume is high - and the AA and DTT assays have a notable selectivity for these species over the EPR and DCFH assays.

It should be noted that the MLR models represent a suboptimal prediction of the OP response from measured components, as numerous species which are known source components (e.g. PAHs from combustion processes and those which distinguish gasoline from diesel vehicle emissions, or VOCs relevant to biomass burning such as methanol or acrolein) could not be included in models. Not all measurements which were associated in the literature with a particular assay response passed the stages of variable selection for mass-normalised models, which could reflect a lower limit of detection either in the OP_m_ assay responses or in the individual component measurements. Synergistic effects between individual measured components (e.g. transition metals with organic components such as quinones or carboxylic acids, Wang et al., 2018) cannot be interpreted from linear models when the complexation and oxidation states of the contributing compounds are essentially unknown. MLR models do not fully account for the proportion of each measurement which may originate from multiple emissions sources, and PMF-CMB or mixed effects models can address this issue more adequately. Validation of both the multivariate and MLR models using secondary datasets (both from Beijing and other locations) is also needed prior to their future implementation.

## 4 Conclusions

This study presents a detailed and comprehensive analysis of PM_2.5_ oxidative potential and particle-bound ROS concentrations measured in winter 2016 and summer 2017 during the APHH-Beijing campaign at a central site in Beijing, China. Four acellular methods for measuring OP were applied, providing a broad assessment of the oxidative properties of particles including particle-bound ROS concentrations, superoxide radical production and catalytic redox activity. We correlated the acellular assay responses with an extensive and comprehensive dataset including 107 additional atmospheric measurements (particle components, trace gases, meteorological parameters) to delineate chemical particle components and atmospheric processes and sources responsible for driving PM_2.5_ OP. Higher volume-normalised and mass-normalised OP values across all assays were observed in the winter compared to the summer. An inverse correlation was observed between AA_m_ and DTT_m_ with overall PM_2.5_ mass concentrations; i.e. days with higher PM_2.5_ mass concentrations have lower intrinsic OP values. This is likely due to an increase in OP-inactive material in high PM_2.5_ mass days and/or a mass fraction that is at present undetermined and highlights that a focus on total PM exposure only does not necessarily capture accurately the oxidising properties and therefore certain toxicological effects of PM.

Univariate analysis with the additional 107 measurement parameters acquired during the APHH-Beijing campaign highlight significant assay-specific responses to chemical components of PM_2.5_, as well as a seasonal difference in what components drive aerosol OP. It also highlights the importance of considering both volume-normalised and mass-normalised OP metrics when drawing conclusions on the role of chemical composition on OP, as assay correlations vary significantly between the two metrics. The data presented in this study illustrate that mass-normalised OP_m_ values provide a more nuanced picture of specific chemical components and sources that influence intrinsic OP, whereas many more correlations with OP_v_ values are observed, likely due to collinearity of many chemical components with overall PM_2.5_ mass concentrations driven by changes in meteorological conditions. Both metrics, mass-normalised OP_m_ as well as volume-normalised OP_v_, are important to consider with OP_v_ a more relevant metric with respect to exposure and epidemiological studies, whereas OP_m_ provides more insight into what sources and what composition drives OP concentrations in particles. Furthermore, OP_m_ may allow easier study and site inter-comparisons.

The multivariate statistical analyses encapsulated the observations from the univariate analyses into comprehensive single models of OP relating to PM composition, mirroring the observations in the univariate analyses that OP_m_ measured by each assay is related to different compounds present in the particle. It is clear from these differences that assay chemistry must contribute directly to its chemical selectivity, as the independent chemical measurements were given equal analytical weight with respect to each assay. The relationship between each assay and the independent measurements also confirmed that while there may exist a correlative relationship between an assay and non-redox-active compounds such as *n*-alkanes or PAHs, the assay is more likely to be measuring either secondary oxidation products of these primary compounds or species co-emitted that contribute to particle OP. This represents a gap in the chemical analysis of these samples, and more detailed redox-active compound speciation is required, especially for functionalised organics. Furthermore, variable selection of measurements and evaluation through multiple linear regression models indicated that OP_m_ is well predicted by measurement panels characteristic of combustion sources, particularly (exhaust and nonexhaust) vehicle emissions and biogenic SOA. This study demonstrates further that these commonly applied acellular assays are sensitive to a wide and differing range of chemical components, highlighting the advantage of using these assays as a they encompass multiple chemical components and sources of aerosol into an integrated measurement. Further comprehensive work is needed to identify the direct links between these OP assays and biological and toxicology data.

## Supplementary Material

The supplement related to this article is available online at: https://doi.org/10.5194/acp-21-5549-2021-supplement.

Supplementary material

## Figures and Tables

**Table 1:** Correlation of volume-normalised (OP_v_, top panel) and mass-normalised (OP_m_, bottom panel) assay responses in the winter (upper right values, regular font) and summer (lower left values, italic font) campaign. It should be noted that assay responses expressed as mass-normalised (OP per μg) are correlated with mass-normalised additional particle-phase composition measurements (i.e. μg or ng per μg PM_2.5_).

OP_v_ R_s_	AA_v_	DCFH_v_	EPR_v_	DTT_v_
AA_v_		0 89***	0.86***	**0.83** ^***^
DCFH_v_	0.35*		**0.86** ^***^	0.72***
EPR_v_	*0.19*	*0.01*		**0.88** ^***^
DTT_v_	0.41*	0.58***	*0.07*	
OP_m_ R_s_	AA_m_	DCFH_m_	EPR_m_	DTT_m_
AA_m_		−0.29	0.22	**0.60** ^**^
DCFH_m_	*−0.20*		−0.08	−0.15
EPR_m_	*−0.26*	*0.15*		0.27
DTT_m_	*0.20*	*−0.28*	*0.14*	

Bold font indicates Rs ≥ 0.5; * p< 0.05, ** p < 0.01, *** p < 0.001.

**Table 2:** Performance assessment of PLSR models for all assays, for both mass-normalised and volume-normalised data. Models are considered to perform well when both cumulative (i.e. across all latent variables included in the model) *R* ^2^ and *Q* ^2^ values are high, or at a minimum where *Q* ^2^ values are within 10% of the *R* ^2^ value, indicating that the variance is well accounted for in model cross-validation. Permutation tests were rejected for robustness if any single random permutation model performance surpassed the performance of the real cross-validated model; on this basis, the winter DCFH_m_ and summer DTT_v_ models were rejected (highlighted with *), although fewer than three random models outperformed the real model, and none of the permuted model *Q* ^2^ values outperformed those of the real model.

		Mass(μgμg^1^)				Volume (μgm^3^)			
Assay	Season	Optimal LVs	Cumulative _R_2	Cumulative _Q_2	Permutation test pass	Optimal LVs	Cumulative _R_2	Cumulative _Q_2	Permutation test pass
EPR	winter	1	43.2	19.3	no	2	83.9	75.2	yes
	summer	1	11.3	−10.0	no	1	52.0	3.7	no
AA	winter	1	81.4	78.2	yes	2	94.1	87.9	yes
	summer	2	79.3	49.7	yes	1	41.8	22.6	no
DTT	winter	2	76.0	62.0	yes	2	86.8	67.0	yes
	summer	1	47.4	31.6	no	1	66.2	50.9	no^*^
DCFH	winter	2	71.9	50.4	no^*^	2	67.0	55.2	yes
	summer	1	28.2	−6.6	no	1	86.0	66.7	yes

**Table 3:** Characteristic loadings most influential in PLSR models of OP_m_ as defined by ordered variable importance in projection for each model. Upward arrows indicate positive correlation with the assay measurement, downward arrows for inverse correlation and * for p < 0.05 in Spearman correlation of the feature with the assay in the univariate analysis.

EPR_m_ winter		AA_m_ winter		DTT_m_ winter		DCFH_m_ winter	
feature	VIP	feature	VIP	feature	VIP	feature	VIP
indeno(1,2,3-cd)-pyrene*	2.12 ↑	*cis*-pinonic acid^*^	1.44 ↑	SO_2_*	1.46 ↓	NH_4_^+^	2.16 ↑
acenaphthylene	2.02 ↑	Cl^−^*	1.42 ↑	Ca^2+^*	1.40 ↑	chrysene*	1.61 ↓
benzo(ghi)-perylene^*^	2.01 ↑	total OC*	1.33 ↑	Fe*	1.37 ↑	benzo(b)-fluoranthene*	1.59 ↓
benzo(a)pyrene^*^	2.01 ↑	MOOOA*	1.30 ↑	fluorene	1.34 ↑	RH8*	1.59 ↑
fluorene	1.82 ↑	pyrene*	1.30 ↑	acetaldehyde*	1.33 ↓	benzo(a)anthracene*	1.58 ↓
benzo(a)-anthracene^*^	1.81 ↑	2-methylthreitol	1.29 ↑	phenanthrene^*^	1.33 ↑	pyrene^*^	1.58 ↓
dibenzo(a,h)-anthracene^*^	1.80 ↑	ORG*	1.29 ↑	acetone^*^	1.33 ↓	LOOOA*	1.57 ↑
phenanthrene^*^	1.77 ↑	benzo(k)-fluoranthene*	1.29 ↑	Cl^−^*	1.31 ↑	fluoranthene^*^	1.56 ↓
chrysene^*^	1.66 ↑	3-methyl-2,3,4-trihydroxy-1-butene*	1.28 ↑	benzene^*^	1.31 ↓	RH120*/RH240*	1.55 ↑ 1.55 ↑
naphthalene^*^	1.62 ↑	fluoranthene*	1.27 ↑	toluene*	1.30 ↓	K^+^*	1.51 ↑
EPR_m_ summer		AA_m_ summer		DTT_m_ summer		DCFH_m_ summer	
feature	VIP	feature	VIP	feature	VIP	feature	VIP
LOOOA	2.59 ↑	ORG*	1.80 ↑	OH	1.58 ↑	cis-pinonic acid*	2.38 ↓
T8/T120/T240	2.28/2.15/ 2.08 ↑	*cis*-pinonic acid^*^	1.62 ↑	dibenzo(a,h)-anthracene*	1.51 ↑	C31*	1.76 ↓
O_3_	2.00 ↑	MOOOA*	1.58 ↑	C26*	1.48 ↑	pinic acid*	1.74 ↓
RO_2_*	1.76 ↑	cholesterol	1.58 ↓	benzo(a)-pyrene*	1.48 ↑	acetonitrile^*^	1.69 ↑
galactosan^*^	1.74 ↓	naphthalene^*^	1.57 ↑	total OC*	1.46 ↑	3-methyl-2,3,4-trihydroxy-1-butene	1.65 ↓
K^+^	1.70 ↑	palmitic acid^*^	1.49 ↑	C30*	1.46 ↑	benzo(ghi)-perylene	1.62 ↓
17a(H)-22,29,30-trisnorhopane (C27a)	1.55 ↓	RH8	1.39 ↓	C28*	1.43 ↑	C32	1.61 ↓
*cis*-2-methyl-1,3,4-trihydroxy-1-butene	1.55 ↑	stearic acid^*^	1.39 ↑	benzo(ghi)-perylene*	1.41 ↑	dibenzo(a,h)-anthracene*	1.61 ↓
Ba	1.47 ↓	benzo(ghi)-perylene*	1.36 ↑	C33*	1.40 ↑	acetaldehyde^*^	1.61 ↑
RH8	1.46 ↓	benzo(a)-pyrene*	1.34 ↑	C29*	1.39 ↑	isoprene^*^	1.61 ↓

**Table 4:** *R*^2^ values for optimised subset multiple linear regression models of relevant source contributions. *R* ^2^ values greater than 0.7 are highlighted in bold. Full model performance indicators are provided in Sect. S11 of the Supplement, including all model terms, residuals, coefficients and p values.

		EPR *R* ^2^		AA *R* ^2^		DTT *R* ^2^		DCFH *R* ^2^	
	source model	winter	summer	winter	summer	winter	summer	winter	summer
(μgμg^−1^)	vehicle emissions	**0.88**	**0.72**	**0.95**	**0.73**	**0.91**	**0.80**	**0.89**	0.62
	biomass burning	0.41	0.29	0.49	0.47	0.45	0.41	0.58	0.31
	coal/fossil fuel combustion	**0.84**	0.56	**0.88**	0.61	**0.86**	0.68	**0.75**	**0.71**
	cooking markers	0.19	0.11	0.66	0.20	0.39	0.36	0.08	0.24
	dust	0.23	0.23	**0.88**	0.47	**0.72**	0.46	0.50	0.26
	biogenic SOA	0.55	0.35	**0.95**	**0.74**	**0.79**	0.61	0.55	**0.70**
(μgm^−3^)	vehicle emissions	**0.94**	**0.79**	**0.97**	**0.74**	**0.96**	**0.87**	**0.94**	**0.86**
	biomass burning	**0.85**	0.23	**0.89**	0.24	**0.72**	0.62	**0.78**	0.53
	coal/fossil fuel combustion	**0.91**	0.69	**0.95**	0.62	**0.88**	**0.77**	**0.93**	**0.91**
	cooking markers	0.10	0.08	0.09	0.22	0.10	0.44	0.11	0.49
	dust	**0.79**	0.21	**0.92**	0.30	**0.78**	0.54	**0.73**	0.63
	biogenic SOA	**0.87**	0.36	**0.84**	0.59	**0.80**	0.63	**0.94**	**0.90**

## Data Availability

All R code used for statistical data analysis and visualisation can be found at the Beijing GitHub repository, https://github.com/katewolfer/Beijing (last access: 6 April 2021, https://doi.org/10.5281/zenodo.4665696. Wolfer, 2021). All code was written by Kate Wolfer, except for the named package dependencies stated in the code.

## Data availability

All statistical analyses, processed data and model parameters are available in the Supplement. Raw data have not been made fully available as they will be used for additional publications. Selected raw data are available on request; please contact the authors.
