# Supplementary material for: Atmospheric conditions and composition that influence PM2.5 oxidative potential in Beijing, China
Source: Atmos Chem Phys. Author manuscript; Available in PMC 2021 Aug 29. (PMC7611584; doi:10.5194/acp-21-5549-2021)
Supplement: Supplementary material [file EMS133136-supplement-Supplementary_material.pdf]

Supplement of Atmos. Chem. Phys., 21, 5549–5573, 2021
https://doi.org/10.5194/acp-21-5549-2021-supplement
© Author(s) 2021. CC BY 4.0 License.

Supplement of

Atmospheric conditions and composition that influence $PM_{2.5}$ oxidative potential in Beijing, China

Steven J. Campbell et al.

Correspondence to: Steven J. Campbell (stevenjohn.campbell@unibas.ch)

The copyright of individual parts of the supplement might differ from the article licence.

Section S1 : APHH site location

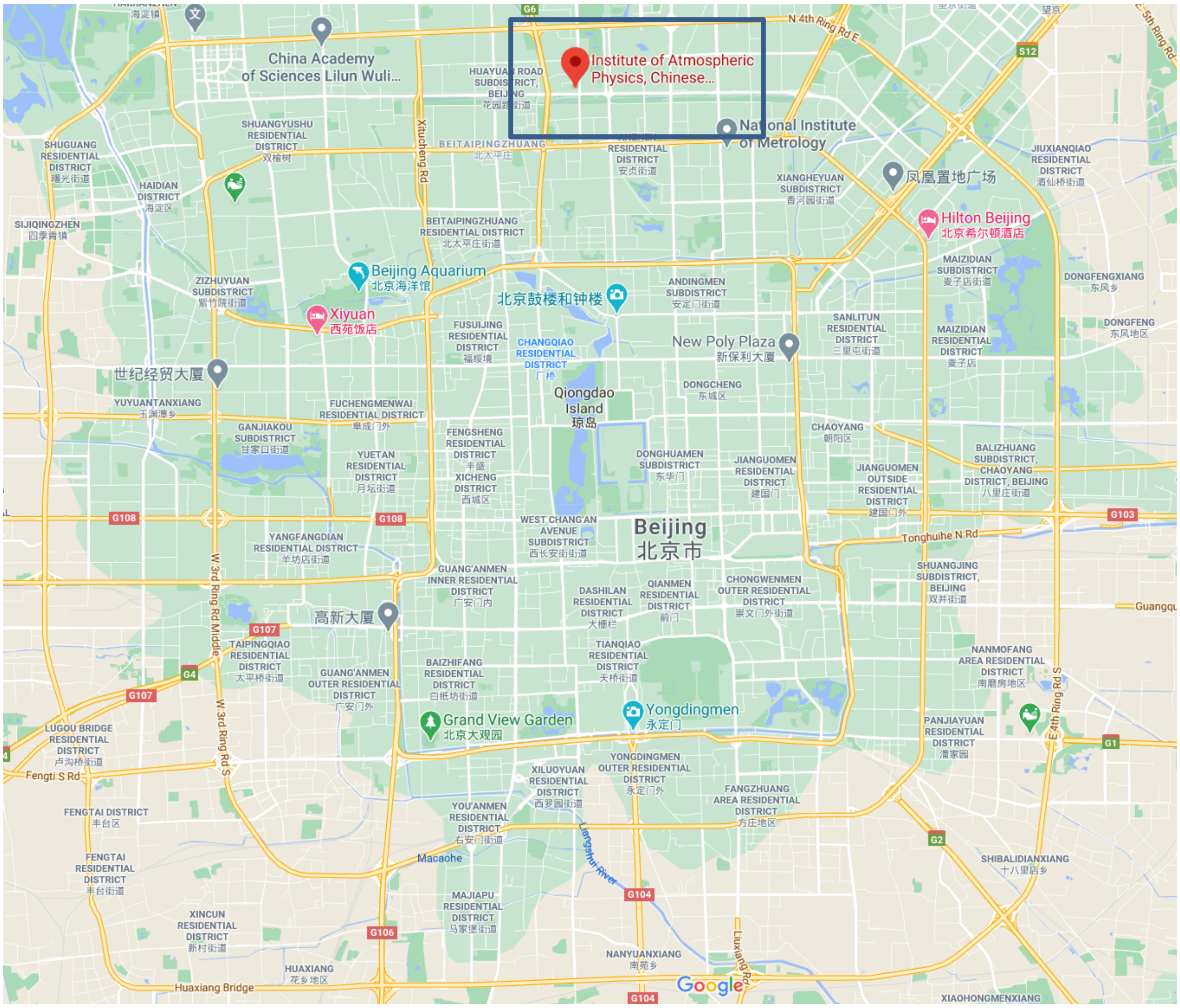

Figure S1. Aerosol samples were collected at the Institute of Atmospheric Physics (IAP) in Beijing, China. Winter PM was collected during the months of Nov-Dec 2016 and summer PM was collected during the months of May-June 2017. A $PM_{2.5}$ high volume air sampler (RE-6070VFC, TICSH, USA) was used at a flow rate of ~1.06 m^3/min. PM was collected onto quartz microfiber filters (Whatman, 20.3 x 25.4 cm) with a collection area of 405 cm^2. Image taken from Google Maps (Last access: 07.09.2020). © Google Maps

Section S2: OP assay selection and biological relevance

Current knowledge on oxidative potential assays used for the assessment of PM health impacts has recently been comprehensively summarised by Bates et al. (2019). In this extensive review, the authors define oxidative potential as "the catalytic generation of ROS by … inhaled components with simultaneous depletion of antioxidants", making a clear distinction between the ROS species (hydrogen peroxide, superoxide, hydroxyl radical, organic peroxides) already present in PM (sometimes referred to as particle-bound ROS), and the ROS and oxidative effects which are generated by particle components after inhalation or absorption *in situ* i.e. on epithelial surfaces, and elsewhere in the body (referred to as intrinsic or particle-induced OP). In the case of intrinsic OP measurement being specifically required, the rate of ROS production over a time period is analysed (Bates et al., 2019; Calas et al., 2017), and is ideally conducted with a biologically appropriate medium such as surrogate lung fluid (SLF). The full distinction between particle-bound ROS and intrinsic OP can only be accurately apportioned in conjunction with detailed chemical characterisation of PM, which is a major motivation of the present study. However, OP can only relate directly to health effects when PM composition-assay-biological response relationships are established, which will be discussed below.

The four assays utilised in the present study were selected for their general use in acellular OP measurements; each has advantages and drawbacks, and appear to have at least partial selectivity for specific PM components. The samples used in our study were incubated during the assay procedures, but reported in concentration terms, and thus we consider that they represent both particle-bound ROS and intrinsic OP. There is representational overlap of PM composition between OP assays (Godri et al., 2011; Janssen et al., 2014; Yang et al., 2014a), the extent and details of which have not been always been fully apparent in previous studies, which generally do not employ all four assays simultaneously and do not acquire such additional comprehensive PM chemical profiles (the broadest example we have encountered is the work of Calas et al. (2018), which was conducted in Chamonix, France, using PM_{10} samples, and incorporated a respiratory tract lining fluid assay in addition to the four assays we have employed). An important secondary concern with respect to measuring OP in PM is capturing the extent and biological impacts from translocating particles both in lung tissues (Oberdörster et al., 1992) and systemically (Oberdörster et al., 2004). However, the translocation of particles still cannot be easily measured outside cell and animal studies, and accounting for translocation effects (other than the total oxidative burden exerted by PM) is outside the practical scope of OP assays, as it would need the proportion of translocating particles to be at least estimable, and for particle composition to remain stable during migration (Peters et al., 2006), which is unlikely.

A particularly critical aspect of assay measurement is sample collection and preparation. Filters are most frequently used for aerosol sample collection, and can only represent the complete sample in OP assays insofar as the choice of extraction conditions allows. Assays are most often used with pure water extracts, or occasionally methanolic filter extracts (Yang et al., 2014b), which give rapid, simple and reproducible extractions and facilitate comparability between studies. However, with reference to a real biological context, the "water-soluble" component of PM is almost purely a technical term (Calas et al., 2017); the scope of differences between experimental extraction conditions and

real conditions in the lung is well illustrated in the comparison of the uptake of components of PM from dust standards by PBS as compared with simple simulated lung fluids (Pelfrêne et al., 2017). Although redox-active and polar small molecule species are generally by nature water-soluble (i.e. hydrophilic) (Pietrogrande et al., 2013), epithelial surfaces are complex mixtures of lipids, proteins and small molecules in aqueous media (Samet and Cheng, 1994), which change the absorption capacity of less hydrophilic compounds in PM (e.g. oxy-PAHs) when compared with pure deionized water, PBS, or even with methanol (Ayres et al., 2008). Complicating the assessment of the organic contribution to PM OP is the incomplete coverage of the vast catalogue of compounds which are present in PM (Nozière et al., 2015), which are often conflated into a single measurement of total organic carbon, which cannot be stratified or contextualised with respect to their physicochemical properties or ligand activity in PM (Ayres et al., 2008).

A criticism of OP assays is their simplicity of chemical interaction compared with the real biological sequelae that they are attempting to capture, but OP assays cannot be straightforwardly applied as direct biological proxies (Pietrogrande et al., 2019). Moreover, OP would be more difficult to measure routinely and in large-scale real-world studies if more complicated mixtures of chemical substrates were used. The ideal implementation of OP assays is through automated real-time ("online") measurements (Yu et al., 2019a), which are highly preferable to filter-based ("offline") measurements for their improved time resolution and capture of more transient and unstable species contributing to OP (Campbell et al., 2019; Venkatachari and Hopke, 2008). For online assay application, high throughput and simplicity of assay implementation, and the associated epidemiological interpretation, are more important than the precise reflection of physiological phenomena. Even synthetic lung fluid is a highly simplified system (see e.g. Samet and Cheng (1994) for a detailed breakdown of the components of lung fluids as compared with the mixtures used in e.g. Pelfrêne et al. (2017), including mucus, which is produced in the lung in response to inflammatory stimuli, would much further modify solubility, and is little mentioned in the context of OP assays), and is more complex than water or methanol extraction in terms of procedure and analysis (Calas et al., 2017; Pietrogrande et al., 2019). The addition of multiple biologically relevant compounds to the analytical substrate can detract from the total and holistic measurement of the OP of the sample; the reaction products from each added compound need to be monitored simultaneously from a single run or sample, which increases the technical demands of the assay (Calas et al., 2018), and the analytes may have interactions which may be modulated by matrix effects from the sample itself, and which may be non-linear in response. Targeted methods which are consistent and simple to execute are much more useful for combination with the much-needed extensive epidemiological and toxicological studies for validation of OP in assessing the health impacts of PM. Biological imitation as an analytical aim is better served by cellular assays, but establishing clear biological endpoints for cellular studies can be difficult (Ayres et al., 2008), there can be variability in results associated with the robustness and heterogeneity of cultured cells (Moreb et al., 2007; Watanabe et al., 2002), and cellular assays are not easily adaptable to high-throughput analysis. Cell cultures are not completely representative of the (alveolar) physiology and processes they are intended to emulate (Gebb and Stevens, 2004), as they consist of single cells in artificial media, rather than being embedded in tissues within the organ structure and surrounded by constantly exchanging interstitial fluids. Importantly, commonly used cell lines often exhibit features which render them less suitable for the representation of normal cells and cellular processes; for example,

both BEAS-2B cells (Reddel et al., 1988) and A549 cells are immortalised (Han et al., 2020), and the latter line is derived from non-small cell lung carcinoma cells (Mason and Williams, 1980).

With respect to the epidemiology related to the OP of PM, effects have been linked with lung inflammation (Janssen et al., 2015) and respiratory diseases such as asthma (Weichenthal et al., 2016b; Yang et al., 2016), negative cardiovascular outcomes (Abrams et al., 2017; Atkinson et al., 2016; Weichenthal et al., 2016a), and with diabetes prevalence (Strak et al., 2017). However, it should not be neglected that for health status (Chaves et al., 2007; Kelly et al., 1999), genetic/phenomic (Fuertes et al., 2020; Singh et al., 2007; Wang et al., 2001) and socioeconomic (Farhat et al., 2018; Mondal et al., 2010) reasons, the human response to the oxidative effects of PM will be variable, and the healthy human antioxidant baseline is broad in terms of capacity and response mechanisms. In addition, the interpretation of OP becomes complex in terms of potential health effects when considering the role and precise definition of antioxidants. Lipids, proteins, carbohydrates and DNA present in interstitial fluids and tissues all have antioxidant activity, in that they scavenge radicals and react with oxidising species, and although they are less effective than other defined antioxidant compounds such as ascorbic acid, α-tocopherol and coenzyme Q10, they are more abundant and more frequently the first point of encounter in the body by oxidants (Niki, 2010).

Finally, the utility of oxidative potential in the assessment of PM toxicity has only been demonstrated in a limited number of studies (Abrams et al., 2017; Atkinson et al., 2016; Janssen et al., 2015; Strak et al., 2017; Weichenthal et al., 2016a, 2016b; Yang et al., 2016). The range and nature of toxicological effects exerted across the human population by the oxidising components of PM, and the application of OP assays in epidemiological investigations into the health effects of PM, have both yet to be fully established, and large cohort epidemiological studies incorporating healthcare records, extensive study metadata collection and biological testing are needed. For the prevention and management of health impacts associated with PM (Brook et al., 2004; Yang and Zhang, 2018), and for the development of economic and environmental policies to adequately control air pollution (Feng and Zheng, 2019; Yang and Zhang, 2018), the detailed chemical composition of PM and how it relates to OP are required, encompassing multiple locations, proximities to different emissions sources and including granular time resolution to best inform decision making. Studies such as APHH-Beijing are one datapoint amongst the many that will be required to fully elucidate the links between OP and health.

Section S3: Detailed assay protocols

Filter extraction for AA and DCFH assays

Depending on the mass of aerosol collected on the filter, between 1-3 punches of the filter were collected (0.78 – 2.34 cm^2), to ensure the measurement was above the respective assay's detection limit. Filter homogeneity was tested using the DCFH assay, to make sure filter punches at different locations of each respective sample filter were consistent. Filter punches were taken with a Teflon cutter (i.d. = 10 mm) to avoid contamination from transition metals, which

could complicate quantification. The filter material was cut into small pieces and extracted into 1.5 mL of Milli-Q water (resistivity ≥ 18.2 M Ω cm^{-1}) and vortexed for three minutes. The resulting slurry was then extracted into a clean glass 5 mL syringe (Hamilton) via a home-built Teflon needle (i.d. = 1/8 in) connected via a Luer lock for analysis with the DCFH or AA assay, or extracted into a Teflon syringe fitted with a Luer lock (Medicine IVL05) to avoid trace metal contamination. The extraction mixture was then filtered through a 0.45 μm PTFE Iso-Disc filter (Supelco, 54144-U) to remove any remaining filter material, and split into three 833 μL aliquots for triplicate measurement. The OP of the resulting samples was then measured using the methods described below.

DCFH protocol

The DCFH/HRP assay used here is described in detail in elsewhere (Fuller et al., 2014) and will be briefly described here. DCFH was freshly prepared daily using the following procedure: 10 mg of DCFH-DA was dissolved in 10 mL methanol (Milli-Q), with vortexing to aid dissolution. This stock solution was stored in the freezer at -18°C for up to one week. 338 μL of DCFH-DA in methanol was reacted in the dark for 30 minutes with 2.77 mL NaOH (0.01 M) at room temperature. The reaction was subsequently quenched with 6.92 mL of 1 M potassium phosphate buffer solution. This solution was made up to 50 mL with water (Milli-Q) and stored on ice for maximum one day.

1.54 mg of HRP was dissolved in 50 mL of water (HPLC grade) to prepare a 10 units mL^{-1} stock solution, which was stored in the fridge for maximum one week. From this stock solution, a 1.38 units mL^{-1} reaction solution of HRP was prepared by taking 6.82 mL of HRP stock solution and adding the equivalent volume of 1 M potassium phosphate buffer. This solution was made up to 50 mL with water (Milli-Q) and stored on ice for maximum one day.

833 μL of filter extract (see Section "Filter Extraction for AA and DCFH Assays" in the Supplementary Information) was added to 1084 μL HRP and the equivalent volume of DCFH. The reaction mixture was incubated at 37°C for 15 minutes to allow complete reaction of the DCFH/HRP (Fuller et al., 2014). For each filter sample, three 833 μL aliquots of the filter extraction mixture were analysed in parallel as three technical replicates, to establish measurement uncertainty for each sample. Fluorescence measurements were then conducted in a modified cuvette holder (Ocean Optics model CUV). The fluorescent product 2,7-dichlorofluoroscein (DCF) was excited at $\lambda_{excitation}$ = 485 nm, fluorescence at $\lambda_{emission}$ = 520 nm by an LED (Roithner APG2C1-435 435 nm, 380 mW at 350 mA) at 2.56 V and 16 mA, connected to an optical fibre (Ocean Optics 00S-003948-07) that was subsequently coupled to an aspheric lens (Thorlabs, type C230TMD-A) to focus the light from the optical fibre into the cuvette holder. The same lens and optical fibre were then connected to a UV spectrometer (Ocean Optics UV2000+). The ROS concentration for each sample, in nmol H_2O_2 equivalents, was then calculated using a H_2O_2 calibration curve (**Figure S2**). Filter blanks and chemical blanks were performed on a daily basis to monitor assay stability over multiple days of sample analysis.

DCFH calibration curve

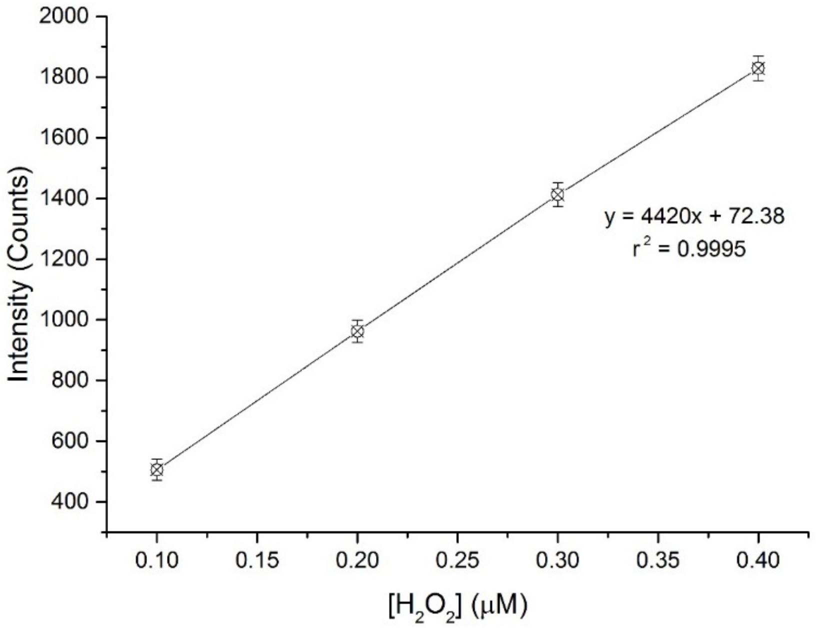

Figure S2. Calibration curve for DCFH solution, plotted using 0.1 μM, 0.2 μM, 0.3 μM and 0.4 μM standard solutions of H_2O_2, with the background signal subtracted. Errors are derived from three repeats of each measurement.

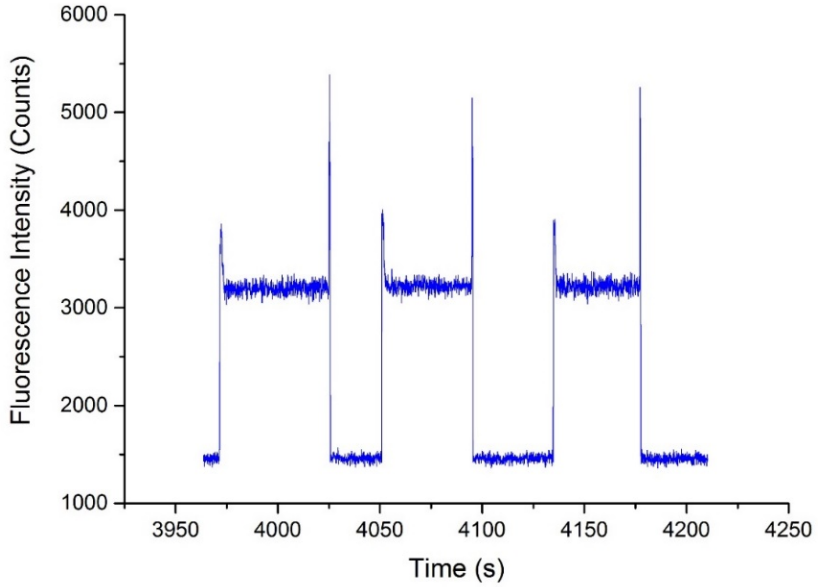

Figure S3. The raw spectrum above illustrates three repeat DCFH assay measurements of one sample, and the consistency between the repeats shown here is representative of that across all of the samples. The error of each individual repeat originates from the inherent "noise" in the signal from the detector and was calculated using the standard deviation of the fluorescence intensity. The errors from the three repeats were combined to give the total error in the average fluorescence intensity of one sample.

AA protocol

The ascorbic acid (AA) assay used in this study is described in detail in Campbell et al. (2019) and will only be briefly described here. The AA method quantifies the oxidation product of AA, dehydroascorbic acid (DHA). Under acidic conditions, DHA reacts with *o*-phenylenediamine (OPDA) to form the product 3-(1,2-dihydroxyethyl)fluoro[3,4-b]quinoxaline-1-one (DFQ) (Burini, 2007; Deutsch and Weeks, 1965), a highly fluorescent compound which can be detected by fluorescence spectroscopy. Due to the 1:1 reaction stoichiometry between DHA and OPDA, monitoring the change in concentration of DFQ can therefore be related to the extent of oxidation of AA, and hence the OP of the sample.

A 200 μM AA solution was prepared as described previously (Godri et al., 2011) in Chelex-resin treated Milli-Q purified water. The Chelex resin treatment was necessary to eliminate the presence of trace transition metals in already pure Milli-Q purified water (resistivity ≥ 18.2 M Ω cm^{-1}). 3 g of Chelex per 100 ml of solution was mixed for 24 hours (with constant stirring) before vacuum filtration in a Sartorius vacuum filtration unit, using a Whatman cellulose nitrate filter with 4.7 cm diameter and 0.45 μm pore size. This process was needed to ensure the background concentrations of transition metals were as low as possible, as their presence can cause the formation of DHA *via* ROS production or direct oxidation of AA. All solutions were prepared and contained in sterilized plastic bottles and containers to minimize contamination from trace metals and biological material. AA solutions were made fresh daily to ensure the background DHA concentration, formed from AA degradation in solution, was minimized, therefore keeping the background signal as low and stable as possible. OPDA was dissolved in 500 mL of 0.1 M HCl at a concentration of 46 mM.

833 μL of the filter extract in Milli-Q water at pH 7 (see Section "Filter Extraction" in the Supplementary Information) is added to 100 μL of AA and incubated at 37°C for 40 minutes. The pH of the working AA solution was 2.5, which at this stage of method development was required to improve the signal stability, however the filter extraction is performed at pH 7 (Campbell et al., 2019). OPDA (46 mM, 100 μL) was added to the reaction mixture, and allowed 10 mins to react at room temperature. The fluorescent product of the condensation reaction of DHA + OPDA, 3-(1,2-dihydroxyethyl)-fluoro[3,4-b]quinoxaline-1-one (DFQ), was excited at $\lambda_{excitation}$ = 365 nm with a high-power UV LED (Roithner Lasertechnik, type UVLED-365-330-SMD). The assay is then expressed in terms of the DHA concentration using a calibration curve of known DHA concentrations (**Figure S4**). Filter blanks and chemical blanks were performed on a daily basis to monitor assay stability over multiple days of sample analysis.

AA calibration curve

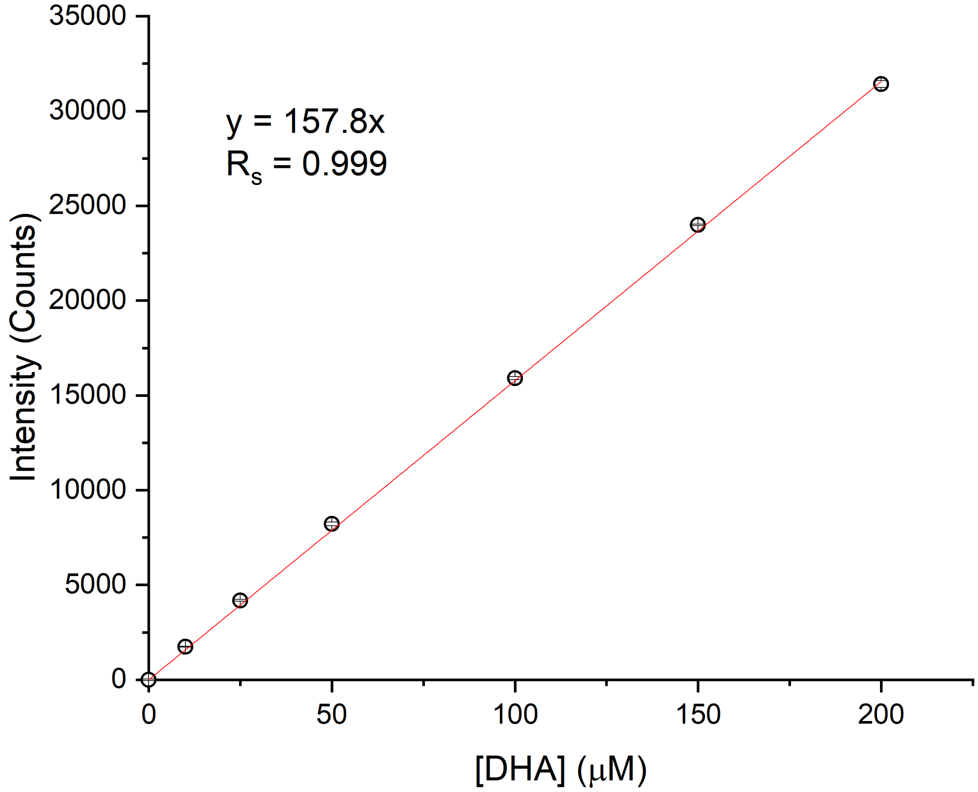

Figure S4. DHA calibration for AA assay.

DTT assay methods

The DTT protocol measures the oxidative potential of redox-active species in PM by adding DTT to PM extracts under biological conditions (37°C, pH = 7.4). The redox-active compounds in the PM oxidise the DTT to the disulfide form, and the rate at which this reaction occurs can be measured by periodically stopping the reaction by adding 5,5'-dithiobis(2-nitrobenzoic acid) (DTNB) in excess. DTNB reacts with the remaining DTT to form DTT-disulfide and 2-nitro-5-thiobenzoic acid (TNB), TNB is a coloured product that can be measured at 412 nm. By quenching the reaction at various times with DTNB the linear rate of DTT consumption can be determined. This rate of consumption is corrected for the mass of PM in the reaction to get an intrinsic DTT value: DTT_m (pmol DTT min^{-1} μg^{-1} $PM_{2.5}$), or volume normalised DTT_v (nmol DTT min^{-1} m^{-3}. At present, saturation effects at high $PM_{2.5}$ mass concentrations cannot be ruled out regarding the DTT assay (Charrier et al., 2016; Charrier and Anastasio, 2012).

DTT stock solution

The 10 mM DTT stock solution was prepared as follows: 0.154 g of DTT was quantitatively transferred to a 100 mL volumetric flask and dissolved in DI water. It was stored in amber glass bottles in a fridge. 10 mM DTNB: 0.396 g of DTNB was quantitatively transferred to a 100 mL volumetric flask and dissolved in methanol; the stock was then transferred to an amber glass bottle with glass stopper (wrapped in aluminium foil to block light) and stored in a fridge. 0.5 M potassium phosphate buffer: 0.5 M dipotassium phosphate (dibasic) was prepared by quantitatively transferring 8.71 g K_2HPO_4 into a 100 mL volumetric flask and dissolving in DI water.0.5 M monopotassium phosphate (monobasic) was prepared by quantitatively transferring 1.701 g KH_2PO_4 into a 100 mL volumetric flask and dissolving in DI water. The monobasic solution was added to the dibasic solution until the pH stabilised at 7.40 and stored at room temperature in an acid washed glass bottle.0.05 µM PQN preparation (working solution), the 5 mM PQN stock was stored in a volumetric flask in a fridge (defrosted using a 37°C water bath before use).

DTT $PM_{2.5}$ filter extraction

Rectangle (1 x 1.5 cm, SA 150 mm^2) and circular (0.8 cm Ø, SA 50 mm^2) punches were taken from each filter to have ~20 µg $PM_{2.5}$ in the reaction (two rectangle punches were used for filter blanks). The equation for working out PM in reaction is:

$$\frac{\frac{Total\ PM\ on\ filter\ (\mu g)}{Filter\ SA\ (mm^2)} \times \sum SA\ of\ punches\ \ (mm^2)}{Final\ extract\ V\ (mL)} \times V\ of\ sample\ in\ reaction\ (mL)$$

For 47 mm Teflon filters the equation would be:

$$\frac{\frac{Total\ PM\ on\ filter\ (\mu g)}{855\ mm^2} \times \sum SA\ of\ punches\ \ (mm^2)}{10\ mL} \times 0.7\ mL = PM\ in\ reaction\ (\mu g)$$

These punches were extracted in 5 mL methanol for 15 minutes *via* sonication; the extracts were then dried to ~1-2 mL using nitrogen blowdown. These extracts were then made up to 10 mL (volumes differed by ±5 mL in order to get the $PM_{2.5}$ in reaction to ~20 µg) using DI water and then extracted again for 15 minutes *via* sonication. The PM extract was then filtered through a 0.45 µm syringe filter, this filter extract was used in the same volume as PQN and DI water blank (0.7 mL).

DTT assay analysis

0.2 mM DTNB preparation: a 50 × dilution of the 10 mM DTNB stock was prepared by transferring 0.4 mL 10 mM DTNB into 19.6 mL DI water in a 50 mL amber glass bottle. 0.7 mL of 0.2 mM DTNB was transferred to 1.5 mL amber glass vials (5 vials per DTT run, one for each time point) (For other DTNB volumes: 29.4 mL DI & 0.6 mL DTNB, 39.2 mL DI & 0.8 mL DTNB).

0.7 mL of sample (PQN, PM extract, filter blank, or DI water) was transferred to an acid washed centrifuge tube with 0.2 mL 0.5 M k-buffer, this solution was heated to 37 ºC in a water bath. Immediately prior to starting the experiment a 1 mM DTT solution was prepared from the 10 mM DTT stock (5 mL 10 mM DTT stock made up in a 50 mL volumetric flask covered in foil to block light).

100 µL of 1 mM DTT was added to the sample / k-buffer solution. The solution was shaken and 100 µL of this solution was immediately transferred to an amber glass vial containing 0.2 mM DTNB, the coloured product of this reaction was immediately analysed using a dual-beam UV-vis.

At various time points (0, 10, 20, 30, and 40 minutes) 100 µL of the reaction solution was transferred to the 0.2 mM DTNB vials, each vial was immediately analysed using UV-vis. Three measurements were taken for each time point at 412 and 700 nm (700 nm is the background reading).

Two DTT runs were carried out at the same time, and the second run was off-set from the first by 5 minutes so that samples were analysed every 5 minutes. The second run used the same 1 mM DTT as the first run, but the DTT in the beaker was replaced by DTT in the volumetric flask. If more than two runs were carried out, the 1 mM DTT was be remade for each set of two runs.

During the DTT experiments for our samples, two filter blank DTT analyses were carried daily out along with three repeats of the first filter sample. To ensure the results were repeatable, the results for that day were only kept if the coefficient of variation for both of these were below 15%. To account for day-to-day drift, periodically PQN positive standards were run through the DTT experiment.

DTT calibration

For the calibration the solutions were not added to the water bath for heating and instead various concentrations of DTT solution were used. 0.7 mL of DI water and 0.2 mL 0.5 M k-buffer was added to five acid washed centrifuge

tubes. Each tube had 100 µL of a different DTT concentration added and then had 100 µL removed and added to 0.2 mM DTNB.The DTT concentrations used were:

- 100 µM: 5 mL in 50 mL DI
- 80 µM: 4 mL in 50 mL DI
- 60 µM: 3 mL in 50 mL DI
- 40 µM: 2 mL in 50 mL DI
- 20 µM: 1 mL in 50 mL DI
- 0 µM: 0.8 mL of DI water was used to take the place of DTT

The absorbance for each concentration was recorded in the same way as for samples and had the background reading at 700 nm and DI water absorbance subtracted from the absorbance at 412 nm. The absorbance (x-axis) was then plotted against DTT concentration (y-axis) to give the calibration curve:

The equation of the straight line was then used to determine DTT concentration from absorbance; DTT conc. (µM) = 280.51 * absorbance – 7.697.

DTT calibration curve

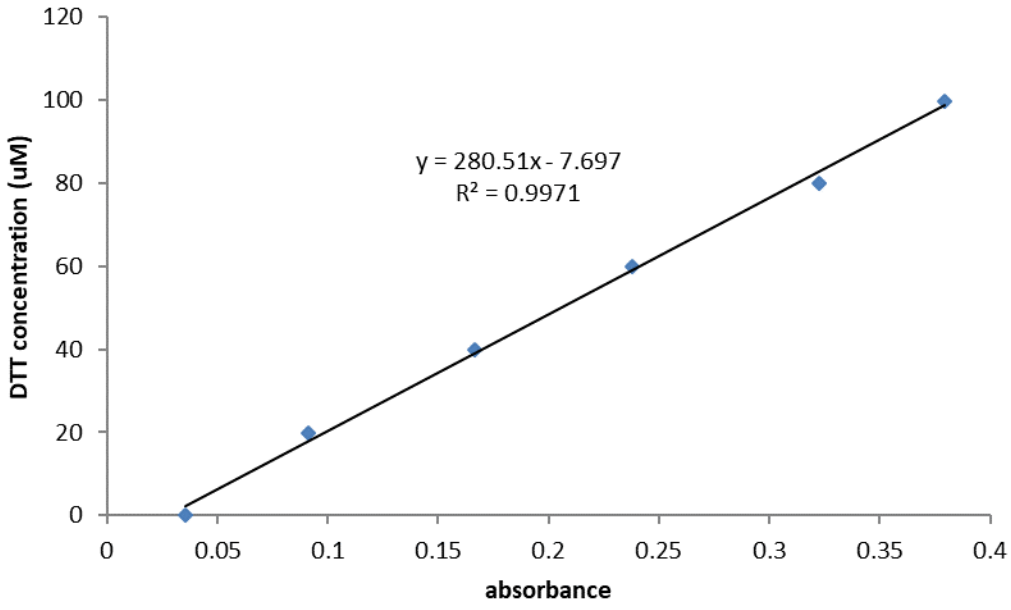

Figure S5. Calibration for DTT assay**.**

EPR protocol

PM suspensions were prepared fresh each day as a 1 mg/mL stock solution in a physiological buffer (Krebs buffer, composition in mM: 119 NaCl, 25 $NaHCO_3$, 5.5 D-glucose, 4.7 KCl, 1.17 $MgSO_4$, 1.18 KH_2PO_4, 2.5 $CaCl_2$), with 15 min sonication in a bath sonicator (FB15051; Fisherbrand, Loughborough, England).

Electron paramagnetic resonance was used to establish superoxide free radical ($O_2^{\cdot-}$) generation in the absence of cells or tissue. Suspensions were incubated with the spin trap, 1-hydroxyl-2,2,6,6-tetrame- thyl-4-oxo-piperidine (Tempone-H; 1 mM final concentration. Tempone-H can react with peroxynitrite, peroxyl radicals and other radicals, however, it shows preferential selectivity for superoxide (Dikalov et al., 1997). Previous work has shown that the Tempone-H EPR signal induced by particulates can be attenuated with superoxide scavengers, but not hydroxyl radical scavengers (Miller *et al*. 2009 & unpublished). Urban dust (UD; National Institute of Standards and Technology (NIST) SRM-1649a) and diesel exhaust particles (DEP; NIST SRM-2975) were used as reference material particles with known ability to generate superoxide in this assay. Pyrogallol, which spontaneously generates superoxide in aqueous solutions, was used as a non-particle positive control. The concentration of PM has been standardised so that all suspensions were tested at 0.3 mg/mL in physiological (Krebs) buffer.

Samples were kept at 37°C throughout and measurements were taken after 60 min by drawing 50 µL of sample into a capillary tube (VWR International, Lutterworth, UK) and sealing with a plug of soft sealant (Cristaseal, VWR International). An X-band EPR spectrometer (Magnettech MS-200, Berlin, Germany) was used with the following parameters: microwave frequency, 9.3-9.55 Hz; microwave power, 20 mW; modulation frequency, 100 kHz; modulation amplitude, 1500 mG; center field, 3365 G; sweep width, 50 G; sweep time, 30 s; number of passes, 1. Baseline signals (Tempone-H in buffer alone) were subtracted from that of experimental readings. Free radical generation was quantified using the first derivative of the initial peak of the spectra obtained from reaction of Tempone-H with superoxide.

Section S4: Filter homogeneity

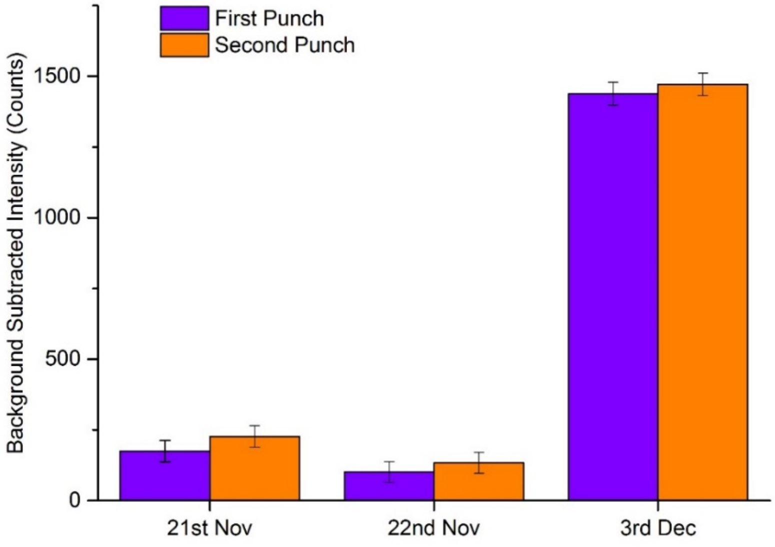

Figure S6*:* Testing the homogeneity of the filters and the DCFH assay by taking two separate punches of each of the samples from 21st Nov, 22nd Nov and 3rd Dec 2016 (winter campaign). The errors are derived from the three repeat measurements of each punch. In each sample the two separate punches were within error of each other, confirming the validity of using two combined punches in the extraction process to boost the signal for the "clean" days and then consequently scaling by a factor of a half. Taking and measuring the separate punches of each sample on different days also confirmed the repeatability of the DCFH/HRP assay.

Section S5: OP vs. $PM_{2.5}$ and average PM composition

Table S1. Summary of OP_v measurements for winter 2016 and summer 2017, including average $PM_{2.5}$ mass (ug m^{-3}).

**Assay (OP_v)**	**Season**	**Average $PM_{2.5}$ Mass (µg m^{-3})**	**Average Assay Response ***	**Spearman R_s (OP_v vs $PM_{2.5}$)**
**AA_v**	winter	98.7 ± 75	32.4 ± 14.8	0.89
**$DCFH_v$**	winter	98.7 ± 75	0.71 ± 0.52	0.96
**EPR_v**	winter	98.7 ± 75	$2.4\times10^6 \pm 1.6\times10^6$	0.89
**DTT_v**	winter	98.7 ± 75	2.9 ± 1.9	0.81
**AA_v**	summer	36.7 ± 16.8	8.5 ± 2.7	0.21
**$DCFH_v$**	summer	36.7 ± 16.8	0.17 ± 0.11	0.76
**EPR_v**	summer	36.7 ± 16.8	5.8×10^5	0.12
**DTT_v**	summer	36.7 ± 16.8	0.90 ± 0.40	0.61

Units: AA_v = DHA µM m^{-3}, $DCFH_v$ = nmol H_2O_2 m^{-3}, EPR_v = counts m^{-3}, DTT_v = nmol DTT min m^{-3}

Table S2. Summary of OP_m measurements for winter 2016 and summer 2017, including average $PM_{2.5}$ mass (µg m^{-3})

**Assay (OP_m)**	**Season**	**Average $PM_{2.5}$ Mass (µg m^{-3})**	**Average Assay Response ***
**OP_{AA}**	winter	98.7 ± 75	0.47 ± 0.23
**OP_{DCFH}**	winter	98.7 ± 75	0.0071 ± 0.0031
**OP_{EPR}**	winter	98.7 ± 75	7362 ± 1457
**OP_{DTT}**	winter	98.7 ± 75	37.5 ± 14.4
**OP_{AA}**	summer	36.7 ± 16.8	0.32 ± 0.10
**OP_{DCFH}**	summer	36.7 ± 16.8	0.0057 ± 0.0028
**OP_{EPR}**	summer	36.7 ± 16.8	4993 ± 2408
**OP_{DTT}**	summer	36.7 ± 16.8	26.5 ± 9.1

Units: OP_{AA} = DHA µg^{-1}, OP_{DCFH} = nmol H_2O_2 µg^{-1}, OP_{EPR} = counts µg^{-1}, OP_{DTT} = pmol min^{-1} µg^{-1}

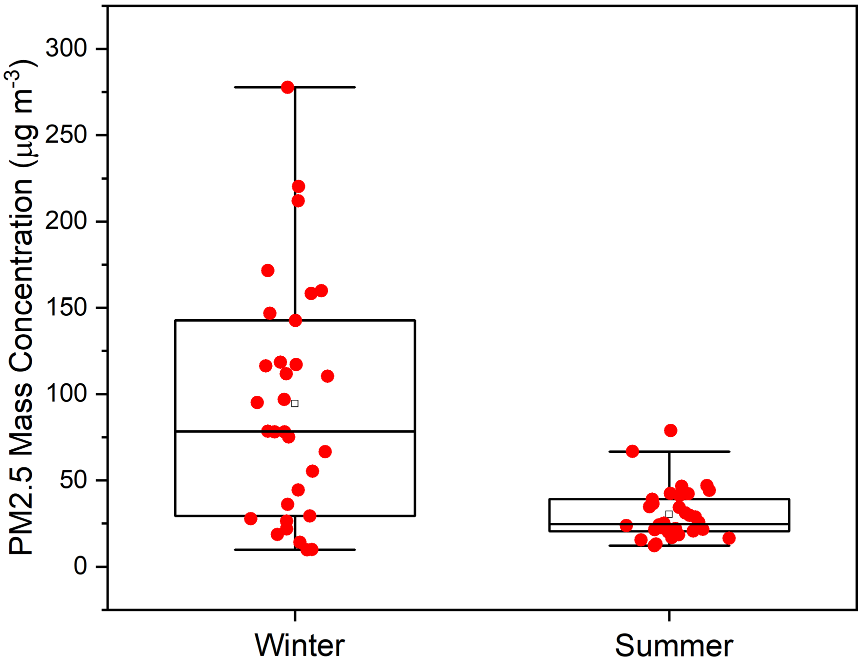

Figure S7. $PM_{2.5}$ mass concentrations in both the winter (2016) and summer (2017) campaign.

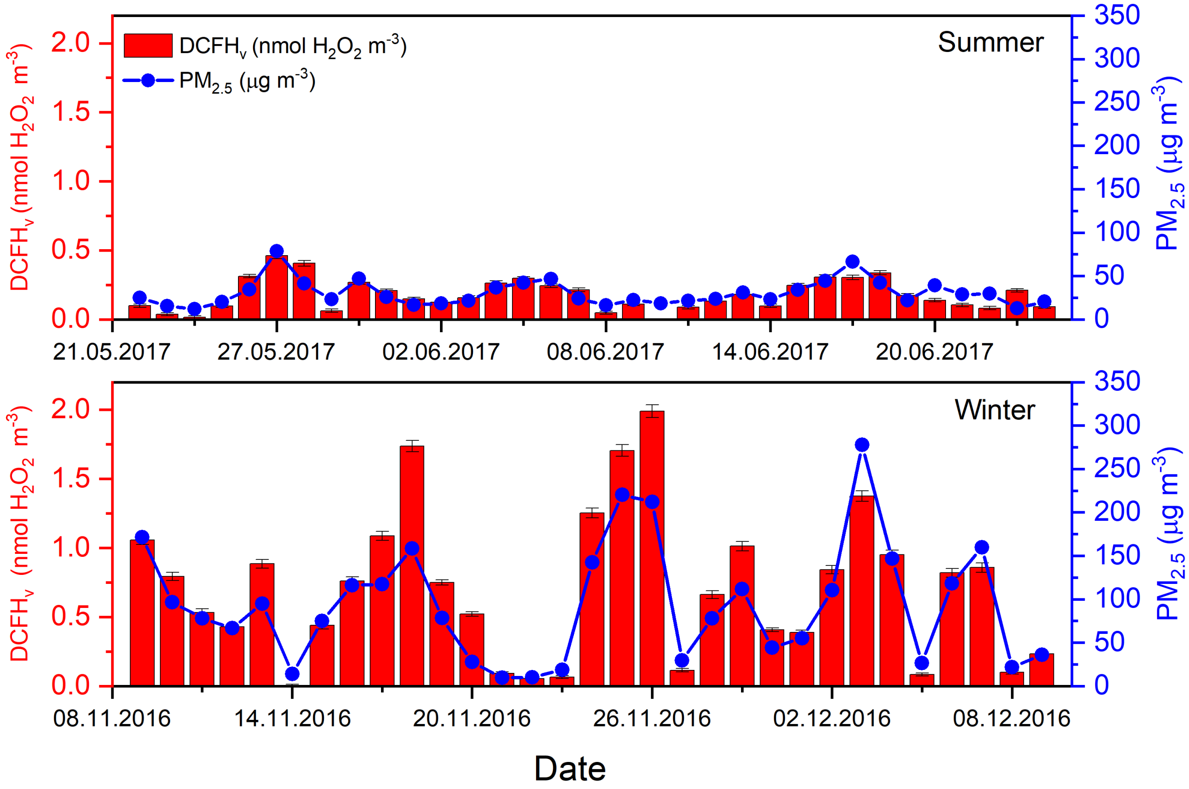

Figure S8. 24- hour averaged $PM_{2.5}$ mass (blue) and $DCFH_v$ (red), analysed from a 24-hour high volume filter (red) (see section Filter Collection), for both Winter 2016 (08/11/2016 – 08/12/2016) and Summer 2017 (21/05/2017-24/06/2017).

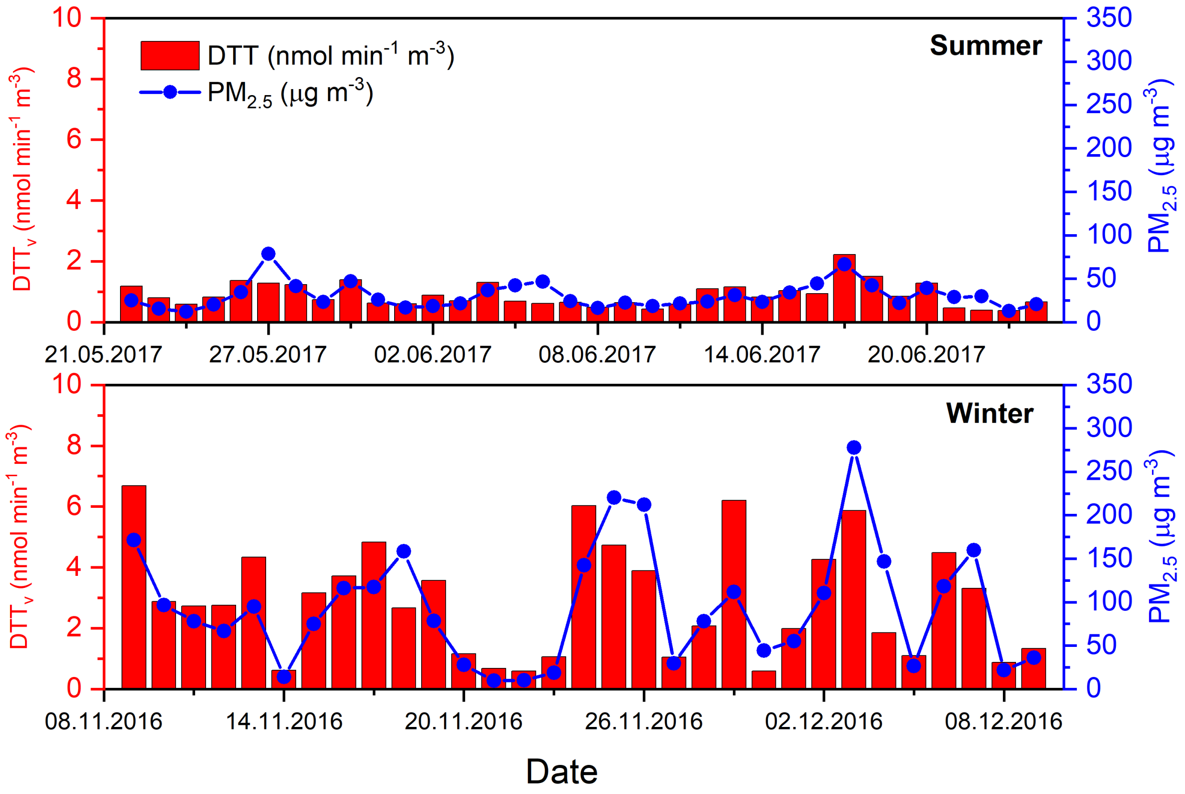

Figure S9. 24- hour averaged $PM_{2.5}$ mass (blue) and OP_{DTT} (red), analysed from a 24-hour high volume filter (red) (see section Filter Collection), for both Winter 2016 (08/11/2016 – 08/12/2016) and Summer 2017 (21/05/2017-24/06/2017).

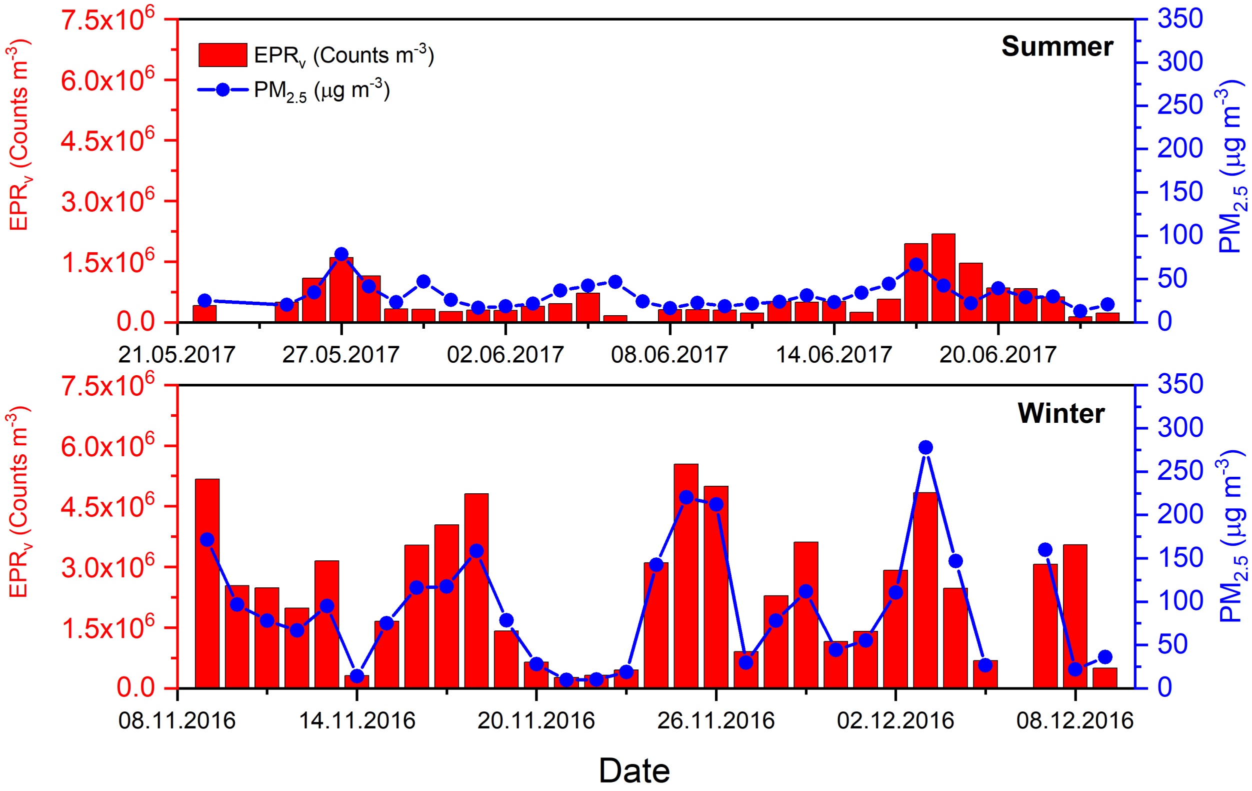

Figure S10. 24- hour averaged $PM_{2.5}$ mass (blue) and OP_{EPR} (red), analysed from a 24-hour high volume filter (red) (see section Filter Collection), for both Winter 2016 (08/11/2016 – 08/12/2016) and Summer 2017 (21/05/2017-24/06/2017).

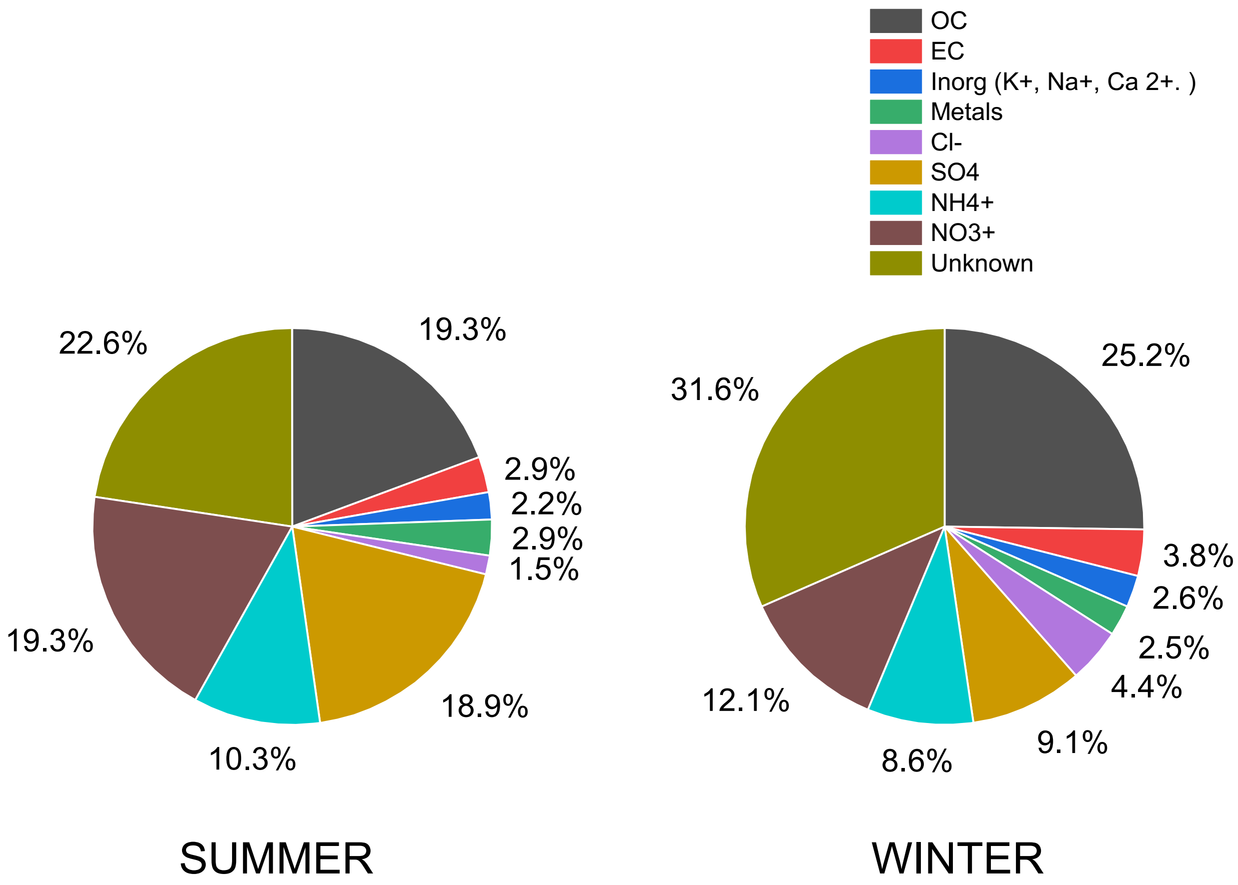

Figure S11. Averaged $PM_{2.5}$ composition in winter and summer.

Table S3. Cumulative scores where $R_s \geq 0.5$, out of a total of 117, for PM OP expressed as mass-normalised (OP_m) and volume normalised (OP_v).

	OP_m		OP_v	
**assay**	**winter**	**summer**	**winter**	**summer**
**AA**	54	15	67	4
**DCFH**	8	2	52	18
**EPR**	3	1	41	0
**DTT**	18	8	52	15

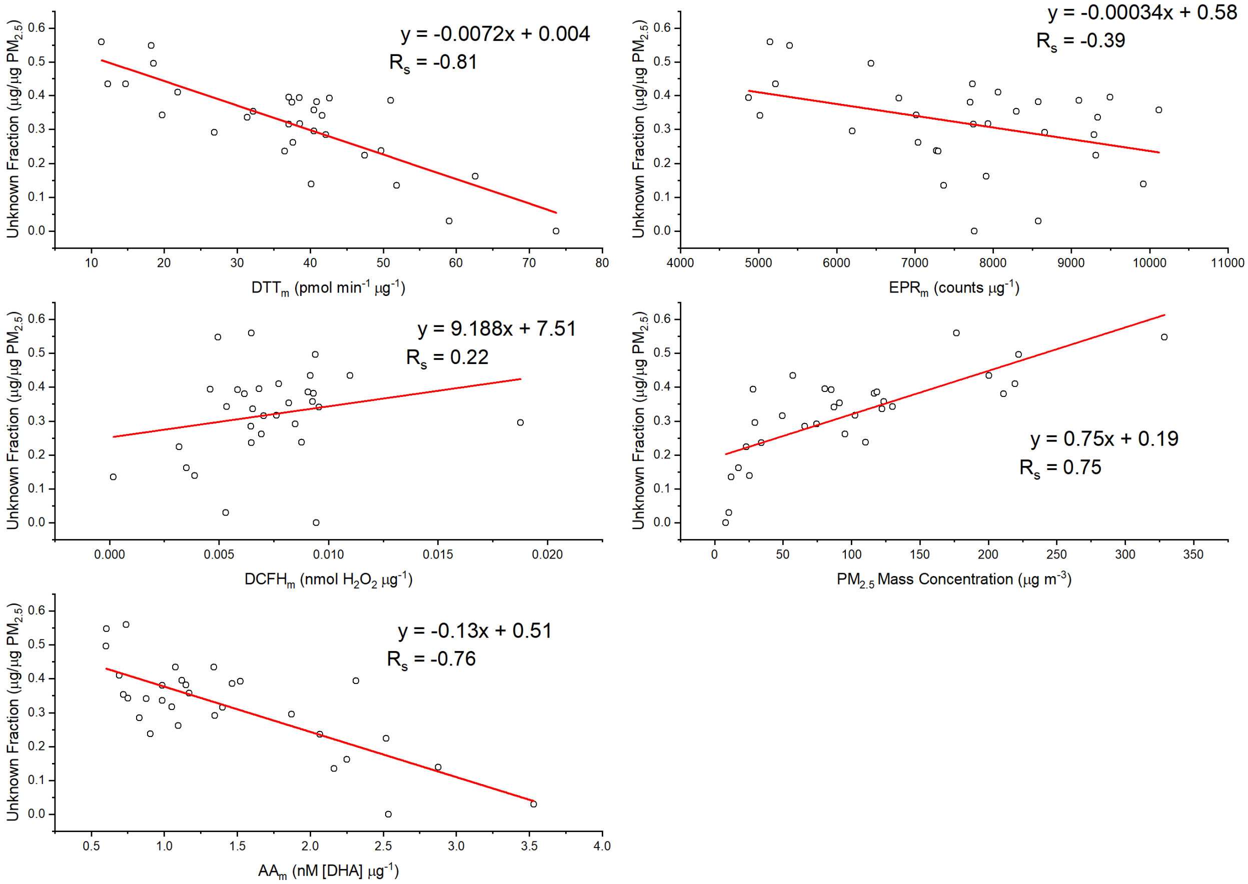

Figure S12. Correlations between OP_m values and the unknown composition percentage of $PM_{2.5}$ in this study. Statistically significant, inverse correlations are observed between AA_m and DTT_m with the unknown $PM_{2.5}$ composition, indicating that species within that fraction are responsible for the inhibition of $PM_{2.5}$ OP.

Section S6: Summary statistics for all measurements

Table S4 A. Summary statistics for all mass-normalised measurements. SOA tracer data is not presented as it will be used for future publications. Abbreviations: MW: Mann-Whitney-U test; SD: standard deviation; OC: organic carbon; EC: elemental carbon; ORG: total organic fraction (from AMS measurements); LOOOA: less-oxidised organic aerosol (from AMS measurements); MOOOA: more-oxidised organic aerosol (from AMS measurements); RH; relative humidity; T: temperature.

feature	units	seasonal MW p-value	winter mean	winter min	winter max	winter SD	summer mean	summer min	summer max	summer SD
**EPR**	counts μg^{-1}	8.08E-06	7632.26	4872.00	10120.00	1481.83	4980.68	944.00	11824.00	2448.26
**AA**	counts μg^{-1}	9.63E-03	331.04	141.37	783.31	166.36	224.06	95.52	339.58	74.24
**DTT**	nmol min^{-1} μg^{-1}	1.67E-03	37.23	11.40	73.70	14.66	26.44	11.60	46.10	9.30
**DCFH**	nmol H_2O_2 μg^{-1}	0.05	7.28E-03	1.75E-04	1.88E-02	3.16E-03	6.04E-03	1.30E-03	1.61E-02	2.88E-03
**total OC**	µg/µg PM	0.01	2.54E-01	1.20E-01	4.86E-01	9.64E-02	1.93E-01	7.95E-02	3.41E-01	6.36E-02
**total EC**	µg/µg PM	6.62E-03	3.79E-02	1.78E-02	6.97E-02	1.38E-02	2.91E-02	8.71E-03	7.44E-02	1.61E-02
**K^+**	µg/µg PM	4.88E-04	1.41E-02	6.71E-03	3.07E-02	5.46E-03	9.88E-03	3.80E-03	2.62E-02	5.04E-03
**Na^+**	µg/µg PM	0.96	5.54E-03	9.73E-04	1.21E-02	2.78E-03	6.33E-03	8.65E-04	3.31E-02	5.99E-03
**Ca^{2+}**	µg/µg PM	0.79	6.50E-03	1.24E-03	2.56E-02	6.11E-03	6.01E-03	9.87E-04	2.11E-02	5.15E-03
**NH_4^+**	µg/µg PM	0.83	8.62E-02	4.15E-02	1.41E-01	2.02E-02	1.03E-01	2.03E-03	3.88E-01	7.71E-02
**NO_3^-**	µg/µg PM	7.80E-05	1.22E-01	6.62E-02	1.79E-01	3.02E-02	1.93E-01	7.52E-02	3.86E-01	8.24E-02
**SO_4^{2-}**	µg/µg PM	4.57E-12	9.19E-02	4.05E-02	2.51E-01	4.05E-02	1.89E-01	1.04E-01	5.18E-01	7.64E-02
**Cl^-**	µg/µg PM	4.03E-09	4.78E-02	1.64E-02	9.79E-02	1.96E-02	1.53E-02	4.04E-03	8.88E-02	1.60E-02
**Al**	µg/µg PM	0.60	6.93E-03	9.37E-04	2.06E-02	4.55E-03	6.81E-03	0	2.49E-02	5.65E-03
**Ti**	µg/µg PM	8.82E-03	9.29E-04	3.05E-05	8.24E-03	1.92E-03	6.65E-04	1.81E-04	1.75E-03	3.85E-04
**V**	µg/µg PM	3.58E-03	5.75E-05	8.83E-07	4.42E-04	1.08E-04	1.10E-04	3.36E-06	2.85E-04	9.08E-05
**Cr**	µg/µg PM	9.58E-06	4.08E-04	5.60E-05	1.64E-03	3.77E-04	1.48E-04	0	4.50E-04	1.03E-04
**Mn**	µg/µg PM	2.24E-03	5.84E-04	1.83E-04	2.78E-03	4.61E-04	8.04E-04	2.78E-04	2.27E-03	4.27E-04
**Fe**	µg/µg PM	9.16E-04	1.00E-02	2.64E-03	3.73E-02	6.84E-03	1.51E-02	5.88E-03	3.21E-02	7.04E-03
**Co**	µg/µg PM	0.49	8.79E-06	1.90E-06	5.07E-05	1.13E-05	2.38E-05	1.65E-06	4.84E-05	2.35E-05
**Ni**	µg/µg PM	0.05	8.41E-05	1.62E-05	5.25E-04	1.10E-04	6.05E-05	0	3.96E-04	9.44E-05
**Cu**	µg/µg PM	0.15	2.54E-04	1.75E-05	2.17E-03	3.65E-04	1.69E-04	1.35E-05	5.40E-04	1.30E-04
**Zn**	µg/µg PM	0.44	4.39E-03	1.17E-03	2.15E-02	3.92E-03	3.31E-03	1.53E-03	7.57E-03	1.41E-03
**Cd**	µg/µg PM	0.82	2.75E-04	1.25E-04	7.48E-04	1.49E-04	3.04E-04	4.04E-05	7.12E-04	2.90E-04

**Sb**	µg/µg PM	7.31E-09	9.28E-05	1.72E-05	2.04E-04	4.22E-05	1.86E-03	2.97E-04	3.94E-03	1.42E-03
**Ba**	µg/µg PM	1.31E-03	1.54E-04	2.43E-05	6.83E-04	1.75E-04	4.26E-04	1.48E-05	1.52E-03	3.99E-04
**Pb**	µg/µg PM	0.94	9.73E-04	6.40E-05	2.91E-03	6.35E-04	9.15E-04	2.82E-04	1.85E-03	4.07E-04
**galactosan**	µg/µg PM	5.24E-16	5.43E-04	7.35E-05	1.63E-03	3.72E-04	4.98E-05	1.68E-05	1.44E-04	2.74E-05
**mannosan**	µg/µg PM	3.41E-15	7.10E-04	1.00E-04	1.76E-03	4.27E-04	7.86E-05	1.47E-05	2.98E-04	5.84E-05
**levoglucosan**	µg/µg PM	3.65E-14	6.35E-03	9.56E-04	1.91E-02	4.04E-03	8.22E-04	2.14E-04	3.72E-03	6.88E-04
**ORG**	µg/µg PM	0.03	3.97E-01	3.01E-01	4.86E-01	5.84E-02	3.57E-01	1.99E-01	6.87E-01	1.01E-01
**MOOOA**	µg/µg PM	0.74	7.11E-02	1.66E-02	1.19E-01	2.84E-02	6.83E-02	1.61E-02	1.04E-01	2.71E-02
**LOOOA**	µg/µg PM	0.14	4.71E-02	7.00E-03	1.18E-01	3.16E-02	6.64E-02	7.38E-03	1.69E-01	4.52E-02
**O_3**	ppb	5.72E-16	8.47	2.36	25.81	5.87	53.92	8.40	98.47	21.10
**CO**	ppb	1.35E-11	1473.47	476.13	2820.28	671.32	527.69	282.16	1156.72	166.21
**NO**	ppb	1.12E-10	43.90	1.16	122.20	29.79	4.89	0.40	19.75	5.23
**NO_2**	ppb	4.73E-07	37.35	9.95	67.12	13.91	21.42	12.65	37.31	6.11
**NO_y**	ppb	6.66E-07	88.90	12.54	184.47	46.57	35.04	17.75	66.83	12.43
**SO_2**	ppb	1.95E-05	5.68	0.88	12.62	3.40	2.37	0.05	8.14	1.89
**RH8**	%	0.69	49.98	17.16	94.71	17.83	49.98	28.28	99.70	19.29
**RH120**	%	0.32	48.24	15.49	97.45	19.33	44.68	18.73	99.55	20.63
**RH240**	%	0.46	48.72	15.06	99.36	20.53	45.49	17.03	98.55	19.99
**T8**	°C	4.30E-18	5.33	-2.23	10.95	3.25	26.52	18.22	33.45	3.81
**T120**	°C	4.30E-18	4.36	-3.80	9.52	3.32	24.94	17.11	31.82	3.78
**T240**	°C	4.30E-18	3.62	-5.12	8.33	3.41	23.86	16.46	30.98	3.81
**methanol**	ppb	0.02	20.30	1.59	53.22	14.42	27.64	11.16	43.53	8.23
**acetonitrile**	ppb	0.59	0.45	0.02	1.18	0.36	0.93	-0.03	3.61	1.10
**acetaldehyde**	ppb	1.00	4.51	0.88	10.11	2.89	4.58	1.09	11.87	2.99
**acrolein**	ppb	2.12E-03	0.42	0.04	0.95	0.28	0.18	-0.02	0.59	0.11
**acetone**	ppb	3.42E-03	2.65	0.46	6.14	1.65	3.82	1.49	6.26	1.17
**isoprene**	ppb	1.47E-02	1.09	0.02	2.42	0.76	0.51	0.07	1.32	0.26
**methyl vinyl ketone /methacrolein**	ppb	0.36	0.82	0	2.29	0.71	0.56	-0.04	1.77	0.42
**methyl ethyl ketone**	ppb	0.37	0.22	0.04	0.49	0.14	0.35	-0.13	1.07	0.33
**benzene**	ppb	1.28E-06	1.90	0.16	4.90	1.41	0.35	0.05	1.01	0.20
**toluene**	ppb	1.21E-03	1.85	0.04	4.72	1.46	0.44	0.15	0.88	0.19
**C2-benzenes**	ppb	1.69E-03	1.98	0.13	5.50	1.58	0.58	0.12	1.23	0.25
**C3-benzenes**	ppb	6.05E-03	0.48	0.01	1.17	0.37	0.14	0.01	0.31	0.07
**J O^1D**	s^{-1}	1.47E-11	7.15E-07	-1.40E-08	3.32E-06	5.88E-07	6.61E-06	1.17E-06	9.53E-06	2.21E-06
**J NO_2**	s^{-1}	6.44E-10	7.84E-04	4.23E-05	2.22E-03	4.41E-04	2.78E-03	5.28E-04	3.73E-03	8.53E-04
**naphthalene**	µg/µg PM	1.37E-04	4.67E-06	1.43E-06	1.09E-05	2.40E-06	1.97E-06	2.61E-07	3.87E-06	1.36E-06
**acenaphthylne**	µg/µg PM	1.89E-06	9.18E-06	1.22E-06	2.07E-05	5.63E-06	9.98E-07	2.01E-07	2.54E-06	6.33E-07
**acenaphthene**	µg/µg PM	2.04E-03	1.36E-06	2.67E-07	3.71E-06	9.97E-07	4.78E-07	1.18E-07	1.99E-06	5.13E-07
**fluorene**	µg/µg PM	9.63E-08	1.04E-05	1.93E-06	2.28E-05	6.37E-06	1.19E-06	2.21E-07	2.18E-06	5.31E-07
**phenanthrene**	µg/µg PM	1.44E-09	1.52E-04	3.22E-05	3.54E-04	9.69E-05	7.68E-06	1.67E-06	1.53E-05	4.09E-06
**fluoranthene**	µg/µg PM	1.44E-09	2.03E-04	4.79E-05	4.54E-04	1.32E-04	2.14E-05	9.84E-06	3.98E-05	9.10E-06

**pyrene**	µg/µg PM	1.01E-08	1.75E-04	3.96E-05	3.93E-04	1.14E-04	2.55E-05	1.27E-05	5.46E-05	1.15E-05
**benzo(a)-anthracene**	µg/µg PM	1.44E-09	2.04E-04	3.84E-05	4.53E-04	1.32E-04	1.39E-05	5.10E-06	2.70E-05	6.37E-06
**chrysene**	µg/µg PM	1.44E-09	2.04E-04	5.63E-05	3.81E-04	1.12E-04	2.26E-05	1.31E-05	4.12E-05	8.41E-06
**benzo(b)-fluoranthene**	µg/µg PM	1.44E-09	1.97E-04	5.57E-05	3.88E-04	1.09E-04	3.22E-05	1.84E-05	5.46E-05	1.13E-05
**benzo(k)-fluoranthene**	µg/µg PM	4.93E-06	9.04E-05	2.19E-05	2.12E-04	5.54E-05	2.56E-05	1.26E-05	4.83E-05	9.88E-06
**benzo(a)-pyrene**	µg/µg PM	2.80E-07	2.95E-04	3.20E-05	7.07E-04	1.96E-04	2.68E-05	1.38E-05	4.45E-05	1.00E-05
**indeno (1,2,3-cd)pyrene**	µg/µg PM	3.84E-06	1.38E-04	3.07E-05	2.82E-04	7.67E-05	3.16E-05	1.63E-05	5.64E-05	1.18E-05
**dibenzo-(a,h)-anthracene**	µg/µg PM	1.44E-09	5.27E-05	1.02E-05	1.25E-04	3.39E-05	4.59E-06	1.98E-06	9.21E-06	2.17E-06
**benzo(ghi)-perylene**	µg/µg PM	5.36E-07	1.40E-04	3.23E-05	2.79E-04	7.94E-05	2.72E-05	7.41E-06	5.04E-05	1.24E-05
**C24**	µg/µg PM	4.50E-18	9.66E-04	1.72E-04	4.39E-03	9.68E-04	4.97E-05	1.13E-05	1.92E-04	4.50E-05
**C25**	µg/µg PM	3.95E-15	9.98E-04	1.70E-04	4.55E-03	9.85E-04	1.01E-04	1.17E-05	3.41E-04	9.03E-05
**C26**	µg/µg PM	7.54E-17	5.93E-04	8.88E-05	2.65E-03	5.71E-04	5.28E-05	2.21E-06	2.06E-04	4.05E-05
**C27**	µg/µg PM	6.00E-09	5.47E-04	8.31E-05	2.23E-03	4.73E-04	1.40E-04	4.90E-06	3.92E-04	9.00E-05
**C28**	µg/µg PM	3.52E-13	2.46E-04	3.56E-05	8.79E-04	1.97E-04	4.29E-05	1.38E-06	1.31E-04	2.59E-05
**C29**	µg/µg PM	2.61E-08	4.98E-04	6.84E-05	1.88E-03	3.83E-04	1.55E-04	4.38E-06	3.94E-04	8.30E-05
**C30**	µg/µg PM	5.94E-10	1.38E-04	1.98E-05	4.28E-04	1.14E-04	3.27E-05	1.33E-06	9.30E-05	1.99E-05
**C31**	µg/µg PM	3.09E-02	2.08E-04	3.15E-05	7.60E-04	1.62E-04	1.28E-04	2.47E-06	3.46E-04	8.08E-05
**C32**	µg/µg PM	1.62E-07	7.79E-05	3.47E-06	2.79E-04	5.51E-05	2.73E-05	1.23E-06	8.69E-05	1.96E-05
**C33**	µg/µg PM	1.18E-02	6.68E-05	7.91E-06	2.77E-04	7.01E-05	2.83E-05	3.15E-07	8.92E-05	2.12E-05
**C34**	µg/µg PM	3.38E-10	4.33E-05	3.32E-06	3.36E-04	5.95E-05	3.74E-06	3.13E-07	3.13E-05	5.59E-06
**OH**	ppt	8.70E-12	9.32E-02	5.98E-02	1.57E-01	3.05E-02	2.05E-04	5.29E-05	4.21E-04	8.39E-05
**HO_2**	ppt	6.64E-07	9.68E-01	3.36E-01	2.44	7.40E-01	5.15E-03	3.73E-04	1.17E-02	3.05E-03
**RO_2**	ppt	1.81E-10	1.77	6.44E-01	3.86	9.04E-01	3.11E-02	2.92E-03	1.00E-01	2.66E-02
**palmitic acid**	µg/µg PM	1.34E-10	8.83E-03	2.28E-04	1.13E-01	2.02E-02	8.20E-04	1.45E-04	2.18E-03	4.84E-04
**stearic acid**	µg/µg PM	2.21E-09	5.72E-03	1.16E-04	8.90E-02	1.60E-02	4.98E-04	9.01E-05	1.14E-03	2.68E-04
**cholesterol**	µg/µg PM	2.20E-01	3.11E-05	1.12E-06	2.34E-04	4.70E-05	1.41E-05	3.49E-06	3.36E-05	7.84E-06
**17a(H)-22,29,30-trisnorhopane (C27a)**	µg/µg PM	4.42E-17	4.03E-05	8.58E-06	1.50E-04	3.25E-05	1.86E-06	1.04E-07	1.14E-05	2.05E-06
**17b(H),21a(H)-norhopane (C30ba)**	µg/µg PM	8.24E-14	4.55E-05	8.98E-06	1.78E-04	3.75E-05	5.77E-06	2.08E-07	3.01E-05	5.93E-06

Table S4 B. Summary statistics for all volume-normalised measurements. SOA tracer data is not presented as it will be used for future publications.

feature	units	seasonal MW p-value	winter mean	winter min	winter max	winter SD	summer mean	summer min	summer max	summer SD
**EPR**	counts m^{-3}	4.48E-07	2.46 E+06	2.64 E+05	5.54 E+06	1.64 E+06	6.43 E+05	1.37 E+05	2.18 E+06	5.18 E+05
**AA**	[DHA] m^{-3}	1.12E-13	32.39	7.44	57.78	14.78	8.57	4.94	13.04	2.29
**DTT**	nmol min^{-1} m^{-3}	7.88E-07	2.94	0.59	6.68	1.85	0.90	0.38	2.22	0.40
**DCFH**	[H_2O_2] m^{-3}	1.58E-05	0.71	2.47E-03	1.99	0.53	0.18	-6.46E-03	0.46	0.11
**total OC**	µg/m^3	1.23E-07	20.18	3.95	48.81	12.27	6.51	1.82	12.71	2.33
**total EC**	µg/m^3	2.34E-07	3.23	0.28	6.58	1.93	0.92	0.24	1.66	0.36
**K^+**	µg/m^3	3.71E-05	1.32	0.15	3.80	1.04	0.38	0.11	2.05	0.36
**Na^+**	µg/m^3	7.28E-04	0.42	0.09	0.93	0.25	0.21	0.03	0.73	0.16
**Ca^{2+}**	µg/m^3	5.10E-06	0.34	0.16	0.62	0.12	0.18	0.03	0.36	0.10
**NH_4^+**	µg/m^3	7.04E-04	8.09	0.50	22.62	5.67	3.70	0.08	14.83	3.15
**NO_3^-**	µg/m^3	0.05	12.38	0.87	34.63	9.54	7.22	1.53	26.12	5.00
**SO_4^{2-}**	µg/m^3	0.95	8.51	1.27	24.21	7.16	6.92	1.96	19.48	4.01
**Cl^-**	µg/m^3	6.50E-09	3.70	0	8.73	2.34	0.48	0.12	1.96	0.42
**Al**	µg/m^3	6.68E-04	0.59	0.05	1.64	0.44	0.23	0	0.58	0.15
**Ti**	µg/m^3	0.09	0.04	2.27E-03	0.12	0.03	0.02	5.14E-03	0.04	0.01
**V**	µg/m^3	0.13	2.27E-03	3.23E-05	6.88E-03	1.93E-03	5.05E-03	1.10E-04	0.02	6.37E-03
**Cr**	µg/m^3	6.84E-11	0.02	4.78E-03	0.07	0.02	4.81E-03	9.50E-04	9.96E-03	2.50E-03
**Mn**	µg/m^3	0.09	0.04	9.05E-03	0.11	0.03	0.03	4.96E-03	0.08	0.01
**Fe**	µg/m^3	0.16	0.70	0.19	1.87	0.44	0.49	0.15	1.15	0.20
**Co**	µg/m^3	0.82	4.69E-04	1.96E-04	1.24E-03	2.56E-04	9.37E-04	7.00E-05	2.25E-03	1.16E-03
**Ni**	µg/m^3	2.57E-04	4.63E-03	1.38E-03	1.42E-02	3.05E-03	1.99E-03	1.40E-04	6.97E-03	2.03E-03
**Cu**	µg/m^3	2.08E-04	1.77E-02	1.49E-03	0.05	1.37E-02	6.23E-03	3.20E-04	0.02	5.13E-03
**Zn**	µg/m^3	6.57E-06	0.30	0.07	0.70	0.20	0.12	0.03	0.31	0.06
**Cd**	µg/m^3	0.05	0.02	3.55E-03	0.06	0.02	9.29E-03	1.41E-03	0.02	7.44E-03
**Sb**	µg/m^3	8.49E-04	9.04E-03	1.40E-04	0.02	6.69E-03	0.05	7.04E-03	0.12	0.04
**Ba**	µg/m^3	0.31	9.49E-03	7.19E-04	0.03	8.01E-03	1.20E-02	3.50E-04	0.04	9.63E-03
**Pb**	µg/m^3	9.41E-05	0.09	1.07E-02	0.31	0.07	0.03	4.82E-03	0.14	0.03
**galactosan**	µg/m^3	4.50E-18	0.04	3.14E-03	0.10	0.03	1.46E-03	3.46E-05	4.04E-03	8.39E-04
**mannosan**	µg/m^3	2.14E-17	0.05	3.83E-03	0.11	0.04	2.38E-03	3.48E-04	1.33E-02	2.25E-03
**levoglucosan**	µg/m^3	7.70E-16	0.48	0.03	1.00	0.31	0.03	2.72E-03	0.17	0.03
**ORG**	µg/m^3	8.39E-06	35.32	4.13	94.85	23.93	10.01	4.19	21.83	3.93
**MOOOA**	µg/m^3	2.71E-04	17.62	0.91	51.19	13.97	4.21	0.52	15.11	3.32
**LOOOA**	µg/m^3	6.43E-03	14.65	1.52	48.43	13.90	4.64	1.14	10.44	2.64
**O_3**	ppb	9.56E-04	9.00	0.71	27.00	7.23	2.79	0.49	7.96	1.68
**CO**	ppb	5.40E-03	8.03	0.26	22.95	7.12	1.89	0.60	5.20	1.02
**NO**	ppb	0.11	5.09	0.11	18.32	5.16	2.24	0.14	11.27	2.30
**NO_2**	ppb	5.72E-16	8.47	2.36	25.81	5.87	53.92	8.40	98.47	21.10
**NO_y**	ppb	1.35E-11	1.47 E+03	4.76 E+02	2.82 E+03	6.71 E+02	5.28 E+02	2.82 E+02	1.16 E+03	1.66 E+02
**SO_2**	ppb	1.12E-10	43.90	1.16	122.20	29.79	4.89	0.40	19.75	5.23
**RH8**	%	4.73E-07	37.35	9.95	67.12	13.91	21.42	12.65	37.31	6.11
**RH120**	%	6.66E-07	88.90	12.54	184.47	46.57	35.04	17.75	66.83	12.43
**RH240**	%	1.95E-05	5.68	0.88	12.62	3.40	2.37	0.05	8.14	1.89
**T8**	°C	0.69	49.98	17.16	94.71	17.83	49.98	28.28	99.70	19.29
**T120**	°C	0.32	48.24	15.49	97.45	19.33	44.68	18.73	99.55	20.63
**T240**	°C	0.46	48.72	15.06	99.36	20.53	45.49	17.03	98.55	19.99
**methanol**	ppb	4.30E-18	5.33	-2.23	10.95	3.25	26.52	18.22	33.45	3.81
**acetonitrile**	ppb	4.30E-18	4.36	-3.80	9.52	3.32	24.94	17.11	31.82	3.78
**acetaldehyde**	ppb	4.30E-18	3.62	-5.12	8.33	3.41	23.86	16.46	30.98	3.81

**acrolein**	ppb	0.02	20.30	1.59	53.22	14.42	27.64	11.16	43.53	8.23
**acetone**	ppb	0.59	0.45	0.02	1.18	0.36	0.93	-0.03	3.61	1.10
**isoprene**	ppb	1.00	4.51	0.88	10.11	2.89	4.58	1.09	11.87	2.99
**methyl vinyl ketone /methacrolein**	ppb	2.12E-03	0.42	0.04	0.95	0.28	0.18	-0.02	0.59	0.11
**methyl ethyl ketone**	ppb	3.42E-03	2.65	0.46	6.14	1.65	3.82	1.49	6.26	1.17
**benzene**	ppb	0.01	1.09	0.02	2.42	0.76	0.51	0.07	1.32	0.26
**toluene**	ppb	0.36	0.82	0.00	2.29	0.71	0.56	-0.04	1.77	0.42
**C2-benzenes**	ppb	0.37	0.22	0.04	0.49	0.14	0.35	-0.13	1.07	0.33
**C3-benzenes**	ppb	1.28E-06	1.90	0.16	4.90	1.41	0.35	0.05	1.01	0.20
**J O^1D**	s^{-1}	1.21E-03	1.85	0.04	4.72	1.46	0.44	0.15	0.88	0.19
**J NO_2**	s^{-1}	1.69E-03	1.98	0.13	5.50	1.58	0.58	0.12	1.23	0.25
**naphthalene**	µg/m³	6.05E-03	0.48	0.01	1.17	0.37	0.14	0.01	0.31	0.07
**acenaphthylne**	µg/m³	1.47E-11	7.15E-07	-1.39<br>E-08	3.32E-06	5.88E-07	6.61E-06	1.17E-06	9.53E-06	2.21E-06
**acenaphthene**	µg/m³	1.49E-12	7.84E-04	4.23E-05	2.22E-03	4.41E-04	2.78E-03	5.28E-04	3.73E-03	8.53E-04
**fluorene**	µg/m³	1.10E-07	2.71E-04	7.83E-05	4.88E-04	1.41E-04	5.69E-05	1.21E-05	1.61E-04	3.91E-05
**phenanthrene**	µg/m³	9.97E-08	4.84E-04	1.63E-04	9.60E-04	2.93E-04	2.63E-05	1.05E-05	6.39E-05	1.32E-05
**fluoranthene**	µg/m³	9.32E-06	7.15E-05	1.86E-05	1.70E-04	4.38E-05	1.28E-05	4.28E-06	3.26E-05	9.32E-06
**pyrene**	µg/m³	1.38E-08	5.55E-04	2.30E-04	1.09E-03	3.26E-04	3.34E-05	1.24E-05	7.07E-05	1.49E-05
**benzo(a)-anthracene**	µg/m³	1.44E-09	7.83E-03	3.15E-03	1.45E-02	3.94E-03	2.29E-04	4.26E-05	4.47E-04	9.89E-05
**chrysene**	µg/m³	1.44E-09	9.78E-03	4.71E-03	1.97E-02	3.65E-03	6.90E-04	2.92E-04	1.67E-03	3.78E-04
**benzo(b)-fluoranthene**	µg/m³	1.44E-09	8.34E-03	4.08E-03	1.69E-02	3.16E-03	7.72E-04	3.26E-04	1.38E-03	2.84E-04
**benzo(k)-fluoranthene**	µg/m³	1.44E-09	1.00E-02	3.89E-03	1.86E-02	4.66E-03	3.98E-04	2.63E-04	5.09E-04	6.65E-05
**benzo(a)-pyrene**	µg/m³	1.44E-09	1.07E-02	3.70E-03	1.99E-02	4.51E-03	6.90E-04	3.81E-04	1.16E-03	2.09E-04
**indeno (1,2,3-cd)pyrene**	µg/m³	1.44E-09	1.03E-02	3.62E-03	1.77E-02	4.25E-03	9.92E-04	4.93E-04	1.73E-03	3.03E-04
**dibenzo-(a,h)-anthracene**	µg/m³	1.44E-09	4.48E-03	2.20E-03	8.41E-03	1.65E-03	7.67E-04	4.69E-04	1.14E-03	1.81E-04
**benzo(ghi)-perylene**	µg/m³	1.44E-09	1.50E-02	4.47E-03	2.98E-02	8.91E-03	8.01E-04	4.95E-04	1.18E-03	1.71E-04
**C24**	µg/m³	1.44E-09	7.82E-03	2.20E-03	1.58E-02	4.57E-03	9.57E-04	5.51E-04	1.50E-03	2.61E-04
**C25**	µg/m³	1.44E-09	2.70E-03	1.04E-03	5.23E-03	1.39E-03	1.35E-04	9.07E-05	2.31E-04	4.11E-05
**C26**	µg/m³	1.44E-09	7.48E-03	2.61E-03	1.34E-02	3.75E-03	7.82E-04	3.94E-04	1.21E-03	1.84E-04
**C27**	µg/m³	1.13E-18	0.07	6.07E-03	0.29	0.06	1.34E-03	5.60E-04	3.38E-03	6.98E-04
**C28**	µg/m³	1.13E-18	0.07	6.26E-03	0.29	0.06	2.80E-03	3.88E-04	6.24E-03	1.49E-03
**C29**	µg/m³	1.13E-18	0.04	4.35E-03	0.16	0.04	1.55E-03	7.29E-05	3.62E-03	6.50E-04
**C30**	µg/m³	2.35E-15	0.04	4.47E-03	0.13	0.03	4.32E-03	1.62E-04	1.04E-02	1.90E-03
**C31**	µg/m³	1.13E-18	0.02	2.33E-03	0.06	1.31E-02	1.35E-03	4.57E-05	2.32E-03	5.50E-04
**C32**	µg/m³	1.65E-13	0.04	5.32E-03	0.10	0.03	5.19E-03	1.45E-04	0.02	3.37E-03
**C33**	µg/m³	3.05E-15	8.89E-03	1.21E-03	0.02	5.66E-03	1.03E-03	4.40E-05	1.86E-03	4.20E-04
**C34**	µg/m³	1.23E-07	1.38E-02	1.71E-03	0.03	8.29E-03	4.25E-03	8.17E-05	0.02	3.10E-03
**OH**	ppt	2.03E-12	5.38E-03	6.21E-04	0.02	3.68E-03	8.38E-04	4.05E-05	1.53E-03	3.84E-04
**HO_2**	ppt	1.14E-08	4.05E-03	3.38E-04	0.02	3.63E-03	9.29E-04	1.04E-05	3.16E-03	6.37E-04
**RO_2**	ppt	5.72E-16	2.79	0.14	8.57	2.47	0.11	0.01	0.72	0.13
**palmitic acid**	µg/m³	8.70E-12	0.09	0.06	0.16	0.03	2.05E-04	5.29E-05	4.21E-04	8.39E-05
**stearic acid**	µg/m³	6.64E-07	0.97	0.34	2.44	0.74	5.15E-03	3.73E-04	1.17E-02	3.05E-03
**cholesterol**	µg/m³	1.81E-10	1.77E-03	6.44E-04	3.86E-03	9.04E-04	3.11E-05	2.92E-06	1.00E-04	2.66E-05
**17a(H)-22,29,30-trisnorhopane (C27a)**	µg/m³	1.56E-16	0.34	0.03	1.17	0.26	0.03	0.01	0.07	0.01
**17b(H),21a(H)-norhopane (C30ba)**	µg/m³	1.18E-15	0.19	0.02	0.92	0.21	0.02	0.01	0.04	0.01

Section S7: Volume-normalised concentration stacked bar plots

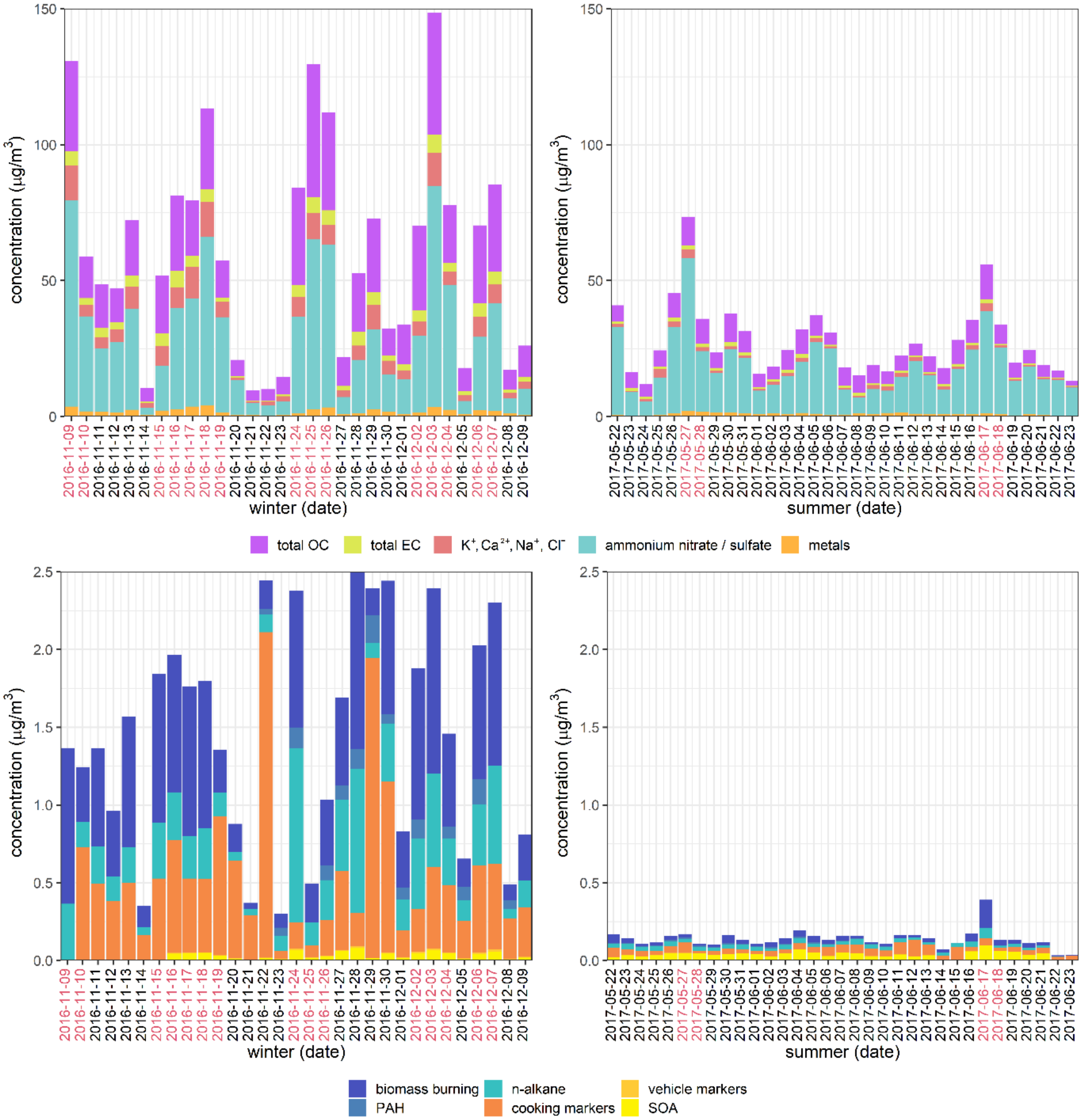

Figure S13. Abbreviations: OC: organic carbon ; EC: elemental carbon; PAH: polycyclic aromatic hydrocarbon; SOA: secondary organic aerosol "Metals" is the summed concentrations of Al, Ti, V, Cr, Mn, Fe, Co, Ni, Cu, Zn, Cd, Sb, Ba, Pb; "biomass burning" is the summed concentrations of palmitic acid, stearic acid and cholesterol; "PAH" is the summed concentrations of naphthalene, acenaphthylne, acenaphthene, fluorene, phenanthrene, fluoranthene, pyrene, benzo(a)anthracene, chrysene, benzo(b)fluoranthene, benzo(k)fluoranthene, benzo(a)pyrene, indeno(1,2,3-cd)pyrene, dibenzo(a,h)anthracene and benzo(ghi)perylene; "n-alkane" is the summed concentrations of C24, C25, C26, C27, C28, C29, C30, C31, C32, C33, C34; "cooking markers" is the summed concentrations of palmitic acid, stearic acid, cholesterol; "vehicle markers" is the summed concentrations of 17a(H)-22,29,30-trisnorhopane (C27a) and 17b(H),21a(H)-norhopane (C30ba); "SOA" is the summed concentrations of 2-methylthreitol, 2-methylerythritol, 2-methylglyceric acid, cis-2-methyl-1,3,4-trihydroxy-1-butene, -methyl-2,3,4-trihydroxy-1-butene, trans-2-methyl-1,3,4-trihydroxy-1-butene, C5-alkene triols, 2-methyltetrols, 3-hydroxyglutaric acid, cis-pinonic acid, acid, MBTCA, β-caryophyllinic acid, glutaric acid derivative, 3-acetylpentanedioic acid, 3-acetylhexanedioic acid, 3-isopropylpentanedioic acid and 2,3-dihydroxy-4-oxopentanoic acid. Dates marked in red indicate partial or total day haze events as described in Shi et al. (2019).

Section S8: Assay correlations with individual component measurements

Mass-normalised data

Table S5. Spearman rank correlations for all EPR_m assay responses with all individual measurements. Benjamini-Hochberg adjusted p-values may be identical for different measurements as the correlations and adjustments are based on rank order.

assay	feature	winter R^2	winter p-value	winter BH p-value	summer R^2	summer p-value	summer BH p-value
**EPR**	total OC	0.28	0.13	0.34	-0.10	0.61	0.86
**EPR**	total EC	0.45	1.11E-02	0.11	-0.19	0.30	0.85
**EPR**	K^+	0.33	7.01E-02	0.26	0.32	0.09	0.82
**EPR**	Na^+	0.17	0.37	0.59	0.11	0.58	0.85
**EPR**	Ca^{2+}	0.16	0.39	0.59	0.09	0.63	0.87
**EPR**	NH_4^+	0.22	0.23	0.44	0.30	9.77E-02	0.82
**EPR**	NO_3^-	0.11	0.56	0.71	-0.16	0.39	0.85
**EPR**	SO_4^{2-}	0.02	0.90	0.93	0.43	0.02	0.45
**EPR**	Cl^-	0.21	0.27	0.48	-0.14	0.47	0.85
**EPR**	Al	-0.03	0.85	0.93	-0.23	0.21	0.85
**EPR**	Ti	0.20	0.28	0.48	-0.25	0.18	0.85
**EPR**	V	0.16	0.42	0.61	-0.22	0.45	0.85
**EPR**	Cr	0.06	0.76	0.85	-0.14	0.44	0.85
**EPR**	Mn	0.29	0.11	0.32	-0.19	0.31	0.85
**EPR**	Fe	0.31	9.00E-02	0.28	-0.06	0.74	0.91
**EPR**	Co	0.25	0.17	0.39	below LOD	below LOD	below LOD
**EPR**	Ni	0.33	7.42E-02	0.26	0.16	0.55	0.85
**EPR**	Cu	0.36	4.67E-02	0.22	0.04	0.85	0.92
**EPR**	Zn	0.31	9.14E-02	0.28	0.17	0.37	0.85
**EPR**	Cd	0.38	3.76E-02	0.22	-0.30	0.62	0.87
**EPR**	Sb	0.40	2.46E-02	0.18	0.12	0.78	0.91
**EPR**	Ba	0.13	0.49	0.67	-0.20	0.35	0.85
**EPR**	Pb	0.46	0.01	0.11	0.06	0.77	0.91
**EPR**	galactosan	0.21	0.25	0.45	-0.49	1.02E-02	0.45
**EPR**	mannosan	0.28	0.13	0.34	-0.19	0.34	0.85
**EPR**	levoglucosan	0.25	0.18	0.39	-0.30	0.13	0.84
**EPR**	ORG	0.52	0.01	0.11	0.18	0.38	0.85
**EPR**	MOOOA	-0.28	0.19	0.39	-0.14	0.48	0.85
**EPR**	LOOOA	-0.38	0.07	0.26	0.30	0.13	0.84
**EPR**	O3	0.06	0.73	0.84	0.31	0.09	0.82
**EPR**	CO	-0.16	0.39	0.59	0.10	0.61	0.86
**EPR**	NO	-0.02	0.91	0.93	-0.05	0.81	0.92
**EPR**	NO_2	-0.14	0.44	0.63	0.01	0.97	0.99
**EPR**	NO_y	-0.09	0.63	0.75	-0.06	0.76	0.91
**EPR**	SO_2	-0.22	0.23	0.44	0.22	0.23	0.85
**EPR**	RH8	0.02	0.91	0.93	-0.16	0.42	0.85
**EPR**	RH120	-0.01	0.94	0.95	-0.11	0.56	0.85
**EPR**	RH240	0.00	0.98	0.98	-0.11	0.58	0.85
**EPR**	T8	0.06	0.76	0.85	0.36	0.06	0.82
**EPR**	T120	0.02	0.90	0.93	0.32	0.09	0.82
**EPR**	T240	0.02	0.90	0.93	0.31	9.96E-02	0.82
**EPR**	methanol	-0.54	0.03	0.18	-0.21	0.27	0.85
**EPR**	acetonitrile	-0.41	0.10	0.30	-0.19	0.33	0.85
**EPR**	acetaldehyde	-0.38	0.14	0.34	-0.29	0.13	0.84
**EPR**	acrolein	-0.40	0.13	0.34	0.11	0.55	0.85
**EPR**	acetone	-0.28	0.28	0.48	0.14	0.47	0.85
**EPR**	isoprene	-0.37	0.14	0.36	0.01	0.97	0.99

**EPR**	methyl vinyl ketone /methacrolein	-0.48	4.96E-02	0.22	0.07	0.73	0.91
**EPR**	methyl ethyl ketone	-0.47	6.78E-02	0.26	-0.16	0.40	0.85
**EPR**	benzene	-0.33	0.20	0.40	-0.22	0.26	0.85
**EPR**	toluene	-0.42	8.98E-02	0.28	0.01	0.96	0.99
**EPR**	C2-benzenes	-0.44	8.00E-02	0.28	-0.01	0.96	0.99
**EPR**	C3-benzenes	-0.45	9.24E-02	0.28	0.07	0.72	0.91
**EPR**	J O^1D	0.11	0.57	0.71	0.28	0.13	0.84
**EPR**	J NO_2	0.02	0.90	0.93	0.37	4.33E-02	0.82
**EPR**	naphthalene	0.69	6.54E-03	0.11	-0.27	0.29	0.85
**EPR**	acenaphthylne	0.70	5.21E-03	0.11	0.07	0.83	0.92
**EPR**	acenaphthene	0.61	2.09E-02	0.18	0.22	0.52	0.85
**EPR**	fluorene	0.72	3.78E-03	0.11	-0.17	0.55	0.85
**EPR**	phenanthrene	0.71	4.82E-03	0.11	-0.02	0.93	0.98
**EPR**	fluoranthene	0.56	3.89E-02	0.22	0.25	0.32	0.85
**EPR**	pyrene	0.56	3.53E-02	0.22	0.23	0.36	0.85
**EPR**	benzo(a)anthracene	0.60	2.33E-02	0.18	0.00	0.99	0.99
**EPR**	chrysene	0.54	4.70E-02	0.22	0.14	0.58	0.85
**EPR**	benzo(b)fluoranthene	0.53	4.92E-02	0.22	0.07	0.78	0.91
**EPR**	benzo(k)fluoranthene	0.50	6.66E-02	0.26	0.08	0.75	0.91
**EPR**	benzo(a)pyrene	0.68	7.56E-03	0.11	0.03	0.92	0.98
**EPR**	indeno(1,2,3-cd)pyrene	0.73	2.92E-03	0.11	0.06	0.82	0.92
**EPR**	dibenzo(a,h)-anthracene	0.66	9.98E-03	0.11	0.16	0.53	0.85
**EPR**	benzo(ghi)perylene	0.71	4.45E-03	0.11	0.03	0.91	0.98
**EPR**	C24	0.10	0.60	0.73	-0.10	0.60	0.86
**EPR**	C25	0.08	0.67	0.78	-0.07	0.71	0.91
**EPR**	C26	0.18	0.33	0.54	-0.08	0.67	0.90
**EPR**	C27	0.12	0.52	0.69	0.05	0.79	0.91
**EPR**	C28	0.24	0.18	0.39	-0.11	0.55	0.85
**EPR**	C29	0.26	0.17	0.39	0.11	0.57	0.85
**EPR**	C30	0.25	0.18	0.39	-0.12	0.51	0.85
**EPR**	C31	0.35	0.05	0.23	0.16	0.40	0.85
**EPR**	C32	0.16	0.40	0.60	-0.17	0.37	0.85
**EPR**	C33	0.41	2.33E-02	0.18	0.15	0.41	0.85
**EPR**	C34	0.36	4.65E-02	0.22	-0.10	0.58	0.85
**EPR**	OH	-0.44	0.10	0.30	0.15	0.45	0.85
**EPR**	HO_2	0.19	0.56	0.71	-0.04	0.84	0.92
**EPR**	RO_2	0.20	0.54	0.71	0.45	1.37E-02	0.45
**EPR**	palmitic acid	0.10	0.58	0.72	-0.18	0.34	0.85
**EPR**	stearic acid	0.07	0.72	0.83	-0.22	0.24	0.85
**EPR**	cholesterol	0.27	0.15	0.37	0.01	0.98	0.99
**EPR**	17a(H)-22,29,30-trisnorhopane (C27a)	0.12	0.52	0.69	-0.33	6.85E-02	0.82
**EPR**	17b(H),21a(H)-norhopane (C30ba)	0.09	0.64	0.76	-0.30	9.92E-02	0.82
**EPR**	2-methylthreitol	0.17	0.43	0.62	0.15	0.45	0.85
**EPR**	2-methylerythritol	0.28	0.18	0.39	0.16	0.42	0.85
**EPR**	2-methylglyceric acid	0.21	0.33	0.54	0.27	0.18	0.85
**EPR**	cis-2-methyl-1,3,4-trihydroxy-1-butene	0.28	0.19	0.39	0.06	0.76	0.91
**EPR**	3-methyl-2,3,4-trihydroxy-1-butene	0.27	0.21	0.40	0.18	0.36	0.85
**EPR**	trans-2-methyl-1,3,4-trihydroxy-1-butene	0.25	0.24	0.45	-0.06	0.76	0.91
**EPR**	C5-alkene triols	0.24	0.26	0.46	0.08	0.69	0.91

**EPR**	2-methyltetrols	0.27	0.20	0.40	0.17	0.41	0.85
**EPR**	3-hydroxyglutaric acid	0.15	0.48	0.66	0.04	0.85	0.92
**EPR**	cis-pinonic acid	0.15	0.47	0.65	-0.26	0.18	0.85
**EPR**	pinic acid	0.18	0.40	0.60	-0.23	0.26	0.85
**EPR**	3-methyl-1,2,3-butanetricarboxylic acid	0.19	0.37	0.59	-0.19	0.34	0.85
**EPR**	β-caryophyllinic acid	0.19	0.37	0.59	0.13	0.52	0.85
**EPR**	glutaric acid derivative	0.07	0.77	0.85	0.25	0.21	0.85
**EPR**	3-acetylpentanedioic acid	0.03	0.87	0.93	-0.16	0.43	0.85
**EPR**	3-acetylhexanedioic acid	0.09	0.67	0.78	-0.21	0.29	0.85
**EPR**	3-isopropyl-pentanedioic acid	0.16	0.45	0.64	-0.14	0.48	0.85
**EPR**	2,3-dihydroxy-4-oxopentanoic acid	0.13	0.55	0.71	0.14	0.49	0.85

Table S6. Spearman rank correlations for all AA_m assay responses with all individual measurements.

assay	feature	winter R^2	winter p-value	winter BH p-value	summer R^2	summer p-value	summer BH p-value
**AA**	total OC	0.72	4.59E-06	2.89E-05	0.50	3.16E-03	2.13E-02
**AA**	total EC	0.51	3.58E-03	7.10E-03	0.46	7.72E-03	3.44E-02
**AA**	K^+	0.36	4.69E-02	5.57E-02	-0.05	0.77	0.83
**AA**	Na^+	0.70	9.90E-06	5.88E-05	0.20	0.28	0.48
**AA**	Ca^{2+}	0.78	3.80E-07	4.93E-06	0.37	4.13E-02	0.11
**AA**	NH_4^+	0.04	0.82	0.83	-0.13	0.48	0.62
**AA**	NO_3^-	-0.43	1.59E-02	2.18E-02	-0.26	0.14	0.29
**AA**	SO_4^{2-}	-0.06	0.73	0.75	-0.24	0.18	0.35
**AA**	Cl^-	0.87	9.77E-10	1.05E-07	0.09	0.63	0.75
**AA**	Al	0.19	0.30	0.34	0.06	0.75	0.83
**AA**	Ti	0.34	6.70E-02	7.71E-02	0.27	0.12	0.26
**AA**	V	0.54	2.34E-03	5.12E-03	0.29	0.29	0.48
**AA**	Cr	0.73	3.97E-06	2.88E-05	0.15	0.42	0.61
**AA**	Mn	0.74	1.65E-06	1.47E-05	0.33	5.81E-02	0.14
**AA**	Fe	0.76	5.54E-07	5.93E-06	0.41	1.67E-02	5.76E-02
**AA**	Co	0.83	1.01E-08	5.39E-07	-0.50	0.67	0.78
**AA**	Ni	0.80	8.86E-08	2.53E-06	0.05	0.84	0.87
**AA**	Cu	0.49	5.59E-03	9.20E-03	-0.07	0.73	0.82
**AA**	Zn	0.77	7.02E-07	6.83E-06	-0.19	0.28	0.48
**AA**	Cd	0.50	4.11E-03	7.79E-03	0.40	0.50	0.65
**AA**	Sb	0.10	0.61	0.65	0.03	0.93	0.95
**AA**	Ba	0.40	2.51E-02	3.28E-02	0.16	0.45	0.62
**AA**	Pb	0.56	1.09E-03	2.93E-03	-0.22	0.22	0.39
**AA**	galactosan	0.47	7.04E-03	1.11E-02	0.62	3.05E-04	4.67E-03
**AA**	mannosan	0.53	2.00E-03	4.65E-03	0.56	1.42E-03	1.18E-02
**AA**	levoglucosan	0.51	3.12E-03	6.29E-03	0.47	1.09E-02	4.18E-02
**AA**	ORG	0.84	3.53E-07	4.93E-06	0.68	5.26E-05	1.41E-03
**AA**	MOOOA	-0.71	8.69E-05	3.44E-04	0.59	7.24E-04	8.61E-03
**AA**	LOOOA	-0.44	2.96E-02	3.70E-02	-0.41	2.83E-02	8.18E-02
**AA**	O_3	0.34	6.10E-02	7.10E-02	-0.54	1.05E-03	1.03E-02
**AA**	CO	-0.38	3.66E-02	4.44E-02	-0.11	0.55	0.69
**AA**	NO	-0.50	4.32E-03	7.79E-03	0.31	0.08	0.18
**AA**	NO_2	-0.39	2.98E-02	3.70E-02	0.13	0.47	0.62
**AA**	NO_y	-0.46	8.41E-03	1.27E-02	0.23	0.20	0.38

**AA**	SO_2	-0.39	3.16E-02	3.89E-02	-0.37	3.46E-02	9.49E-02
**AA**	RH8	-0.58	6.57E-04	2.01E-03	-0.07	0.69	0.80
**AA**	RH120	-0.57	8.65E-04	2.50E-03	-0.15	0.42	0.61
**AA**	RH240	-0.52	2.73E-03	5.72E-03	-0.16	0.39	0.58
**AA**	T8	-0.42	1.90E-02	2.51E-02	-0.24	0.20	0.37
**AA**	T120	-0.45	1.12E-02	1.61E-02	-0.18	0.34	0.53
**AA**	T240	-0.46	9.63E-03	1.41E-02	-0.12	0.51	0.65
**AA**	methanol	-0.65	4.99E-03	8.34E-03	-0.05	0.78	0.83
**AA**	acetonitrile	-0.59	1.25E-02	1.76E-02	-0.16	0.38	0.57
**AA**	acetaldehyde	-0.65	4.37E-03	7.79E-03	-0.14	0.45	0.62
**AA**	acrolein	-0.68	3.79E-03	7.38E-03	0.07	0.70	0.80
**AA**	acetone	-0.65	4.57E-03	8.01E-03	-0.32	8.15E-02	0.19
**AA**	isoprene	-0.62	7.61E-03	1.16E-02	0.25	0.18	0.35
**AA**	methyl vinyl ketone /methacrolein	-0.65	4.37E-03	7.79E-03	0.11	0.57	0.70
**AA**	methyl ethyl ketone	0.16	0.56	0.61	-0.14	0.46	0.62
**AA**	benzene	-0.59	1.25E-02	1.76E-02	0.06	0.73	0.82
**AA**	toluene	-0.66	4.17E-03	7.79E-03	-0.08	0.66	0.77
**AA**	C2-benzenes	-0.65	4.78E-03	8.19E-03	-0.09	0.61	0.74
**AA**	C3-benzenes	-0.60	1.81E-02	2.44E-02	-0.01	0.95	0.96
**AA**	J O^1D	-0.16	0.41	0.45	-0.39	2.32E-02	7.09E-02
**AA**	J NO_2	-0.11	0.56	0.61	-0.44	1.02E-02	4.06E-02
**AA**	naphthalene	0.68	7.56E-03	1.16E-02	0.53	2.01E-02	6.53E-02
**AA**	acenaphthylne	0.71	4.82E-03	8.19E-03	0.63	2.20E-02	6.92E-02
**AA**	acenaphthene	0.59	2.74E-02	3.49E-02	0.23	0.47	0.62
**AA**	fluorene	0.67	8.70E-03	1.29E-02	0.38	0.14	0.29
**AA**	phenanthrene	0.74	2.45E-03	5.23E-03	0.38	9.51E-02	0.22
**AA**	fluoranthene	0.96	9.47E-08	2.53E-06	0.06	0.79	0.83
**AA**	pyrene	0.95	2.77E-07	4.93E-06	0.24	0.31	0.50
**AA**	benzo(a)anthracene	0.87	5.68E-05	2.34E-04	0.50	2.40E-02	7.14E-02
**AA**	chrysene	0.92	4.08E-06	2.88E-05	0.45	4.75E-02	0.12
**AA**	benzo(b)fluoranthene	0.88	3.11E-05	1.58E-04	0.48	3.30E-02	9.28E-02
**AA**	benzo(k)fluoranthene	0.95	2.77E-07	4.93E-06	0.53	1.57E-02	5.59E-02
**AA**	benzo(a)pyrene	0.81	4.32E-04	1.45E-03	0.60	5.60E-03	3.00E-02
**AA**	indeno(1,2,3-cd)pyrene	0.62	1.86E-02	2.49E-02	0.55	1.16E-02	4.30E-02
**AA**	dibenzo(a,h)-anthracene	0.69	6.54E-03	1.04E-02	0.56	9.61E-03	3.95E-02
**AA**	benzo(ghi)perylene	0.82	3.31E-04	1.18E-03	0.58	6.74E-03	3.28E-02
**AA**	C24	0.53	1.94E-03	4.62E-03	0.53	1.66E-03	1.27E-02
**AA**	C25	0.51	3.04E-03	6.25E-03	0.50	3.19E-03	2.13E-02
**AA**	C26	0.54	1.55E-03	3.76E-03	0.47	6.24E-03	3.18E-02
**AA**	C27	0.55	1.26E-03	3.29E-03	0.47	5.48E-03	3.00E-02
**AA**	C28	0.53	2.23E-03	4.98E-03	0.49	3.41E-03	2.14E-02
**AA**	C29	0.56	9.99E-04	2.74E-03	0.34	5.12E-02	0.13
**AA**	C30	0.59	4.52E-04	1.47E-03	0.53	1.44E-03	1.18E-02
**AA**	C31	0.57	7.79E-04	2.31E-03	0.45	8.62E-03	3.69E-02
**AA**	C32	0.61	2.59E-04	9.56E-04	0.55	8.57E-04	9.17E-03
**AA**	C33	0.57	9.01E-04	2.54E-03	0.20	0.27	0.47
**AA**	C34	0.55	1.47E-03	3.67E-03	0.46	7.67E-03	3.44E-02
**AA**	OH	-0.14	6.21E-01	6.57E-01	-0.02	0.90	0.93
**AA**	HO_2	0.03	9.31E-01	9.31E-01	-0.10	0.61	0.74
**AA**	RO_2	0.12	7.13E-01	7.41E-01	-0.17	0.36	0.55
**AA**	palmitic acid	0.69	2.23E-05	1.19E-04	0.61	1.56E-04	3.33E-03
**AA**	stearic acid	0.69	2.15E-05	1.19E-04	0.57	6.05E-04	8.09E-03
**AA**	cholesterol	0.59	5.36E-04	1.69E-03	-0.13	0.46	0.62

**AA**	17a(H)-22,29,30-trisnorhopane (C27a)	0.68	3.62E-05	1.76E-04	0.67	1.84E-05	7.84E-04
**AA**	17b(H),21a(H)-norhopane (C30ba)	0.67	4.68E-05	2.18E-04	0.74	9.87E-07	1.06E-04
**AA**	2-methylthreitol	0.73	5.56E-05	2.34E-04	0.20	0.31	0.50
**AA**	2-methylerythritol	0.45	2.59E-02	3.34E-02	0.17	0.37	0.57
**AA**	2-methylglyceric acid	0.60	2.13E-03	4.86E-03	0.23	0.24	0.42
**AA**	cis-2-methyl-1,3,4-trihydroxy-1-butene	0.80	3.35E-06	2.76E-05	0.05	0.78	0.83
**AA**	3-methyl-2,3,4-trihydroxy-1-butene	0.83	4.15E-07	4.93E-06	0.14	0.47	0.62
**AA**	trans-2-methyl-1,3,4-trihydroxy-1-butene	0.66	4.19E-04	1.45E-03	0.44	1.78E-02	5.95E-02
**AA**	C5-alkene triols	0.73	5.39E-05	2.34E-04	0.31	0.10	0.23
**AA**	2-methyltetrols	0.55	5.86E-03	9.51E-03	0.18	0.34	0.53
**AA**	3-hydroxyglutaric acid	0.49	1.44E-02	2.00E-02	-0.01	0.96	0.96
**AA**	cis-pinonic acid	0.79	4.31E-06	2.88E-05	0.70	2.20E-05	7.84E-04
**AA**	pinic acid	0.69	2.20E-04	8.41E-04	0.51	4.36E-03	2.59E-02
**AA**	3-methyl-1,2,3-butanetricarboxylic acid	0.43	3.79E-02	4.55E-02	0.63	2.61E-04	4.65E-03
**AA**	β-caryophyllinic acid	0.06	0.78	0.80	-0.05	0.79	0.83
**AA**	glutaric acid derivative	0.30	0.18	0.21	0.30	0.11	0.24
**AA**	3-acetylpentanedioic acid	0.39	5.62E-02	6.61E-02	0.30	0.11	0.24
**AA**	3-acetylhexanedioic acid	0.08	0.70	0.74	0.35	6.21E-02	0.15
**AA**	3-isopropyl-pentanedioic acid	0.62	1.36E-03	3.47E-03	0.27	0.16	0.32
**AA**	2,3-dihydroxy-4-oxopentanoic acid	0.34	0.10	0.12	0.11	0.57	0.70

Table S7. Spearman rank correlations for all DTT_m assay responses with all individual measurements.

assay	feature	winter R^2	winter p-value	winter BH p-value	summer R^2	summer p-value	summer BH p-value
**DTT**	total OC	0.59	4.33E-04	4.77E-03	0.58	3.87E-04	2.07E-02
**DTT**	total EC	0.32	7.79E-02	0.12	0.52	1.78E-03	2.66E-02
**DTT**	K^+	0.32	7.79E-02	0.12	0.27	0.15	0.29
**DTT**	Na^+	0.72	5.71E-06	6.11E-04	0.41	2.09E-02	8.27E-02
**DTT**	Ca^{2+}	0.69	2.58E-05	9.21E-04	0.46	9.87E-03	5.03E-02
**DTT**	NH_4^+	0.37	4.12E-02	7.60E-02	0.42	1.50E-02	6.44E-02
**DTT**	NO_3^-	-0.05	0.78	0.81	0.10	0.58	0.71
**DTT**	SO_4^{2-}	0.32	7.53E-02	0.12	0.04	0.82	0.87
**DTT**	Cl^-	0.63	2.88E-04	4.77E-03	0.40	2.24E-02	8.33E-02
**DTT**	Al	-0.13	0.47	0.51	-0.04	0.80	0.87
**DTT**	Ti	0.21	0.27	0.32	0.20	0.26	0.44
**DTT**	V	0.38	4.11E-02	7.60E-02	-0.29	0.30	0.46
**DTT**	Cr	0.45	1.17E-02	3.06E-02	0.33	6.42E-02	0.16
**DTT**	Mn	0.66	5.79E-05	1.55E-03	0.24	0.18	0.35
**DTT**	Fe	0.69	1.90E-05	9.21E-04	0.48	4.49E-03	3.43E-02
**DTT**	Co	0.53	1.98E-03	1.08E-02	1.00	0.00	0.00
**DTT**	Ni	0.52	2.60E-03	1.21E-02	0.31	0.22	0.38
**DTT**	Cu	0.27	0.15	0.19	0.07	0.73	0.80
**DTT**	Zn	0.60	4.73E-04	4.77E-03	0.10	0.59	0.71
**DTT**	Cd	0.59	4.79E-04	4.77E-03	0.30	0.62	0.73

**DTT**	Sb	0.06	0.74	0.77	-0.15	0.70	0.78
**DTT**	Ba	0.46	8.55E-03	2.45E-02	0.33	0.11	0.23
**DTT**	Pb	0.59	4.90E-04	4.77E-03	0.15	0.40	0.57
**DTT**	galactosan	0.20	0.28	0.33	0.38	4.49E-02	0.14
**DTT**	mannosan	0.22	0.23	0.29	0.48	8.89E-03	4.85E-02
**DTT**	levoglucosan	0.19	0.31	0.35	0.41	2.93E-02	0.10
**DTT**	ORG	0.40	5.05E-02	8.72E-02	0.20	0.29	0.46
**DTT**	MOOOA	-0.44	3.35E-02	6.89E-02	0.18	0.35	0.50
**DTT**	LOOOA	-0.39	5.74E-02	9.44E-02	-0.37	4.57E-02	0.14
**DTT**	O_3	0.28	0.13	0.18	-0.21	0.24	0.41
**DTT**	CO	-0.45	1.06E-02	2.85E-02	-0.19	0.30	0.46
**DTT**	NO	-0.53	1.94E-03	1.08E-02	0.09	0.63	0.73
**DTT**	NO_2	-0.48	5.97E-03	2.00E-02	-0.09	0.64	0.73
**DTT**	NO_y	-0.49	4.99E-03	1.84E-02	-0.10	0.59	0.71
**DTT**	SO_2	-0.60	3.21E-04	4.77E-03	-0.06	0.73	0.80
**DTT**	RH8	-0.26	0.16	0.21	-0.11	0.56	0.71
**DTT**	RH120	-0.27	0.15	0.19	-0.12	0.54	0.71
**DTT**	RH240	-0.19	0.30	0.34	-0.11	0.55	0.71
**DTT**	T8	-0.26	0.16	0.20	-0.19	0.31	0.46
**DTT**	T120	-0.29	0.12	0.17	-0.19	0.31	0.46
**DTT**	T240	-0.30	9.63E-02	0.14	-0.15	0.44	0.59
**DTT**	methanol	-0.74	6.80E-04	5.60E-03	-0.19	0.30	0.46
**DTT**	acetonitrile	-0.66	3.99E-03	1.60E-02	-0.28	0.13	0.26
**DTT**	acetaldehyde	-0.75	5.99E-04	5.34E-03	-0.10	0.61	0.72
**DTT**	acrolein	-0.71	2.11E-03	1.08E-02	0.01	0.94	0.95
**DTT**	acetone	-0.73	9.80E-04	7.41E-03	-0.04	0.82	0.87
**DTT**	isoprene	-0.69	2.35E-03	1.14E-02	-0.10	0.61	0.72
**DTT**	methyl vinyl ketone /methacrolein	-0.72	1.04E-03	7.41E-03	-0.13	0.48	0.64
**DTT**	methyl ethyl ketone	-0.31	0.25	0.30	-0.15	0.42	0.58
**DTT**	benzene	-0.64	5.45E-03	1.94E-02	-0.20	0.28	0.45
**DTT**	toluene	-0.70	1.91E-03	1.08E-02	-0.09	0.65	0.73
**DTT**	C2-benzenes	-0.71	1.54E-03	1.03E-02	-0.11	0.57	0.71
**DTT**	C3-benzenes	-0.69	4.77E-03	1.82E-02	-0.04	0.85	0.89
**DTT**	J O^1D	0.10	0.59	0.62	-0.24	0.17	0.33
**DTT**	J NO_2	0.02	0.92	0.93	-0.23	0.20	0.37
**DTT**	naphthalene	0.56	3.53E-02	7.00E-02	0.24	0.33	0.49
**DTT**	acenaphthylne	0.72	3.78E-03	1.60E-02	0.37	0.22	0.38
**DTT**	acenaphthene	0.61	2.09E-02	4.85E-02	0.52	8.49E-02	0.19
**DTT**	fluorene	0.82	3.79E-04	4.77E-03	0.54	2.93E-02	0.10
**DTT**	phenanthrene	0.75	2.03E-03	1.08E-02	0.31	0.19	0.35
**DTT**	fluoranthene	0.67	8.70E-03	2.45E-02	0.18	0.43	0.59
**DTT**	pyrene	0.69	6.54E-03	2.12E-02	0.41	7.33E-02	0.18
**DTT**	benzo(a)anthracene	0.61	1.97E-02	4.69E-02	0.60	5.02E-03	3.58E-02
**DTT**	chrysene	0.54	4.70E-02	8.38E-02	0.51	2.26E-02	8.33E-02
**DTT**	benzo(b)fluoranthene	0.60	2.21E-02	5.02E-02	0.55	1.16E-02	5.66E-02
**DTT**	benzo(k)fluoranthene	0.62	1.86E-02	4.52E-02	0.54	1.37E-02	6.37E-02
**DTT**	benzo(a)pyrene	0.58	3.04E-02	6.50E-02	0.63	2.73E-03	2.66E-02
**DTT**	indeno(1,2,3-cd)pyrene	0.63	1.65E-02	4.21E-02	0.63	2.73E-03	2.66E-02
**DTT**	dibenzo(a,h)-anthracene	0.66	1.07E-02	2.85E-02	0.68	1.01E-03	2.63E-02
**DTT**	benzo(ghi)perylene	0.56	3.53E-02	7.00E-02	0.59	6.07E-03	3.82E-02
**DTT**	C24	0.25	0.18	0.23	0.45	7.86E-03	4.67E-02
**DTT**	C25	0.21	0.26	0.31	0.49	3.88E-03	3.22E-02
**DTT**	C26	0.27	0.15	0.19	0.54	1.22E-03	2.63E-02
**DTT**	C27	0.30	0.10	0.15	0.53	1.45E-03	2.63E-02
**DTT**	C28	0.36	4.82E-02	8.45E-02	0.51	2.46E-03	2.66E-02

**DTT**	C29	0.35	5.64E-02	9.44E-02	0.52	2.12E-03	2.66E-02
**DTT**	C30	0.37	3.88E-02	7.42E-02	0.53	1.48E-03	2.63E-02
**DTT**	C31	0.40	2.61E-02	5.78E-02	0.49	3.92E-03	3.22E-02
**DTT**	C32	0.38	3.74E-02	7.27E-02	0.45	9.06E-03	4.85E-02
**DTT**	C33	0.50	3.77E-03	1.60E-02	0.47	5.67E-03	3.79E-02
**DTT**	C34	0.42	1.73E-02	4.31E-02	0.33	5.81E-02	0.16
**DTT**	OH	0.03	0.91	0.93	0.40	3.26E-02	0.11
**DTT**	HO_2	0.52	8.42E-02	0.13	0.02	0.91	0.94
**DTT**	RO_2	0.25	0.44	0.49	0.11	0.57	0.71
**DTT**	palmitic acid	0.48	7.85E-03	2.40E-02	0.34	5.07E-02	0.15
**DTT**	stearic acid	0.47	8.39E-03	2.45E-02	0.33	6.25E-02	0.16
**DTT**	cholesterol	0.30	0.11	0.15	0.34	5.02E-02	0.15
**DTT**	17a(H)-22,29,30-trisnorhopane (C27a)	0.30	0.11	0.16	0.42	1.61E-02	6.64E-02
**DTT**	17b(H),21a(H)-norhopane (C30ba)	0.28	0.14	0.18	0.42	1.44E-02	6.41E-02
**DTT**	2-methylthreitol	0.34	0.11	0.15	0.02	0.90	0.94
**DTT**	2-methylerythritol	0.39	5.74E-02	9.44E-02	-0.02	0.94	0.95
**DTT**	2-methylglyceric acid	0.28	0.19	0.24	0.09	0.64	0.73
**DTT**	cis-2-methyl-1,3,4-trihydroxy-1-butene	0.53	7.47E-03	2.35E-02	0.21	0.27	0.45
**DTT**	3-methyl-2,3,4-trihydroxy-1-butene	0.56	4.03E-03	1.60E-02	0.28	0.14	0.28
**DTT**	trans-2-methyl-1,3,4-trihydroxy-1-butene	0.44	3.20E-02	6.70E-02	0.36	5.46E-02	0.15
**DTT**	C5-alkene triols	0.45	2.65E-02	5.78E-02	0.31	0.11	0.23
**DTT**	2-methyltetrols	0.38	7.07E-02	0.11	0.01	0.97	0.97
**DTT**	3-hydroxyglutaric acid	0.18	0.39	0.44	0.18	0.36	0.51
**DTT**	cis-pinonic acid	0.54	5.95E-03	2.00E-02	0.33	0.08	0.19
**DTT**	pinic acid	0.36	8.80E-02	0.13	0.36	5.49E-02	0.15
**DTT**	3-methyl-1,2,3-butanetricarboxylic acid	0.24	0.26	0.31	0.39	3.45E-02	0.11
**DTT**	β-caryophyllinic acid	-0.12	0.59	0.62	0.33	8.25E-02	0.19
**DTT**	glutaric acid derivative	-0.02	0.92	0.93	0.31	0.11	0.23
**DTT**	3-acetylpentanedioic acid	0.22	0.30	0.35	0.33	8.42E-02	0.19
**DTT**	3-acetylhexanedioic acid	-0.01	0.98	0.98	0.31	0.10	0.23
**DTT**	3-isopropyl-pentanedioic acid	0.42	4.29E-02	7.77E-02	0.21	0.28	0.45
**DTT**	2,3-dihydroxy-4-oxopentanoic acid	0.12	0.57	0.61	0.29	0.13	0.26

Table S8. Spearman rank correlations for all $DCFH_m$ assay responses with all individual measurements.

assay	feature	winter R^2	winter p-value	winter BH p-value	summer R^2	summer p-value	summer BH p-value
**DCFH**	total OC	-0.41	2.25E-02	9.64E-02	-0.25	0.17	0.45
**DCFH**	total EC	-0.47	7.15E-03	5.79E-02	-0.34	5.32E-02	0.38
**DCFH**	K^+	0.38	3.68E-02	0.14	-0.05	0.80	0.90
**DCFH**	Na^+	-0.14	0.46	0.65	-0.03	0.87	0.94
**DCFH**	Ca^{2+}	-0.33	7.62E-02	0.23	-0.24	0.20	0.47
**DCFH**	NH_4^+	0.34	6.17E-02	0.20	-0.21	0.26	0.50
**DCFH**	NO_3^-	0.40	2.54E-02	0.10	0.05	0.79	0.90
**DCFH**	SO_4^{2-}	0.31	9.27E-02	0.26	0.10	0.57	0.79
**DCFH**	Cl^-	-0.38	4.48E-02	0.16	-0.23	0.21	0.48

**DCFH**	Al	-0.24	0.20	0.40	0.15	0.40	0.63
**DCFH**	Ti	0.11	0.58	0.67	-0.13	0.48	0.70
**DCFH**	V	0.14	0.46	0.65	-0.09	0.76	0.89
**DCFH**	Cr	-0.18	0.34	0.57	-0.16	0.37	0.60
**DCFH**	Mn	-0.06	0.75	0.78	-0.16	0.40	0.63
**DCFH**	Fe	-0.21	0.26	0.46	-0.31	8.37E-02	0.38
**DCFH**	Co	-0.22	0.23	0.44	0.50	0.67	0.85
**DCFH**	Ni	-0.20	0.29	0.50	-0.29	0.25	0.50
**DCFH**	Cu	-0.29	0.12	0.30	-0.23	0.27	0.50
**DCFH**	Zn	-0.11	0.56	0.67	0.15	0.40	0.63
**DCFH**	Cd	0.11	0.55	0.67	0.20	0.75	0.89
**DCFH**	Sb	-0.20	0.29	0.50	-0.74	3.66E-02	0.37
**DCFH**	Ba	-0.30	9.87E-02	0.26	-0.24	0.26	0.50
**DCFH**	Pb	0.17	0.36	0.57	0.20	0.28	0.52
**DCFH**	galactosan	-0.13	0.49	0.66	-0.27	0.16	0.45
**DCFH**	mannosan	-0.26	0.15	0.34	-0.25	0.19	0.45
**DCFH**	levoglucosan	-0.33	7.41E-02	0.23	-0.21	0.27	0.50
**DCFH**	ORG	-0.40	5.57E-02	0.19	0.03	0.90	0.94
**DCFH**	MOOOA	0.25	0.24	0.44	0.19	0.33	0.56
**DCFH**	LOOOA	0.63	1.00E-03	2.09E-02	0.44	2.00E-02	0.37
**DCFH**	O_3	-0.17	0.36	0.57	0.09	0.64	0.84
**DCFH**	CO	0.11	0.54	0.67	0.21	0.25	0.50
**DCFH**	NO	0.06	0.73	0.78	-0.17	0.36	0.59
**DCFH**	NO_2	-0.09	0.64	0.71	0.01	0.96	0.97
**DCFH**	NO_y	0.06	0.74	0.78	-0.04	0.83	0.92
**DCFH**	SO_2	0.17	0.35	0.57	0.07	0.70	0.87
**DCFH**	RH8	0.56	9.49E-04	2.09E-02	0.10	0.60	0.81
**DCFH**	RH120	0.55	1.27E-03	2.09E-02	0.17	0.36	0.59
**DCFH**	RH240	0.56	1.03E-03	2.09E-02	0.17	0.36	0.59
**DCFH**	T8	0.14	0.46	0.65	0.06	0.77	0.89
**DCFH**	T120	0.14	0.45	0.65	0.02	0.91	0.95
**DCFH**	T240	0.12	0.52	0.66	-0.02	0.93	0.96
**DCFH**	methanol	0.17	0.51	0.66	0.26	0.17	0.45
**DCFH**	acetonitrile	0.32	0.22	0.43	0.39	0.03	0.37
**DCFH**	acetaldehyde	0.37	0.14	0.33	0.39	0.03	0.37
**DCFH**	acrolein	0.24	0.38	0.58	-0.08	0.69	0.87
**DCFH**	acetone	0.38	0.14	0.32	0.26	0.16	0.45
**DCFH**	isoprene	0.41	9.83E-02	0.26	-0.43	1.92E-02	0.37
**DCFH**	methyl vinyl ketone /methacrolein	0.25	0.34	0.57	-0.41	2.33E-02	0.37
**DCFH**	methyl ethyl ketone	0.10	0.71	0.77	0.30	0.11	0.42
**DCFH**	benzene	0.32	0.21	0.42	0.25	0.19	0.45
**DCFH**	toluene	0.34	0.18	0.39	0.29	0.12	0.43
**DCFH**	C2-benzenes	0.39	0.12	0.31	0.22	0.24	0.50
**DCFH**	C3-benzenes	0.25	0.38	0.58	-0.07	0.72	0.87
**DCFH**	J O^1D	-0.06	0.76	0.78	-0.10	0.60	0.81
**DCFH**	J NO_2	-0.08	0.66	0.73	-0.11	0.54	0.77
**DCFH**	naphthalene	-0.64	1.38E-02	7.76E-02	-0.14	0.59	0.81
**DCFH**	acenaphthylne	-0.67	8.12E-03	5.79E-02	-0.49	0.11	0.41
**DCFH**	acenaphthene	-0.52	5.86E-02	0.20	-0.22	0.50	0.72
**DCFH**	fluorene	-0.65	1.14E-02	7.17E-02	-0.38	0.17	0.45
**DCFH**	phenanthrene	-0.60	2.21E-02	9.64E-02	-0.09	0.73	0.87
**DCFH**	fluoranthene	-0.68	7.56E-03	5.79E-02	0.09	0.72	0.87
**DCFH**	pyrene	-0.71	4.82E-03	5.16E-02	-0.11	0.66	0.85
**DCFH**	benzo(a)anthracene	-0.79	7.95E-04	2.09E-02	-0.44	0.06	0.38
**DCFH**	chrysene	-0.80	6.28E-04	2.09E-02	-0.32	0.18	0.45
**DCFH**	benzo(b)fluoranthene	-0.75	2.03E-03	2.65E-02	-0.31	0.19	0.45

**DCFH**	benzo(k)fluoranthene	-0.70	5.63E-03	5.48E-02	-0.42	7.26E-02	0.38
**DCFH**	benzo(a)pyrene	-0.77	1.37E-03	2.09E-02	-0.43	6.87E-02	0.38
**DCFH**	indeno(1,2,3-cd)pyrene	-0.61	1.97E-02	9.58E-02	-0.41	8.23E-02	0.38
**DCFH**	dibenzo(a,h)-anthracene	-0.67	8.12E-03	5.79E-02	-0.49	3.27E-02	0.37
**DCFH**	benzo(ghi)perylene	-0.75	2.23E-03	2.65E-02	-0.41	7.80E-02	0.38
**DCFH**	C24	-0.42	1.73E-02	8.98E-02	-0.26	0.16	0.45
**DCFH**	C25	-0.45	1.09E-02	7.17E-02	-0.22	0.23	0.49
**DCFH**	C26	-0.38	3.38E-02	0.13	-0.30	9.98E-02	0.40
**DCFH**	C27	-0.42	1.76E-02	8.98E-02	-0.31	8.84E-02	0.38
**DCFH**	C28	-0.30	0.10	0.26	-0.27	0.13	0.45
**DCFH**	C29	-0.28	0.13	0.31	-0.34	5.40E-02	0.38
**DCFH**	C30	-0.26	0.16	0.34	-0.31	8.80E-02	0.38
**DCFH**	C31	-0.32	7.91E-02	0.24	-0.44	1.10E-02	0.37
**DCFH**	C32	-0.38	3.34E-02	0.13	-0.34	5.32E-02	0.38
**DCFH**	C33	-0.15	0.42	0.62	-0.29	0.10	0.40
**DCFH**	C34	-0.22	0.24	0.44	-0.19	0.30	0.53
**DCFH**	OH	-0.24	0.40	0.60	0.15	0.44	0.66
**DCFH**	HO_2	0.18	0.57	0.67	-0.04	0.84	0.92
**DCFH**	RO_2	0.04	0.90	0.90	0.10	0.62	0.82
**DCFH**	palmitic acid	-0.12	0.51	0.66	-0.37	3.84E-02	0.37
**DCFH**	stearic acid	-0.05	0.77	0.78	-0.35	5.21E-02	0.38
**DCFH**	cholesterol	-0.12	0.53	0.66	-0.33	6.74E-02	0.38
**DCFH**	17a(H)-22,29,30-trisnorhopane (C27a)	-0.45	1.29E-02	7.65E-02	-0.26	0.16	0.45
**DCFH**	17b(H),21a(H)-norhopane (C30ba)	-0.42	2.12E-02	9.64E-02	-0.22	0.23	0.49
**DCFH**	2-methylthreitol	-0.25	0.24	0.44	-0.01	0.95	0.97
**DCFH**	2-methylerythritol	-0.10	0.63	0.71	0.00	1.00	1.00
**DCFH**	2-methylglyceric acid	-0.28	0.19	0.40	-0.11	0.57	0.79
**DCFH**	cis-2-methyl-1,3,4-trihydroxy-1-butene	-0.26	0.22	0.43	-0.19	0.32	0.56
**DCFH**	3-methyl-2,3,4-trihydroxy-1-butene	-0.15	0.48	0.66	-0.35	0.07	0.38
**DCFH**	trans-2-methyl-1,3,4-trihydroxy-1-butene	-0.18	0.40	0.60	-0.28	0.15	0.45
**DCFH**	C5-alkene triols	-0.20	0.36	0.57	-0.29	0.13	0.45
**DCFH**	2-methyltetrols	-0.14	0.51	0.66	-0.03	0.88	0.94
**DCFH**	3-hydroxyglutaric acid	0.11	0.59	0.68	-0.05	0.79	0.90
**DCFH**	cis-pinonic acid	-0.35	9.49E-02	0.26	-0.54	0.00	0.32
**DCFH**	pinic acid	-0.30	0.15	0.34	-0.42	0.03	0.37
**DCFH**	3-methyl-1,2,3-butanetricarboxylic acid	0.14	0.51	0.66	-0.26	0.17	0.45
**DCFH**	β-caryophyllinic acid	-0.09	0.67	0.73	0.15	0.44	0.66
**DCFH**	glutaric acid derivative	0.07	0.74	0.78	-0.23	0.24	0.50
**DCFH**	3-acetylpentanedioic acid	0.06	0.77	0.78	0.04	0.85	0.93
**DCFH**	3-acetylhexanedioic acid	-0.11	0.60	0.68	-0.26	0.18	0.45
**DCFH**	3-isopropyl-pentanedioic acid	-0.12	0.57	0.67	-0.03	0.89	0.94
**DCFH**	2,3-dihydroxy-4-oxopentanoic acid	0.13	0.56	0.67	-0.15	0.44	0.66

Volume-normalised data

Table S9. Spearman rank correlations for all EPR_v assay responses with all individual measurements.

assay	feature	winter R^2	winter p-value	winter BH p-value	summer R^2	summer p-value	summer BH p-value
**EPR**	total OC	0.85	3.24E-09	1.15E-07	0.14	0.44	0.91
**EPR**	total EC	0.82	2.31E-08	2.47E-07	0.02	0.91	0.99
**EPR**	K^+	0.80	1.51E-07	1.27E-06	0.20	0.30	0.89
**EPR**	Na^+	0.71	1.27E-05	6.46E-05	0.10	0.59	0.97
**EPR**	Ca^{2+}	0.55	2.08E-03	7.16E-03	0.36	0.05	0.89
**EPR**	NH_4^+	0.86	1.06E-09	1.13E-07	-0.04	0.82	0.99
**EPR**	NO_3^-	0.83	1.00E-08	1.38E-07	-0.12	0.53	0.97
**EPR**	SO_4^{2-}	0.84	6.78E-09	1.38E-07	0.06	0.74	0.98
**EPR**	Cl^-	0.83	1.37E-08	1.63E-07	0.16	0.39	0.91
**EPR**	Al	0.72	7.24E-06	4.08E-05	-0.21	0.24	0.89
**EPR**	Ti	0.37	5.15E-02	8.23E-02	-0.27	0.13	0.89
**EPR**	V	0.23	0.23	0.25	-0.10	0.73	0.98
**EPR**	Cr	0.69	2.73E-05	1.33E-04	-0.06	0.75	0.98
**EPR**	Mn	0.84	8.39E-09	1.38E-07	-0.20	0.27	0.89
**EPR**	Fe	0.80	8.59E-08	8.36E-07	-0.27	0.14	0.89
**EPR**	Co	0.75	1.80E-06	1.20E-05	0.50	0.67	0.98
**EPR**	Ni	0.66	6.91E-05	3.21E-04	0.06	0.82	0.99
**EPR**	Cu	0.75	1.80E-06	1.20E-05	-0.15	0.45	0.91
**EPR**	Zn	0.80	1.54E-07	1.27E-06	-0.10	0.60	0.97
**EPR**	Cd	0.83	1.04E-08	1.38E-07	-0.60	0.28	0.89
**EPR**	Sb	0.85	2.88E-09	1.15E-07	-0.23	0.56	0.97
**EPR**	Ba	0.57	1.08E-03	4.14E-03	-0.33	0.11	0.89
**EPR**	Pb	0.84	4.71E-09	1.26E-07	-0.17	0.36	0.91
**EPR**	galactosan	0.49	6.29E-03	1.87E-02	-0.20	0.26	0.89
**EPR**	mannosan	0.48	6.63E-03	1.92E-02	-0.09	0.62	0.97
**EPR**	levoglucosan	0.52	3.49E-03	1.13E-02	-0.10	0.58	0.97
**EPR**	ORG	0.85	2.70E-07	2.06E-06	-0.21	0.28	0.89
**EPR**	MOOOA	0.79	6.26E-06	3.72E-05	-0.19	0.33	0.89
**EPR**	LOOOA	0.82	2.05E-06	1.29E-05	-0.12	0.56	0.97
**EPR**	O_3	-0.31	9.76E-02	0.13	-0.05	0.78	0.99
**EPR**	CO	0.31	9.76E-02	0.13	-0.07	0.72	0.98
**EPR**	NO	0.52	3.05E-03	1.02E-02	0.21	0.26	0.89
**EPR**	NO_2	0.42	2.11E-02	4.70E-02	0.18	0.32	0.89
**EPR**	NO_y	0.47	8.83E-03	2.49E-02	0.14	0.45	0.91
**EPR**	SO_2	0.45	1.32E-02	3.53E-02	-0.14	0.45	0.91
**EPR**	RH8	0.59	5.36E-04	2.29E-03	-0.35	0.06	0.89
**EPR**	RH120	0.60	5.23E-04	2.29E-03	-0.34	0.07	0.89
**EPR**	RH240	0.55	1.83E-03	6.54E-03	-0.32	0.08	0.89
**EPR**	T8	0.28	0.13	0.17	0.20	0.29	0.89
**EPR**	T120	0.27	0.15	0.19	0.20	0.30	0.89
**EPR**	T240	0.25	0.19	0.22	0.23	0.23	0.89
**EPR**	methanol	0.51	4.56E-02	7.73E-02	0.00	0.99	0.99
**EPR**	acetonitrile	0.47	6.39E-02	9.63E-02	-0.19	0.32	0.89
**EPR**	acetaldehyde	0.53	3.50E-02	6.64E-02	-0.08	0.65	0.98
**EPR**	acrolein	0.48	6.87E-02	0.10	0.15	0.43	0.91
**EPR**	acetone	0.51	4.41E-02	7.62E-02	0.22	0.23	0.89
**EPR**	isoprene	0.51	4.13E-02	7.62E-02	0.04	0.85	0.99
**EPR**	methyl vinyl ketone /methacrolein	0.56	2.35E-02	5.04E-02	0.10	0.61	0.97
**EPR**	methyl ethyl ketone	3.57E-03	0.99	0.99	-0.10	0.59	0.97
**EPR**	benzene	0.51	4.27E-02	7.62E-02	-0.19	0.31	0.89

**EPR**	toluene	0.57	2.02E-02	4.60E-02	-0.08	0.68	0.98
**EPR**	C2-benzenes	0.57	2.02E-02	4.60E-02	-0.17	0.37	0.91
**EPR**	C3-benzenes	0.38	0.17	0.21	-0.15	0.44	0.91
**EPR**	J O^1D	0.30	0.11	0.15	0.23	0.20	0.89
**EPR**	J NO_2	0.30	0.12	0.16	0.29	0.11	0.89
**EPR**	naphthalene	0.64	1.91E-02	4.60E-02	-0.17	0.48	0.95
**EPR**	acenaphthylne	0.37	0.21	0.24	-0.14	0.64	0.98
**EPR**	acenaphthene	0.36	0.23	0.25	0.06	0.86	0.99
**EPR**	fluorene	0.52	7.07E-02	0.10	-0.33	0.21	0.89
**EPR**	phenanthrene	0.50	8.19E-02	0.12	-0.02	0.94	0.99
**EPR**	fluoranthene	0.60	2.87E-02	5.79E-02	0.19	0.42	0.91
**EPR**	pyrene	0.64	1.78E-02	4.53E-02	0.28	0.24	0.89
**EPR**	benzo(a)anthracene	0.51	7.43E-02	0.11	0.32	0.17	0.89
**EPR**	chrysene	0.68	1.03E-02	2.84E-02	0.13	0.59	0.97
**EPR**	benzo(b)fluoranthene	0.71	6.09E-03	1.86E-02	0.01	0.96	0.99
**EPR**	benzo(k)fluoranthene	0.74	4.11E-03	1.29E-02	0.01	0.98	0.99
**EPR**	benzo(a)pyrene	0.40	0.17	0.21	-0.01	0.96	0.99
**EPR**	indeno(1,2,3-cd)pyrene	0.62	2.52E-02	5.28E-02	-0.02	0.94	0.99
**EPR**	dibenzo(a,h)-anthracene	0.45	0.13	0.17	0.21	0.38	0.91
**EPR**	benzo(ghi)perylene	0.57	4.38E-02	7.62E-02	-0.05	0.83	0.99
**EPR**	C24	0.39	3.27E-02	6.36E-02	-0.12	0.52	0.97
**EPR**	C25	0.39	3.54E-02	6.64E-02	0.05	0.80	0.99
**EPR**	C26	0.40	2.75E-02	5.65E-02	-0.03	0.87	0.99
**EPR**	C27	0.37	4.64E-02	7.73E-02	0.27	0.14	0.89
**EPR**	C28	0.42	2.02E-02	4.60E-02	0.08	0.66	0.98
**EPR**	C29	0.43	1.69E-02	4.42E-02	0.27	0.13	0.89
**EPR**	C30	0.40	3.04E-02	6.02E-02	0.03	0.85	0.99
**EPR**	C31	0.42	1.99E-02	4.60E-02	0.27	0.14	0.89
**EPR**	C32	0.42	2.21E-02	4.84E-02	0.01	0.94	0.99
**EPR**	C33	0.35	5.90E-02	9.15E-02	0.20	0.28	0.89
**EPR**	C34	0.36	5.24E-02	8.25E-02	0.01	0.95	0.99
**EPR**	OH	-0.12	0.69	0.72	0.19	0.32	0.89
**EPR**	HO2	-0.09	0.79	0.81	-0.17	0.37	0.91
**EPR**	RO2	-0.25	0.47	0.49	0.10	0.60	0.97
**EPR**	palmitic acid	0.01	0.96	0.97	-0.09	0.63	0.97
**EPR**	stearic acid	0.04	0.83	0.85	-0.15	0.41	0.91
**EPR**	cholesterol	0.22	0.25	0.27	0.06	0.76	0.98
**EPR**	17a(H)-22,29,30-trisnorhopane (C27a)	0.37	4.70E-02	7.73E-02	0.25	0.17	0.89
**EPR**	17b(H),21a(H)-norhopane (C30ba)	0.37	4.89E-02	7.92E-02	0.06	0.74	0.98
**EPR**	2-methylthreitol	0.28	0.20	0.23	-0.04	0.85	0.99
**EPR**	2-methylerythritol	0.42	4.33E-02	7.62E-02	-0.06	0.75	0.98
**EPR**	2-methylglyceric acid	0.39	6.34E-02	9.63E-02	0.23	0.23	0.89
**EPR**	cis-2-methyl-1,3,4-trihydroxy-1-butene	0.27	0.21	0.24	0.00	0.98	0.99
**EPR**	3-methyl-2,3,4-trihydroxy-1-butene	0.31	0.16	0.19	0.21	0.28	0.89
**EPR**	trans-2-methyl-1,3,4-trihydroxy-1-butene	0.29	0.18	0.22	0.12	0.54	0.97
**EPR**	C5-alkene triols	0.27	0.22	0.25	0.13	0.49	0.96
**EPR**	2-methyltetrols	0.38	7.66E-02	0.11	-0.06	0.76	0.98
**EPR**	3-hydroxyglutaric acid	0.31	0.15	0.19	-0.03	0.86	0.99
**EPR**	cis-pinonic acid	0.25	0.26	0.28	-0.22	0.26	0.89
**EPR**	pinic acid	0.26	0.24	0.26	-0.35	0.06	0.89

**EPR**	3-methyl-1,2,3-butanetricarboxylic acid	0.22	0.31	0.32	-0.03	0.89	0.99
**EPR**	β-caryophyllinic acid	0.33	0.13	0.17	0.16	0.42	0.91
**EPR**	glutaric acid derivative	0.31	0.15	0.19	-0.03	0.88	0.99
**EPR**	3-acetylpentanedioic acid	0.79	8.19E-06	4.38E-05	-0.22	0.24	0.89
**EPR**	3-acetylhexanedioic acid	0.66	6.41E-04	2.64E-03	-0.26	0.17	0.89
**EPR**	3-isopropyl-pentanedioic acid	0.63	1.23E-03	4.55E-03	-0.02	0.91	0.99
**EPR**	2,3-dihydroxy-4-oxopentanoic acid	0.66	6.75E-04	2.67E-03	0.07	0.74	0.98

Table S10. Spearman rank correlations for all AA_v assay responses with all individual measurements.

assay	feature	winter R^2	winter p-value	winter BH p-value	summer R^2	summer p-value	summer BH p-value
**AA**	total OC	0.92	2.14E-13	1.14E-11	0.49	3.66E-03	0.10
**AA**	total EC	0.88	9.30E-11	2.47E-09	0.30	9.43E-02	0.39
**AA**	K^+	0.86	8.60E-10	1.15E-08	0.23	0.22	0.54
**AA**	Na^+	0.81	4.32E-08	3.56E-07	0.14	0.47	0.66
**AA**	Ca^{2+}	0.43	1.68E-02	2.64E-02	0.12	0.53	0.73
**AA**	NH_4^+	0.84	3.71E-09	3.30E-08	0.16	0.36	0.64
**AA**	NO_3^-	0.84	3.24E-09	3.15E-08	0.35	4.50E-02	0.27
**AA**	SO_4^{2-}	0.75	1.05E-06	6.25E-06	0.47	5.64E-03	0.10
**AA**	Cl^-	0.93	5.23E-14	5.59E-12	0.02	0.90	0.92
**AA**	Al	0.65	7.89E-05	2.28E-04	0.23	0.20	0.54
**AA**	Ti	0.29	1.20E-01	1.42E-01	0.17	0.34	0.64
**AA**	V	0.11	0.57	0.61	0.41	0.13	0.46
**AA**	Cr	0.67	3.40E-05	1.10E-04	-0.12	0.52	0.72
**AA**	Mn	0.85	1.16E-09	1.38E-08	0.36	4.08E-02	0.27
**AA**	Fe	0.76	9.09E-07	5.72E-06	0.22	0.21	0.54
**AA**	Co	0.70	1.04E-05	4.13E-05	-0.50	0.67	0.78
**AA**	Ni	0.62	2.30E-04	5.71E-04	-0.43	0.11	0.42
**AA**	Cu	0.71	7.93E-06	3.54E-05	0.07	0.72	0.78
**AA**	Zn	0.87	2.68E-10	4.10E-09	0.15	0.40	0.66
**AA**	Cd	0.87	1.39E-10	2.47E-09	0.20	0.75	0.80
**AA**	Sb	0.88	1.16E-10	2.47E-09	0.18	0.63	0.77
**AA**	Ba	0.40	2.66E-02	3.56E-02	-0.17	0.40	0.66
**AA**	Pb	0.91	1.65E-12	5.90E-11	0.18	0.31	0.64
**AA**	galactosan	0.66	5.19E-05	1.63E-04	0.20	0.27	0.60
**AA**	mannosan	0.66	5.81E-05	1.78E-04	0.26	0.14	0.47
**AA**	levoglucosan	0.68	2.51E-05	8.97E-05	0.21	0.23	0.54
**AA**	ORG	0.90	3.09E-09	3.15E-08	0.54	2.27E-03	0.08
**AA**	MOOOA	0.81	1.38E-06	7.75E-06	0.49	7.35E-03	0.10
**AA**	LOOOA	0.76	1.48E-05	5.64E-05	0.40	3.07E-02	0.25
**AA**	O_3	-0.07	0.69	0.72	0.08	0.68	0.78
**AA**	CO	0.10	0.58	0.61	0.14	0.44	0.66
**AA**	NO	0.36	4.43E-02	5.64E-02	0.03	0.86	0.90
**AA**	NO_2	0.23	0.22	0.24	0.13	0.48	0.68
**AA**	NO_y	0.29	0.11	0.13	0.13	0.45	0.66
**AA**	SO_2	0.23	0.20	0.23	0.15	0.41	0.66
**AA**	RH8	0.53	1.96E-03	3.82E-03	0.17	0.36	0.64
**AA**	RH120	0.52	2.52E-03	4.72E-03	0.17	0.35	0.64
**AA**	RH240	0.50	4.22E-03	7.52E-03	0.17	0.36	0.64

**AA**	T8	0.16	0.38	0.43	0.07	0.71	0.78
**AA**	T120	0.15	0.42	0.47	0.08	0.67	0.78
**AA**	T240	0.14	0.45	0.50	0.07	0.70	0.78
**AA**	methanol	0.54	2.47E-02	3.42E-02	0.15	0.43	0.66
**AA**	acetonitrile	0.53	2.97E-02	3.93E-02	0.11	0.54	0.73
**AA**	acetaldehyde	0.56	1.84E-02	2.70E-02	0.10	0.60	0.77
**AA**	acrolein	0.51	4.27E-02	5.53E-02	0.18	0.33	0.64
**AA**	acetone	0.56	2.04E-02	2.95E-02	0.16	0.40	0.66
**AA**	isoprene	0.56	1.84E-02	2.70E-02	0.04	0.83	0.88
**AA**	methyl vinyl ketone /methacrolein	0.57	1.78E-02	2.70E-02	0.14	0.46	0.66
**AA**	methyl ethyl ketone	-0.08	0.78	0.79	0.14	0.45	0.66
**AA**	benzene	0.56	1.84E-02	2.70E-02	0.03	0.87	0.90
**AA**	toluene	0.60	1.12E-02	1.82E-02	-0.14	0.44	0.66
**AA**	C2-benzenes	0.58	1.55E-02	2.48E-02	-0.26	0.16	0.47
**AA**	C3-benzenes	0.51	5.37E-02	6.76E-02	-0.11	0.54	0.73
**AA**	J O^1D	0.25	0.18	0.20	0.07	0.69	0.78
**AA**	J NO_2	0.33	7.84E-02	9.64E-02	0.14	0.43	0.66
**AA**	naphthalene	0.88	3.11E-05	1.04E-04	0.13	0.59	0.76
**AA**	acenaphthylne	0.80	6.28E-04	1.32E-03	0.26	0.39	0.66
**AA**	acenaphthene	0.69	6.54E-03	1.11E-02	0.00	1.00	1.00
**AA**	fluorene	0.86	6.85E-05	2.03E-04	0.22	0.42	0.66
**AA**	phenanthrene	0.90	9.56E-06	3.93E-05	0.12	0.62	0.77
**AA**	fluoranthene	0.89	2.00E-05	7.37E-05	0.13	0.59	0.76
**AA**	pyrene	0.92	2.98E-06	1.60E-05	0.04	0.87	0.90
**AA**	benzo(a)anthracene	0.84	1.86E-04	4.99E-04	0.02	0.92	0.93
**AA**	chrysene	0.96	9.47E-08	6.76E-07	0.22	0.35	0.64
**AA**	benzo(b)fluoranthene	0.96	5.08E-08	3.88E-07	0.42	6.24E-02	0.31
**AA**	benzo(k)fluoranthene	0.90	9.56E-06	3.93E-05	0.40	7.69E-02	0.34
**AA**	benzo(a)pyrene	0.73	2.92E-03	5.30E-03	0.43	5.74E-02	0.31
**AA**	indeno(1,2,3-cd)pyrene	0.92	4.08E-06	1.99E-05	0.45	4.59E-02	0.27
**AA**	dibenzo(a,h)-anthracene	0.85	9.77E-05	2.75E-04	0.28	0.23	0.54
**AA**	benzo(ghi)perylene	0.92	4.08E-06	1.99E-05	0.18	0.44	0.66
**AA**	C24	0.59	5.00E-04	1.11E-03	-0.38	2.82E-02	0.25
**AA**	C25	0.59	4.94E-04	1.11E-03	-0.22	0.21	0.54
**AA**	C26	0.60	3.52E-04	8.55E-04	-0.09	0.63	0.77
**AA**	C27	0.58	6.29E-04	1.32E-03	0.06	0.74	0.80
**AA**	C28	0.59	4.78E-04	1.11E-03	0.25	0.16	0.47
**AA**	C29	0.63	1.32E-04	3.63E-04	0.26	0.15	0.47
**AA**	C30	0.58	5.96E-04	1.30E-03	0.28	0.11	0.42
**AA**	C31	0.62	2.30E-04	5.71E-04	0.33	6.14E-02	0.31
**AA**	C32	0.56	9.59E-04	1.94E-03	0.24	0.19	0.54
**AA**	C33	0.52	2.45E-03	4.68E-03	0.22	0.21	0.54
**AA**	C34	0.55	1.23E-03	2.45E-03	0.08	0.66	0.78
**AA**	OH	-0.14	0.62	0.65	-0.11	0.58	0.76
**AA**	HO_2	-0.01	0.98	0.98	-0.08	0.68	0.78
**AA**	RO_2	0.04	0.90	0.91	-0.09	0.65	0.78
**AA**	palmitic acid	0.07	0.70	0.72	0.30	8.71E-02	0.37
**AA**	stearic acid	0.11	0.58	0.61	0.36	3.96E-02	0.27
**AA**	cholesterol	0.28	0.13	0.15	0.09	0.62	0.77
**AA**	17a(H)-22,29,30-trisnorhopane (C27a)	0.61	3.74E-04	8.90E-04	0.32	6.63E-02	0.31
**AA**	17b(H),21a(H)-norhopane (C30ba)	0.59	6.72E-04	1.38E-03	0.21	0.23	0.54
**AA**	2-methylthreitol	0.47	2.17E-02	3.05E-02	0.18	0.36	0.64

**AA**	2-methylerythritol	0.59	2.66E-03	4.92E-03	0.21	0.29	0.61
**AA**	2-methylglyceric acid	0.52	9.76E-03	1.61E-02	0.35	6.41E-02	0.31
**AA**	cis-2-methyl-1,3,4-trihydroxy-1-butene	0.56	4.51E-03	7.90E-03	0.29	0.12	0.44
**AA**	3-methyl-2,3,4-trihydroxy-1-butene	0.47	2.14E-02	3.05E-02	0.28	0.14	0.47
**AA**	trans-2-methyl-1,3,4-trihydroxy-1-butene	0.48	1.81E-02	2.70E-02	0.51	4.58E-03	0.10
**AA**	C5-alkene triols	0.46	2.52E-02	3.42E-02	0.46	1.18E-02	0.13
**AA**	2-methyltetrols	0.56	4.66E-03	8.04E-03	0.19	0.31	0.64
**AA**	3-hydroxyglutaric acid	0.46	2.49E-02	3.42E-02	0.49	7.35E-03	0.10
**AA**	cis-pinonic acid	0.39	5.74E-02	7.15E-02	0.29	0.12	0.44
**AA**	pinic acid	0.42	4.29E-02	5.53E-02	0.22	0.24	0.55
**AA**	3-methyl-1,2,3-butanetricarboxylic acid	0.29	0.18	0.20	0.24	0.22	0.54
**AA**	β-caryophyllinic acid	0.53	7.32E-03	1.22E-02	0.38	4.39E-02	0.27
**AA**	glutaric acid derivative	0.33	0.11	0.14	0.22	0.25	0.57
**AA**	3-acetylpentanedioic acid	0.83	6.97E-07	4.66E-06	0.59	7.82E-04	0.05
**AA**	3-acetylhexanedioic acid	0.75	2.94E-05	1.01E-04	0.43	2.00E-02	0.19
**AA**	3-isopropyl-pentanedioic acid	0.69	1.98E-04	5.18E-04	0.58	8.98E-04	0.05
**AA**	2,3-dihydroxy-4-oxopentanoic acid	0.78	5.96E-06	2.77E-05	0.47	1.07E-02	0.13

Table S11. Spearman rank correlations for all DTT_v assay responses with all individual measurements.

assay	feature	winter R^2	winter p-value	winter BH p-value	summer R^2	summer p-value	summer BH p-value
**DTT**	total OC	0.84	2.82E-09	1.24E-07	0.64	5.77E-05	5.91E-03
**DTT**	total EC	0.78	1.83E-07	2.40E-06	0.41	1.79E-02	7.97E-02
**DTT**	K^+	0.77	3.15E-07	3.37E-06	0.56	1.02E-03	1.32E-02
**DTT**	Na^+	0.77	5.07E-07	4.93E-06	0.40	2.68E-02	1.02E-01
**DTT**	Ca^{2+}	0.64	1.56E-04	5.76E-04	0.19	0.30	0.53
**DTT**	NH_4^+	0.81	3.76E-08	6.71E-07	0.60	2.48E-04	5.91E-03
**DTT**	NO_3^-	0.78	2.02E-07	2.40E-06	0.53	1.39E-03	1.32E-02
**DTT**	SO_4^{2-}	0.75	1.07E-06	8.21E-06	0.62	1.37E-04	5.91E-03
**DTT**	Cl^-	0.83	6.75E-09	1.81E-07	0.24	0.18	0.38
**DTT**	Al	0.47	7.38E-03	1.58E-02	0.20	0.27	0.50
**DTT**	Ti	0.23	0.22	0.30	0.24	0.17	0.37
**DTT**	V	0.09	0.66	0.70	-0.12	0.68	0.84
**DTT**	Cr	0.59	4.94E-04	1.39E-03	0.28	0.12	0.31
**DTT**	Mn	0.80	5.82E-08	8.90E-07	0.40	1.97E-02	8.43E-02
**DTT**	Fe	0.75	1.07E-06	8.21E-06	0.37	3.21E-02	0.12
**DTT**	Co	0.63	1.41E-04	5.39E-04	0.50	0.67	0.84
**DTT**	Ni	0.57	7.46E-04	1.95E-03	-0.18	0.53	0.75
**DTT**	Cu	0.64	1.15E-04	4.72E-04	0.29	0.15	0.35
**DTT**	Zn	0.77	6.08E-07	5.42E-06	0.53	1.48E-03	1.32E-02
**DTT**	Cd	0.83	8.62E-09	1.84E-07	0.10	0.87	0.94
**DTT**	Sb	0.84	3.46E-09	1.24E-07	-0.14	0.71	0.86
**DTT**	Ba	0.47	7.38E-03	1.58E-02	0.01	0.94	0.97
**DTT**	Pb	0.85	1.39E-09	1.24E-07	0.53	1.38E-03	1.32E-02
**DTT**	galactosan	0.50	4.29E-03	9.97E-03	0.42	1.46E-02	7.09E-02
**DTT**	mannosan	0.54	1.80E-03	4.59E-03	0.48	4.87E-03	3.72E-02

**DTT**	levoglucosan	0.53	2.15E-03	5.36E-03	0.56	6.21E-04	1.11E-02
**DTT**	ORG	0.79	4.49E-06	2.40E-05	0.63	2.76E-04	5.91E-03
**DTT**	MOOOA	0.80	2.95E-06	1.68E-05	0.32	0.10	0.25
**DTT**	LOOOA	0.67	3.63E-04	1.11E-03	0.42	2.34E-02	9.64E-02
**DTT**	O_3	-0.07	0.73	0.75	0.24	0.19	0.39
**DTT**	CO	-0.03	0.88	0.89	0.05	0.79	0.89
**DTT**	NO	0.27	0.14	0.20	-0.11	0.52	0.75
**DTT**	NO_2	0.12	0.54	0.57	-0.03	0.86	0.94
**DTT**	NO_y	0.21	0.26	0.34	-0.10	0.57	0.77
**DTT**	SO_2	0.04	0.85	0.87	0.34	5.00E-02	0.17
**DTT**	RH8	0.62	1.96E-04	6.76E-04	0.10	0.60	0.77
**DTT**	RH120	0.61	2.78E-04	9.02E-04	0.11	0.54	0.75
**DTT**	RH240	0.60	3.81E-04	1.13E-03	0.12	0.52	0.75
**DTT**	T8	0.22	0.23	0.31	0.20	0.29	0.51
**DTT**	T120	0.21	0.26	0.34	0.16	0.38	0.63
**DTT**	T240	0.20	0.27	0.34	0.15	0.44	0.67
**DTT**	methanol	0.25	0.32	0.38	0.01	0.94	0.97
**DTT**	acetonitrile	0.21	0.41	0.46	0.06	0.74	0.87
**DTT**	acetaldehyde	0.25	0.32	0.38	0.12	0.52	0.75
**DTT**	acrolein	0.20	0.46	0.50	0.03	0.86	0.94
**DTT**	acetone	0.26	0.31	0.38	0.32	7.91E-02	0.22
**DTT**	isoprene	0.28	0.27	0.34	-0.33	6.57E-02	0.19
**DTT**	methyl vinyl ketone /methacrolein	0.25	0.34	0.39	-0.22	0.24	0.45
**DTT**	methyl ethyl ketone	-0.22	0.42	0.46	0.17	0.36	0.61
**DTT**	benzene	0.25	0.33	0.39	-0.03	0.86	0.94
**DTT**	toluene	0.29	0.26	0.34	0.01	0.97	0.98
**DTT**	C2-benzenes	0.28	0.27	0.34	-0.05	0.78	0.89
**DTT**	C3-benzenes	0.24	0.39	0.44	0.01	0.95	0.97
**DTT**	J O^1D	0.48	7.82E-03	1.60E-02	0.00	0.99	0.99
**DTT**	J NO_2	0.48	7.73E-03	1.60E-02	0.10	0.58	0.77
**DTT**	naphthalene	0.85	1.36E-04	5.39E-04	-0.04	0.89	0.95
**DTT**	acenaphthylne	0.88	3.83E-05	1.78E-04	0.09	0.76	0.89
**DTT**	acenaphthene	0.79	7.08E-04	1.89E-03	0.03	0.91	0.97
**DTT**	fluorene	0.92	2.98E-06	1.68E-05	0.29	0.27	0.50
**DTT**	phenanthrene	0.93	2.14E-06	1.43E-05	0.13	0.59	0.77
**DTT**	fluoranthene	0.81	4.32E-04	1.25E-03	0.33	0.15	0.35
**DTT**	pyrene	0.82	3.31E-04	1.04E-03	0.29	0.21	0.42
**DTT**	benzo(a)anthracene	0.83	2.17E-04	7.24E-04	0.19	0.43	0.67
**DTT**	chrysene	0.92	2.98E-06	1.68E-05	0.33	0.15	0.35
**DTT**	benzo(b)fluoranthene	0.93	2.14E-06	1.43E-05	0.57	8.28E-03	5.21E-02
**DTT**	benzo(k)fluoranthene	0.86	8.20E-05	3.66E-04	0.46	3.97E-02	0.14
**DTT**	benzo(a)pyrene	0.75	2.23E-03	5.42E-03	0.54	1.40E-02	7.09E-02
**DTT**	indeno(1,2,3-cd)pyrene	0.90	1.24E-05	6.30E-05	0.54	1.43E-02	7.09E-02
**DTT**	dibenzo(a,h)-anthracene	0.85	9.77E-05	4.18E-04	0.40	7.81E-02	0.22
**DTT**	benzo(ghi)perylene	0.89	1.58E-05	7.68E-05	0.28	0.23	0.45
**DTT**	C24	0.40	2.61E-02	4.46E-02	-0.19	0.28	0.50
**DTT**	C25	0.40	2.63E-02	4.46E-02	-0.08	0.68	0.84
**DTT**	C26	0.42	1.89E-02	3.42E-02	0.15	0.41	0.65
**DTT**	C27	0.41	2.28E-02	4.00E-02	0.25	0.16	0.35
**DTT**	C28	0.47	7.91E-03	1.60E-02	0.33	6.48E-02	0.19
**DTT**	C29	0.47	8.28E-03	1.64E-02	0.44	1.12E-02	6.33E-02
**DTT**	C30	0.41	2.07E-02	3.69E-02	0.37	3.64E-02	0.13
**DTT**	C31	0.42	1.74E-02	3.21E-02	0.33	6.31E-02	0.19
**DTT**	C32	0.46	9.63E-03	1.87E-02	0.28	0.12	0.30
**DTT**	C33	0.39	2.98E-02	4.98E-02	0.42	1.62E-02	7.54E-02

**DTT**	C34	0.39	3.22E-02	5.30E-02	0.07	0.69	0.84
**DTT**	OH	-0.02	0.95	0.95	0.19	0.34	0.58
**DTT**	HO_2	0.34	0.28	0.34	-0.05	0.79	0.89
**DTT**	RO_2	0.30	0.34	0.39	0.15	0.42	0.66
**DTT**	palmitic acid	0.07	0.70	0.72	0.06	0.72	0.86
**DTT**	stearic acid	0.08	0.68	0.72	0.11	0.54	0.75
**DTT**	cholesterol	0.16	0.39	0.44	0.61	1.79E-04	5.91E-03
**DTT**	17a(H)-22,29,30-trisnorhopane (C27a)	0.43	1.67E-02	3.13E-02	0.07	0.69	0.84
**DTT**	17b(H),21a(H)-norhopane (C30ba)	0.36	5.12E-02	8.17E-02	0.01	0.96	0.98
**DTT**	2-methylthreitol	0.36	8.32E-02	0.12	0.15	0.44	0.67
**DTT**	2-methylerythritol	0.54	6.25E-03	1.42E-02	0.18	0.35	0.59
**DTT**	2-methylglyceric acid	0.37	7.62E-02	0.11	0.35	6.17E-02	0.19
**DTT**	cis-2-methyl-1,3,4-trihydroxy-1-butene	0.38	6.84E-02	0.11	0.24	0.21	0.42
**DTT**	3-methyl-2,3,4-trihydroxy-1-butene	0.42	4.24E-02	6.88E-02	0.29	0.13	0.31
**DTT**	trans-2-methyl-1,3,4-trihydroxy-1-butene	0.38	6.98E-02	0.11	0.49	6.82E-03	4.56E-02
**DTT**	C5-alkene triols	0.34	0.10	0.15	0.42	2.47E-02	9.79E-02
**DTT**	2-methyltetrols	0.49	1.44E-02	2.74E-02	0.16	0.40	0.64
**DTT**	3-hydroxyglutaric acid	0.28	0.19	0.27	0.57	1.13E-03	1.32E-02
**DTT**	cis-pinonic acid	0.33	0.12	0.17	-0.10	0.60	0.77
**DTT**	pinic acid	0.27	0.20	0.28	0.25	0.19	0.39
**DTT**	3-methyl-1,2,3-butanetricarboxylic acid	0.15	0.48	0.52	-0.12	0.55	0.75
**DTT**	β-caryophyllinic acid	0.40	5.51E-02	8.67E-02	0.58	1.08E-03	1.32E-02
**DTT**	glutaric acid derivative	0.22	0.31	0.38	0.29	0.13	0.32
**DTT**	3-acetylpentanedioic acid	0.69	1.69E-04	6.04E-04	0.35	6.09E-02	0.19
**DTT**	3-acetylhexanedioic acid	0.59	2.39E-03	5.67E-03	0.50	6.32E-03	4.51E-02
**DTT**	3-isopropyl-pentanedioic acid	0.54	6.45E-03	1.44E-02	0.47	1.04E-02	6.17E-02
**DTT**	2,3-dihydroxy-4-oxopentanoic acid	0.65	6.34E-04	1.74E-03	0.55	1.83E-03	1.51E-02

Table S12. Spearman rank correlations for all $DCFH_v$ assay responses with all individual measurements.

assay	feature	winter R^2	winter p-value	winter BH p-value	summer R^2	summer p-value	summer BH p-value
**DCFH**	total OC	0.89	2.02E-11	4.32E-10	0.64	5.84E-05	6.94E-04
**DCFH**	total EC	0.80	8.64E-08	5.77E-07	0.20	0.28	0.45
**DCFH**	K^+	0.86	4.11E-10	5.24E-09	0.53	1.98E-03	1.18E-02
**DCFH**	Na^+	0.75	9.48E-07	5.63E-06	0.17	0.35	0.54
**DCFH**	Ca^{2+}	0.47	8.62E-03	1.54E-02	-0.10	0.59	0.74
**DCFH**	NH_4^+	0.96	5.41E-17	5.79E-15	0.41	1.75E-02	5.50E-02
**DCFH**	NO_3^-	0.91	5.98E-13	2.13E-11	0.62	1.14E-04	1.02E-03
**DCFH**	SO_4^{2-}	0.93	9.51E-14	5.09E-12	0.77	1.89E-07	6.73E-06
**DCFH**	Cl^-	0.86	4.41E-10	5.24E-09	0.08	0.66	0.78
**DCFH**	Al	0.68	2.22E-05	9.51E-05	0.24	0.18	0.35
**DCFH**	Ti	0.32	8.55E-02	0.10	0.21	0.25	0.43
**DCFH**	V	0.19	0.31	0.33	0.26	0.35	0.54
**DCFH**	Cr	0.68	2.48E-05	1.02E-04	0.16	0.39	0.58
**DCFH**	Mn	0.86	3.64E-10	5.24E-09	0.37	3.48E-02	9.99E-02

**DCFH**	Fe	0.77	5.07E-07	3.19E-06	0.20	0.25	0.43
**DCFH**	Co	0.73	2.89E-06	1.55E-05	0.50	0.67	0.78
**DCFH**	Ni	0.62	1.82E-04	6.48E-04	-0.26	0.35	0.54
**DCFH**	Cu	0.69	1.54E-05	7.18E-05	0.36	6.87E-02	0.17
**DCFH**	Zn	0.84	8.10E-09	6.19E-08	0.63	8.19E-05	8.76E-04
**DCFH**	Cd	0.87	1.51E-10	2.70E-09	0.10	0.87	0.94
**DCFH**	Sb	0.81	4.20E-08	3.00E-07	-0.21	0.59	0.74
**DCFH**	Ba	0.41	2.28E-02	3.44E-02	-0.13	0.55	0.70
**DCFH**	Pb	0.90	9.75E-12	2.61E-10	0.63	9.23E-05	8.98E-04
**DCFH**	galactosan	0.57	7.39E-04	2.26E-03	0.44	1.10E-02	4.37E-02
**DCFH**	mannosan	0.57	8.21E-04	2.44E-03	0.42	1.58E-02	5.13E-02
**DCFH**	levoglucosan	0.58	6.71E-04	2.11E-03	0.42	1.55E-02	5.13E-02
**DCFH**	ORG	0.91	1.01E-09	1.08E-08	0.74	5.14E-06	9.16E-05
**DCFH**	MOOOA	0.90	3.09E-09	3.00E-08	0.50	5.86E-03	2.78E-02
**DCFH**	LOOOA	0.89	4.01E-09	3.30E-08	0.70	2.28E-05	3.05E-04
**DCFH**	O_3	-0.23	0.21	0.24	0.33	6.00E-02	0.15
**DCFH**	CO	0.27	0.13	0.16	0.20	0.25	0.43
**DCFH**	NO	0.44	1.34E-02	2.13E-02	-0.24	0.18	0.35
**DCFH**	NO_2	0.32	8.07E-02	0.10	-0.02	0.91	0.95
**DCFH**	NO_y	0.40	2.51E-02	3.73E-02	-0.09	0.63	0.76
**DCFH**	SO_2	0.37	3.83E-02	5.19E-02	0.26	0.14	0.29
**DCFH**	RH8	0.71	8.94E-06	4.35E-05	0.23	0.22	0.40
**DCFH**	RH120	0.69	1.67E-05	7.46E-05	0.27	0.13	0.28
**DCFH**	RH240	0.65	7.07E-05	2.80E-04	0.28	0.13	0.28
**DCFH**	T8	0.25	0.18	0.21	0.25	0.18	0.35
**DCFH**	T120	0.24	0.19	0.21	0.21	0.26	0.43
**DCFH**	T240	0.23	0.21	0.24	0.17	0.36	0.55
**DCFH**	methanol	0.57	1.61E-02	2.53E-02	0.15	0.41	0.59
**DCFH**	acetonitrile	0.59	1.21E-02	1.99E-02	0.37	4.16E-02	0.11
**DCFH**	acetaldehyde	0.67	3.01E-03	7.66E-03	0.28	0.13	0.28
**DCFH**	acrolein	0.63	9.41E-03	1.65E-02	-0.08	0.68	0.79
**DCFH**	acetone	0.67	3.01E-03	7.66E-03	0.32	8.03E-02	0.19
**DCFH**	isoprene	0.65	4.78E-03	1.00E-02	-0.42	2.02E-02	6.16E-02
**DCFH**	methyl vinyl ketone /methacrolein	0.62	7.61E-03	1.38E-02	-0.29	0.11	0.24
**DCFH**	methyl ethyl ketone	-0.14	0.59	0.62	0.33	7.14E-02	0.17
**DCFH**	benzene	0.64	5.68E-03	1.11E-02	0.14	0.44	0.62
**DCFH**	toluene	0.66	3.81E-03	8.67E-03	0.14	0.46	0.62
**DCFH**	C2-benzenes	0.66	3.99E-03	8.71E-03	0.02	0.91	0.95
**DCFH**	C3-benzenes	0.51	4.98E-02	6.58E-02	-0.08	0.69	0.79
**DCFH**	J O^1D	0.22	0.25	0.27	0.13	0.46	0.62
**DCFH**	J NO_2	0.23	0.23	0.25	0.14	0.43	0.62
**DCFH**	naphthalene	0.82	3.31E-04	1.07E-03	-0.16	0.51	0.67
**DCFH**	acenaphthylne	0.57	3.20E-02	4.44E-02	-0.01	0.99	0.99
**DCFH**	acenaphthene	0.59	2.60E-02	3.81E-02	0.06	0.85	0.92
**DCFH**	fluorene	0.70	5.21E-03	1.03E-02	0.02	0.95	0.97
**DCFH**	phenanthrene	0.70	5.21E-03	1.03E-02	0.36	0.12	0.25
**DCFH**	fluoranthene	0.78	9.94E-04	2.88E-03	0.58	7.29E-03	3.25E-02
**DCFH**	pyrene	0.77	1.23E-03	3.47E-03	0.48	3.23E-02	9.61E-02
**DCFH**	benzo(a)anthracene	0.59	2.74E-02	3.96E-02	0.28	0.24	0.43
**DCFH**	chrysene	0.82	3.31E-04	1.07E-03	0.60	5.16E-03	2.63E-02
**DCFH**	benzo(b)fluoranthene	0.86	8.20E-05	3.03E-04	0.67	1.21E-03	7.62E-03
**DCFH**	benzo(k)fluoranthene	0.86	8.20E-05	3.03E-04	0.55	1.19E-02	4.56E-02
**DCFH**	benzo(a)pyrene	0.49	7.83E-02	9.85E-02	0.60	5.16E-03	2.63E-02
**DCFH**	indeno(1,2,3-cd)pyrene	0.72	3.78E-03	8.67E-03	0.54	1.34E-02	4.77E-02
**DCFH**	dibenzo(a,h)-anthracene	0.69	6.07E-03	1.16E-02	0.30	0.20	0.37
**DCFH**	benzo(ghi)perylene	0.72	3.48E-03	8.38E-03	0.18	0.46	0.62

**DCFH**	C24	0.48	6.40E-03	1.20E-02	-0.42	1.58E-02	5.13E-02
**DCFH**	C25	0.47	7.32E-03	1.35E-02	-0.34	5.42E-02	0.14
**DCFH**	C26	0.50	3.94E-03	8.71E-03	-0.11	0.54	0.70
**DCFH**	C27	0.45	1.05E-02	1.79E-02	0.04	0.84	0.92
**DCFH**	C28	0.51	3.46E-03	8.38E-03	0.19	0.29	0.46
**DCFH**	C29	0.54	1.72E-03	4.71E-03	0.31	7.53E-02	0.18
**DCFH**	C30	0.50	4.32E-03	9.25E-03	0.13	0.47	0.63
**DCFH**	C31	0.52	2.70E-03	7.23E-03	-0.01	0.97	0.98
**DCFH**	C32	0.51	3.52E-03	8.38E-03	-0.02	0.90	0.95
**DCFH**	C33	0.37	3.83E-02	5.19E-02	0.24	0.19	0.35
**DCFH**	C34	0.42	1.85E-02	2.87E-02	-0.06	0.75	0.85
**DCFH**	OH	0.03	0.92	0.93	0.02	0.92	0.95
**DCFH**	HO_2	0.05	0.88	0.90	-0.10	0.61	0.76
**DCFH**	RO_2	-0.02	0.95	0.95	0.09	0.63	0.76
**DCFH**	palmitic acid	0.07	0.71	0.73	-0.05	0.79	0.89
**DCFH**	stearic acid	0.11	0.56	0.59	0.04	0.81	0.90
**DCFH**	cholesterol	0.21	0.26	0.28	0.45	8.19E-03	3.37E-02
**DCFH**	17a(H)-22,29,30-trisnorhopane (C27a)	0.45	1.32E-02	2.13E-02	-0.09	0.63	0.76
**DCFH**	17b(H),21a(H)-norhopane (C30ba)	0.45	1.21E-02	1.99E-02	-0.16	0.37	0.55
**DCFH**	2-methylthreitol	0.38	6.37E-02	8.11E-02	0.46	1.24E-02	4.59E-02
**DCFH**	2-methylerythritol	0.55	5.15E-03	1.03E-02	0.50	5.97E-03	2.78E-02
**DCFH**	2-methylglyceric acid	0.45	2.92E-02	4.12E-02	0.62	2.92E-04	2.41E-03
**DCFH**	cis-2-methyl-1,3,4-trihydroxy-1-butene	0.36	8.16E-02	0.10	0.39	3.64E-02	9.99E-02
**DCFH**	3-methyl-2,3,4-trihydroxy-1-butene	0.34	0.10	0.12	0.32	8.88E-02	0.20
**DCFH**	trans-2-methyl-1,3,4-trihydroxy-1-butene	0.45	2.89E-02	4.12E-02	0.72	1.02E-05	1.55E-04
**DCFH**	C5-alkene triols	0.40	5.33E-02	6.96E-02	0.61	4.88E-04	3.73E-03
**DCFH**	2-methyltetrols	0.52	9.76E-03	1.68E-02	0.48	7.77E-03	3.33E-02
**DCFH**	3-hydroxyglutaric acid	0.41	4.79E-02	6.41E-02	0.58	8.98E-04	6.00E-03
**DCFH**	cis-pinonic acid	0.34	0.11	0.13	-0.20	0.29	0.46
**DCFH**	pinic acid	0.39	6.30E-02	8.11E-02	0.39	3.56E-02	9.99E-02
**DCFH**	3-methyl-1,2,3-butanetricarboxylic acid	0.28	0.18	0.21	-0.02	0.92	0.95
**DCFH**	β-caryophyllinic acid	0.47	2.03E-02	3.11E-02	0.82	4.82E-08	2.58E-06
**DCFH**	glutaric acid derivative	0.32	0.13	0.15	0.55	2.22E-03	1.25E-02
**DCFH**	3-acetylpentanedioic acid	0.89	4.01E-09	3.30E-08	0.58	8.98E-04	6.00E-03
**DCFH**	3-acetylhexanedioic acid	0.79	3.81E-06	1.94E-05	0.77	1.28E-06	2.74E-05
**DCFH**	3-isopropylpentanedioic acid	0.69	2.15E-04	7.40E-04	0.77	1.18E-06	2.74E-05
**DCFH**	2,3-dihydroxy-4-oxopentanoic acid	0.81	1.58E-06	8.91E-06	0.89	8.16E-11	8.73E-09

Section S9: Multivariate modelling (mass-normalised data only)

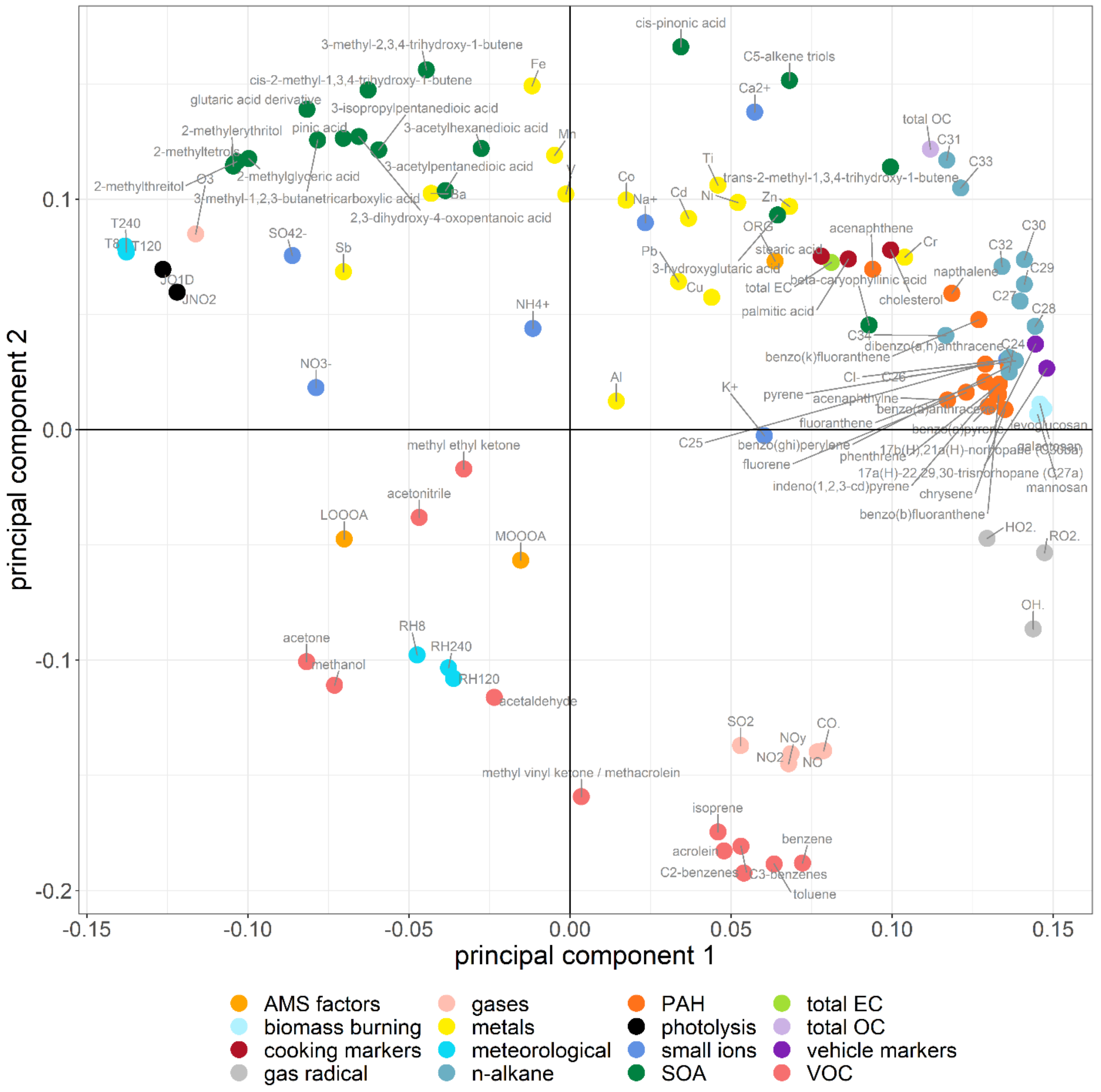

Figure S14. Principal components analysis loading plot for all data points. Points are coloured by measurement category.

PLSR models

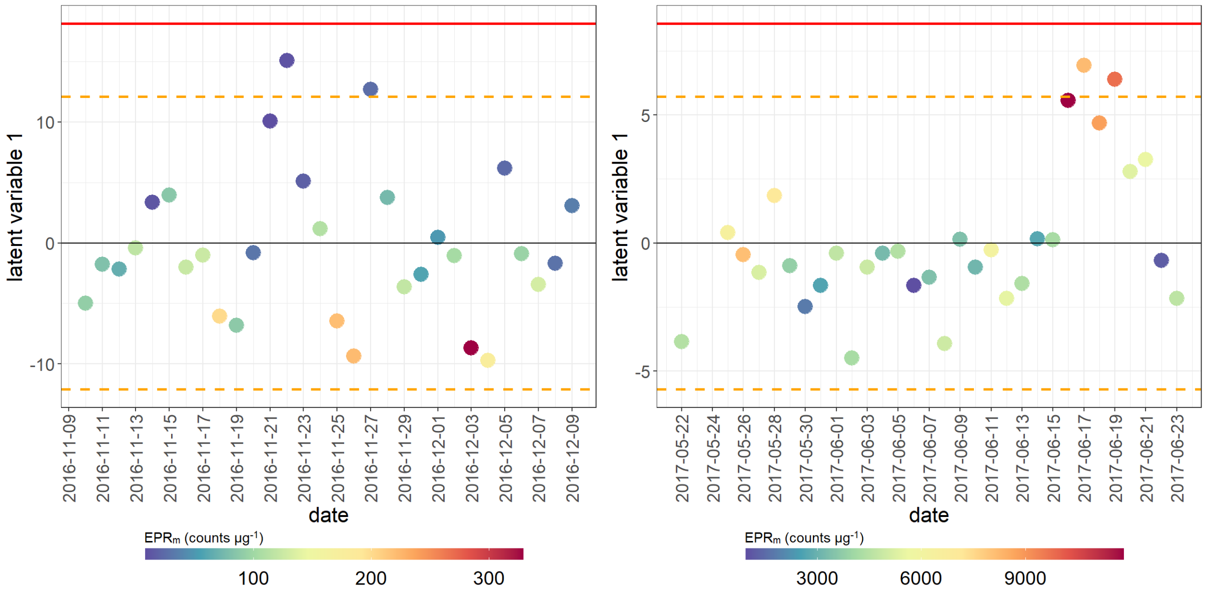

Figure S15. PLSR scores plots for EPR_m assay. Left: winter samples, LV 1 R^2Y 43.2%, Q^2 19.3%; right: summer samples LV 1 R^2Y 11.3%, Q^2 -10.0%. Models optimised to a single latent variable only.

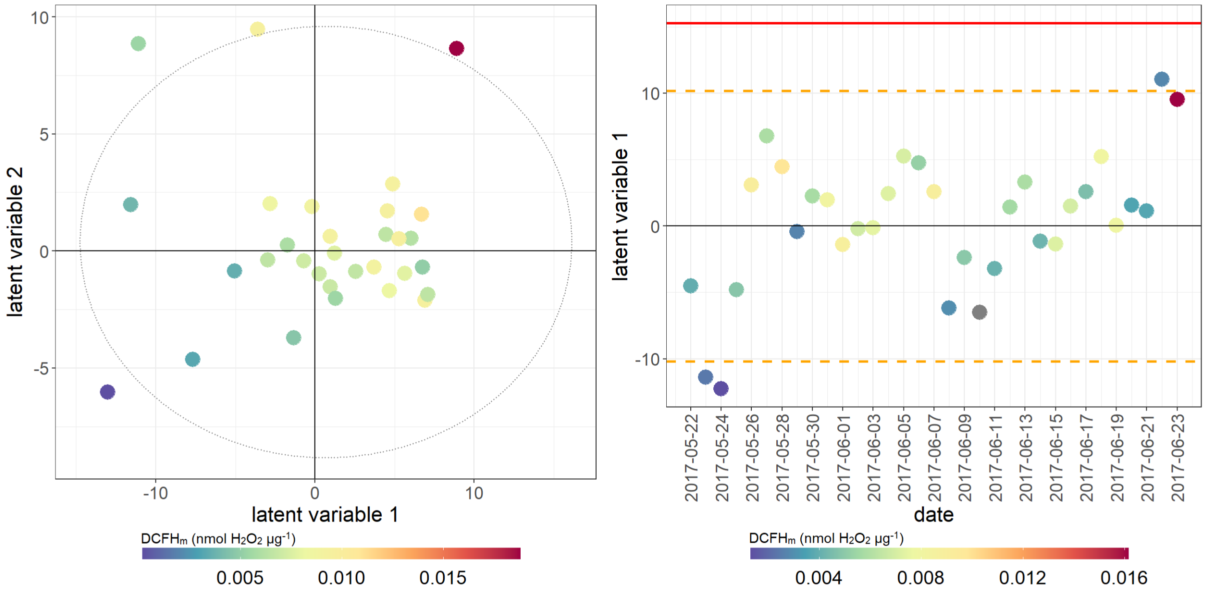

Figure S16. PLSR scores plot for $DCFH_m$ assay. Left: winter samples, optimised to two latent variables; LV 1 R^2Y 40.8%, Q^2 21.9%, LV 2 R^2Y 31.2%, Q^2 36.5% (second component slightly overfits model); right: summer samples, optimised to one latent variable; LV 1 R^2Y 28.2%, Q^2 -6.6%.

VIP plots

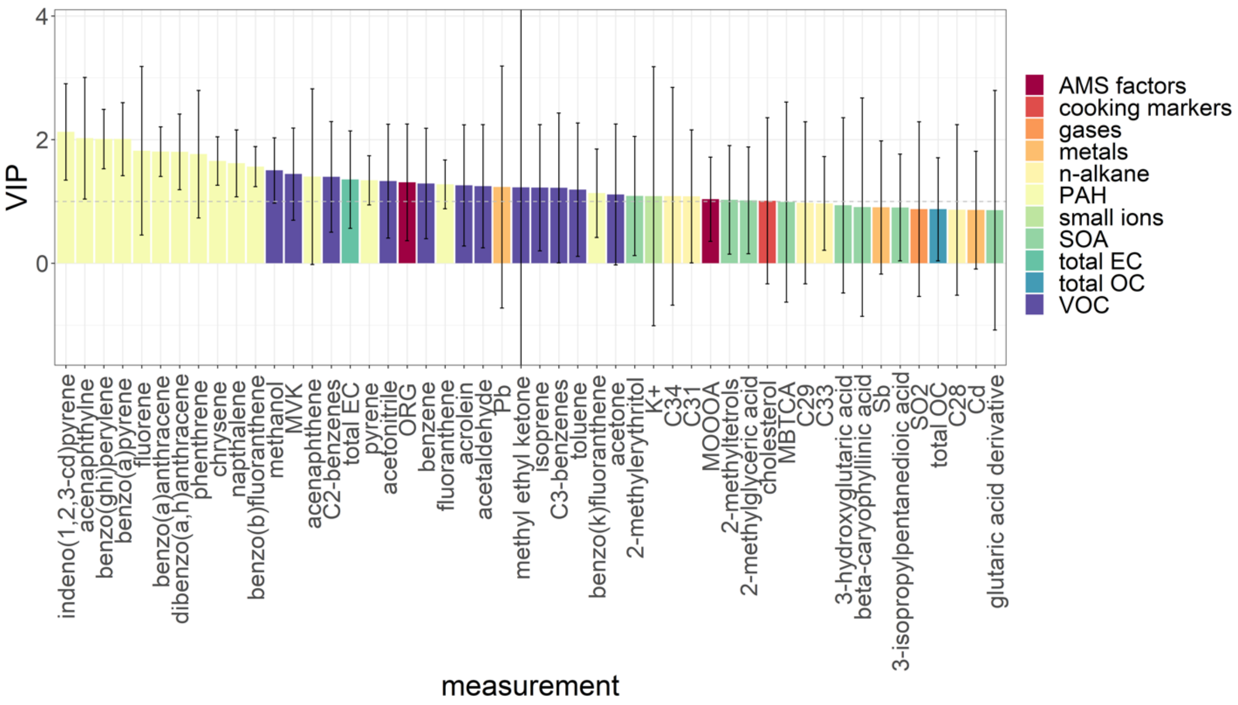

Figure S17. Variable importance in projection (VIP) plot for winter EPR_m PLSR model (top 50 features only). Error bars represent the standard error or the mean for each feature, and are often large due to the intrinsic noisiness and instability of the individual measurements. Terms with VIP > 1 contribute most significantly to the model. **Abbreviations:** 3MTHB: 3-methyl-2,3,4-trihydroxy-1-butene; C2MTHB: cis-2-methyl-1,3,4-trihydroxy-1-butene; T2MTHB: trans-2-methyl-1,3,4-trihydroxy-1-butene; 17a-TNH:17a(H)-22,29,30-trisnorhopane (C27a); 17b-NH: 17b(H),21a(H)-norhopane (C30ba); MVK: methyl vinyl ketone or methacrolein.

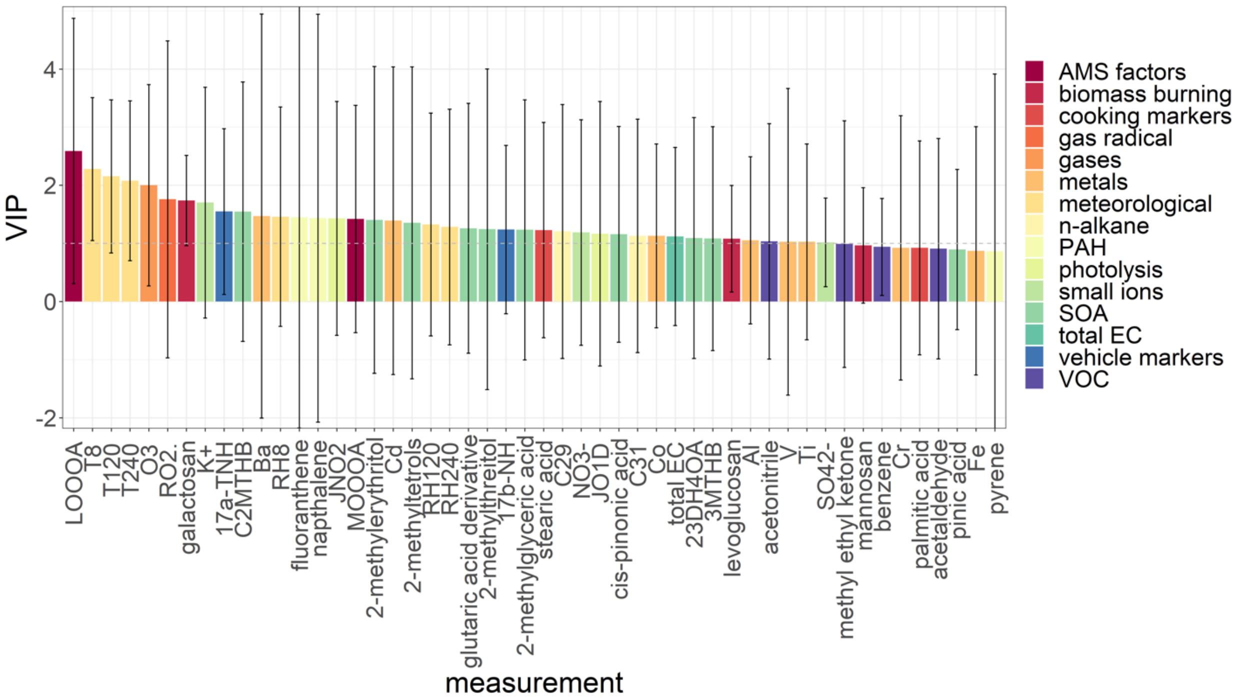

Figure S18. Variable importance in projection (VIP) plot for summer EPR_m PLSR model (top 50 features only).

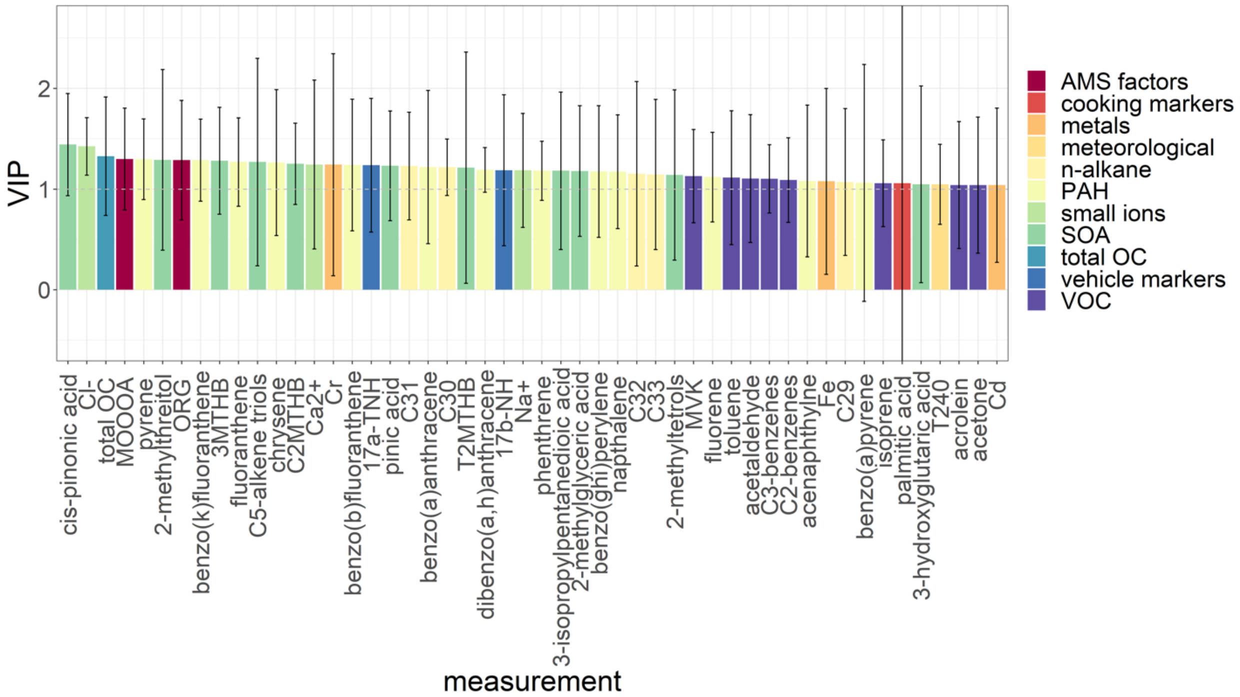

Figure S19. Variable importance in projection (VIP) plot for winter AA_m PLSR model (top 50 features only).

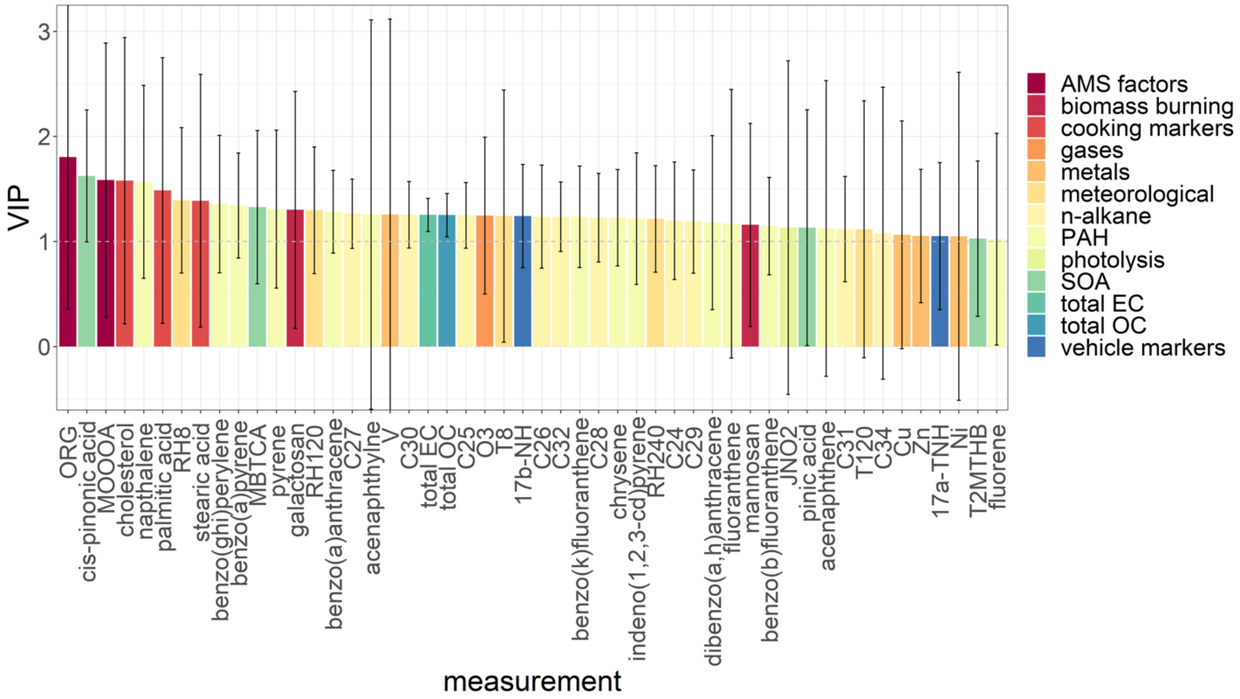

Figure S20. Variable importance in projection (VIP) plot for summer AA_m PLSR model (top 50 features only).

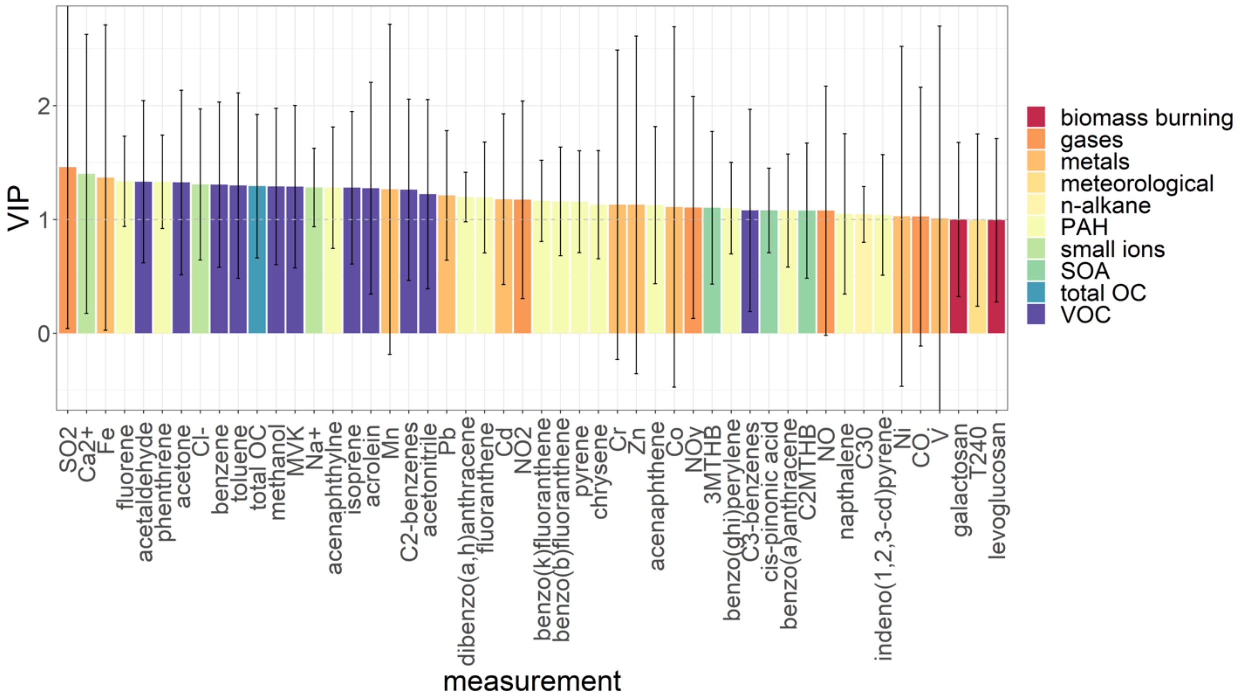

Figure S21. Variable importance in projection (VIP) plot for winter DTT_m PLSR model (top 50 features only).

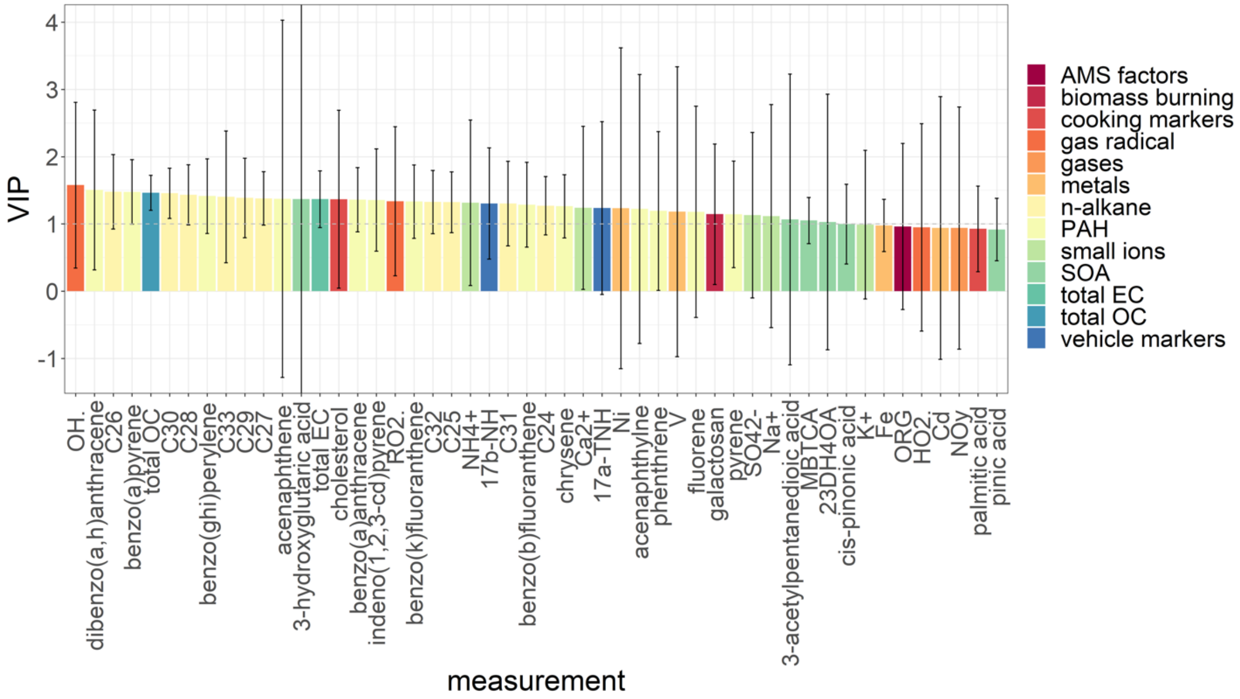

Figure S22. Variable importance in projection (VIP) plot for summer DTT_m PLSR model (top 50 features only).

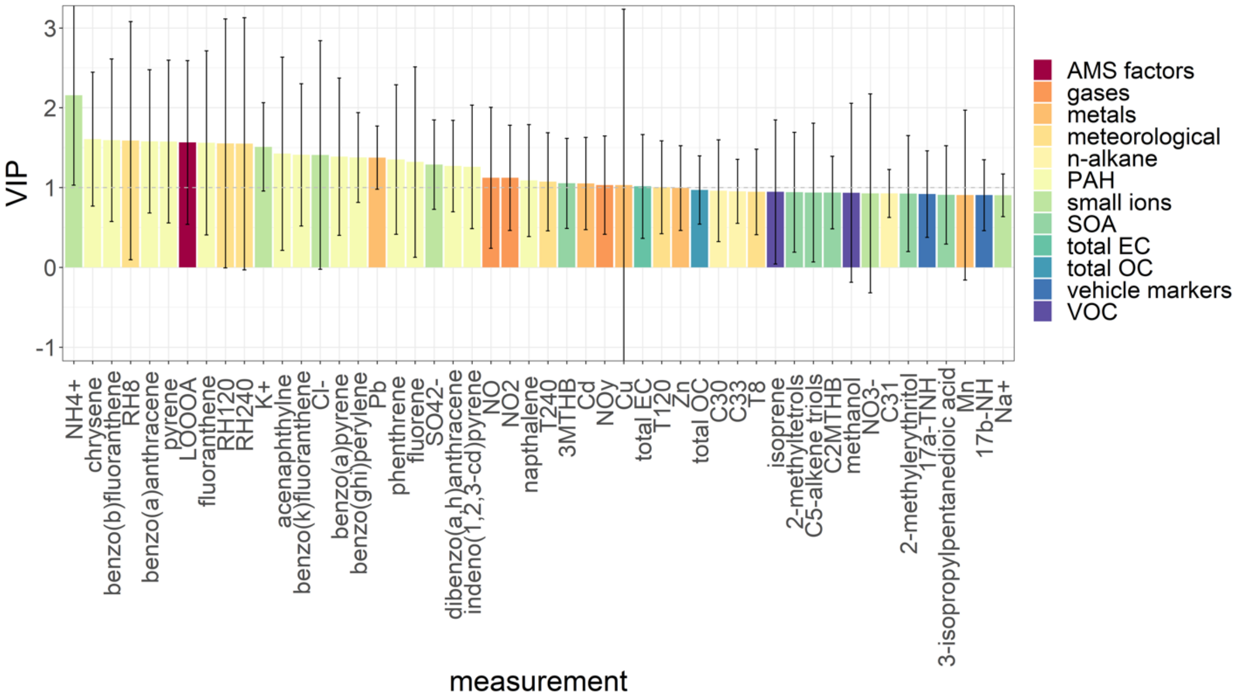

Figure S23. Variable importance in projection (VIP) plot for winter $DCFH_m$ PLSR model (top 50 features only).

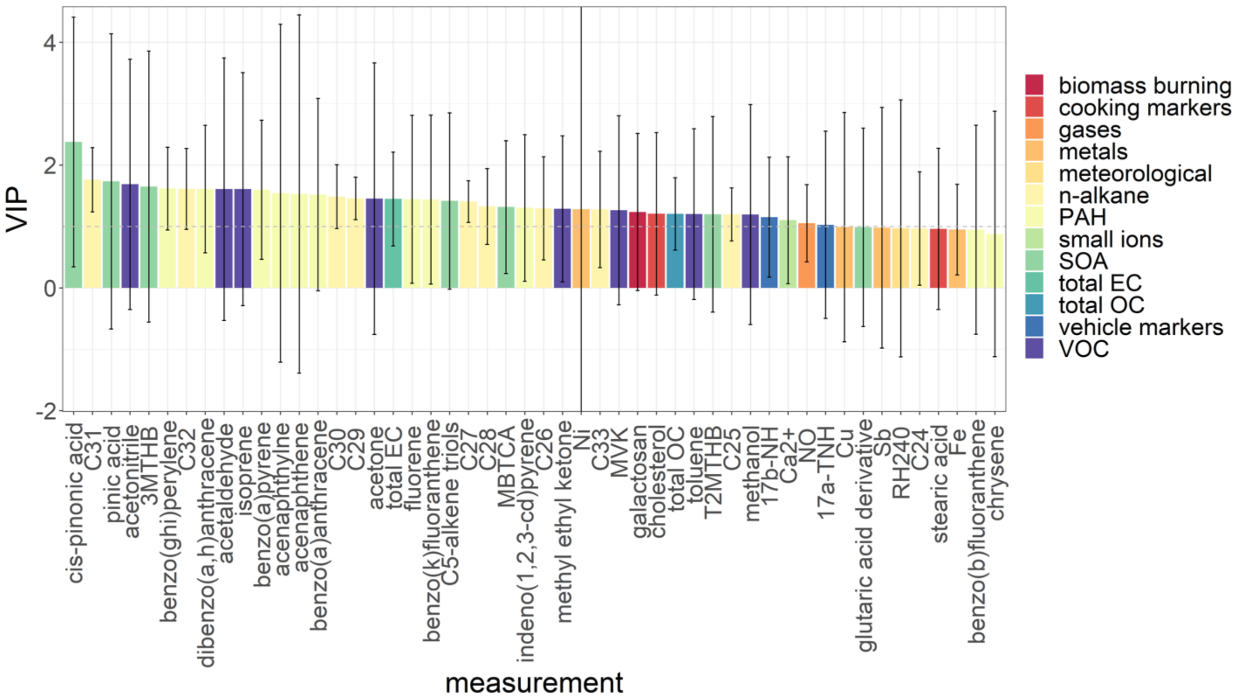

Figure S24. Variable importance in projection (VIP) plot for summer $DCFH_m$ PLSR model (top 50 features only).

Section S10: SPECIEUROPE database search methods

A combination of literature search and the SPECIEUROPE database (Pernigotti et al., 2016) was used to derive subsets of individual measurements for multiple linear regression modelling of source attributions. The aim of this analysis was to putatively identify the influence of known PM sources on the OP assay responses. The same panel of measurements was used for both mass-normalised and volume-normalised data model derivation. The SPECIEUROPE database was downloaded in csv format from https://source-apportionment.jrc.ec.europa.eu/Specieurope/profiles.aspx?source=999 (accessed on 25 May 2020) and imported into R for manipulation. All database sampling locations and dates were included, as any relevant components of a source type were potentially of interest, and single components (e.g. potassium) could be included across multiple source types (e.g. cement, salt, biomass burning, exhaust). The SPECIATE database (Simon et al., 2010) (https://www.epa.gov/sites/production/files/2020-07/speciate_5.1_0.zip) was considered for use, but is very extensive, making database investigation and search term collation less straightforward.

The source categories were also derived from common categories observed in the literature. Soil was considered for a separate category; however, insufficient information was available as to whether this would be a relevant influence on the urban sampling site, and the models produced were poor, and thus this category was not pursued further. To obtain the broad category subsets, a list of search terms was manually constructed using the "Name" and "Specie" columns from the database, which both had to be manually relabelled to ensure exact match of search words (mainly due to spelling inconsistencies). The search terms used for the "Name" column included (in no particular order): "brake", "car", "wood", "fire", "fireplace", "fuel", "combustion", "coal", "dust", "vehicle", "exhaust", "burn" and/or "burning", "power", "urban", "suburban", "road", "taxi", "tire" and/or "tyre", "traffic". To simplify subsets, the following terms were excluded, which were related mainly to agricultural and heavy/light industrial processes: "aluminium", "asphalt", "bronze", "CaCl2", "ceramic", "cruise", "crustal", "fertilizer", "frit", "harbour", "industry" and/or "industrial", "lignite", "lime", "metal", "mill", "nitrate", "olive", "ore", "pellet", "petrochem*", "phosphate", "plant", "poor state of pavement", "production", "rock", "salt", "ship", "slag", "steel", "tile", "tobacco", "works". All entries containing any of these terms in the "Name" field were removed from further analysis. Of the remaining entries, compounds in the "Specie" field were then relabelled to match labelling of the collated APHH dataset columns, and 51 compounds could be matched between the APHH dataset and the reduced SPECIEUROPE database.

The filtered entries were then assigned manually to match each of the six specified source categories. "Vehicle emissions" included all Name entries containing "brake", "car", "fuel", "combustion", "vehicle", "exhaust", "road", "taxi", "tire" and/or "tyre", "traffic". "Biomass burning" included "wood", "fire", all terms including "fireplace", "fuel", "combustion", "burn" and/or "burning". "Coal/fossil fuel combustion", included all terms containing "boiler", "fireplace", "fuel", "combustion", "burn" and/or "burning", "power". "Dust" included most Name entries containing "dust", and also included "ammonium nitrate (secondary)", "ammonium sulfate (secondary)", "salt marine", "soil dust", "NaCl", "CaCl2", "MgCl2", and "Mix of NaCl and CaCl2". Both "cooking markers" and "biogenic SOA" associated categories (e.g. categories relating to "soil" and "burning leaves" were deemed to be too broad and not representative of biogenic SOA due to the overlap with dust and combustion respectively; no cooking categories are present in the database) were derived purely from literature sources as they are poorly represented in the SPECIEUROPE database. A full breakdown of which measurements were included in the final model panel for each source category is given in **Table S13**, with reasons for feature non-selection and literature sources used.

The final list of subsets was then again examined manually, and compounds which were less representative of the source category removed. Any features missing more than 33% of the season measurements were excluded after the initial round of modelling if replacement by the median was observed to exert a strong influence on the MLR model residuals. Residuals were examined for bias with respect to sample order and distribution, and most sample order bias was related to missing measurements, which tended to be at the beginning and end of each season. All models were visualised with plots (example in **Figure S28**), which gave the predicted vs. actual OP assay response values (error

bars indicate variability of the predicted response over 500 rounds of bootstrapped cross-validation of the model), residuals plotted with respect to run order, the variability of feature coefficients through model cross-validation, the stacked concentration data over the season sampling period, and kernel density distributions of residuals. Final models for the mass-normalised data are provided in **Tables S14-S21**. All concentrations were expressed as μg/μg or μg/m^3 and converted from ng where appropriate.

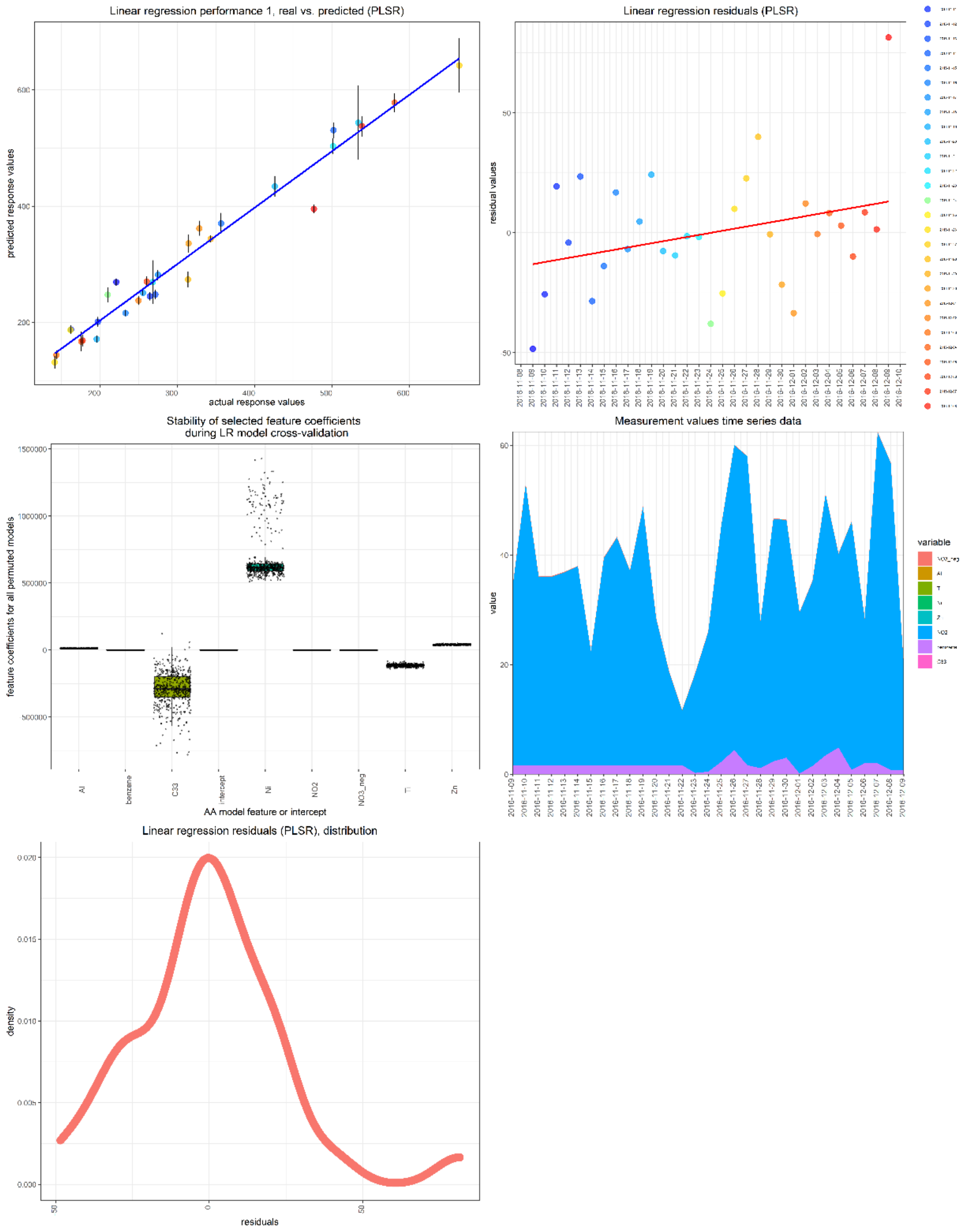

Figure S25. Example plot for MLR model (winter AA_m model).

Table S13. Source category assignment of individual measured PM components used to derive MLR models, from both SPECIEUROPE categories and literature sources. 1 indicates inclusion in the model; 0 indicates exclusion.

measurement	category	vehicle emissions	biomass burning	coal/ fossil fuel combustion	cooking markers	dust	biogenic SOA	notes
**total OC**	total carbon	-	-	-	-	-	-	Not included due to overlap with multiple separate measurements
**total EC**	total carbon	**1** (Cao et al., 2004; Yang et al., 2005; Zhang et al., 2015)	**1** (Ji et al., 2016; Zhang et al., 2015)	**1** (Zhang et al., 2015)	0	0	0	
**K^+**	small ions	**1**	**1** (Duan et al., 2004; Yu et al., 2018)	**1**	0	**1** (Liu et al., 2014a)	0	Included for vehicle emissions and fossil fuel combustion to test differences from biomass burning
**Na^+**	small ions	**1** (Zíková et al., 2016)	0	0	0	**1** (Liu et al., 2014a)	0	
**Ca^{2+}**	small ions	**1**	0	0	0	**1** (Huang et al., 2017; Liu et al., 2014a; Yu et al., 2019b)	0	
**NH_4^+**	small ions	-	-	-	-	-	-	Not included as influences multiple chemical processes
**NO_3^-**	small ions	**1** (Chen et al., 2014; Zíková et al., 2016)	0	0	0	0	**1** (Fry et al., 2014; Wang et al., 2018)	
**SO_4^{2-}**	small ions	0	0	**1** (Ianniello et al., 2011)	0	**1** (Liu et al., 2014a, 2005)	**1** (Fry et al., 2014; Wang et al., 2018)	
**Cl^-**	small ions	0	0	**1** (Chen et al., 2014; Ianniello et al., 2011; Yu et al., 2019b)	0	**1** (Ianniello et al., 2011)	0	
**Al**	metals	**1**	0	0	0	**1**	0	
**Ti**	metals	**1**	0	0	0	**1**	0	
**V**	metals	**1**	0	0	0	0	0	

**Cr**	metals	**1**	0	0	0	0	0	
**Mn**	metals	**1**	0	0	0	**1** (Yu et al., 2019b)	0	
**Fe**	metals	**1** (Zíková et al., 2016)	0	0	0	**1** (Liu et al., 2014a; Yu et al., 2019b)	0	
**Co**	metals	**1**	0	0	0	0	0	
**Ni**	metals	**1**	0	0	0	0	0	
**Cu**	metals	**1** (Liu et al., 2014b)	**1**	**1**	0	0	0	
**Zn**	metals	**1** (Liu et al., 2014b; Zíková et al., 2016)	0	**1** (Zíková et al., 2016)	0	**1**	0	
**Cd**	metals	**1**	0	**1**	0	0	0	
**Sb**	metals	**1**	0	**1**	0	0	0	
**Ba**	metals	**1**	0	**1**	0	0	0	
**Pb**	metals	**1**	0	**1** (Liu et al., 2014a; Zíková et al., 2016)	0	0	0	
**galactosan**	biomass burning	0	1	0	0	0	0	
**mannosan**	biomass burning	0	1	0	0	0	0	
**levoglucosan**	biomass burning	0	**1** (Zhang et al., 2008)	0	0	0	0	
**ORG**	AMS factors	0	0	0	0	0	0	
**MOOOA**	AMS factors	-	-	-	-	-	-	Not included, composite measurements
**LOOOA**	AMS factors	-	-	-	-	-	-	
**O_3**	gases	**1** (Duan et al., 2008)	**1**	0	0	0	**1** (Ghirardo et al., 2016)	
**CO**	gases	**1** (Duan et al., 2008)	**1** (Zhang et al., 2017a)	**1** (Zhang et al., 2017a)	**1**	0	**1**	
**NO**	gases	**1** (Du et al., 2012)	0	0	0	0	0	
**NO_2**	gases	**1** (Du et al., 2012)	0	0	0	0	0	
**NO_y**	gases	1 (Du et al., 2012; Duan et al., 2008)	0	0	0	0	0	

**SO_2**	gases	0	0	**1** (Ji et al., 2016)	0	0	0	
**RH8**	meteo	-	-	-	-	-	-	Not included as influences multiple processes, difficult to interpret
**RH120**	meteo	-	-	-	-	-	-	
**RH240**	meteo	-	-	-	-	-	-	
**T8**	meteo	-	-	-	-	-	-	
**T120**	meteo	-	-	-	-	-	-	
**T240**	meteo	-	-	-	-	-	-	
**methanol**	VOC	-	-	-	-	-	-	Missing multiple measurements, not included as confers strong bias / instability on models and residuals
**acetonitrile**	VOC	-	-	-	-	-	-	
**acetaldehyde**	VOC	-	-	-	-	-	-	
**acrolein**	VOC	-	-	-	-	-	-	
**acetone**	VOC	-	-	-	-	-	-	
**isoprene**	VOC	0	0	0	0	0	**1** (Duan et al., 2008; Ghirardo et al., 2016)	Missing multiple measurements
**methyl vinyl ketone /methacrolein**	VOC	0	0	0	0	0	**1** (Pang et al., 2009)	
**methyl ethyl ketone**	VOC	0	0	0	0	0	**1** (Shao et al., 2009)	
**benzene**	VOC	**1** (Duan et al., 2008)	0	0	0	0	0	
**toluene**	VOC	**1** (Duan et al., 2008)	0	0	0	0	0	
**C2-benzenes**	VOC	**1** (Squires et al., 2020)	0	0	0	0	0	Composite measurements but not overlapping with other measured components
**C3-benzenes**	VOC	**1** (Squires et al., 2020)	0	0	0	0	0	
JO^1D	photolysis	-	-	-	-	-	-	Not included as influences multiple chemical processes, difficult to interpret exact influence
JNO_2	photolysis	-	-	-	-	-	-	
naphthalene	PAH	-	-	-	-	-	-	Missing multiple measurements, not included as confers strong bias / instability
acenaphthylne	PAH	-	-	-	-	-	-	
acenaphthene	PAH	-	-	-	-	-	-	
fluorene	PAH	-	-	-	-	-	-	
phenanthrene	PAH	-	-	-	-	-	-	
fluoranthene	PAH	-	-	-	-	-	-	
pyrene	PAH	-	-	-	-	-	-	

benzo(a)-anthracene	PAH	-	-	-	-	-	-	on models and residuals
chrysene	PAH	-	-	-	-	-	-	
benzo(b)-fluoranthene	PAH	-	-	-	-	-	-	
benzo(k)-fluoranthene	PAH	-	-	-	-	-	-	
benzo(a)-pyrene	PAH	-	-	-	-	-	-	
indeno(1,2,3-cd)pyrene	PAH	-	-	-	-	-	-	
dibenzo(a,h)-anthracene	PAH	-	-	-	-	-	-	
benzo(ghi)-perylene	PAH	-	-	-	-	-	-	
**C24**	n-alkane	**1**	0	**1**	0	0	0	Ref. for all n-alkanes: (Li et al., 2013; Zhang et al., 2017b)
**C25**	n-alkane	**1**	0	**1**	0	0	0	
**C26**	n-alkane	**1**	0	**1**	0	0	0	
**C27**	n-alkane	**1**	0	**1**	0	0	0	
**C28**	n-alkane	**1**	0	**1**	0	0	0	
**C29**	n-alkane	**1**	0	**1**	0	0	0	
**C30**	n-alkane	**1**	0	**1**	0	0	0	
**C31**	n-alkane	**1**	0	**1**	0	0	0	
**C32**	n-alkane	**1**	0	**1**	0	0	0	
**C33**	n-alkane	**1**	0	**1**	0	0	0	
**C34**	n-alkane	**1**	0	**1**	0	0	0	
OH	gas radical	-	-	-	-	-	-	Not included, influences multiple aerosol chemistries
HO_2	gas radical	-	-	-	-	-	-	
RO_2	gas radical	-	-	-	-	-	-	
**palmitic acid**	cooking markers	0	0	0	**1** (Li et al., 2013)	0	0	
**stearic acid**	cooking markers	0	0	0	**1** (Li et al., 2013)	0	0	
**cholesterol**	cooking markers	0	0	0	**1** (He et al., 2006)	0	0	
**17a(H)-22,29,30-trisnorhopane (C27a)**	vehicle markers	**1** (He et al., 2006)	0	0	0	0	0	
**17b(H),21a(H)-norhopane (C30ba)**	vehicle markers	**1** (He et al., 2006)	0	0	0	0	0	
**2-methyl-threitol**	SOA	0	0	0	0	0	**1** (Ding et al., 2012)	
**2-methyl-erythritol**	SOA	0	0	0	0	0	**1** (Ding et al., 2012)	
**2-methyl-glyceric acid**	SOA	0	0	0	0	0	**1** (Ding et al., 2012)	
**cis-2-methyl-1,3,4-trihydroxy-1-butene**	SOA	0	0	0	0	0	**1**	

**3-methyl-2,3,4-trihydroxy-1-butene**	SOA	0	0	0	0	0	**1**	
**trans-2-methyl-1,3,4-trihydroxy-1-butene**	SOA	0	0	0	0	0	**1**	
C5-alkene triols	SOA	-	-	-	-	-	-	Not included, composite measurements partially measured separately
2-methyltetrols	SOA	-	-	-	-	-	-	
**3-hydroxy-glutaric acid**	SOA	0	0	0	0	0	**1** (Ding et al., 2012)	
**cis-pinonic acid**	SOA	0	0	0	0	0	**1** (Ding et al., 2012)	
**pinic acid**	SOA	0	0	0	0	0	**1** (Ding et al., 2012)	
**3-methyl-1,2,3-butanetricarboxylic acid**	SOA	0	0	0	0	0	**1** (Ding et al., 2012)	
**β-caryophyllinic acid**	SOA	0	0	0	0	0	**1** (Ding et al., 2012)	
glutaric acid derivative	SOA	-	-	-	-	-	-	Not included, composite measurement
**3-acetyl-pentanedioic acid**	SOA	0	0	0	0	0	**1**	
**3-acetyl-hexanedioic acid**	SOA	0	0	0	0	0	**1**	
**3-isopropyl-pentanedioic acid**	SOA	0	0	0	0	0	**1**	
**2,3-dihydroxy-4-oxopentanoic acid**	SOA	0	0	0	0	0	**1** (Ding et al., 2012)	

Section S11: Multiple linear regression model parameters (mass-normalised only)

Winter OP_m EPR

Table S14. MLR model parameters for winter EPR source models. Coefficient variation represents variance in cross-validated models through 500 fully random permutations with bootstrapping. Values are (mean(min,max)) of the permuted model term coefficients.

	vehicle emissions	biomass burning	coal / fossil fuel	cooking markers	dust	biogenic SOA
**model terms**	total EC + Ba + NO + benzene + C27 + C31 + C32 + C33	total EC + K^+ + mannosan + levoglucosan	total EC + SO_4^{2-} + Cu + Cd + Ba + C27 + C30 + C31	CO + palmitic acid + stearic acid + cholesterol	K^+ + Cl^- + Al + Ti	NO_3^- + O3 + CO + methyl ethyl ketone + cis-2-methyl-1,3,4-trihydroxy-1-butene + MBTCA + 3-acetylpentanedioic acid + 2,3-dihydroxy-4-oxopentanoic acid
**residuals deviance min**	-1.51E+03	-2.24E+03	-1.49E+03	-2.49E+03	-2.52E+03	-2.26E+03
**residuals deviance median**	-6.41E+00	2.30E+02	1.50E+02	-6.80E+01	-1.16E+02	1.20E+02
**residuals deviance mean**	-2.32E-12	-1.41E-12	-2.58E-12	-1.76E-12	7.92E-13	-1.55E-12
**residuals deviance max**	9.31E+02	2.58E+03	1.09E+03	2.78E+03	2.59E+03	1.50E+03
**null deviance**	6.59E+07	6.59E+07	6.59E+07	6.59E+07	6.59E+07	6.59E+07
**residual deviance**	8.23E+06	3.88E+07	1.08E+07	5.33E+07	5.09E+07	2.94E+07
**R^2**	**0.88**	0.41	**0.84**	0.19	0.23	0.55
**intercept coefficient**	6.93E+03	4.05E+03	1.66E+03	8.19E+03	5.90E+03	1.13E+04
**coefficient 1**	6.82E+04	5.71E+04	1.25E+05	-6.05E-01	9.14E+04	1.11E+04
**coefficient 2**	-6.46E+06	1.23E+05	1.93E+04	1.71E+04	2.14E+04	-2.40E+02
**coefficient 3**	2.25E+01	-2.24E+06	-1.64E+06	-2.99E+04	-6.25E+04	-1.69E+00
**coefficient 4**	-8.38E+02	1.99E+05	5.43E+06	1.16E+07	-1.58E+05	-6.17E+03
**coefficient 5**	-6.31E+06	-	-5.33E+06	-	-	1.27E+09
**coefficient 6**	1.20E+07	-	-3.16E+06	-	-	1.50E+08
**coefficient 7**	-1.34E+07	-	-2.03E+07	-	-	9.43E+08
**coefficient 8**	2.44E+07	-	1.79E+07	-	-	-1.46E+08
**intercept coeff. variation**	6.99e+03 (6.26e+03, 7.99e+03)	4.09e+03 (2.54e+03, 5.53e+03)	1.57e+03 (-4.51e+02, 3.21e+03)	8.23e+03 (7.51e+03, 9.29e+03)	5.90e+03 (3.95e+03, 7.78e+03)	1.14e+04 (6.61e+03, 1.42e+04)
**coeff. variation 1**	6.95e+04 (4.74e+04, 9.98e+04)	5.74e+04 (2.00e+04, 9.83e+04)	1.26e+05 (9.67e+04, 1.52e+05)	-6.05e-01 (-1.11e+00, 2.72e-01)	8.66e+04 (-1.17e+04, 1.68e+05)	1.10e+04 (-7.19e+03, 2.34e+04)
**coeff. variation 2**	-6.54e+06 (-8.95e+06, -5.29e+06)	1.23e+05 (4.05e+04, 2.17e+05)	1.99e+04 (1.04e+04, 3.88e+04)	2.98e+04 (-3.45e+05, 4.78e+05)	2.23e+04 (-1.63e+03, 5.39e+04)	-2.45e+02 (-3.97e+02, -9.78e+01)
**coeff. variation 3**	2.21e+01 (1.20e+01, 3.20e+01)	-1.64e+06 (-9.78e+06, 1.12e+07)	-1.42e+06 (-4.60e+06, 5.44e+06)	-6.41e+04 (-9.24e+05, 4.25e+05)	-5.93e+04 (-1.48e+05, 2.25e+05)	-1.71e+00 (-2.80e+00, -4.07e-01)
**coeff. variation 4**	-8.51e+02 (-1.33e+03, -5.78e+02)	1.22e+05 (-1.40e+06, 1.19e+06)	5.48e+06 (2.20e+06, 8.67e+06)	1.13e+07 (-1.81e+06, 4.24e+07)	-1.50e+05 (-5.25e+05, 1.03e+06)	-5.97e+03 (-1.14e+04, 2.23e+02)

**coeff. variation 5**	-6.27e+06 (-7.95e+06, -4.46e+06)	-	-5.52e+06 (-8.44e+06, -4.12e+06)	-	-	1.28e+09 (6.13e+06, 2.76e+09)
**coeff. variation 6**	1.23e+07 (7.97e+06, 1.92e+07)	-	-3.21e+06 (-5.71e+06, -8.09e+05)	-	-	1.50e+08 (-2.32e+07, 2.59e+08)
**coeff. variation 7**	-1.49e+07 (-3.52e+07, -4.89e+06)	-	-2.03e+07 (-3.65e+07, -1.13e+07)	-	-	9.09e+08 (-5.59e+08, 2.68e+09)
**coeff. variation 8**	2.39e+07 (1.06e+07, 3.29e+07)	-	1.79e+07 (1.01e+07, 2.88e+07)	-	-	-1.47e+08 (-2.88e+08, 4.38e+07)
**intercept std error**	6.20E+02	9.07E+02	8.89E+02	7.23E+02	1.02E+03	1.90E+03
**std error 1**	1.18E+04	1.98E+04	1.67E+04	4.05E-01	5.23E+04	7.58E+03
**std error 2**	7.65E+05	4.27E+04	6.07E+03	1.98E+05	1.53E+04	7.76E+01
**std error 3**	5.29E+00	1.85E+06	4.65E+05	2.46E+05	5.86E+04	5.97E-01
**std error 4**	1.45E+02	1.97E+05	1.42E+06	6.95E+06	1.57E+05	2.34E+03
**std error 5**	8.09E+05	-	9.20E+05	-	-	6.17E+08
**std error 6**	2.45E+06	-	9.81E+05	-	-	5.31E+07
**std error 7**	3.66E+06	-	4.29E+06	-	-	5.45E+08
**std error 8**	5.32E+06	-	3.95E+06	-	-	6.22E+07
**intercept p-value**	**1.52E-10**	**1.38E-04**	7.49E-02	**1.48E-11**	**4.18E-06**	**5.55E-06**
**p-value 1**	**8.36E-06**	7.88E-03	**1.81E-07**	1.47E-01	9.25E-02	1.56E-01
**p-value 2**	**2.36E-08**	7.72E-03	**4.34E-03**	9.32E-01	1.74E-01	**5.25E-03**
**p-value 3**	**3.26E-04**	2.38E-01	**1.88E-03**	9.04E-01	2.96E-01	**9.65E-03**
**p-value 4**	**8.34E-06**	3.22E-01	**9.54E-04**	1.07E-01	3.23E-01	1.53E-02
**p-value 5**	**9.03E-08**	-	**7.82E-06**	-	-	5.12E-02
**p-value 6**	**6.94E-05**	-	**3.92E-03**	-	-	1.00E-02
**p-value 7**	**1.42E-03**	-	**9.92E-05**	-	-	9.73E-02
**p-value 8**	**1.47E-04**	-	**1.64E-04**	-	-	2.85E-02

Summer OP_m EPR

Table S15. MLR model parameters for summer EPR source models**.**

	**vehicle emissions**	**biomass burning**	**coal / fossil fuel**	**cooking markers**	**dust**	**biogenic SOA**
**model terms**	total EC + Ni + CO + NO + NOy + C26 + C27 + C30	total EC + K^+ + galactosan + O_3	total EC + SO_4^{2-} + Zn + Pb + SO_2 + C29	CO + palmitic acid + stearic acid + cholesterol	K+ + Na+ + Cl- + Al	SO_4^{2-} + O3 + 2-methylglyceric acid + MBTCA
**residuals deviance min**	-2.60E+03	-3.73E+03	-4.09E+03	-3.79E+03	-2.93E+03	-4.88E+03
**residuals deviance median**	-1.20E+02	-7.73E+01	-1.61E+02	-2.46E+02	-3.39E+02	-2.11E+02
**residuals deviance mean**	5.66E-12	-5.52E-14	3.49E-12	-3.67E-12	9.51E-13	5.52E-14
**residuals deviance max**	2.47E+03	4.95E+03	3.35E+03	6.12E+03	5.74E+03	5.10E+03
**null deviance**	1.80E+08	1.80E+08	1.80E+08	1.80E+08	1.80E+08	1.80E+08
**residual deviance**	5.08E+07	1.28E+08	7.99E+07	1.61E+08	1.39E+08	1.17E+08

**R²**	0.72	0.29	0.56	0.11	0.23	0.35
**intercept coefficient**	5.82E+03	1.50E+03	2.74E+03	6.03E+03	4.10E+03	-1.83E+03
**coefficient 1**	-2.79E+05	2.39E+04	-1.85E+05	2.67E-01	1.66E+05	7.66E+03
**coefficient 2**	2.70E+07	1.40E+05	7.97E+03	4.40E+06	2.02E+05	5.29E+01
**coefficient 3**	8.54E+00	-2.02E+07	5.77E+05	-9.85E+06	-7.49E+04	1.71E+07
**coefficient 4**	3.99E+02	4.42E+01	-2.50E+06	5.44E+06	-1.30E+05	-7.83E+06
**coefficient 5**	-2.09E+02	-	2.88E+02	-	-	-
**coefficient 6**	-1.77E+08	-	3.73E+07	-	-	-
**coefficient 7**	6.68E+07	-	-	-	-	-
**coefficient 8**	2.12E+08	-	-	-	-	-
**intercept coeff. variation**	5.91e+03 (3.20e+03, 8.70e+03)	1.51e+03 (-3.86e+03, 3.97e+03)	5.17e+01 (-3.15e+03, 4.46e+03)	6.40e+03 (2.58e+03, 1.07e+04)	3.99e+03 (1.15e+03, 5.82e+03)	2.23e+03 (-6.93e+02, 5.15e+03)
**coeff. variation 1**	-2.80e+05 (-3.89e+05, -1.85e+05)	2.45e+04 (-2.14e+04, 1.07e+05)	-1.47e+05 (-2.60e+05, -7.37e+04)	7.52e-03 (-5.17e+00, 4.65e+00)	2.24e+05 (1.81e+04, 4.43e+05)	3.72e+01 (5.55e-02, 6.67e+01)
**coeff. variation 2**	2.77e+07 (1.03e+07, 5.55e+07)	1.43e+05 (3.12e+04, 3.16e+05)	1.83e+04 (-1.46e+03, 4.06e+04)	2.48e+06 (-1.89e+06, 1.18e+07)	7.48e+04 (-4.28e+05, 4.45e+05)	2.13e+07 (5.27e+06, 4.09e+07)
**coeff. variation 3**	8.39e+00 (5.35e+00, 1.19e+01)	-2.14e+07 (-6.56e+07, -5.13e+06)	6.12e+05 (8.79e+04, 1.19e+06)	-7.01e+06 (-2.60e+07, 3.21e+06)	-1.13e+05 (-3.99e+05, 5.16e+04)	5.71e+08 (-1.13e+09, 1.16e+09)
**coeff. variation 4**	3.98e+02 (1.41e+02, 6.46e+02)	4.40e+01 (5.21e+00, 1.00e+02)	-2.45e+06 (-3.92e+06, -4.04e+05)	3.46e+06 (-8.57e+07, 8.04e+07)	-1.48e+06 (-4.00e+06, 4.94e+05)	-8.04e+08 (-1.50e+09, 2.30e+08)
**coeff. variation 5**	-2.10e+02 (-3.17e+02, -1.17e+02)	-	3.80e+02 (1.26e+02, 6.43e+02)	-	-	2.81e+07 (-6.68e+06, 5.87e+07)
**coeff. variation 6**	-1.75e+08 (-2.59e+08, -1.04e+08)	-	3.39e+07 (2.17e+07, 5.11e+07)	-	-	-
**coeff. variation 7**	6.68e+07 (4.52e+07, 9.11e+07)	-	-	-	-	-
**coeff. variation 8**	2.10e+08 (5.16e+07, 3.25e+08)	-	-	-	-	-
**intercept std error**	1432.08	2141.16	1.49E+03	2123.54	981.42	2054.73
**std error 1**	4.94E+04	3.37E+04	37435.18	2.76	8.63E+04	5101.29
**std error 2**	7.00E+06	8.08E+04	4601.03	3.77E+06	1.69E+05	19.25
**std error 3**	2.14	1.85E+07	3.50E+05	6.93E+06	5.89E+04	5.88E+06
**std error 4**	142.36	22.54	1.17E+06	5.43E+07	7.37E+04	6.02E+06
**std error 5**	53.56	-	174.27	-	-	-
**std error 6**	3.56E+07	-	7.28E+06	-	-	-
**std error 7**	1.02E+07	-	-	-	-	-
**std error 8**	5.27E+07	-	-	-	-	-
**intercept p-value**	**0.0004**	0.49	0.08	**0.01**	0.00	0.38
**p-value 1**	**0.0001**	0.48	**4.04E-05**	0.92	0.06	0.14
**p-value 2**	**0.0007**	0.09	0.10	0.25	0.24	0.01
**p-value 3**	**0.0005**	0.29	0.11	0.17	0.21	0.01
**p-value 4**	**0.01**	0.06	**0.04**	0.92	0.09	0.20
**p-value 5**	**0.0007**	-	0.11	-	-	-
**p-value 6**	**4.58E-05**	-	**2.39E-05**	-	-	-
**p-value 7**	**9.57E-07**	-	-	-	-	-
**p-value 8**	**4.95E-04**	-	-	-	-	-

Winter OP_m AA

Table S16. MLR model parameters for winter AA source models.

	vehicle emissions	biomass burning	coal / fossil fuel	cooking markers	dust	biogenic SOA
**model terms**	Ca^{2+} + NO_3^- + Cr + Co + Ni + Zn + O_3 + C33	Cu + mannosan + levoglucosan + O_3	SO_4^{2-} + Cl- + Cu + Zn + Cd + Pb + C30 + C32	CO + palmitic acid + stearic acid + cholesterol	Ca^{2+} + Cl^- + Al + Ti + Mn + Fe + Zn	NO3_neg + methyl vinyl ketone/ methacrolein + methyl ethyl ketone + 2-methylglyceric acid + trans-2-methyl-1,3,4-trihydroxy-1-butene + cis-pinonic acid` + MTBCA + 3-acetylhexanedioic acid
**residuals deviance min**	-9.02E+01	-1.44E+02	-1.64E+02	-1.97E+02	-1.15E+02	-8.77E+01
**residuals deviance median**	-1.03E+00	-2.18E+01	7.85E+00	-1.55E+01	1.20E+01	-2.41E+00
**residuals deviance mean**	6.33E-14	5.78E-14	2.70E-14	9.44E-14	8.34E-14	-9.35E-14
**residuals deviance max**	5.65E+01	2.97E+02	9.32E+01	2.37E+02	1.36E+02	9.10E+01
**null deviance**	8.30E+05	8.30E+05	8.30E+05	8.30E+05	8.30E+05	8.30E+05
**residual deviance**	4.49E+04	4.27E+05	1.01E+05	2.84E+05	1.01E+05	4.32E+04
**R^2**	**0.95**	0.49	**0.88**	0.66	**0.88**	**0.95**
**intercept coefficient**	2.05E+02	1.16E+02	2.80E+01	2.93E+02	-6.17E+01	3.94E+02
**coefficient 1**	1.78E+04	1.09E+05	-9.86E+02	-5.61E-02	2.23E+04	-2.09E+03
**coefficient 2**	-1.22E+03	-2.79E+05	6.26E+03	3.95E+04	4.69E+03	-5.53E+01
**coefficient 3**	4.25E+05	4.35E+04	-2.03E+05	-4.50E+04	5.82E+03	3.42E+02
**coefficient 4**	-3.63E+07	1.28E+01	4.87E+04	9.51E+05	-6.17E+04	1.76E+06
**coefficient 5**	1.62E+06	-	2.93E+05	-	2.08E+05	8.02E+06
**coefficient 6**	1.73E+04	-	-1.36E+05	-	-1.46E+04	1.02E+06
**coefficient 7**	7.46E+00	-	-9.48E+05	-	1.54E+04	1.63E+06
**coefficient 8**	4.65E+05	-	1.53E+06	-	-	-6.23E+07
**intercept coeff. variation**	2.01e+02 (9.30e+01, 3.09e+02)	1.14e+02 (-9.76e+00, 2.04e+02)	3.40e+01 (-1.06e+02, 1.94e+02)	2.91e+02 (1.98e+02, 3.69e+02)	-5.57e+01 (-1.55e+02, 4.29e+01)	3.95e+02 (3.03e+02, 5.04e+02)
**coeff. variation 1**	1.75e+04 (2.17e+03, 3.17e+04)	1.81e+05 (-1.67e+04, 8.59e+05)	-9.83e+02 (-2.36e+03, 1.10e+02)	-5.64e-02 (-9.13e-02, -9.72e-03)	2.22e+04 (8.51e+03, 4.06e+04)	-2.09e+03 (-2.72e+03, -1.46e+03)
**coeff. variation 2**	-1.21e+03 (-1.97e+03, -5.41e+02)	-1.88e+05 (-5.21e+05, 1.05e+06)	5.89e+03 (2.67e+03, 8.42e+03)	3.68e+04 (-8.66e+02, 7.98e+04)	4.23e+03 (2.91e+02, 6.89e+03)	-5.66e+01 (-9.70e+01, -2.21e+01)
**coeff. variation 3**	4.21e+05 (2.35e+05, 7.77e+05)	3.22e+04 (-1.16e+05, 6.15e+04)	-1.59e+05 (-3.54e+05, 2.57e+05)	-3.90e+04 (-9.48e+04, 2.07e+04)	5.95e+03 (-3.73e+03, 1.46e+04)	3.48e+02 (1.04e+02, 5.05e+02)
**coeff.**	-3.59e+07	1.21e+01	5.06e+04	9.67e+05	-5.48e+04	1.71e+06

**variation 4**	(-5.59e+07, -7.59e+06)	(8.17e-01, 1.92e+01)	(1.58e+03, 7.61e+04)	(-3.82e+05, 3.42e+06)	(-1.61e+05, 1.85e+04)	(-4.30e+05, 3.04e+06)
**coeff. variation 5**	1.66e+06 (6.82e+05, 4.58e+06)	-	2.39e+05 (-1.92e+05, 5.62e+05)	-	2.10e+05 (-3.66e+05, 6.32e+05)	7.67e+06 (2.29e+06, 1.31e+07)
**coeff. variation 6**	1.82e+04 (-1.15e+04, 3.14e+04)	-	-1.28e+05 (-1.89e+05, 4.82e+04)	-	-1.37e+04 (-3.48e+04, 1.39e+04)	1.05e+06 (5.71e+05, 1.85e+06)
**coeff. variation 7**	7.42e+00 (3.64e+00, 1.01e+01)	-	-9.65e+05 (-2.48e+06, 6.62e+05)	-	1.60e+04 (-4.31e+04, 4.61e+04)	1.80e+06 (4.48e+05, 6.07e+06)
**coeff. variation 8**	4.21e+05 (-2.28e+04, 8.85e+05)	-	1.55e+06 (-9.23e+05, 4.71e+06)	-	-	-6.20e+07 (-8.43e+07, -4.37e+07)
**intercept std error**	4.39E+01	5.08E+01	5.35E+01	5.28E+01	4.33E+01	5.43E+01
**std error 1**	5.60E+03	6.64E+04	5.31E+02	2.95E-02	6.47E+03	3.14E+02
**std error 2**	3.06E+02	2.14E+05	1.39E+03	1.45E+04	1.01E+03	1.80E+01
**std error 3**	6.66E+04	2.14E+04	5.49E+04	1.79E+04	2.87E+03	1.04E+02
**std error 4**	7.40E+06	4.70E+00	1.19E+04	5.07E+05	1.59E+04	7.57E+05
**std error 5**	6.20E+05	-	1.67E+05	-	1.11E+05	2.66E+06
**std error 6**	4.03E+03	-	3.74E+04	-	8.60E+03	3.76E+05
**std error 7**	1.78E+00	-	4.07E+05	-	7.10E+03	6.58E+05
**std error 8**	1.76E+05	-	7.24E+05	-	-	1.39E+07
**intercept p-value**	**1.16E-04**	**0.03**	0.61	**7.86E-06**	0.17	**2.82E-07**
**p-value 1**	**4.25E-03**	0.11	0.08	0.07	**2.18E-03**	**1.05E-06**
**p-value 2**	**6.18E-04**	0.20	**1.73E-04**	**0.01**	**1.17E-04**	**0.01**
**p-value 3**	**2.02E-06**	0.05	**1.24E-03**	**0.02**	**0.05**	**3.41E-03**
**p-value 4**	**6.64E-05**	0.01	**4.65E-04**	0.07	**7.68E-04**	**0.03**
**p-value 5**	**0.02**	-	0.09	-	0.07	**0.01**
**p-value 6**	**3.01E-04**	-	**1.43E-03**	-	0.10	**0.01**
**p-value 7**	**3.77E-04**	-	**0.03**	-	**0.04**	**0.02**
**p-value 8**	**0.01**	-	**0.05**	-	-	**1.87E-04**

Summer OP_m AA

Table S17. MLR model parameters for summer AA source models**.**

	**vehicle emissions**	**biomass burning**	**coal / fossil fuel**	**cooking markers**	**dust**	**biogenic SOA**
**model terms**	total EC + NO_3^- + Ti + Fe + Zn + O3 + NO_2 + C33	total EC + mannosan + O_3 + CO	Zn + Cd + Ba + C24 + C26 + C27 + C30 + C31	CO + palmitic acid + stearic acid + cholesterol	Al + Ti + Fe + Zn	O_3 + CO + methyl ethyl ketone + 2-methylglyceric acid + 3-hydroxyglutaric acid + cis-pinonic acid + β-caryophyllinic acid + 2,3-dihydroxy-4-oxopentanoic acid
**residuals deviance min**	-9.07E+01	-1.02E+02	-1.12E+02	-1.64E+02	-1.11E+02	-7.37E+01
**residuals deviance median**	4.16E+00	-4.01E+00	4.01E+00	-7.39E+00	5.42E+00	-3.51E+00
**residuals deviance mean**	-1.90E-13	-1.16E-13	-1.07E-13	-3.70E-14	-1.38E-13	-1.69E-13
**residuals deviance max**	1.07E+02	9.60E+01	7.44E+01	1.10E+02	1.13E+02	9.45E+01
**null deviance**	1.76E+05	1.76E+05	1.76E+05	1.76E+05	1.76E+05	1.76E+05

**residual deviance**	4.84E+04	9.30E+04	6.87E+04	1.41E+05	9.38E+04	4.58E+04
**R^2**	**0.73**	0.47	0.61	0.20	0.47	**0.74**
**intercept coefficient**	6.43E+02	1.85E+02	1.88E+02	1.62E+02	1.91E+02	1.99E+02
**coefficient 1**	6.13E+02	1.85E+02	1.87E+02	1.69E+02	1.93E+02	2.03E+02
**coefficient 2**	-6.34E+03	1.21E+03	-3.81E+04	9.89E-03	-2.61E+03	-1.94
**coefficient 3**	-3.01E+02	4.46E+05	2.68E+05	-1.23E+05	-7.22E+04	0.09
**coefficient 4**	-1.29E+05	-1.46	-9.19E+04	3.30E+05	1.28E+04	-6.11E+01
**coefficient 5**	2.16E+04	0.09	2.03E+06	-9.59E+05	-2.93E+04	-2.08E+05
**coefficient 6**	-4.51E+04	-	-4.75E+06	-	-	-3.31E+06
**coefficient 7**	-3.01	-	8.33E+05	-	-	7.32E+05
**coefficient 8**	-6.41	-	7.49E+06	-	-	1.74E+06
**intercept coeff. variation**	6.13e+02 (5.32e+02, 7.31e+02)	1.82e+02 (4.40e+01, 2.60e+02)	1.83e+02 (7.99e+01, 2.71e+02)	1.63e+02 (-3.29e+01, 2.48e+02)	1.93e+02 (1.60e+02, 2.30e+02)	1.99e+02 (2.86e+01, 3.69e+02)
**coeff. variation 1**	-6.31e+03 (-8.61e+03, -3.08e+03)	1.17e+03 (-3.61e+02, 2.44e+03)	-3.90e+04 (-5.85e+04, -2.31e+04)	1.81e-02 (-9.21e-02, 2.78e-01)	-2.76e+03 (-9.37e+03, 9.92e+02)	-1.93e+00 (-2.95e+00, -8.56e-01)
**coeff. variation 2**	-3.09e+02 (-5.22e+02, -1.08e+02)	4.77e+05 (3.17e+05, 1.25e+06)	2.87e+05 (-1.63e+05, 6.02e+05)	-9.83e+04 (-2.79e+05, 2.96e+05)	-6.75e+04 (-1.85e+05, 9.23e+04)	9.43e-02 (-3.20e-03, 2.50e-01)
**coeff. variation 3**	-1.28e+05 (-2.23e+05, -2.86e+04)	-1.45e+00 (-2.27e+00, -6.51e-01)	-9.53e+04 (-1.66e+05, -2.22e+04)	2.96e+05 (-3.03e+05, 7.03e+05)	1.28e+04 (7.60e+03, 1.83e+04)	-6.07e+01 (-1.11e+02, -8.97e-01)
**coeff. variation 4**	2.15e+04 (1.44e+04, 2.80e+04)	9.50e-02 (2.23e-02, 2.54e-01)	2.16e+06 (1.40e+06, 4.82e+06)	-1.06e+06 (-6.76e+06, 1.09e+06)	-2.96e+04 (-4.29e+04, -1.97e+04)	-2.04e+05 (-4.34e+05, 3.60e+04)
**coeff. variation 5**	-4.58e+04 (-6.47e+04, -3.20e+04)	-	-4.81e+06 (-9.74e+06, -1.61e+06)	-	-	-3.30e+06 (-5.67e+06, 2.16e+06)
**coeff. variation 6**	-3.01e+00 (-3.66e+00, -2.24e+00)	-	7.94e+05 (-2.48e+05, 1.58e+06)	-	-	7.34e+05 (4.03e+05, 1.18e+06)
**coeff. variation 7**	-6.27e+00 (-1.01e+01, -3.97e+00)	-	7.51e+06 (3.54e+06, 1.18e+07)	-	-	1.74e+06 (1.13e+06, 2.63e+06)
**coeff. variation 8**	2.17e+06 (9.47e+05, 3.09e+06)	-	-5.42e+05 (-1.34e+06, 3.37e+05)	-	-	-
**intercept std error**	9.24E+01	5.80E+01	3.39E+01	6.36E+01	2.91E+01	6.86E+01
**std error 1**	1.89E+03	8.16E+02	9.96E+03	0.08	3.50E+03	0.51
**std error 2**	1.10E+02	1.96E+05	1.06E+05	7.10E+04	6.15E+04	0.06
**std error 3**	4.31E+04	0.66	4.06E+04	1.56E+05	2.87E+03	2.78E+01
**std error 4**	4.42E+03	0.07	7.56E+05	1.61E+06	9.25E+03	1.25E+05
**std error 5**	9.35E+03	-	1.45E+06	-	-	1.46E+06
**std error 6**	0.65	-	3.74E+05	-	-	1.94E+05
**std error 7**	1.89	-	2.15E+06	-	-	4.71E+05
**std error 8**	7.92E+05	-	3.26E+05	-	-	-
**intercept p-value**	**7.42E-07**	**3.48E-03**	**1.15E-05**	**1.31E-02**	**6.81E-07**	**0.01**
**p-value 1**	**2.64E-03**	0.15	**8.23E-04**	0.90	0.41	**6.31E-04**
**p-value 2**	**1.17E-02**	**0.03**	**1.86E-02**	0.10	0.38	0.19
**p-value 3**	**6.38E-03**	**0.04**	**0.03**	**0.04**	**3.04E-04**	**0.02**
**p-value 4**	**5.44E-05**	0.21	**1.29E-02**	0.56	**0.01**	**0.04**
**p-value 5**	**6.40E-05**	-	**3.18E-03**	-	-	0.10
**p-value 6**	**1.11E-04**	-	**0.04**	-	-	**4.93E-04**
**p-value 7**	**2.39E-03**	-	**1.92E-03**	-	-	0.19
**p-value 8**	**1.20E-02**	-	0.08	-	-	**4.55E-03**

Winter OP_m DTT

Table S18. MLR model parameters for winter DTT source models.

	vehicle emissions	biomass burning	coal / fossil fuel	cooking markers	dust	biogenic SOA
**model terms**	Ca^{2+} + Fe + Cu + Sb + Pb + CO + NO_y + 17b(H),21a(H)-norhopane (C30ba)	K^+ + Cu + mannosan + CO	Cl^- + Ba + Pb + SO_2 + C28 + C29 + C30 + C31	CO + palmitic acid + stearic acid + cholesterol	Ca^{2+} + Al + Ti + Mn	CO + isoprene + methyl vinyl ketone /methacrolein + 2-methylerythritol + cis-pinonic acid + β-caryophyllinic acid + 3-acetyl-pentanedioic acid
**residuals deviance min**	-6.88E+00	-1.75E+01	-1.02E+01	-1.95E+01	-1.40E+01	-1.64E+01
**residuals deviance median**	-4.13E-01	-2.86E+00	-7.95E-01	-1.35E+00	-8.35E-01	-6.62E-01
**residuals deviance mean**	3.52E-14	-1.35E-14	-3.84E-15	4.41E-15	1.82E-14	6.07E-15
**residuals deviance max**	8.30E+00	2.19E+01	1.24E+01	2.46E+01	2.23E+01	1.36E+01
**null deviance**	6.45E+03	6.45E+03	6.45E+03	6.45E+03	6.45E+03	6.45E+03
**residual deviance**	5.51E+02	3.55E+03	9.09E+02	3.92E+03	1.82E+03	1.38E+03
**R^2**	**0.91**	0.45	**0.86**	0.39	**0.72**	**0.79**
**intercept coefficient**	23.19	45.54	27.30	47.15	21.48	29.72
**coefficient 1**	6.73E+03	7.48E+02	1.93E+02	-0.01	2.58E+03	0.00
**coefficient 2**	-3.49E+03	1.38E+04	3.33E+04	2.60E+03	-8.60E+02	17.64
**coefficient 3**	-3.40E+04	-5.91E+03	1.02E+04	-3.04E+03	-6.66E+03	-23.10
**coefficient 4**	1.58E+05	-0.01	-2.04	-8.68E+03	1.91E+04	6.71E+05
**coefficient 5**	1.79E+04	-	6.14E+04	-	-	1.26E+05
**coefficient 6**	-0.02	-	-5.11E+04	-	-	-5.67E+04
**coefficient 7**	0.19	-	-1.17E+05	-	-	-1.32E+07
**coefficient 8**	-2.42E+05	-	1.14E+05	-	-	-
**intercept coeff. variation**	2.36e+01 (1.08e+01, 4.30e+01)	4.43e+01 (2.54e+01, 5.98e+01)	2.71e+01 (1.39e+01, 4.22e+01)	4.70e+01 (3.73e+01, 5.87e+01)	2.12e+01 (1.17e+01, 2.69e+01)	3.01e+01 (1.70e+01, 4.27e+01)
**coeff. variation 1**	6.86e+03 (4.75e+03, 9.66e+03)	7.35e+02 (1.17e+02, 1.43e+03)	1.95e+02 (-3.65e+01, 4.06e+02)	-1.05e-02 (-1.89e-02, -4.62e-03)	2.53e+03 (1.66e+03, 3.34e+03)	-3.63e-03 (-8.93e-03, 7.99e-04)
**coeff. variation 2**	-3.53e+03 (-5.43e+03, -2.05e+03)	1.73e+04 (-6.68e+03, 6.64e+04)	3.35e+04 (1.02e+04, 5.16e+04)	2.33e+03 (-1.41e+03, 6.19e+03)	-8.25e+02 (-1.47e+03, 2.64e+02)	1.81e+01 (-1.64e+00, 3.49e+01)
**coeff. variation 3**	-3.62e+04 (-8.38e+04, -2.25e+04)	-5.56e+03 (-1.98e+04, 7.05e+03)	1.02e+04 (5.43e+03, 1.53e+04)	-2.37e+03 (-7.83e+03, 4.89e+03)	-6.64e+03 (-1.08e+04, -4.14e+03)	-2.36e+01 (-4.49e+01, -1.52e+00)
**coeff. variation 4**	1.61e+05 (8.52e+04, 2.73e+05)	-1.20e-02 (-1.89e-02, -4.71e-03)	-2.00e+00 (-2.86e+00, -7.45e-01)	-7.29e+03 (-1.83e+05, 2.02e+05)	1.99e+04 (1.14e+04, 3.94e+04)	6.64e+05 (1.61e+05, 9.85e+05)
**coeff. variation 5**	1.81e+04 (1.25e+04, 2.51e+04)	-	6.10e+04 (1.10e+03, 1.40e+05)	-	-	1.24e+05 (1.12e+04, 1.84e+05)
**coeff. variation 6**	-1.73e-02 (-2.94e-02, -7.29e-03)	-	-5.12e+04 (-7.94e+04, -3.02e+04)	-	-	-5.86e+04 (-9.16e+04, -1.92e+04)
**coeff.**	2.04e-01	-	-1.16e+05	-	-	-1.25e+07

**variation 7**	(4.88e-02, 4.00e-01)		(-2.68e+05, 1.65e+04)			(-2.38e+07, -2.30e+06)
**coeff. variation 8**	-2.51e+05 (-3.79e+05, -1.49e+05)	-	1.14e+05 (4.20e+04, 2.22e+05)	-	-	-
**intercept std error**	4.91	9.53	5.10	6.20	3.79	7.37
**std error 1**	8.16E+02	4.00E+02	8.69E+01	0.00	5.22E+02	2.56E-03
**std error 2**	5.95E+02	6.05E+03	8.41E+03	1.70E+03	3.56E+02	8.13
**std error 3**	5.58E+03	5.62E+03	2.80E+03	2.11E+03	1.91E+03	8.30
**std error 4**	3.26E+04	3.49E-03	0.40	5.96E+04	5.45E+03	2.33E+05
**std error 5**	2.82E+03	-	2.71E+04	-	-	3.80E+04
**std error 6**	0.00	-	1.45E+04	-	-	1.32E+04
**std error 7**	0.06	-	5.21E+04	-	-	3.62E+06
**std error 8**	4.00E+04	-	4.19E+04	-	-	-
**intercept p-value**	**1.04E-04**	**6.06E-05**	**2.25E-05**	**4.49E-08**	**5.77E-06**	**5.20E-04**
**p-value 1**	**3.53E-08**	0.07	**0.04**	**0.01**	**3.85E-05**	0.20
**p-value 2**	**6.63E-06**	**0.03**	**6.63E-04**	0.14	**0.02**	**0.04**
**p-value 3**	**3.99E-06**	0.30	**1.42E-03**	0.16	**1.77E-03**	**0.01**
**p-value 4**	**7.45E-05**	**1.55E-03**	**4.51E-05**	0.89	**1.71E-03**	**0.01**
**p-value 5**	**2.21E-06**	-	**0.03**	-	-	**2.97E-03**
**p-value 6**	**2.66E-04**	-	**1.90E-03**	-	-	**2.62E-04**
**p-value 7**	**2.86E-03**	-	**0.03**	-	-	**1.40E-03**
**p-value 8**	**4.27E-06**	-	**0.01**	-	-	-

Summer OP_m DTT

Table S19. MLR model parameters for summer DTT source models**.**

	**vehicle emissions**	**biomass burning**	**coal / fossil fuel**	**cooking markers**	**dust**	**biogenic SOA**
**model terms**	total EC + Cr + Co + Ni + Pb + C25 + C26 + C27	total EC + K^+ + mannosan + O_3	Cl^- + Ba + Pb + C25 + C26 + C27 + C28	CO + palmitic acid + stearic acid + cholesterol	K^+ + Al + Mn + Fe	isoprene + 2-methylthreitol + 2-methylerythritol + 3-methyl-2,3,4-trihydroxy-1-butene + trans-2-methyl-1,3,4-trihydroxy-1-butene + 3-hydroxy-glutaric acid + pinic acid + 3-acetylpentane-dioic acid
**residuals deviance min**	-10.52	-12.40	-8.31	-12.94	-10.09	-12.59
**residuals deviance median**	-0.59	-0.62	-0.32	0.68	0.11	1.15
**residuals deviance mean**	8.88E-15	-1.62E-16	8.50E-15	-4.14E-15	5.39E-17	3.12E-15
**residuals deviance max**	8.20	17.30	11.84	16.14	12.17	10.50
**null deviance**	2.77E+03	2.77E+03	2.77E+03	2.77E+03	2.77E+03	2.77E+03
**residual deviance**	5.58E+02	1.62E+03	8.85E+02	1.77E+03	1.49E+03	1.08E+03
**R^2**	**0.80**	0.41	0.68	0.36	0.46	0.61
**intercept coefficient**	-1.81	7.34	7.60	22.04	14.69	18.08
**coefficient 1**	-2.88E+02	3.30E+02	1.54E+02	-0.01	2.69E+02	-7.40

**coefficient 2**	5.11E+04	4.43E+02	6.78E+03	1.04E+04	-1.15E+03	3.02E+05
**coefficient 3**	2.40E+05	3.59E+04	6.69E+03	-1.63E+04	-1.07E+04	-1.70E+05
**coefficient 4**	-4.48E+04	0.04	-2.55E+05	5.98E+05	1.69E+03	4.64E+06
**coefficient 5**	6.65E+03	-	5.84E+05	-	-	-2.05E+06
**coefficient 6**	-2.36E+05	-	1.47E+05	-	-	6.07E+05
**coefficient 7**	5.63E+05	-	-4.20E+05	-	-	5.04E+04
**coefficient 8**	9.83E+04	-	-	-	-	2.47E+06
**intercept coeff. variation**	-2.12e+00 (-8.51e+00, 5.23e+00)	6.83e+00 (-4.67e+00, 1.61e+01)	7.46e+00 (-1.32e+00, 1.40e+01)	2.16e+01 (2.80e+00, 2.98e+01)	1.47e+01 (9.49e+00, 2.38e+01)	1.74e+01 (5.29e+00, 3.30e+01)
**coeff. variation 1**	-2.78e+02 (-6.23e+02, -1.97e+01)	3.25e+02 (3.50e+01, 5.86e+02)	1.45e+02 (-8.58e+01, 3.83e+02)	-8.79e-03 (-1.91e-02, 1.54e-02)	2.34e+02 (-5.82e+02, 5.73e+02)	-7.29e+00 (-1.94e+01, 1.21e+00)
**coeff. variation 2**	5.15e+04 (3.13e+04, 7.04e+04)	4.57e+02 (5.68e+01, 9.78e+02)	6.45e+03 (-3.33e+03, 1.33e+04)	1.27e+04 (-3.57e+03, 4.96e+04)	-1.15e+03 (-1.72e+03, -4.58e+02)	2.87e+05 (-9.55e+04, 5.26e+05)
**coeff. variation 3**	2.57e+05 (3.38e+04, 4.97e+05)	4.23e+04 (1.47e+04, 1.27e+05)	6.79e+03 (2.27e+03, 1.30e+04)	-1.97e+04 (-7.94e+04, 1.41e+04)	-1.06e+04 (-2.33e+04, -3.69e+02)	-1.62e+05 (-2.83e+05, 3.10e+04)
**coeff. variation 4**	-4.69e+04 (-1.29e+05, -4.42e+03)	4.77e-02 (-7.66e-02, 1.50e-01)	-2.57e+05 (-3.62e+05, -1.67e+05)	5.93e+05 (2.02e+05, 8.31e+05)	1.70e+03 (1.21e+03, 2.31e+03)	4.64e+06 (2.41e+06, 6.90e+06)
**coeff. variation 5**	6.81e+03 (3.75e+03, 1.07e+04)	-	6.08e+05 (3.68e+05, 1.10e+06)	2.16e+01 (2.80e+00, 2.98e+01)	1.47e+01 (9.49e+00, 2.38e+01)	-2.13e+06 (-4.36e+06, 1.65e+05)
**coeff. variation 6**	-2.32e+05 (-3.00e+05, -1.65e+05)	-	1.46e+05 (8.47e+04, 2.34e+05)	-	-	6.28e+05 (2.50e+05, 1.44e+06)
**coeff. variation 7**	5.50e+05 (4.05e+05, 7.07e+05)	-	-4.33e+05 (-8.60e+05, -1.74e+05)	-	-	5.78e+04 (2.48e+04, 1.83e+05)
**coeff. variation 8**	9.83e+04 (2.12e+04, 1.49e+05)	-	-	-	-	2.32e+06 (-2.88e+05, 3.61e+06)
**intercept std error**	4.47	7.44	4.16	7.12	3.82	6.04
**std error 1**	1.58E+02	1.08E+02	8.17E+01	0.01	2.71E+02	6.03
**std error 2**	1.11E+04	2.80E+02	4.21E+03	7.95E+03	3.29E+02	1.15E+05
**std error 3**	1.54E+05	2.60E+04	3.01E+03	1.75E+04	5.48E+03	5.76E+04
**std error 4**	2.27E+04	0.08	5.96E+04	1.81E+05	3.98E+02	1.09E+06
**std error 5**	2.15E+03	-	1.53E+05	-	-	1.08E+06
**std error 6**	4.36E+04	-	4.70E+04	-	-	2.28E+05
**std error 7**	9.98E+04	-	1.96E+05	-	-	2.50E+04
**std error 8**	3.94E+04	-	-	-	-	1.03E+06
**intercept p-value**	0.69	0.33	0.08	**4.42E-03**	**6.32E-04**	**6.33E-03**
**p-value 1**	0.08	**4.84E-03**	0.07	0.31	0.33	0.23
**p-value 2**	**1.14E-04**	0.13	0.12	0.20	**1.59E-03**	**1.52E-02**
**p-value 3**	0.13	0.18	**0.04**	0.36	0.06	**6.88E-03**
**p-value 4**	0.06	0.58	**2.37E-04**	**2.60E-03**	**2.26E-04**	**2.80E-04**
**p-value 5**	**4.89E-03**	-	**7.68E-04**	-	-	0.07
**p-value 6**	**1.49E-05**	-	**4.34E-03**	-	-	**1.36E-02**
**p-value 7**	**8.15E-06**	-	**0.04**	-	-	**0.05**
**p-value 8**	**1.98E-02**	-	-	-	-	**2.49E-02**

Winter OP_m DCFH

Table S20. MLR model parameters for winter DCFH source models.

	vehicle emissions	biomass burning	coal / fossil fuel	cooking markers	dust	biogenic SOA
**model terms**	Mn + Co + Zn + CO + NO2 + C24 + C25 + C34	total EC + Cu + galactosan + levoglucosan + O_3 + CO	SO_4^{2-} + Cu + Pb + C25 + C26 + C28 + C29 + C31	CO + palmitic acid + stearic acid + cholesterol	K^+ + Na^+ + Cl^- + Fe	SO_4^{2-} + CO + 2-methylerythritol + cis-2-methyl-1,3,4-trihydroxy-1-butene + 3-methyl-2,3,4-trihydroxy-1-butene + trans-2-methyl-1,3,4-trihydroxy-1-butene + cis-pinonic acid + β-caryophyllinic acid
**residuals deviance min**	-2.45E-03	-4.85E-03	-2.86E-03	-6.28E-03	-3.01E-03	-6.01E-03
**residuals deviance median**	8.78E-05	-5.43E-05	-2.04E-04	-7.06E-05	-4.94E-05	-1.37E-04
**residuals deviance mean**	-2.14E-17	-2.93E-18	-2.51E-18	-1.08E-18	1.91E-18	-3.32E-18
**residuals deviance max**	1.97E-03	6.66E-03	4.00E-03	1.05E-02	8.74E-03	5.13E-03
**null deviance**	2.99E-04	2.99E-04	2.99E-04	2.99E-04	2.99E-04	2.99E-04
**residual deviance**	3.21E-05	1.25E-04	7.57E-05	2.74E-04	1.49E-04	1.36E-04
**R^2**	**0.89**	0.58	**0.75**	0.08	0.50	0.55
**intercept coefficient**	1.34E-02	0.02	4.57E-03	7.57E-03	6.40E-03	0.01
**coefficient 1**	-10.67	-0.09	0.05	-4.14E-08	0.38	0.07
**coefficient 2**	-334.25	-1.90	-2.52	0.26	0.21	-2.46E-06
**coefficient 3**	2.19	20.15	1.50	-0.32	-0.09	-1.99E+02
**coefficient 4**	4.71E-06	-1.79	-13.39	-23.43	-0.15	-5.61E+03
**coefficient 5**	-2.97E-04	-2.85E-04	34.14	-	-	2.69E+03
**coefficient 6**	24.92	-2.40E-06	-24.61	-	-	3.12E+02
**coefficient 7**	-23.91	-	6.27	-	-	-56.66
**coefficient 8**	-59.27	-	-31.26	-	-	15.85
**intercept coeff. variation**	1.30e-02 (9.71e-03, 1.56e-02)	1.75e-02 (1.07e-02, 2.56e-02)	4.69e-03 (2.81e-03, 8.33e-03)	7.45e-03 (4.26e-03, 9.82e-03)	6.33e-03 (3.86e-03, 8.63e-03)	8.19e-03 (1.81e-03, 1.18e-02)
**coeff. variation 1**	-1.02e+01 (-1.61e+01, -3.06e+00)	-9.52e-02 (-2.00e-01, 1.26e-02)	4.70e-02 (-3.41e-03, 6.58e-02)	9.41e-09 (-1.34e-06, 1.22e-06)	3.90e-01 (2.31e-01, 6.69e-01)	6.82e-02 (3.10e-02, 1.05e-01)
**coeff. variation 2**	-3.34e+02 (-4.50e+02, -9.60e+01)	-1.44e+00 (-5.16e+00, 4.49e+00)	-3.21e+00 (-2.54e+01, -1.59e+00)	1.58e-01 (-1.63e+00, 1.53e+00)	2.01e-01 (-3.96e-02, 4.95e-01)	-2.51e-06 (-4.12e-06, 1.19e-06)
**coeff. variation 3**	2.14e+00 (9.87e-01, 2.72e+00)	2.08e+01 (6.99e+00, 3.30e+01)	1.61e+00 (4.14e-01, 4.02e+00)	-1.24e-01 (-1.79e+00, 2.42e+00)	-8.59e-02 (-1.66e-01, -2.11e-02)	-2.05e+02 (-3.62e+02, 5.40e+01)

**coeff. variation 4**	4.65e-06 (3.11e-06, 6.75e-06)	-1.84e+00 (-2.95e+00, -5.93e-01)	-1.41e+01 (-3.57e+01, 3.70e+00)	-2.34e+01 (-1.36e+02, 3.46e+01)	-1.59e-01 (-3.44e-01, 8.32e-02)	-5.73e+03 (-9.85e+03, 1.47e+03)
**coeff. variation 5**	-2.88e-04 (-3.84e-04, -1.66e-04)	-2.72e-04 (-5.07e-04, -1.72e-05)	3.55e+01 (3.06e+00, 7.77e+01)	-	-	2.81e+03 (1.06e+02, 5.39e+03)
**coeff. variation 6**	2.52e+01 (1.77e+01, 4.14e+01)	-2.35e-06 (-4.71e-06, -5.10e-07)	-2.55e+01 (-5.96e+01, 1.24e+00)	-	-	3.28e+02 (-2.03e+01, 6.90e+02)
**coeff. variation 7**	-2.42e+01 (-4.06e+01, -1.63e+01)	1.75e-02 (1.07e-02, 2.56e-02)	5.82e+00 (-9.04e+00, 1.51e+01)		-	-5.75e+01 (-1.67e+02, -2.85e+01)
**coeff. variation 8**	-5.61e+01 (-7.88e+01, -1.80e+01)	-9.52e-02 (-2.00e-01, 1.26e-02)	-2.96e+01 (-4.40e+01, 2.00e+00)	-	-	1.63e+01 (5.17e+00, 2.94e+01)
**intercept std error**	1.23E-03	3.50E-03	1.12E-03	1.64E-03	1.55E-03	1.98E-03
**std error 1**	1.71	0.05	0.01	9.17E-07	0.09	0.02
**std error 2**	35.34	1.24	1.08	0.45	0.22	9.26E-07
**std error 3**	0.24	6.71	0.86	0.56	0.03	84.29
**std error 4**	7.74E-07	0.64	7.37	15.74	0.09	2011.24
**std error 5**	4.03E-05	1.34E-04	13.71	-	-	1065.22
**std error 6**	5.80	1.23E-06	9.03	-	-	133.67
**std error 7**	5.82	-	4.16	-	-	20.70
**std error 8**	9.48	-	8.92	-	-	5.30
**intercept p-value**	**2.47E-10**	**3.68E-05**	**4.92E-04**	**9.09E-05**	**3.35E-04**	**3.79E-04**
**p-value 1**	**2.84E-06**	**0.05**	**6.03E-04**	0.96	**3.86E-04**	**7.76E-04**
**p-value 2**	**3.30E-09**	0.14	**0.03**	0.57	0.36	**1.43E-02**
**p-value 3**	**7.07E-09**	**6.18E-03**	0.10	0.57	**6.61E-03**	**0.03**
**p-value 4**	**3.97E-06**	**1.00E-02**	0.08	0.15	0.12	**1.07E-02**
**p-value 5**	**2.24E-07**	**0.04**	**0.02**	-	-	**0.02**
**p-value 6**	**2.92E-04**	0.06	**1.24E-02**	-	-	**0.03**
**p-value 7**	**4.66E-04**	-	0.15	-	-	**1.20E-02**
**p-value 8**	**2.71E-06**	-	**2.01E-03**	-	-	**6.75E-03**

Summer OP_m DCFH

Table S21. MLR model parameters for summer DCFH source models**.**

	**vehicle emissions**	**biomass burning**	**coal / fossil fuel**	**cooking markers**	**dust**	**biogenic SOA**
**model terms**	total EC +<br>Ba +<br>O_3 +<br>CO +<br>NO_y +<br>C29 +<br>C31	total EC +<br>K^+ +<br>O_3 +<br>CO	SO_4^{2-} +<br>Cl^- +<br>Cu +<br>Cd +<br>C24 +<br>C26 +<br>C28 +<br>C31	CO +<br>palmitic acid +<br>stearic acid +<br>cholesterol	Na^+ +<br>Ca^{2+} +<br>Mn +<br>Zn	SO_4^{2-} + CO +<br>isoprene +<br>methyl ethyl ketone +<br>3-methyl-2,3,4-trihydroxy-1-butene +<br>trans-2-methyl-1,3,4-trihydroxy-1-butene +<br>3-hydroxyglutaric acid +<br>β-caryophyllinic acid
**residuals deviance min**	-2.92E-03	-3.33E-03	-3.48E-03	-4.25E-03	-3.61E-03	-2.61E-03
**residuals deviance median**	-3.00E-04	6.18E-05	1.98E-04	-3.06E-06	1.81E-04	2.69E-04
**residuals deviance mean**	9.79E-18	5.06E-19	-3.64E-18	-9.46E-19	-1.49E-18	-2.37E-19
**residuals deviance max**	4.05E-03	7.66E-03	4.17E-03	8.23E-03	9.00E-03	2.33E-03

**null deviance**	2.57E-04	2.57E-04	2.57E-04	2.57E-04	2.57E-04	2.57E-04
**residual deviance**	9.72E-05	1.78E-04	7.38E-05	1.96E-04	1.89E-04	7.78E-05
**R^2**	0.62	0.31	**0.71**	0.24	0.26	**0.70**
**intercept coefficient**	0.02	9.63E-03	-0.01	0.01	0.01	-1.76E-03
**coefficient 1**	-0.29	-0.11	0.05	2.20E-06	0.40	0.04
**coefficient 2**	4.64	0.05	-0.09	3.12	-0.34	3.41E-06
**coefficient 3**	-1.61E-04	-5.64E-05	9.23	-7.55	-2.54	-7.01E-03
**coefficient 4**	1.14E-05	4.20E-06	5.99	-1.26E+02	0.44	5.11E-03
**coefficient 5**	-1.26E-04		6.84E+01			-6.21E+02
**coefficient 6**	4.68E+01		-2.22E+02			1.14E+03
**coefficient 7**	-3.33E+01		3.54E+02			-2.33E+02
**coefficient 8**			-3.90E+01			-3.99E+01
**intercept coeff. variation**	1.67e-02 (8.77e-03, 2.51e-02)	9.59e-03 (4.76e-03, 1.30e-02)	-6.12e-03 (-1.13e-02, 1.04e-03)	8.02e-03 (4.61e-03, 1.23e-02)	6.57e-03 (4.37e-03, 9.38e-03)	-1.57e-03 (-8.17e-03, 4.77e-03)
**coeff. variation 1**	-2.88e-01 (-4.07e-01, -1.05e-01)	-1.09e-01 (-1.53e-01, -2.24e-02)	5.01e-02 (2.43e-02, 6.96e-02)	1.92e-06 (-3.92e-06, 7.22e-06)	4.09e-01 (4.37e-02, 7.66e-01)	4.38e-02 (1.01e-02, 5.71e-02)
**coeff. variation 2**	4.57e+00 (8.96e-01, 7.21e+00)	4.73e-02 (-8.93e-02, 2.18e-01)	-8.92e-02 (-1.29e-01, -4.39e-02)	2.96e+00 (-7.34e+00, 9.07e+00)	-3.43e-01 (-4.88e-01, -1.48e-01)	3.55e-06 (3.88e-07, 1.08e-05)
**coeff. variation 3**	-1.61e-04 (-2.46e-04, -6.22e-05)	-5.49e-05 (-1.10e-04, -3.45e-06)	8.73e+00 (2.19e+00, 1.43e+01)	-7.24e+00 (-1.88e+01, 9.31e+00)	-2.75e+00 (-7.56e+00, -1.48e+00)	-7.06e-03 (-1.12e-02, -3.59e-03)
**coeff. variation 4**	1.11e-05 (2.76e-06, 1.84e-05)	4.01e-06 (-4.63e-06, 7.96e-06)	5.62e+00 (-2.69e+00, 8.70e+00)	-1.29e+02 (-2.18e+02, -3.09e+01)	4.37e-01 (-2.19e-01, 1.03e+00)	5.06e-03 (1.77e-03, 7.27e-03)
**coeff. variation 5**	-1.26e-04 (-1.90e-04, -2.89e-05)		6.58e+01 (2.86e+01, 8.99e+01)			-5.97e+02 (-9.78e+02 ,-5.52e+01)
**coeff. variation 6**	4.76e+01 (2.65e+01, 7.13e+01)		-2.15e+02 (-3.03e+02, -1.16e+02)			1.12e+03 (2.09e+02, 1.69e+03)
**coeff. variation 7**	-3.36e+01 (-5.69e+01, -1.95e+01)		3.46e+02 (1.91e+02, 4.72e+02)			-2.32e+02 (-4.54e+02, -8.63e+01)
**coeff. variation 8**			-3.86e+01 (-6.26e+01, -2.51e+01)			-3.97e+01 (-6.81e+01, -6.33e-01)
**intercept std error**	2.83E-03	2.59E-03	2.80E-03	2.37E-03	1.22E-03	2.78E-03
**std error 1**	0.08	0.03	0.01	3.06E-06	0.24	7.57E-03
**std error 2**	1.65	0.09	0.03	2.65	0.14	2.42E-06
**std error 3**	3.72E-05	2.90E-05	4.17	5.82	1.30	1.78E-03
**std error 4**	3.08E-06	3.06E-06	3.16	6.02E+01	0.41	1.36E-03
**std error 5**	3.83E-05		2.26E+01			2.72E+02
**std error 6**	1.38E+01		6.22E+01			3.30E+02
**std error 7**	1.14E+01		8.53E+01			6.31E+01
**std error 8**			7.96			1.41E+01
**intercept p-value**	**3.72E-06**	**8.89E-04**	**0.03**	**2.55E-03**	**1.34E-05**	0.53
**p-value 1**	**8.62E-04**	**3.56E-03**	**7.28E-06**	0.48	0.10	**3.96E-06**
**p-value 2**	**9.27E-03**	0.60	**0.01**	0.25	**0.02**	0.17
**p-value 3**	**2.10E-04**	0.06	**0.04**	0.20	0.06	**6.08E-04**
**p-value 4**	**1.06E-03**	0.18	0.07	**0.04**	0.30	**1.00E-03**
**p-value 5**	**3.01E-03**		**5.82E-03**			**0.03**
**p-value 6**	**2.37E-03**		**1.57E-03**			**2.11E-03**
**p-value 7**	**7.40E-03**		**3.61E-04**			**1.15E-03**
**p-value 8**			**5.38E-05**			**9.31E-03**

Supplementary references

Abrams, J. Y., Weber, R. J., Klein, M., Sarnat, S. E., Chang, H. H., Strickland, M. J., Verma, V., Fang, T., Bates, J. T., Mulholland, J. A., Russell, A. G. and Tolbert, P. E.: Associations between Ambient Fine Particulate Oxidative Potential and Cardiorespiratory Emergency Department Visits, Environ. Health Perspect., 125(12), 129001, doi:10.1289/EHP3048, 2017.

Atkinson, R. W., Samoli, E., Analitis, A., Fuller, G. W., Green, D. C., Anderson, H. R., Purdie, E., Dunster, C., Aitlhadj, L., Kelly, F. J. and Mudway, I. S.: Short-term associations between particle oxidative potential and daily mortality and hospital admissions in London, Int. J. Hyg. Environ. Health, 219(6), 566–572, doi:10.1016/j.ijheh.2016.06.004, 2016.

Ayres, J. G., Borm, P., Cassee, F. R., Castranova, V., Donaldson, K., Ghio, A., Harrison, R. M., Hider, R., Kelly, F., Kooter, I. M., Marano, F., Maynard, R. L., Mudway, I., Nel, A., Sioutas, C., Smith, S., Baeza-Squiban, A., Cho, A., Duggan, S. and Froines, J.: Evaluating the toxicity of airborne particulate matter and nanoparticles by measuring oxidative stress potential - A workshop report and consensus statement, Inhal. Toxicol., 20(1), 75–99, doi:10.1080/08958370701665517, 2008.

Bates, J. T., Fang, T., Verma, V., Zeng, L., Weber, R. J., Tolbert, P. E., Abrams, J. Y., Sarnat, S. E., Klein, M., Mulholland, J. A. and Russell, A. G.: Review of Acellular Assays of Ambient Particulate Matter Oxidative Potential: Methods and Relationships with Composition, Sources, and Health Effects, Environ. Sci. Technol., 53(8), 4003–4019, doi:10.1021/acs.est.8b03430, 2019.

Brook, R. D., Franklin, B., Cascio, W., Hong, Y., Howard, G., Lipsett, M., Luepker, R., Mittleman, M., Samet, J., Smith, S. C. and Tager, I.: Air pollution and cardiovascular disease: A statement for healthcare professionals from the expert panel on population and prevention science of the American Heart Association, Circulation, 109(21), 2655–2671, doi:10.1161/01.CIR.0000128587.30041.C8, 2004.

Burini, G.: Development of a quantitative method for the analysis of total l-ascorbic acid in foods by high-performance liquid chromatography, J. Chromatogr. A, 1154(1–2), 97–102, doi:10.1016/j.chroma.2007.03.013, 2007.

Calas, A., Uzu, G., Martins, J. M. F., Voisin, Di., Spadini, L., Lacroix, T. and Jaffrezo, J. L.: The importance of simulated lung fluid (SLF) extractions for a more relevant evaluation of the oxidative potential of particulate matter, Sci. Rep., 7(1), 1–12, doi:10.1038/s41598-017-11979-3, 2017.

Calas, A., Uzu, G., Kelly, F. J., Houdier, S., Martins, J. M. F., Thomas, F., Molton, F., Charron, A., Dunster, C., Oliete, A., Jacob, V., Besombes, J. L., Chevrier, F. and Jaffrezo, J. L.: Comparison between five acellular oxidative potential measurement assays performed with detailed chemistry on PM10 samples from the city of Chamonix (France), Atmos. Chem. Phys., 18(11), 7863–7875, doi:10.5194/acp-18-7863-2018, 2018.

Campbell, S. J., Utinger, B., Lienhard, D. M., Paulson, S. E., Shen, J., Griffiths, P. T., Stell, A. C. and Kalberer, M.: Development of a physiologically relevant online chemical assay to quantify aerosol oxidative potential, Anal. Chem., 91, 13088–13095, doi:10.1021/acs.analchem.9b03282, 2019.

Cao, J. J., Lee, S. C., Ho, K. F., Zou, S. C., Fung, K., Li, Y., Watson, J. G. and Chow, J. C.: Spatial and seasonal variations of atmospheric organic carbon and elemental carbon in Pearl River Delta Region, China, Atmos. Environ.,

38(27), 4447–4456, doi:10.1016/j.atmosenv.2004.05.016, 2004.

Charrier, J. G. and Anastasio, C.: On dithiothreitol (DTT) as a measure of oxidative potential for ambient particles: evidence for the importance of soluble transition metals, Atmos. Chem. Phys., 12(19), 9321–9333, doi:10.5194/acp-12-9321-2012, 2012.

Charrier, J. G., Mcfall, A. S., Vu, K. K., Baroi, J., Olea, C., Hasson, A. and Anastasio, C.: A bias in the " mass-normalized " DTT response e An effect of non- linear concentration-response curves for copper and manganese, Atmos. Environ., 144, 325–334, doi:10.1016/j.atmosenv.2016.08.071, 2016.

Chaves, F. J., Mansego, M. L., Blesa, S., Gonzalez-Albert, V., Jiménez, J., Tormos, M. C., Espinosa, O., Giner, V., Iradi, A., Saez, G. and Redon, J.: Inadequate Cytoplasmic Antioxidant Enzymes Response Contributes to the Oxidative Stress in Human Hypertension, Am. J. Hypertens., 20(1), 62–69, doi:10.1016/j.amjhyper.2006.06.006, 2007.

Chen, W. N., Chen, Y. C., Kuo, C. Y., Chou, C. H., Cheng, C. H., Huang, C. C., Chang, S. Y., Roja Raman, M., Shang, W. L., Chuang, T. Y. and Liu, S. C.: The real-time method of assessing the contribution of individual sources on visibility degradation in Taichung, Sci. Total Environ., 497–498(110), 219–228, doi:10.1016/j.scitotenv.2014.07.120, 2014.

Deutsch, M. and Weeks, C.: Microfluorometric Assay for Vitamin C, J. Assoc. Off. Anal. Chem, (48), 1248, 1965.

Dikalov, S., Skatchkov, M. and Bassenge, E.: Quantification of peroxynitrite, superoxide, and peroxyl radicals by a new spin trap hydroxylamine 1-hydroxy-2,2,6,6-tetramethyl-4-oxo-piperidine, Biochem. Biophys. Res. Commun., 230(1), 54–57, doi:10.1006/bbrc.1996.5880, 1997.

Ding, X., Wang, X. M., Gao, B., Fu, X. X., He, Q. F., Zhao, X. Y., Yu, J. Z. and Zheng, M.: Tracer-based estimation of secondary organic carbon in the Pearl River Delta, south China, J. Geophys. Res. Atmos., 117(5), 1–14, doi:10.1029/2011JD016596, 2012.

Du, X., Wu, Y., Fu, L., Wang, S., Zhang, S. and Hao, J.: Intake fraction of PM 2.5 and NO X from vehicle emissions in Beijing based on personal exposure data, Atmos. Environ., 57(2), 233–243, doi:10.1016/j.atmosenv.2012.04.046, 2012.

Duan, F., Liu, X., Yu, T. and Cachier, H.: Identification and estimate of biomass burning contribution to the urban aerosol organic carbon concentrations in Beijing, Atmos. Environ., 38(9), 1275–1282, doi:10.1016/j.atmosenv.2003.11.037, 2004.

Duan, J., Tan, J., Yang, L., Wu, S. and Hao, J.: Concentration, sources and ozone formation potential of volatile organic compounds (VOCs) during ozone episode in Beijing, Atmos. Res., 88(1), 25–35, doi:10.1016/j.atmosres.2007.09.004, 2008.

Farhat, Z., Browne, R. W., Bonner, M. R., Tian, L., Deng, F., Swanson, M. and Mu, L.: How do glutathione antioxidant enzymes and total antioxidant status respond to air pollution exposure?, Environ. Int., 112(September 2017), 287–293, doi:10.1016/j.envint.2017.12.033, 2018.

Feng, R. and Zheng, H. jun: Evidence for regional heterogeneous atmospheric particulate matter distribution in China: implications for air pollution control, Environ. Chem. Lett., 17(4), 1839–1847, doi:10.1007/s10311-019-00890-0, 2019.

Fry, J. L., Draper, D. C., Barsanti, K. C., Smith, J. N., Ortega, J., Winkler, P. M., Lawler, M. J., Brown, S. S., Edwards, P. M., Cohen, R. C. and Lee, L.: Secondary organic aerosol formation and organic nitrate yield from NO3 oxidation of biogenic hydrocarbons, Environ. Sci. Technol., 48(20), 11944–11953, doi:10.1021/es502204x, 2014.

Fuertes, E., van der Plaat, D. A. and Minelli, C.: Antioxidant genes and susceptibility to air pollution for respiratory and cardiovascular health, Free Radic. Biol. Med., 151(January), 88–98, doi:10.1016/j.freeradbiomed.2020.01.181, 2020.

Fuller, S. J., Wragg, F. P. H., Nutter, J. and Kalberer, M.: Comparison of on-line and off-line methods to quantify reactive oxygen species (ROS) in atmospheric aerosols, Atmos. Environ., 92, 97–103, doi:10.1016/j.atmosenv.2014.04.006, 2014.

Gebb, S. and Stevens, T.: On lung endothelial cell heterogeneity, Microvasc. Res., 68(1), 1–12, doi:10.1016/j.mvr.2004.02.002, 2004.

Ghirardo, A., Xie, J., Zheng, X., Wang, Y., Grote, R., Block, K., Wildt, J., Mentel, T., Kiendler-Scharr, A., Hallquist, M., Butterbach-Bahl, K. and Schnitzler, J. P.: Urban stress-induced biogenic VOC emissions and SOA-forming potentials in Beijing, Atmos. Chem. Phys., 16(5), 2901–2920, doi:10.5194/acp-16-2901-2016, 2016.

Godri, K. J., Harrison, R. M., Evans, T., Baker, T., Dunster, C., Mudway, I. S. and Kelly, F. J.: Increased oxidative burden associated with traffic component of ambient particulate matter at roadside and Urban background schools sites in London, PLoS One, 6(7), doi:10.1371/journal.pone.0021961, 2011.

Han, X., Na, T., Wu, T. and Yuan, B. Z.: Human lung epithelial BEAS-2B cells exhibit characteristics of mesenchymal stem cells, PLoS One, 15(1), 1–18, doi:10.1371/journal.pone.0227174, 2020.

He, L. Y., Hu, M., Huang, X. F., Zhang, Y. H. and Tang, X. Y.: Seasonal pollution characteristics of organic compounds in atmospheric fine particles in Beijing, Sci. Total Environ., 359(1–3), 167–176, doi:10.1016/j.scitotenv.2005.05.044, 2006.

Huang, X., Liu, Z., Liu, J., Hu, B., Wen, T., Tang, G., Zhang, J., Wu, F., Ji, D., Wang, L. and Wang, Y.: Chemical characterization and source identification of PM2.5 at multiple sites in the Beijing-Tianjin-Hebei region, China, Atmos. Chem. Phys., 17(21), 12941–12962, doi:10.5194/acp-17-12941-2017, 2017.

Ianniello, A., Spataro, F., Esposito, G., Allegrini, I., Hu, M. and Zhu, T.: Chemical characteristics of inorganic ammonium salts in PM2.5 in the atmosphere of Beijing (China), Atmos. Chem. Phys., 11(21), 10803–10822, doi:10.5194/acp-11-10803-2011, 2011.

Janssen, N. A. H., Yang, A., Strak, M., Steenhof, M., Hellack, B., Gerlofs-Nijland, M. E., Kuhlbusch, T., Kelly, F., Harrison, R., Brunekreef, B., Hoek, G. and Cassee, F.: Oxidative potential of particulate matter collected at sites with different source characteristics, Sci. Total Environ., 472, 572–581, doi:10.1016/j.scitotenv.2013.11.099, 2014.

Janssen, N. A. H., Strak, M., Yang, A., Hellack, B., Kelly, F. J., Kuhlbusch, T. A. J., Harrison, R. M., Brunekreef, B., Cassee, F. R., Steenhof, M. and Hoek, G.: Associations between three specific a-cellular measures of the oxidative potential of particulate matter and markers of acute airway and nasal inflammation in healthy volunteers, Occup. Environ. Med., 72(1), 49–56, doi:10.1136/oemed-2014-102303, 2015.

Ji, D., Zhang, J., He, J., Wang, X., Pang, B., Liu, Z., Wang, L. and Wang, Y.: Characteristics of atmospheric organic and elemental carbon aerosols in urban Beijing, China, Atmos. Environ., 125, 293–306,

doi:10.1016/j.atmosenv.2015.11.020, 2016.

Kelly, F. J., Mudway, I., Blomberg, A. and Frew, A.: Altered lung antioxidant status in patients with mild asthma Should radiologists image and report sacroiliac joints in patients referr e d for examination of the lumbar spine ?, Lancet, 354, 482–483, 1999.

Li, X., Wang, Y., Guo, X. and Wang, Y.: Seasonal variation and source apportionment of organic and inorganic compounds in PM2.5 and PM10 particulates in Beijing, China, J. Environ. Sci. (China), 25(4), 741–750, doi:10.1016/S1001-0742(12)60121-1, 2013.

Liu, Q., Liu, Y., Yin, J., Zhang, M. and Zhang, T.: Chemical characteristics and source apportionment of PM10 during Asian dust storm and non-dust storm days in Beijing, Atmos. Environ., 91, 85–94, doi:10.1016/j.atmosenv.2014.03.057, 2014a.

Liu, Q., Baumgartner, J., Zhang, Y., Liu, Y., Sun, Y. and Zhang, M.: Oxidative potential and inflammatory impacts of source apportioned ambient air pollution in Beijing, Environ. Sci. Technol., 48(21), 12920–12929, doi:10.1021/es5029876, 2014b.

Liu, X., Zhu, J., Van Espen, P., Adams, F., Xiao, R., Dong, S. and Li, Y.: Single particle characterization of spring and summer aerosols in Beijing: Formation of composite sulfate of calcium and potassium, Atmos. Environ., 39(36), 6909–6918, doi:10.1016/j.atmosenv.2005.08.007, 2005.

Mason, R. J. and Williams, M. C.: Phospholipid composition and ultrastructure of A549 cells and other cultured pulmonary epithelial cells of presumed type II cell origin, Biochim. Biophys. Acta (BBA)/Lipids Lipid Metab., 617(1), 36–50, doi:10.1016/0005-2760(80)90222-2, 1980.

Mondal, N. K., Mukherjee, B., Das, D. and Ray, M. R.: Micronucleus formation, DNA damage and repair in premenopausal women chronically exposed to high level of indoor air pollution from biomass fuel use in rural India, Mutat. Res. - Genet. Toxicol. Environ. Mutagen., 697(1–2), 47–54, doi:10.1016/j.mrgentox.2010.02.006, 2010.

Moreb, J. S., Zucali, J. R., Ostmark, B. and Benson, N. A.: Heterogeneity of aldehyde dehydrogenase expression in lung cancer cell lines is revealed by aldefluor flow cytometry-based assay, Cytom. Part B - Clin. Cytom., 72(4), 281–289, doi:10.1002/cyto.b.20161, 2007.

Niki, E.: Assessment of antioxidant capacity in vitro and in vivo, Free Radic. Biol. Med., 49(4), 503–515, doi:10.1016/j.freeradbiomed.2010.04.016, 2010.

Nozière, B., Kalberer, M., Claeys, M., Allan, J., D'Anna, B., Decesari, S., Finessi, E., Glasius, M., Grgić, I., Hamilton, J. F., Hoffmann, T., Iinuma, Y., Jaoui, M., Kahnt, A., Kampf, C. J., Kourtchev, I., Maenhaut, W., Marsden, N., Saarikoski, S., Schnelle-Kreis, J., Surratt, J. D., Szidat, S., Szmigielski, R. and Wisthaler, A.: The Molecular Identification of Organic Compounds in the Atmosphere: State of the Art and Challenges, Chem. Rev., 115(10), 3919–3983, doi:10.1021/cr5003485, 2015.

Oberdorster, G., Ferin, J., Gelein, R., Soderholm, S. C. and Finkelstein, J.: Role of the alveolar macrophage in lung injury: Studies with ultrafine particles, Environ. Health Perspect., 97, 193–199, doi:10.1289/ehp.97-1519541, 1992.

Oberdörster, G., Sharp, Z., Atudorei, V., Elder, A., Gelein, R., Kreyling, W. and Cox, C.: Translocation of inhaled ultrafine particles to the brain, Inhal. Toxicol., 16(6–7), 437–445, doi:10.1080/08958370490439597, 2004.

Pang, X., Mu, Y., Zhang, Y., Lee, X. and Yuan, J.: Contribution of isoprene to formaldehyde and ozone formation

based on its oxidation products measurement in Beijing, China, Atmos. Environ., 43(13), 2142–2147, doi:10.1016/j.atmosenv.2009.01.022, 2009.

Pelfrêne, A., Cave, M. R., Wragg, J. and Douay, F.: In vitro investigations of human bioaccessibility from reference materials using simulated lung fluids, Int. J. Environ. Res. Public Health, 14(2), 1–15, doi:10.3390/ijerph14020112, 2017.

Pernigotti, D., Belis, C. A. and Spanó, L.: SPECIEUROPE: The European data base for PM source profiles, Atmos. Pollut. Res., 7(2), 307–314, doi:10.1016/j.apr.2015.10.007, 2016.

Peters, A., Veronesi, B., Calderón-Garcidueñas, L., Gehr, P., Chen, L. C., Geiser, M., Reed, W., Rothen-Rutishauser, B., Schürch, S. and Schulz, H.: Translocation and potential neurological effects of fine and ultrafine particles a critical update, Part. Fibre Toxicol., 3, 1–13, doi:10.1186/1743-8977-3-13, 2006.

Pietrogrande, M. C., Bacco, D. and Rossi, M.: Chemical characterization of polar organic markers in aerosols in a local area around Bologna, Italy, Atmos. Environ., 75, 279–286, doi:10.1016/j.atmosenv.2013.04.023, 2013.

Pietrogrande, M. C., Bertoli, I., Manarini, F. and Russo, M.: Ascorbate assay as a measure of oxidative potential for ambient particles: Evidence for the importance of cell-free surrogate lung fluid composition, Atmos. Environ., 211(September 2018), 103–112, doi:10.1016/j.atmosenv.2019.05.012, 2019.

Reddel, R. R., Ke, Y., Gerwin, B. I., McMenamin, M. G., Lechner, J. F., Su, R. T., Brash, D. E., Park, J. B., Rhim, J. S. and Harris, C. C.: Transformation of Human Bronchial Epithelial Cells by Infection with SV40 or Adenovirus-12 SV40 Hybrid Virus, or Transfection via Strontium Phosphate Coprecipitation with a Plasmid Containing SV40 Early Region Genes, Cancer Res., 48(7), 1904–1909, 1988.

Samet, J. M. and Cheng, P. W.: The role of airway mucus in pulmonary toxicology, Environ. Health Perspect., 102(SUPPL. 2), 89–103, doi:10.1289/ehp.9410289, 1994.

Shao, M., Lu, S., Liu, Y., Xie, X., Chang, C., Huang, S. and Chen, Z.: Volatile organic compounds measured in summer in Beijing and their role in ground-level ozone formation, J. Geophys. Res. Atmos., 114(7), 1–13, doi:10.1029/2008JD010863, 2009.

Shi, Z., Vu, T., Kotthaus, S., Harrison, R. M., Grimmond, S., Yue, S., Zhu, T., Lee, J., Han, Y., Demuzere, M., Dunmore, R. E., Ren, L., Liu, D., Wang, Y., Wild, O., Allan, J., Joe Acton, W., Barlow, J., Barratt, B., Beddows, D., Bloss, W. J., Calzolai, G., Carruthers, D., Carslaw, D. C., Chan, Q., Chatzidiakou, L., Chen, Y., Crilley, L., Coe, H., Dai, T., Doherty, R., Duan, F., Fu, P., Ge, B., Ge, M., Guan, D., Hamilton, J. F., He, K., Heal, M., Heard, D., Nicholas Hewitt, C., Hollaway, M., Hu, M., Ji, D., Jiang, X., Jones, R., Kalberer, M., Kelly, F. J., Kramer, L., Langford, B., Lin, C., Lewis, A. C., Li, J., Li, W., Liu, H., Liu, J., Loh, M., Lu, K., Lucarelli, F., Mann, G., McFiggans, G., Miller, M. R., Mills, G., Monk, P., Nemitz, E., O'Connor, F., Ouyang, B., Palmer, P. I., Percival, C., Popoola, O., Reeves, C., Rickard, A. R., Shao, L., Shi, G., Spracklen, D., Stevenson, D., Sun, Y., Sun, Z., Tao, S., Tong, S., Wang, Q., Wang, W., Wang, X., Wang, X., Wang, Z., Wei, L., Whalley, L., Wu, X., Wu, Z., Xie, P., Yang, F., Zhang, Q., Zhang, Y., Zhang, Y. and Zheng, M.: Introduction to the special issue "in-depth study of air pollution sources and processes within Beijing and its surrounding region (APHH-Beijing)," Atmos. Chem. Phys., 19(11), 7519–7546, doi:10.5194/acp-19-7519-2019, 2019.

Simon, H., Beck, L., Bhave, P. V., Divita, F., Hsu, Y., Luecken, D., David Mobley, J., Pouliot, G. A., Reff, A., Sarwar,

G. and Strum, M.: The development and uses of EPA's SPECIATE database, Atmos. Pollut. Res., 1(4), 196–206, doi:10.5094/APR.2010.026, 2010.

Singh, R., Sram, R. J., Binkova, B., Kalina, I., Popov, T. A., Georgieva, T., Garte, S., Taioli, E. and Farmer, P. B.: The relationship between biomarkers of oxidative DNA damage, polycyclic aromatic hydrocarbon DNA adducts, antioxidant status and genetic susceptibility following exposure to environmental air pollution in humans, Mutat. Res. - Fundam. Mol. Mech. Mutagen., 620(1–2), 83–92, doi:10.1016/j.mrfmmm.2007.02.025, 2007.

Squires, F., Nemitz, E., Langford, B., Wild, O., Drysdale, W., Acton, W. J. F., Fu, P., Grimmond, C. S. B., Hamilton, J., Hewitt, C. N., Hollaway, M., Kotthaus, S., Lee, J., Metzger, S., Pingintha-Durden, N., Shaw, M., Vaughan, A., Wang, X., Wu, R., Zhang, Q. and Zhang, Y.: Measurements of traffic dominated pollutant emissions in a Chinese megacity, Atmos. Chem. Phys., 1–33, doi:10.5194/acp-2019-1105, 2020.

Strak, M., Janssen, N., Beelen, R., Schmitz, O., Vaartjes, I., Karssenberg, D., van den Brink, C., Bots, M. L., Dijst, M., Brunekreef, B. and Hoek, G.: Long-term exposure to particulate matter, NO2 and the oxidative potential of particulates and diabetes prevalence in a large national health survey, Environ. Int., 108(2), 228–236, doi:10.1016/j.envint.2017.08.017, 2017.

Venkatachari, P. and Hopke, P. K.: Development and laboratory testing of an automated monitor for the measurement of atmospheric particle-bound reactive oxygen species (ROS)., Aerosol Sci. Technol., 42(8), 629–635, 2008.

Wang, X. L., Rainwater, D. L., VandeBerg, J. F., Mitchell, B. D. and Mahaney, M. C.: Genetic contributions to plasma total antioxidant activity, Arterioscler. Thromb. Vasc. Biol., 21(7), 1190–1195, doi:10.1161/hq0701.092146, 2001.

Wang, Y., Hu, M., Guo, S., Wang, Y., Zheng, J., Yang, Y., Zhu, W., Tang, R., Li, X., Liu, Y., Le Breton, M., Du, Z., Shang, D., Wu, Y., Wu, Z., Song, Y., Lou, S., Hallquist, M. and Yu, J.: The Secondary Formation of Organosulfates under the Interactions between Biogenic Emissions and Anthropogenic Pollutants in Summer of Beijing, Atmos. Chem. Phys. Discuss., 1–37, doi:10.5194/acp-2018-262, 2018.

Watanabe, N., Dickinson, D. A., Krzywanski, D. M., Iles, K. E., Zhang, H., Venglarik, C. J. and Forman, H. J.: A549 subclones demonstrate heterogeneity in toxicological sensitivity and antioxidant profile, Am. J. Physiol. - Lung Cell. Mol. Physiol., 283(4 27-4), 726–736, doi:10.1152/ajplung.00025.2002, 2002.

Weichenthal, S., Lavigne, E., Evans, G., Pollitt, K. and Burnett, R. T.: Ambient PM2.5 and risk of emergency room visits for myocardial infarction: Impact of regional PM2.5 oxidative potential: A case-crossover study, Environ. Heal. A Glob. Access Sci. Source, 15(1), 1–9, doi:10.1186/s12940-016-0129-9, 2016a.

Weichenthal, S. A., Lavigne, E., Evans, G. J., Godri Pollitt, K. J. and Burnett, R. T.: Fine particulate matter and emergency room visits for respiratory illness: Effect modification by oxidative potential, Am. J. Respir. Crit. Care Med., 194(5), 577–586, doi:10.1164/rccm.201512-2434OC, 2016b.

Yang, A., Jedynska, A., Hellack, B., Kooter, I., Hoek, G., Brunekreef, B., Kuhlbusch, T. A. J., Cassee, F. R. and Janssen, N. A. H.: Measurement of the oxidative potential of PM2.5 and its constituents: The effect of extraction solvent and filter type, Atmos. Environ., 83, 35–42, doi:10.1016/j.atmosenv.2013.10.049, 2014a.

Yang, A., Jedynska, A., Hellack, B., Kooter, I., Hoek, G., Brunekreef, B., Kuhlbusch, T. A. J., Cassee, F. R. and Janssen, N. A. H.: Measurement of the oxidative potential of PM2.5 and its constituents: The effect of extraction solvent and filter type, Atmos. Environ., 83, 35–42, doi:10.1016/j.atmosenv.2013.10.049, 2014b.

Yang, A., Janssen, N. A. H., Brunekreef, B., Cassee, F. R., Hoek, G. and Gehring, U.: Children's respiratory health and oxidative potential of PM2.5: The PIAMA birth cohort study, Occup. Environ. Med., 73(3), 154–160, doi:10.1136/oemed-2015-103175, 2016.

Yang, F., He, K., Ye, B., Chen, X., Cha, L., Cadle, S. H., Chan, T. and Mulawa, P. A.: One-year record of organic and elemental carbon in fine particles in downtown Beijing and Shanghai, Atmos. Chem. Phys., 5(6), 1449–1457, doi:10.5194/acp-5-1449-2005, 2005.

Yang, J. and Zhang, B.: Air pollution and healthcare expenditure: Implication for the benefit of air pollution control in China, Environ. Int., 120(August), 443–455, doi:10.1016/j.envint.2018.08.011, 2018.

Yu, H., Puthussery, J. V. and Verma, V.: A semi-automated multi-endpoint reactive oxygen species activity analyzer (SAMERA) for measuring the oxidative potential of ambient PM2.5 aqueous extracts, Aerosol Sci. Technol., 54(3), 304–320, doi:10.1080/02786826.2019.1693492, 2019a.

Yu, J., Yan, C., Liu, Y., Li, X., Zhou, T. and Zheng, M.: Potassium: A tracer for biomass burning in Beijing?, Aerosol Air Qual. Res., 18(9), 2447–2459, doi:10.4209/aaqr.2017.11.0536, 2018.

Yu, S. Y., Liu, W. J., Xu, Y. S., Yi, K., Zhou, M., Tao, S. and Liu, W. X.: Characteristics and oxidative potential of atmospheric PM2.5 in Beijing: Source apportionment and seasonal variation, Sci. Total Environ., 650, 277–287, doi:10.1016/j.scitotenv.2018.09.021, 2019b.

Zhang, T., Claeys, M., Cachier, H., Dong, S., Wang, W., Maenhaut, W. and Liu, X.: Identification and estimation of the biomass burning contribution to Beijing aerosol using levoglucosan as a molecular marker, Atmos. Environ., 42(29), 7013–7021, doi:10.1016/j.atmosenv.2008.04.050, 2008.

Zhang, Y., Chen, J., Yang, H., Li, R. and Yu, Q.: Seasonal variation and potential source regions of PM2.5-bound PAHs in the megacity Beijing, China: Impact of regional transport, Environ. Pollut., 231, 329–338, doi:10.1016/j.envpol.2017.08.025, 2017a.

Zhang, Y. L., Schnelle-Kreis, J., Abbaszade, G., Zimmermann, R., Zotter, P., Shen, R. R., Schäfer, K., Shao, L., Prévôt, A. S. H. and Szidat, S.: Source Apportionment of Elemental Carbon in Beijing, China: Insights from Radiocarbon and Organic Marker Measurements, Environ. Sci. Technol., 49(14), 8408–8415, doi:10.1021/acs.est.5b01944, 2015.

Zhang, Z. H., Khlystov, A., Norford, L. K., Tan, Z. K. and Balasubramanian, R.: Characterization of traffic-related ambient fine particulate matter (PM2.5) in an Asian city: Environmental and health implications, Atmos. Environ., 161, 132–143, doi:10.1016/j.atmosenv.2017.04.040, 2017b.

Zíková, N., Wang, Y., Yang, F., Li, X., Tian, M. and Hopke, P. K.: On the source contribution to Beijing PM2.5 concentrations, Atmos. Environ., 134, 84–95, doi:10.1016/j.atmosenv.2016.03.047, 2016.
